# Arene dearomatization through a catalytic N-centered radical cascade reaction

**DOI:** 10.1038/s41467-020-16369-4

**Published:** 2020-05-20

**Authors:** Rory C. McAtee, Efrey A. Noten, Corey R. J. Stephenson

**Affiliations:** Willard Henry Dow Laboratory, Department of Chemistry, University of Michigan, 930 North University Ave., Ann Arbor, MI 48109 USA

**Keywords:** Photocatalysis, Synthetic chemistry methodology

## Abstract

Arene dearomatization reactions are an important class of synthetic technologies for the rapid assembly of unique chemical architectures. Herein, we report a catalytic protocol to initiate a carboamination/dearomatization cascade that proceeds through transient sulfonamidyl radical intermediates formed from native sulfonamide N–H bonds leading to 1,4-cyclohexadiene-fused sultams. Importantly, this work demonstrates a facile approach to employ two-dimensional aromatic compounds as modular building blocks to generate richly substituted, three-dimensional compounds. These reactions occur at room temperature under visible light irradiation and are catalyzed by the combination of an iridium(III) photocatalyst and a dialkyl phosphate base. Reaction optimization, substrate scope, mechanistic features, and synthetic applications of this transformation are presented.

## Abstract (web summary)

Arene dearomatization reactions allow chemists to rapidly build unique chemical architectures from largely available starting materials. Here, the authors show a photocatalytic carboamination/dearomatization cascade process leading to 1,4-cyclohexadiene-fused sultams via N-centered radicals.

## Introduction

**Fig. 1:**
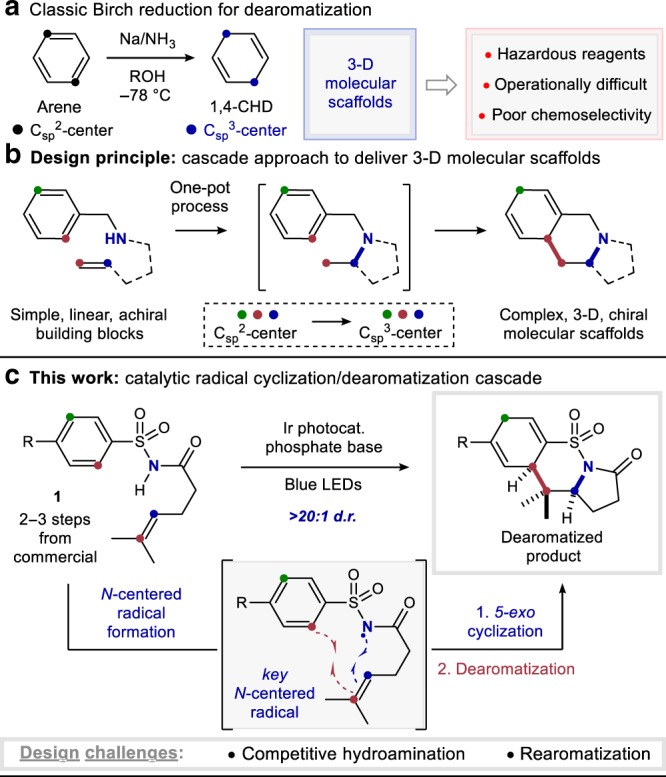
Inspiration and design for our carboamination/dearomatization cascade reaction platform. **a** The classic Birch reduction for arene dearomatization. **b** The design principle of using a radical cascade approach for arene dearomatization. **c** This work: catalytic radical carboamination/dearomatization cascade reactions.

Arenes obtained from inexpensive petrochemical feedstocks are incorporated on industrial scale into pharmaceuticals, agrochemicals, and organic materials. Selective dearomatization reactions^1,2^ of these flat, two-dimensional aromatic compounds are modular strategies to access substituted, three-dimensional chemical space^3^, including fused, complex heterocyclic skeletons^4,5^. In addition, the potential to form stereogenic centers through substituent addition concomitant with the dearomatization process is particularly appealing. Of the selective dearomatization methods reported (including UV-promoted photochemical cycloadditions^6,7^, oxidative^8^, enzymatic^9–11^, transition metal-mediated^12–14^, and nucleophilic dearomatizations^15^), the Birch reduction of arenes to 1,4-cyclohexadienes (1,4-CHD) is most well-known relying on liquid ammonia as solvent and pyrophoric alkali metals at cryogenic temperatures (Fig. 1a)^16,17^. Modifications of the canonical Birch reduction conditions have expanded the scope and synthetic utility of the reaction^18–22^. Although both electrochemical^23^ and visible light-mediated^24–28^ arene dearomatization reactions have been reported with proven utility, they have not been integrated with other reaction pathways via reactive intermediates. A complementary strategy addressing the inherent drawbacks of the classic Birch reduction is to promote an arene dearomatization via a radical cascade sequence. Radical cascades, processes in which multiple chemical bonds are formed in a single operation, are step and atom-economical means to rapidly build complex organic molecules (Fig. 1b)^29–31^. Moreover, initiating cascade sequences from strong N–H bonds leading to dearomatized molecular frameworks has the potential to impact a variety of synthetic endeavors.

Multiple-site concerted proton-electron transfer (MS-CPET) has emerged as a powerful strategy for homolytic activation of strong N–H bonds, often in the presence of weaker ones, avoiding the need for *N*-pre-functionalization^32–34^. The Knowles group recently reported the activation of both amide^35^ and sulfonamide^36^ N–H bonds for intramolecular and intermolecular hydroamination reactions, respectively, of electron-neutral olefins. The authors propose an excited-state redox catalyst and a weakly coordinating phosphate base, jointly mediate the concerted homolytic activation of the strong N–H bonds under visible light irradiation to afford transient *N*-centered amidyl and sulfonamidyl radicals capable of adding to olefins with *anti*-Markovnikov regioselectivity. Separately, Chu and Rovis^37^ described the generation of amidyl radicals from trifluoroacetamide derivatives for sequential 1,5-HAT and δ-C–H functionalization. By using superstoichiometric K_3_PO_4_ as the base, trifluoroacetamides are deprotonated to the amidyl anions then oxidized to the amidyl radical by the excited state of an iridium(III) photocatalyst.

With respect to sulfonamide-based *N*-radical alkene carboamination platforms, Kinoshita and co-workers^38^ demonstrated that *N*-phenylbenzenesulfonamidyl radicals derived from homolysis of a symmetrical sulfonylhydrazide could add to alkenes to give mixtures of products, including sultams. Chemler and co-workers have developed racemic and enantioselective intramolecular Cu(II)-mediated oxidative *N*-radical cyclizations of alkenyl arylsulfonamides at elevated temperatures (120 °C) to garner benzosultams in good yields^39–41^. Later, Kanai and co-workers^42^ reported an intermolecular alkene carboamination reaction of aliphatic alkenes under Cu(I) catalysis and *N*-fluorobenzenesulfonimide (NFSI) as both a bifunctional reagent, for C–C and C–N bond formation, and an oxidant for the concise synthesis of six-membered sultams. Zhao et al.^43^ have disclosed a radical cascade cyclization/allylation of β,γ-unsaturated *N*-aryl hydrazones that gives dihydropyrazoles and tetrahydropyridazines. Very recently, Zhao et al.^44^ have also described a process for a radical 5-*exo* cyclization/addition/aromatization cascade of β,γ-unsaturated *N*-tosyl hydrazones under cooperative photocatalysis and cobalt catalysis enabling the synthesis of dihydropyrazole-fused benzosultams.

We reasoned that the work outlined above, along with our ongoing interests in alkene carboamination reactions with bifunctional arylsulfonamide reagents^45^, would serve as a basis for developing a catalytic protocol to initiate a carboamination/dearomatization cascade starting from γ,δ-unsaturated *N*-arylsulfonyl enamides **1** (Fig. 1c). Ideally, this process would proceed through a transient sulfonamidyl radical intermediate originating from formal removal of the N–H hydrogen atom. This strategy obviates the need for *N*-pre-functionalization, elevated reaction temperatures, and stoichiometric oxidants. The reaction would lead to the selective synthesis of 1,4-cyclohexadiene-fused sultams. Of note, fused sultams have been shown to exhibit a broad range of biological activities^46–48^.

At the outset, we recognized two major challenges in implementing this design strategy. The first was the potential for competitive hydroamination, wherein the vicinal carbon-centered radical that is formed following *N*-radical cyclization would undergo premature hydrogen atom transfer or reduction to give the hydroamination product^33,35,36,49–54^. The second challenge was rearomatization of the cyclohexadienyl radical intermediate. To circumvent these challenges, it was necessary to identify an efficient photocatalytic system capable of selectively reducing the cyclohexadienyl radical to the cyclohexadienyl anion and reestablish the ground state of the photocatalyst. We believed such challenges could be surmounted with judicious choice of reaction additives and parameters. Herein, we describe the combination of the aforementioned reaction classes, whereby visible light-mediated photoredox catalysis is used to promote and control a sulfonamidyl radical cyclization/dearomatization cascade reaction.

## Results

### Reaction optimization

Initially, we chose arylsulfonamide **1a** as the model substrate to test the feasibility of the visible light-induced carboamination/dearomatization cascade reaction and representative results are summarized in Table 1. The expected reaction occured with 1 mol% Ir-photocatalyst **A** ([Ir(dF(CF_3_ppy)_2_)(5,5′-CF_3_-bpy)]PF_6_, Ir^III*/II^ = 1.68 V versus SCE in MeCN) and 65 mol% of tetrabutylammonium dibutylphosphate base providing the desired 1,4-cyclohexadiene-fused sultam **2a** in 48% ^19^F-NMR yield and excellent diastereoselectivity (>20:1) following irradiation with blue LEDs in trifluorotoluene (0.2 M) at room temperature (entry 1). Dearomatized product **2a** was unambiguously characterized by single-crystal X-ray analysis. Other iridium photocatalysts structurally similar to **A** (**B** = [Ir(dF(CF_3_ppy)_2_)(dtbbpy)]PF_6_, Ir^III*/II^ = 1.21 V versus SCE in MeCN; **C** = [Ir(dF(Meppy)_2_)(dtbbpy)]PF_6_, Ir^III*/II^ = 0.97 V versus SCE in MeCN)^55,56^ were also effective in these reactions (entries 2, 3), however the reaction yields diminished as the oxidation potential of the excited-state species decreased. Importantly, decreasing the reaction concentration (0.05 M) delivered the desired product in 67% yield (entry 4). Also, the reaction is moderately to equally successful in other solvents (entry 5-7) with *tert*-butanol providing a noticeable increase in yield (entry 8). A 1:1 mixed solvent system of trifluorotoluene/*tert*-butanol (0.05 M) was finally identified as the optimal solvent combination (entry 9). When the reactions were run using conditions developed by Rovis, relying on step-wise deprotonation/oxidation^37^, no product was isolated (See Supplementary Tables 1 and 2 for complete optimization details). Control reactions of **1a** lacking photocatalyst, base, or light were uniformly unsuccessful, and the starting material was recovered unchanged (entries 10–12).

**Table 1:** Reaction optimization and control experiments.


Entry	Photocatalyst	Solvent	Yield (%)
1	A	PhCF_3_ (0.2 M)	48
2	B	PhCF_3_ (0.2 M)	32
3	C	PhCF_3_ (0.2 M)	7
4	A	PhCF_3_ (0.05 M)	67
5	A	DMF (0.05 M)	50
6	A	CH_2_Cl_2_ (0.05 M)	47
7	A	1,2-DCE (0.05 M)	65
8	A	*t*-BuOH (0.05 M)	73
9	A	PhCF_3_/*t*-BuOH (0.05 M)	83 (75)^a^

Variation from best conditions (Entry 9)		
10	No blue LEDs	0
11	No photocatalyst	0
12	No NBu_4_OP(O)(OBu)_2_	0

See Supplementary Tables 1 and 2 for complete optimization details. Reactions were run on 0.1 mmol scale, and yields are determined by ^19^F-NMR analysis relative to 1,3,5-trifluorobenzene (1 equiv.) as an internal standard.

^a^Isolated yield of **2a** on 0.2 mmol scale.

### Substrate scope

**Fig. 2:**
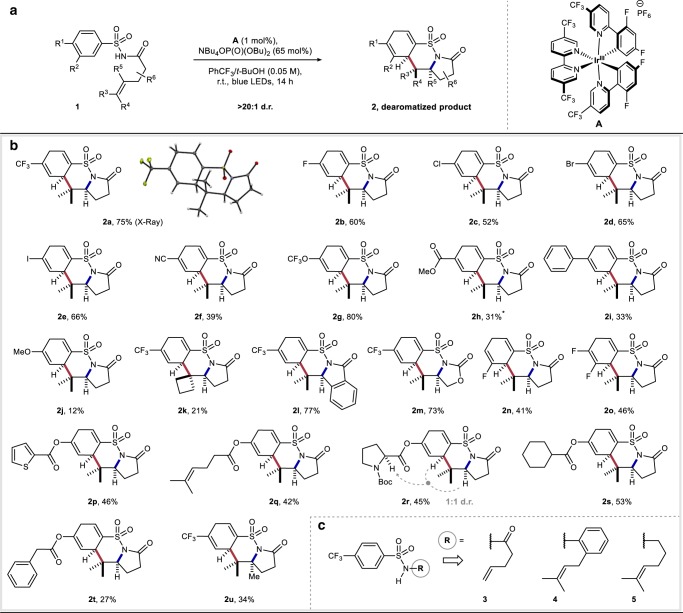
Reaction scope. All yields are isolated yields. Relative configurations of products were assigned by analogy to **2a**. **a** General reaction scheme; all reactions were run on 0.2 mmol scale and degassed by sparging with argon for 15 min prior to exposure to optimized conditions. **b** Arylsulfonamide modifications. **c** Control experiments with differentially substituted alkene side-chains. *Product isolated as an inseparable mixture of diene and arene products (3:1, diene:arene).

With optimized conditions established, we investigated the generality of this carboamination/dearomatization cascade reaction with a series of diversely substituted arylsulfonamides (Fig. 2). For example, arenes bearing various electron-withdrawing (**2a**, **2b**, **2f**–**2i**), electron-neutral (**2c**–**2e**), and electron-donating (**2j**) groups delivered the desired dearomatized products in moderate to good yields (12–80%) and with excellent diastereoselectivity (>20:1). Unsurprisingly, the latter electron-rich product **2j** was prone to rapid oxidative decomposition leading to diminished yields. Dearomatized product **2h** was isolated as an inseparable mixture with the aromatized benzosultam product. Next, we investigated modifications to the alkene tether which allowed for the synthesis of an all-carbon spirocycle (**2k**), fused tetracycle (**2l**), and cyclic carbamate (**2m**) in moderate to good yields (21–77%). Interestingly, 3-fluoro and 3,4-difluoro substituted arylsulfonamide substrates generated single dearomatized regioisomers in good yields (**2n**, **2o**) highlighting the regio- and chemoselective nature of the C–C bond forming cyclization step. Furthermore, this methodology proved tolerant of diverse functionality including electron-rich heterocycles (**2p**), olefins (**2q**), amino acids (**2r**), aliphatic carbocycles (**2s**), and benzyl groups (**2t**) selectively furnishing the corresponding dearomatized products in good yields. Lastly, a tetrasubstituted olefin substrate (**1u**) allowed direct access to dearomatized *tert*-alkylamine^57^ product **2u**, suggesting the initial 5-*exo N*-radical cyclization is tolerant to steric encumbrance. Terminal olefin **3** failed to convert to the desired dearomatized product (Fig. 2c) likely due to the instability of the resultant vicinal primary radical following C–N bond formation. To probe the importance of the carbonyl moiety and to determine whether it is a necessary functionality for the reaction to proceed, we prepared and subjected *o*-prenyl aniline derivative **4** and alkyl sulfonamide **5** to the optimized reaction conditions and found they were unreactive, returning the starting material. These experiments highlight the potential importance of the carbonyl moiety for this process to occur by lowering the p*K*_a_ of the substrate (pK_a_ for **1** ≈ 5)^58^.

### Synthetic applications

**Fig. 3:**
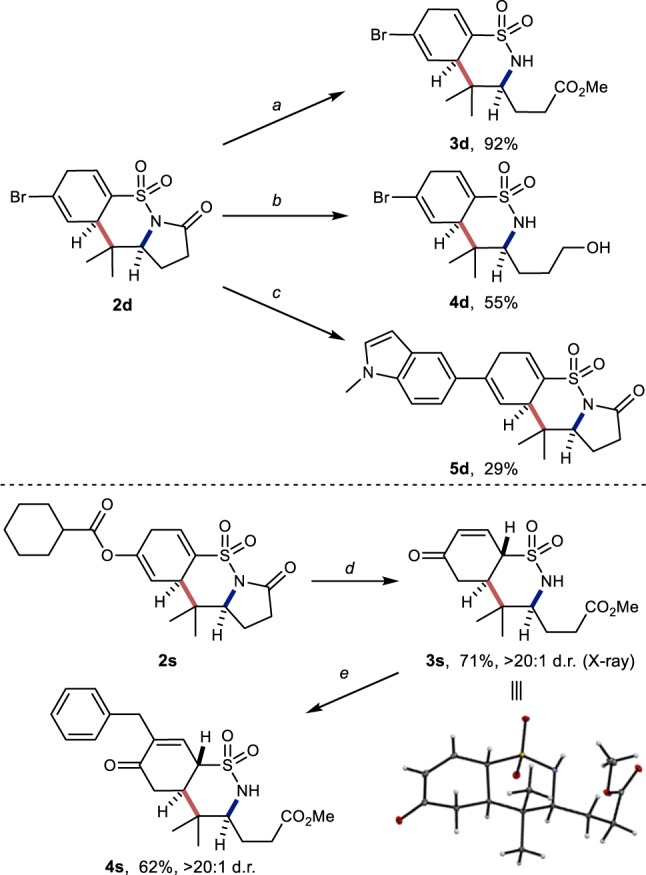
Synthetic modifications of fused-sultam products. All yields are isolated yields. Conditions: **a** Sat. aq. K_2_CO_3_, MeOH, rt, 15 min. **b** LiAlH_4_, THF, −78 °C to room temp., 1.5 h. **c** 1-(methylindol-5-yl)boronic acid, CsF, PPh_3_, Pd(OAc)_2_, THF, reflux, 14 hr. **d** K_2_CO_3_, MeOH, rt, 15 min. **e** K_2_CO_3_, benzyl bromide, DMF, rt, 2 h.

The richly functionalized 1,4-cyclohexadiene-fused sultams accessible with this methodology can be readily diversified, leveraging the functional groups tolerated by the dearomative cyclization (Fig. 3). The *N*-sulfonyl lactam carbonyl is susceptible to nucleophilic addition and subsequent displacement of the sulfonamide leaving group. Thus, **2d** is rapidly converted to bicyclic methyl ester **3d** at room temperature in excellent yield when treated with excess K_2_CO_3_ in methanol. Reaction of **2d** with lithium aluminum hydride in THF directly provides the bicyclic alcohol **4d**. Of note, **3d** and **4d** are the products of the formal intermolecular dearomative reaction between a primary sulfonamide *N*-centered radical and a *γ,δ*-unsaturated ester or a *bis*-homoallylic alcohol, respectively. Palladium-catalyzed Suzuki–Miyaura cross-coupling of **2d** with *N*-methylindole-5-boronic acid under unoptimized anhydrous conditions gave a moderate yield of alkenyl indole **5d**. Under similar conditions used to prepare **3d**, tricyclic sultam **2s** containing alkenyl ester functionality converts in good yield to the *γ*-sulfamoyl enone **3s** as a single diastereomer as revelaed by X-ray crystallographic analysis. Due to the resonance stabilizing effects of the enone on the resulting anion, **3s** is deprotonated *alpha* to the sulfonamide by K_2_CO_3_ to give a dienolate that is alkylated by benzyl bromide *alpha* to the carbonyl. Enone **4s** is thus isolated following re-conjugation of the remaining alkene with the ketone. *N*-benzylated products were not observed.

### Mechanistic investigations

**Fig. 4:**
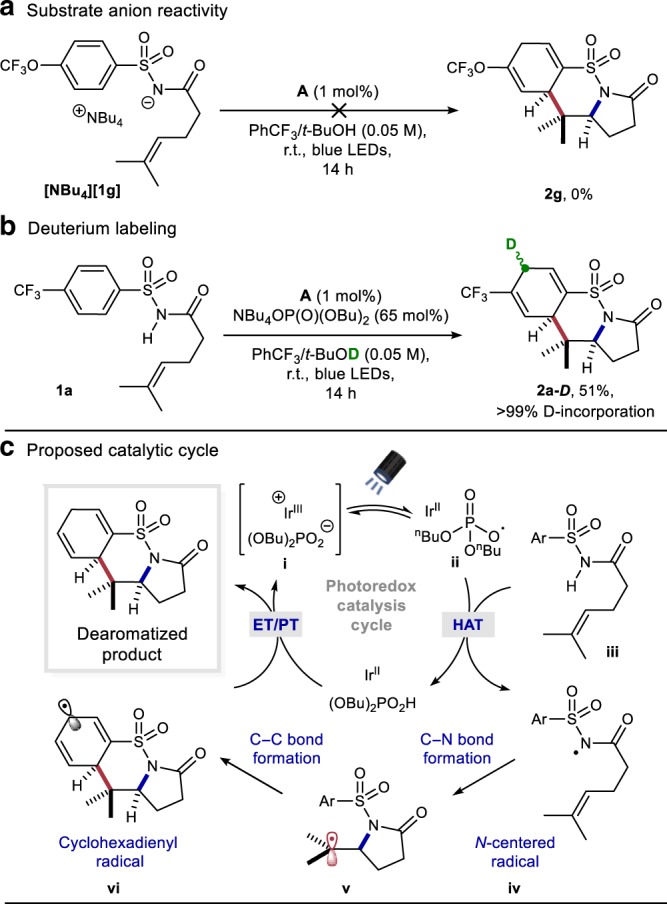
Experiment to probe mechanism and proposed catalytic cycle. **a** Deprotonated substrate is unreactive under reaction conditions. **b** Deuterium labeling experiment. **c** Proposed catalytic cycle.

To gain insight into the reaction mechanism, we performed Stern–Volmer luminescence quenching studies with the model substrate **1a** (Supplementary Figs. 73–76). It was revealed that there is no luminescence quenching of the photoexcited state of catalyst **A** when **1a** is used alone, suggesting that direct oxidation, followed by deprotonation, of the substrate is unlikely. This conclusion was further supported by cyclic voltammetry analysis (CH_2_Cl_2_ containing 0.1 M NBu_4_PF_6_) indicating that direct oxidation of substrate **1a** occurs at potentials >1.6 V (versus SCE) (Supplementary Fig. 77). Comparatively, when tetrabutylammonium dibutylphosphate base is used alone in the Stern–Volmer analysis, significant nonlinear photoluminescence quenching of the photoexcited state of catalyst **A** is observed. This observation aligns with the reports of Knowles^59^, and others^60^, suggesting the formation of a less emissive iridium-phosphate complex which may be the active ground-state catalytic species in solution. Importantly, no additional photoluminescence quenching is observed upon addition of substrate **1a** as one would expect for a MS-CPET process. Cyclic voltammetry studies of **1a** in the presence of varying concentrations of monobasic dibutylphosphate base revealed that current response increased for increasing concentrations of phosphate base but did not show a shifted, less positive potential for the oxidation of substrate **1a** (Supplementary Fig. 77). With no evidence for a MS-CPET process, we next investigated whether sequential sulfonamide deprotonation and one-electron oxidation was the operative mechanism for *N*-centered radical generation. Deprotonation of **1g** with sodium hydride followed by salt metathesis gave the sulfonamidyl anion product **[NBu**_**4**_**][1g]**. When this compound was exposed to the typical reaction conditions in the absence of phosphate, product **2g** was not detected and only starting material was observed by NMR analysis of the crude reaction (Fig. 4a). This result suggests that single-electron oxidation of a sulfonamidyl anion is not the dominant route to the nitrogen-centered radical. Qualitatively, these results together are consistent with a phosphate radical being generated under the reaction conditions and serving as a hydrogen-atom abstracting species and is at least partly responsible for the generation of the nitrogen radical^61,62^. HAT between heteroatoms is known to occur rapidly, even in the presence of weaker C–H bonds^63^. Next, a deuterium labeling experiment was conducted to probe the origin of the additional proton on the carbocycle in the products. By performing the reaction in the presence of *tert*-BuOD, the desired product (**2a-*D***) was produced in 51% yield with complete deuterium incorporation (Fig. 4b). This result suggests that the *tert*-BuOH is serving as a reaction-terminating proton source of a 1,4-cyclohexadienyl anion intermediate in accord with previous mechanistic studies of the classic Birch reduction reaction^64,65^. Interestingly, the mechanism for sulfonamidyl radical formation presented herein differs from the proposal by Knowles and co-workers^36^. This observed divergence may be due to differences in acidity between the primary benzenesulfonamides used by Knowles and the *N*-carbonyl benzenesulfonamides used in this study.

### Proposed catalytic cycle

A catalytic cycle accounting for these observations, and those made during reaction optimization, is shown in Fig. 4c. We suspect that the Ir-photocatalyst **A** and phosphate base first form a ground-state iridium-phosphate complex (**i**). Upon excitation, the Ir-photocatalyst can undergo single-electron transfer from the phosphate salt, generating an oxygen-centered radical (**ii**). Abstraction of the most activated H-atom of the substrate (**iii**) generates a transient sulfonamidyl radical intermediate (**iv**). Next, the *N*-radical can undergo 5-*exo* cyclization onto the tethered alkene to furnish a new C–N bond and a vicinal carbon-centered radical (**v**). This alkyl radical is poised to undergo cyclization with the appended arene to generate a stabilized cyclohexadienyl radical (**vi**) that can undergo single-electron reduction by the reduced Ir(II) state of the photocatalyst (*E*_*1/2*_[Ir^III^/Ir^II^]= −1.07 V versus Fc^+^/Fc)^36^. Favorable proton transfer between the cyclohexadienyl anion and *tert*-BuOH (*tert*-butoxide can then return dibutyl phosphoric acid^66^ back to the phosphate salt) should deliver the dearomatized product and regenerate both catalysts.

## Discussion

In summary, we have reported the discovery and development of a photoredox-mediated *N*-centered radical strategy to facilitate a carboamination/dearomatization cascade reaction. Simple γ,δ-unsaturated *N*-arylsulfonyl enamides were converted into complex and stereodefined 1,4-cyclohexadiene-fused sultams in satisfactory yields and excellent diastereoselectivity. This mild and efficient catalytic system demonstrates a broad substrate scope and high functional group tolerance while avoiding premature hydroamination or undesired rearomatization reactivity. Overall, we believe the photochemical strategy outlined here will inspire future synthetic endeavors aimed at employing simple arene building blocks for the rapid synthesis of complex, three-dimensional molecular frameworks in a single operation.

## Methods

### Representative procedure

To a dry 2-dram vial was added γ,δ-unsaturated *N*-arylsulfonyl enamide **1** (0.2 mmol), base, (65 mol%), and photocatalyst (1 mol%). The vial contents were then dissolved in a 1:1 mixture of *t*-BuOH:PhCF_3_ (0.05 M). The solution was degassed by sparging with argon for 15 min. The reaction was irradiated with two, H150 blue Kessil lamps positioned ~2 cm away and cooled with an overhead fan for 14 h. The reaction was concentrated in vacuo, loaded onto silica, and purified by flash column chromatography to afford the desired 1,4-cyclohexadiene-fused sultam **2**. No unexpected or unusually high safety hazards were encountered.

## Supplementary information

Supplementary Information

## Data Availability

Experimental data as well as ^1^H and ^13^C NMR spectra for all new compounds prepared in the course of these studies are provided in the Supplementary Information of this manuscript. The solid-state X-ray crystal structures of **2a** and **3s** are available free of charge from the Cambridge Crystallographic Data Centre under reference numbers CCDC 1952459 and CCDC 1984122, respectively. These data can be obtained free of charge from The Cambridge Crystallographic Data Center via www.ccdc.cam.ac.uk/data_request/cif. All other data including synthetic procedures are available in the Supplementary Information file.

## References

[1] Wertjes WC, Southgate EH, Sarlah D (2018). Recent advances in chemical dearomatization of nonactivated arenes. Chem. Soc. Rev..

[2] Zhuo C-X, Zhang W, You S-L (2012). Catalytic asymmetric dearomatization reactions. Angew. Chem. Int. Ed..

[3] Lovering F, Bikker J, Humblet C (2009). Escape from flatland: increasing saturation as an approach to improving clinical success. J. Med. Chem..

[4] Roche, S. P. & Porco, J. A. Jr. Dearomatization strategies in the synthesis of complex natural products. *Angew. Chem. Int. Ed.* **50**, 4068–4093. 10.1002/anie.201006017 PMC4136767 21506209

[5] Przydacz A, Skrzyńska A, Albrecht Ł (2019). Breaking aromaticity with aminocatalysis: a convenient strategy for asymmetric synthesis. Angew. Chem. Int. Ed..

[6] Wagner PJ, Sakamoto M (1989). Intramolecular triplet state cyclization of but-3-enoxyacetonaphthones. J. Am. Chem. Soc..

[7] Wagner PJ (2001). Photoinduced ortho [2 + 2] cycloaddition of double bonds to triplet benzenes. Acc. Chem. Res..

[8] Pouységu L, Deffieux D, Quideau S (2010). Hypervalent iodine-mediated phenol dearomatization in natural product synthesis. Tetrahedron.

[9] Hudlicky T, Reed JW (2009). Celebrating 20 years of SYNLETT—special account on the merits of biocatalysis and the impact of arene *cis*-dihydrodiols on enantioselective synthesis. Synlett.

[10] Boyd DR, Bugg TDH (2006). Arene *cis*-dihydrodiol formation: from biology to application. Org. Biomolecular Chem..

[11] Boll M (2005). Dearomatizing benzene ring reductases. J. Mol. Microbiol. Biotechnol..

[12] Pape AR, Kaliappan KP, Kündig EP (2000). Transition-metal-mediated dearomatization reactions. Chem. Rev..

[13] Giustra ZX, Ishibashi JSA, Liu S-Y (2016). Homogeneous metal catalysis for conversion between aromatic and saturated compounds. Coord. Chem. Rev..

[14] Zheng C, You S-L (2016). Catalytic asymmetric dearomatization by transition-metal catalysis: a method for transformations of aromatic compounds. Chem.

[15] López Ortiz F, Iglesias MJ, Fernández I, Andújar Sánchez CM, Ruiz Gómez G (2007). Nucleophilic dearomatizing (DNAr) reactions of aromatic C,H-systems. A mature paradigm in organic synthesis. Chem. Rev..

[16] Birch AJ (1950). The reduction of organic compounds by metal-ammonia solutions. Q. Rev. Chem. Soc..

[17] Holy NL (1974). Reactions of the radical anions and dianions of aromatic hydrocarbons. Chem. Rev..

[18] Dye JL (2005). Alkali metals plus silica gel: powerful reducing agents and convenient hydrogen sources. J. Am. Chem. Soc..

[19] Lei P (2018). A practical and chemoselective ammonia-free Birch reduction. Org. Lett..

[20] Donohoe TJ, Thomas RE (2007). The partial reduction of electron-deficient pyrroles: procedures describing both Birch (Li/NH3) and ammonia-free (Li/DBB) conditions. Nat. Protoc..

[21] Yoo BI, Kim YJ, You Y, Yang JW, Kim SW (2018). Birch reduction of aromatic compounds by inorganic electride [Ca2N]+·e– in an alcoholic solvent: an analogue of solvated electrons. J. Org. Chem..

[22] Szostak M, Spain M, Procter DJ (2014). Determination of the effective redox potentials of SmI2, SmBr2, SmCl2, and their complexes with water by reduction of aromatic hydrocarbons. Reduction of anthracene and stilbene by samarium(II) iodide–water complex. J. Org. Chem..

[23] Peters BK (2019). Scalable and safe synthetic organic electroreduction inspired by Li-ion battery chemistry. Science.

[24] Southgate EH, Pospech J, Fu J, Holycross DR, Sarlah D (2016). Dearomative dihydroxylation with arenophiles. Nat. Chem..

[25] Southgate EH, Holycross DR, Sarlah D (2017). Total synthesis of lycoricidine and narciclasine by chemical dearomatization of bromobenzene. Angew. Chem. Int. Ed..

[26] Hernandez LW, Pospech J, Klöckner U, Bingham TW, Sarlah D (2017). Synthesis of (+)-pancratistatins via catalytic desymmetrization of benzene. J. Am. Chem. Soc..

[27] Okumura M, Shved AS, Sarlah D (2017). Palladium-catalyzed dearomative syn-1,4-carboamination. J. Am. Chem. Soc..

[28] James MJ, Schwarz JL, Strieth-Kalthoff F, Wibbeling B, Glorius F (2018). Dearomative cascade photocatalysis: divergent synthesis through catalyst selective energy transfer. J. Am. Chem. Soc..

[29] Sebren LJ, Devery JJ, Stephenson CRJ (2014). Catalytic radical domino reactions in organic synthesis. ACS Catal..

[30] Skubi KL, Blum TR, Yoon TP (2016). Dual catalysis strategies in photochemical synthesis. Chem. Rev..

[31] Plesniak MP, Huang H-M, Procter DJ (2017). Radical cascade reactions triggered by single electron transfer. Nat. Rev. Chem..

[32] Yayla HG, Knowles RR (2014). Proton-coupled electron transfer in organic synthesis: novel homolytic bond activations and catalytic asymmetric reactions with free radicals. Synlett.

[33] Miller DC, Tarantino KT, Knowles RR (2016). Proton-coupled electron transfer in organic synthesis: fundamentals, applications, and opportunities. Top. Curr. Chem..

[34] Gentry EC, Knowles RR (2016). Synthetic applications of proton-coupled electron transfer. Acc. Chem. Res..

[35] Choi GJ, Zhu Q, Miller DC, Gu CJ, Knowles RR (2016). Catalytic alkylation of remote C–H bonds enabled by proton-coupled electron transfer. Nature.

[36] Zhu Q, Graff DE, Knowles RR (2018). Intermolecular anti-Markovnikov hydroamination of unactivated alkenes with sulfonamides enabled by proton-coupled electron transfer. J. Am. Chem. Soc..

[37] Chu JCK, Rovis T (2016). Amide-directed photoredox-catalysed C–C bond formation at unactivated sp3 C–H bonds. Nature.

[38] Miura Y, Ohnishi T, Kinoshita M, Kinoshita T (1990). Addition of a sulfonamidyl radical to unsaturated aromatic and aliphatic hydrocarbons. Bull. Chem. Soc. Jpn..

[39] Sherman ES, Chemler SR, Tan TB, Gerlits O (2004). Copper(II) acetate promoted oxidative cyclization of arylsulfonyl-*o*-allylanilines. Org. Lett..

[40] Zeng W, Chemler SR (2007). Copper(II)-catalyzed enantioselective intramolecular carboamination of alkenes. J. Am. Chem. Soc..

[41] Chemler SR (2009). The enantioselective intramolecular aminative functionalization of unactivated alkenes, dienes, allenes and alkynes for the synthesis of chiral nitrogen heterocycles. Org. Biomol. Chem..

[42] Kaneko K, Yoshino T, Matsunaga S, Kanai M (2013). Sultam synthesis via Cu-catalyzed intermolecular carboamination of alkenes with N-fluorobenzenesulfonimide. Org. Lett..

[43] Zhao Q-Q, Chen J, Yan D-M, Chen J-R, Xiao W-J (2017). Photocatalytic hydrazonyl radical-mediated radical cyclization/allylation cascade: synthesis of dihydropyrazoles and tetrahydropyridazines. Org. Lett..

[44] Zhao Q-Q, Hu X-Q, Yang M-N, Chen J-R, Xiao W-J (2016). A visible-light photocatalytic N-radical cascade of hydrazones for the synthesis of dihydropyrazole-fused benzosultams. Chem. Commun..

[45] Monos TM, McAtee RC, Stephenson CRJ (2018). Arylsulfonylacetamides as bifunctional reagents for alkene aminoarylation. Science.

[46] Wells GJ, Tao M, Josef KA, Bihovsky R (2001). 1,2-Benzothiazine 1,1-dioxide P2–P3 peptide mimetic aldehyde calpain I inhibitors. J. Medicinal Chem..

[47] Xie Y (2008). Convenient preparation of N-8-quinolinyl benzenesultams as novel NF-κB inhibitors. Tetrahedron Lett..

[48] Silver LH (1998). Clinical efficacy and safety of brinzolamide (Azopt™), a new topical carbonic anhydrase inhibitor for primary open-angle glaucoma and ocular hypertension. Am. J. Ophthalmol..

[49] Musacchio AJ (2017). Catalytic intermolecular hydroaminations of unactivated olefins with secondary alkyl amines. Science.

[50] Miller DC, Choi GJ, Orbe HS, Knowles RR (2015). Catalytic olefin hydroamidation enabled by proton-coupled electron transfer. J. Am. Chem. Soc..

[51] Nguyen TM, Manohar N, Nicewicz DA (2014). Anti-Markovnikov hydroamination of alkenes catalyzed by a two-component organic photoredox system: direct access to phenethylamine derivatives. Angew. Chem. Int. Ed..

[52] Morse PD, Nicewicz DA (2015). Divergent regioselectivity in photoredox-catalyzed hydrofunctionalization reactions of unsaturated amides and thioamides. Chem. Sci..

[53] Chen J, Guo H-M, Zhao Q-Q, Chen J-R, Xiao W-J (2018). Visible light-driven photocatalytic generation of sulfonamidyl radicals for alkene hydroamination of unsaturated sulfonamides. Chem. Commun..

[54] Lardy SW, Schmidt VA (2018). Intermolecular radical mediated anti-markovnikov alkene hydroamination using *N*-hydroxyphthalimide. J. Am. Chem. Soc..

[55] Prier CK, Rankic DA, MacMillan DWC (2013). Visible light photoredox catalysis with transition metal complexes: applications in organic synthesis. Chem. Rev..

[56] Teegardin K, Day JI, Chan J, Weaver J (2016). Advances in photocatalysis: a microreview of visible light mediated ruthenium and iridium catalyzed organic transformations. Org. Process Res. Dev..

[57] Trowbridge A, Reich D, Gaunt MJ (2018). Multicomponent synthesis of tertiary alkylamines by photocatalytic olefin-hydroaminoalkylation. Nature.

[58] Lassalas P (2016). Structure property relationships of carboxylic acid isosteres. J. Med. Chem..

[59] Morton CM (2019). C–H alkylation via multisite-proton-coupled electron transfer of an aliphatic C–H bond. J. Am. Chem. Soc..

[60] Ardo S, Sun Y, Castellano FN, Meyer GJ (2010). Excited-state electron transfer from ruthenium-polypyridyl compounds to anatase TiO_2_ nanocrystallites: evidence for a stark effect. J. Phys. Chem. B.

[61] Margrey KA, Czaplyski WL, Nicewicz DA, Alexanian EJ (2018). A general strategy for aliphatic C–H functionalization enabled by organic photoredox catalysis. J. Am. Chem. Soc..

[62] Wakaki T (2018). C(*sp*3)–H cyanation promoted by visible-light photoredox/phosphate hybrid catalysis. Chem. A Eur. J..

[63] Mayer JM (2011). Understanding hydrogen atom transfer: from bond strengths to Marcus theory. Acc. Chem. Res..

[64] Zimmerman HE, Wang PA (1990). Regioselectivity of the Birch reduction. J. Am. Chem. Soc..

[65] Zimmerman HE, Wang PA (1993). The regioselectivity of the Birch reduction. J. Am. Chem. Soc..

[66] Rueping M, Nachtsheim BJ, Ieawsuwan W, Atodiresei I (2011). Modulating the acidity: highly acidic brønsted acids in asymmetric catalysis. Angew. Chem. Int. Ed..

