# Supplementary material for: Arene dearomatization through a catalytic N-centered radical cascade reaction
Source: Nat Commun. 2020 May 20;11:2528. doi: 10.1038/s41467-020-16369-4 (PMC7239915; doi:10.1038/s41467-020-16369-4)
Supplement: Supplementary file 1 — Supplementary Information [file 41467_2020_16369_MOESM1_ESM.pdf]

Supplementary Information

for

Arene Dearomatization through a Catalytic *N*-Centered Radical Cascade Reaction

McAtee et al.

Supplementary Methods

General Considerations. All chemicals were used as received and stored as recommended by the supplier. Reactions were monitored by thin layer chromatography (TLC) using glass-backed plates pre-coated with 230–400 mesh silica gel (250 mm thickness) with fluorescent indicator F254, available from EMD Millipore (cat. #: 1.05715.0001). Plates were visualized with a dual short wave/long wave UV lamp. Column flash chromatography was performed using 230-400 mesh silica (SiliCycle cat. #: R12030B) gel or via automated column chromatography. NMR spectra were recorded on Varian MR400, Varian Inova 500, Varian Vnmrs 500, or Varian Vnmrs 700 spectrometers. Chemical shifts for ^{1}H NMR were reported as δ, parts per million, relative to the signal of $CHCl_3$ at 7.26 ppm and for DMSO 2.50 ppm. Chemical shifts for ^{13}C NMR were reported as δ, parts per million, relative to the center line signal of the $CDCl_3$ triplet at 77.16 ppm and for DMSO 39.52 ppm for center of septet. ^{19}F NMR chemical shifts were reported as δ, parts per million, relative to $CFCl_3$ at 0.0 ppm. The abbreviations s, br. s, d, dd, br. d, ddd, t, q, br. q, qi, sext, m, and br. m stand for the resonance multiplicity singlet, broad singlet, doublet, doublet of doublets, broad doublet, doublet of doublet of doublets, triplet, quartet, broad quartet, quintet, sextet, multiplet and broad multiplet, respectively. IR spectra were recorded on a Perkin-Elmer Spectrum BX FT-IR spectrometer fitted with an ATR accessory. Melting points were obtained using a Mel-Temp 3.0 (model no. 1401). Mass Spectra were recorded at the Mass Spectrometry Facility at the Department of Chemistry of the University of Michigan in Ann Arbor, MI on an Agilent Q-TOF HPCL-MS with ESI high resolution mass spectrometer using electrospray ionization (ESI), positive ion mode, or electron impact ionization (EI). Fluorescence quenching was recorded using a PTI Horiba Quanta Master using Felix GX software. We thank Dr. James Windak and Dr. Paul Lennon at the University of Michigan Department of Chemistry instrumentation facility for conducting these experiments. X-Ray crystallography work was done by Dr. Jeff. W. Kampf. UV-Vis measurements were obtained on a Shimadzu UV-1601 UV-Vis Spectrometer. Electrochemical data was collected on a CHI600E potentiostat with the accompanying CH Instruments software. H150 Blue grow lights from Kessil were used as the visible light irradiation source.

Unless stated otherwise, all reactions were run on a 0.2 mmol scale in a 2-dram vial equipped with an oval shaped stir bar. 2 x H150 Blue Kessil lamp sufficiently irradiated 1-3 reaction vials at one time, at ~5 cm away (**a**). At this distance, with an overhead fan dissipating the standing atmosphere, the air temperature surrounding the reactions did not exceed 25 ˚C. The reactions were stirred at a rate of ~550 rpms on an IKA magnetic stir plate. The photochemical dearomatization reactions were covered with ~0.5 m x 0.5 m dimension Blue Light Filter Amber Reaction Boxes (**b**) purchased from PLAS Labs, Inc (Lansing, MI). In addition, each experimentalist using blue light wore UVEX Skyper Orange Safety Glasses which were purchased through Amazon.

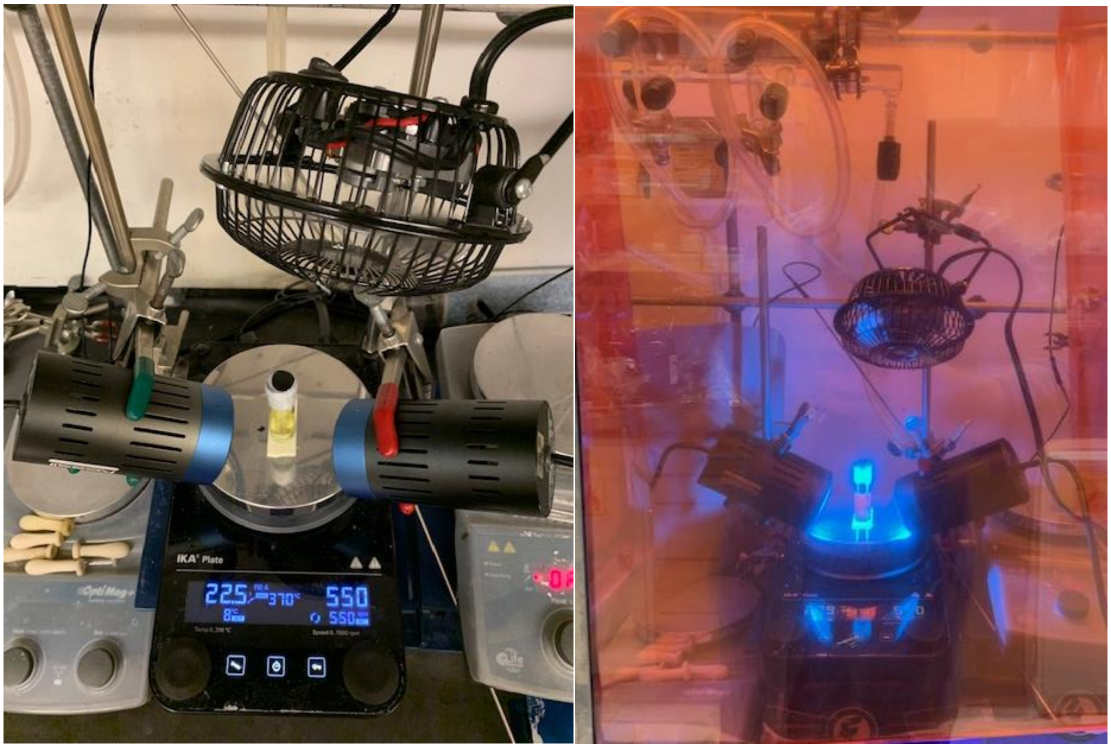

a **b**

Supplementary Figure 1. **a:** A typical reaction configuration. **b**: A typical reaction behind orange filter shielding.

Reaction optimization studies. To an oven-dried 1-dram vial was added **1a** (33.5 mg, 0.1 mmol), base, and photocatalyst. The vial contents were then dissolved in the indicated solvent or solvent mixture. The reaction solution was degassed by sparging with argon for 15 min. Then, the vial was quickly capped and sealed with parafilm. The reaction was irradiated with two, H150 blue Kessil lamps positioned ~5 cm away and cooled with an overhead fan. After 14 h, 1,3,5-trifluorobenzene was added to the reaction as a stoichiometric internal standard (10.3 µL, 0.1 mmol, 1 equiv). An aliquot was removed from the reaction vial and analyzed by ^{19}F NMR spectroscopy to determine the internal standard yield of **2a**.

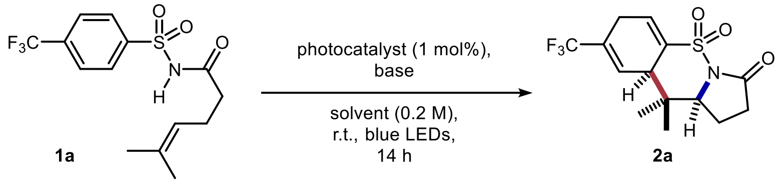

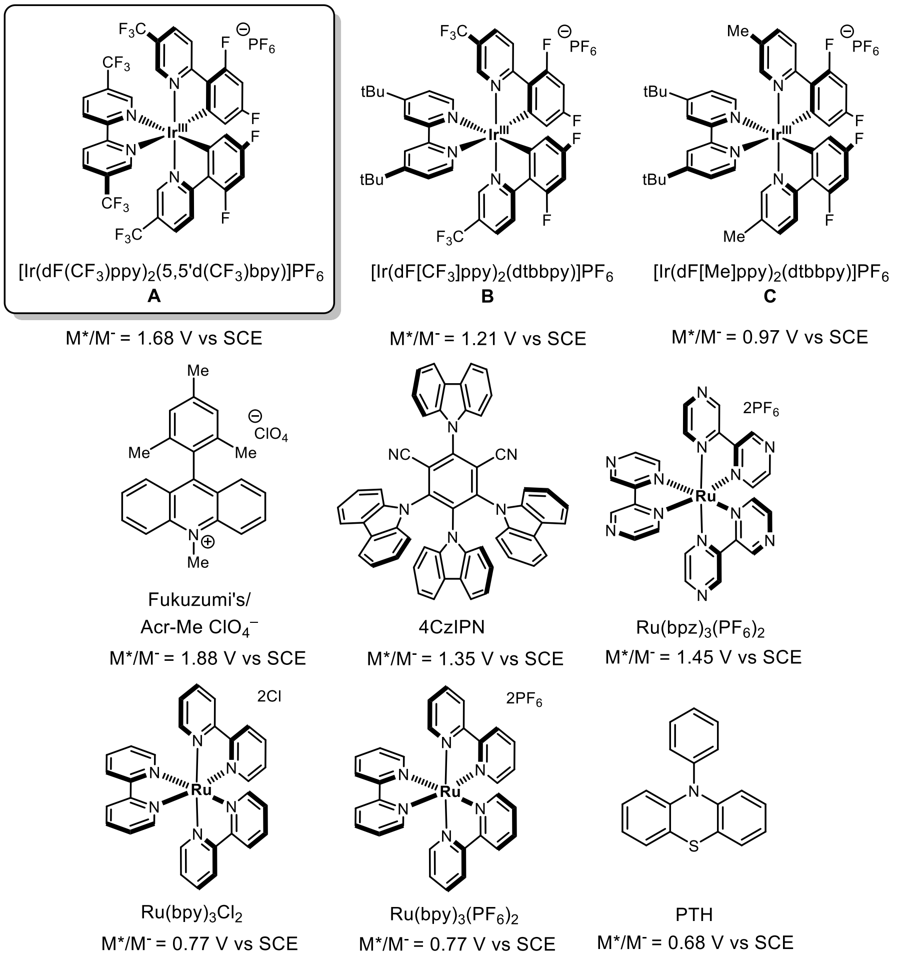

Supplementary Table 1. Photocatalyst optimization conditions and structures of all screened photocatalysts.[1-5]

entry	PC	base	base equiv	solvent	% yield [a]
1	A	no base	-	$PhCF_3$	<5
2	A	$Bu_4N[OP(O)(OBu)_2]$	0.2	$PhCF_3$	7
3	A	$Bu_4N[OP(O)(OBu)_2]$	0.4	$PhCF_3$	30
4	A	$Bu_4N[OP(O)(OBu)_2]$	0.65	$PhCF_3$	48
5	A	$Bu_4N[OP(O)(OBu)_2]$	0.85	$PhCF_3$	50
6	A	$Bu_4N[OP(O)(OBu)_2]$	1.0	$PhCF_3$	33
7	A	$Bu_4N[OP(O)(OBu)_2]$	1.3	$PhCF_3$	21
8	B	$Bu_4N[OP(O)(OBu)_2]$	0.65	$PhCF_3$	32
9	Fukuzumi's	$Bu_4N[OP(O)(OBu)_2]$	0.65	$PhCF_3$	5
10	C	$Bu_4N[OP(O)(OBu)_2]$	0.65	$PhCF_3$	7
11	4CzIPN	$Bu_4N[OP(O)(OBu)_2]$	0.65	$PhCF_3$	16
12	$Ru(bpz)_3(PF_6)_2$	$Bu_4N[OP(O)(OBu)_2]$	0.65	$PhCF_3$	0
13	PTH	$Bu_4N[OP(O)(OBu)_2]$	0.65	$PhCF_3$	0
14	$Ru(bpy)_3Cl_2$	$Bu_4N[OP(O)(OBu)_2]$	0.65	$PhCF_3$	0
15	$Ru(bpy)_3(PF_6)_2$	$Bu_4N[OP(O)(OBu)_2]$	0.65	$PhCF_3$	0
16	no catalyst	$Bu_4N[OP(O)(OBu)_2]$	0.65	$PhCF_3$	<5
17	A	Cs_2CO_3	1.5	$PhCF_3$	0
18	A	$PhCO_3K$	1.5	$PhCF_3$	8
19	A	quinuclidine	1.5	$PhCF_3$	0
20	A	K_3PO_4	1.5	$PhCF_3$	0
21	A	K_2CO_3	1.5	$PhCF_3$	0
22	A	NaOAc	1.5	$PhCF_3$	17
23	A	K_2HPO_4	1.5	$PhCF_3$	11
24	A	CsF	1.5	$PhCF_3$	3
25	A	KOH	1.5	$PhCF_3$	0
26	A	HMTA	1.5	$PhCF_3$	13
27	A	$CF_3(CO_2)K$	1.5	$PhCF_3$	7
28	A	DABCO	1.5	$PhCF_3$	18
29	A	$Bu_4N[OP(O)(OBu)_2]$	0.65	$PhCF_3$ (0.1 M)	59
30	A	$Bu_4N[OP(O)(OBu)_2]$	0.65	$PhCF_3$ (0.05 M)	67
31	A	$Bu_4N[OP(O)(OBu)_2]$	0.65	$PhCF_3$ (0.03 M)	66
32	A	$Bu_4N[OP(O)(OBu)_2]$	0.65	$PhCF_3$ (0.02 M)	56
33	A	$Bu_4N[OP(O)(OBu)_2]$	0.65	$PhCF_3$ (0.01 M)	17
34	A	$Bu_4N[OP(O)(OBu)_2]$	0.65	$PhCF_3$ (0.07 M)	56
35	A	$Bu_4N[OP(O)(OBu)_2]$	0.65	THF (0.05 M)	19
36	A	$Bu_4N[OP(O)(OBu)_2]$	0.65	Ether (0.05 M)	39
37	A	$Bu_4N[OP(O)(OBu)_2]$	0.65	CH_2Cl_2 (0.05 M)	47
38	A	$Bu_4N[OP(O)(OBu)_2]$	0.65	DMF (0.05 M)	50
39	A	$Bu_4N[OP(O)(OBu)_2]$	0.65	DMSO (0.05 M)	34
40	A	$Bu_4N[OP(O)(OBu)_2]$	0.65	Methanol (0.05 M)	0
41	A	$Bu_4N[OP(O)(OBu)_2]$	0.65	Toluene (0.05 M)	14
42	A	$Bu_4N[OP(O)(OBu)_2]$	0.65	MeCN (0.05 M)	55
43	A	$Bu_4N[OP(O)(OBu)_2]$	0.65	Acetone (0.05 M)	61
44	A	$Bu_4N[OP(O)(OBu)_2]$	0.65	1,2-DCE (0.05 M)	65
45	A	$Bu_4N[OP(O)(OBu)_2]$	0.65	DMA (0.05 M)	28
46	A	$Bu_4N[OP(O)(OBu)_2]$	0.65	NMP (0.05 M)	30
47	A	$Bu_4N[OP(O)(OBu)_2]$	0.65	EtOAc (0.05 M)	46
48	A	$Bu_4N[OP(O)(OBu)_2]$	0.65	$CHCl_3$ (0.05 M)	6
49	A	$Bu_4N[OP(O)(OBu)_2]$	0.65	NO_2Ph (0.05 M)	0
50	A	$Bu_4N[OP(O)(OBu)_2]$	0.65	HFIP (0.05 M)	0
51	A	$Bu_4N[OP(O)(OBu)_2]$	0.65	C_6H_6 (0.05 M)	27
52	A	$Bu_4N[OP(O)(OBu)_2]$	0.65	NO_2Me (0.05 M)	0
53	A	$Bu_4N[OP(O)(OBu)_2]$	0.65	t-BuOH (0.05 M)	73
54	A	$Bu_4N[OP(O)(OBu)_2]$	0.65	t-BuOH/$PhCF_3$ (1:1, 0.05 M)	83
55	A	$Bu_4N[OP(O)(OBu)_2]$	0.65	t-BuOH/$PhCF_3$ (2:1, 0.05 M)	78
56	A	$Bu_4N[OP(O)(OBu)_2]$	0.65	t-BuOH/$PhCF_3$ (1:2, 0.05 M)	82
57	A	$Bu_4N[OP(O)(OBu)_2]$	0.65	$PhCF_3$ (0.05 M) @ 60 °C	45
58	A	$Bu_4N[OP(O)(OBu)_2]$	0.65	1,2-DCE (0.05 M) @ 60 °C	62

Supplementary Table 2. Reaction optimization reactions. [a]Yield determined by ^{19}F NMR spectroscopy using 1,3,5-trifluorobenzene as the internal standard.

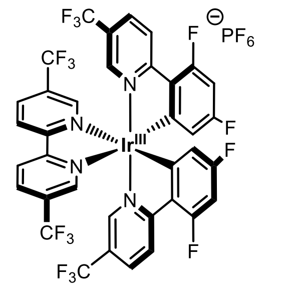

[Ir(dF(CF$_3$)ppy)$_2$(5,5'd(CF$_3$)bpy)]PF$_6$
A

[Ir(dF(CF$_3$)ppy)2(5,5’d(CF$_3$)bpy)]PF$_6$ (**A**) was prepared according to a procedure previously reported in the literature.[6] Spectral data matched values reported in the literature.[7]

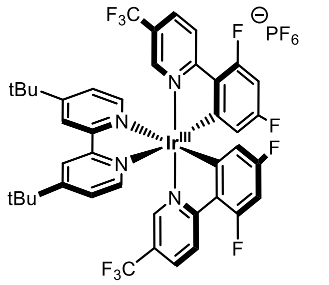

[Ir(dF[CF$_3$]ppy)$_2$(dtbbpy)]PF$_6$
B

[Ir(dF[CF$_3$]ppy)2(dtbbpy)]PF$_6$ (**B**) was prepared as previously reported in the literature.[6] Spectral data matched values reported in the literature.[6]

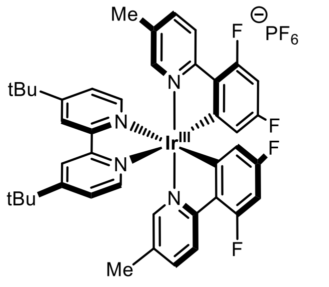

[Ir(dF[Me]ppy)$_2$(dtbbpy)]PF$_6$
C

[Ir(dF[Me]ppy)$_2$(dtbbpy)]PF$_6$ (**C**) was prepared according to a procedure reported in the literature.[6] Spectral data matched values reported in the literature.[8]

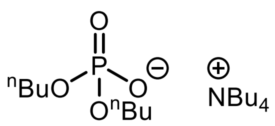

Tetrabutylammonium dibutylphosphate was prepared as previously reported in the literature. Spectral data matched values reported in the literature.[9]

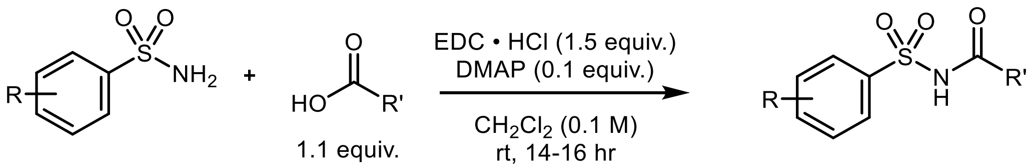

General Procedure A: Sulfonamide coupling with carboxylic acids. In a flame-dried round bottom flask under inert atmosphere, carboxylic acid (1.1 equiv) was dissolved in dry CH_2Cl_2 (0.1 M with respect to aryl sulfonamide). Aryl sulfonamide (1 equiv) and DMAP (0.1 equiv) were sequentially added in one portion each and the mixture was stirred for approximately 1 min at RT. 1-Ethyl-3-(3-dimethylaminopropyl)carbodiimide hydrochloride (1.5 equiv) was added in one portion and the reaction was stirred at room temperature under an argon atmosphere for 14-16 hours. The reaction was concentrated *in vacuo* to provide a viscous oil and the residue was purified with flash chromatography on silica gel (0 to 10% acetone in CH_2Cl_2 gradient). To obtain the products as solids, the concentrated chromatography fractions were triturated with pentane and dried under a vigorous nitrogen stream.

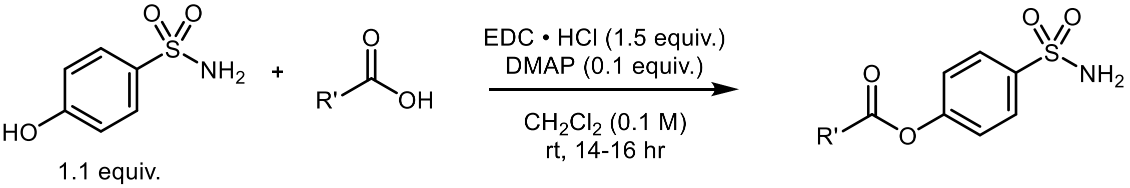

General Procedure B: 4-Hydroxybenzenesulfonamide esterification. In a flame-dried round bottom flask under inert atmosphere, carboxylic acid (1 equiv.) was dissolved in dry CH_2Cl_2 (0.1 M with respect to carboxylic acid). 4-hydroxybenzenesulfonamide (1.1 equiv.) and DMAP (0.1 equiv.) were sequentially added in one portion each and the mixture was stirred for approximately 1 min at RT. 1-Ethyl-3-(3-dimethylaminopropyl)carbodiimide hydrochloride (1.5 equiv) was added in one portion and the reaction was stirred at room temperature under an argon balloon for 14-16 hours. The reaction was concentrated *in vacuo* and the residue was purified with flash chromatography on silica gel (CH_2Cl_2/acetone gradient).

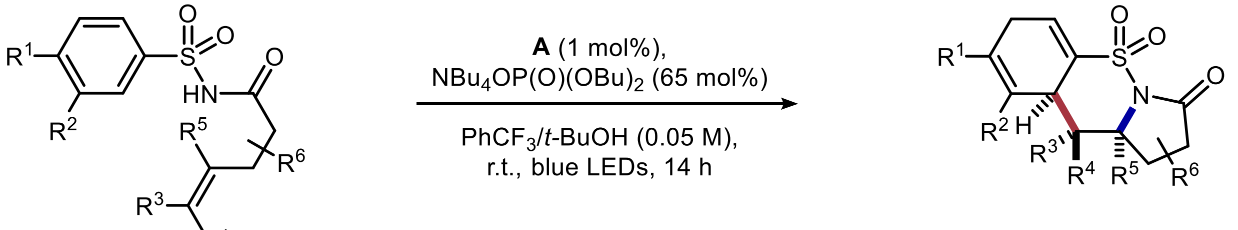

1 **>20:1 d.r.** **2, dearomatized product**

General Procedure C

To an oven dried 2-dram vial was added substrate **1** (0.2 mmol), $NBu_4OP(O)(OBu)_2$ (58 mg, 0.13 mmol, 0.65 equiv), and photocatalyst **A** (2 mg, 1 mol%). The vial contents were then dissolved in a 1:1 mixture of *t*-BuOH:$PhCF_3$ (2 mL each, 0.05 M). The reaction solution was degassed by

sparging with argon for 15 min. Then the vial was quickly capped and sealed with parafilm. The reaction was irradiated with two, H150 blue Kessil lamps positioned ~5 cm away and cooled with an overhead fan. After 14 h, the reaction was directly concentrated *in vacuo.* The resultant residue was subjected to flash column chromatography over silica providing the pure diene **2**.

Characterization of substrates

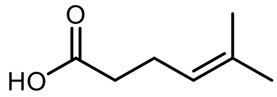

5-methylhex-4-enoic acid: Prepared according to a previous literature report.[10] Spectral data matched values reported in the literature.

^{1}H NMR (500 MHz, $CDCl_3$) = δ 11.46 (br s, 1H), 5.13 – 5.07 (m, 1H), 2.40 – 2.35 (m, 2H), 2.35-2.28 (m, 2H), 1.69 (s, 3H), 1.62 (s, 3H) ppm

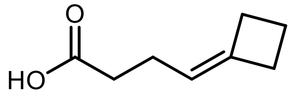

4-cyclobutylidenebutanoic acid: Prepared *via* a Wittig olefination from modified literature procedure.[11] To a suspension of (3-carboxypropyl)triphenylphosphonium bromide (4.29 g, 10 mmol, 2 equiv.) in THF (20 mL, anhydrous) in a 100 mL RBF with 2 stir bars (for better agitation of resulting suspension) was added sodium bis(trimethylsilyl)amide (20 mL, 20 mmol, 4 equiv., 1 M in THF) dropwise at room temperature. The bright orange mixture was stirred at this temperature for 30 minutes. A solution of cyclobutanone (5 mmol, 0.37 mL, 1 equiv.) in THF (5 mL, anhydrous) was added dropwise. The reaction was refluxed (80 °C) for 7.5 hours. After cooling to room temperature, the reaction contents were transferred to a separatory funnel with 100 mL Et_2O and 100 mL 1 M NaOH solution. The aqueous layer was washed with Et_2O (2 x 50 mL), acidified with conc. HCl to pH = 1, and washed with Et_2O (3 x 50 mL). The organic washes of the acidic aqueous phase were dried with Na_2SO_4, filtered, and concentrated. The resulting crude was purified by silica gel chromatography (5-35% EtOAc in hexanes) to deliver the title product as a clear colorless oil. 201 mg, 29% yield. Spectral data matched values reported in the literature.

^{1}H NMR (400 MHz, $CDCl_3$) = δ 11.43 (br s, 1H), 5.04 (tp, J = 7.2, 2.6 Hz, 1H), 2.69 – 2.58 (m, 4H), 2.37 (t, J = 7.4 Hz, 2H), 2.20 (q, J = 7.2 Hz, 2H), 1.93 (p, J = 7.9 Hz, 2H) ppm

^{13}C NMR (100 MHz, $CDCl_3$) = δ 179.8, 142.2, 117.9, 34.3, 31.0, 29.3, 23.4, 17.1 ppm

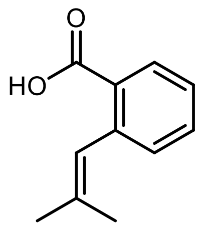

2-(2-methylprop-1-en-1-yl)benzoic acid: Prepared *via* a Wittig olefination according to a previous literature report.[10] Spectral data matched values reported in the literature.

^{1}H NMR: (500 MHz, $CDCl_3$) = δ 10.74 (br s, 1H), 8.05 (d, *J* = 7.8 Hz, 1H), 7.50 (t, *J* = 7.5 Hz, 1H), 7.33 – 7.26 (m, 2H), 6.70 (s, 1H), 1.95 (s, 3H), 1.73 (s, 3H) ppm

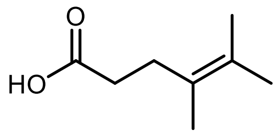

4,5-dimethylhex-4-enoic acid: Prepared according to a previous literature report.[11] Spectral data matched values reported in the literature.

^{1}H NMR (400 MHz, $CDCl_3$) = δ 11.04 (br s, 1H), 2.38 (app s, 4H), 1.67 (s, 3H), 1.64 (app s, 6H) ppm

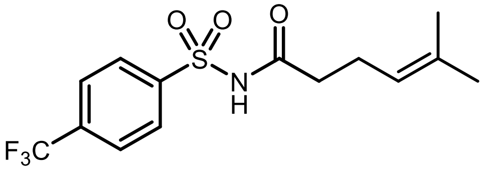

5-methyl-N-((4-(trifluoromethyl)phenyl)sulfonyl)hex-4-enamide (1a): Prepared according to **General Procedure A**. 414 mg, 82%. Colorless solid.

^{1}H NMR (500 MHz, $CDCl_3$) = δ 8.53 (br s, 1H), 8.21 (d, *J* = 8.3 Hz, 2H), 7.82 (d, *J* = 8.4 Hz, 2H), 5.02-4.95 (m, 1H), 2.33 – 2.22 (m, 4H), 1.64 (s, 3H), 1.54 (s, 3H) ppm

^{13}C NMR (176 MHz, $CDCl_3$) = δ 171.1, 142.1, 136.0, 135.7 (q, *J* = 33.2 Hz), 135.4, 134.7, 129.1, 126.3 (q, *J* = 3.6 Hz), 125.5, 123.2 (q, *J* = 273 Hz), 121.4, 120.9, 36.6, 25.7, 23.2, 17.7 ppm

^{19}F NMR (377 MHz, $CDCl_3$) = δ -63.33 ppm

IR (*neat*): 3125, 2901, 1698, 1459, 1404, 1354, 1221, 1133, 1090, 1061, 1038 cm^{-1}

HRMS (ESI+) *m/z* calculated for $C_{14}H_{16}F_3NO_3S$ $[M+H]^+$: 335.0803, found 335.0813.

R_f = 0.5 (1:1, Hex:EtAOc), one streaky yellow spot, $KMnO_4$, UV

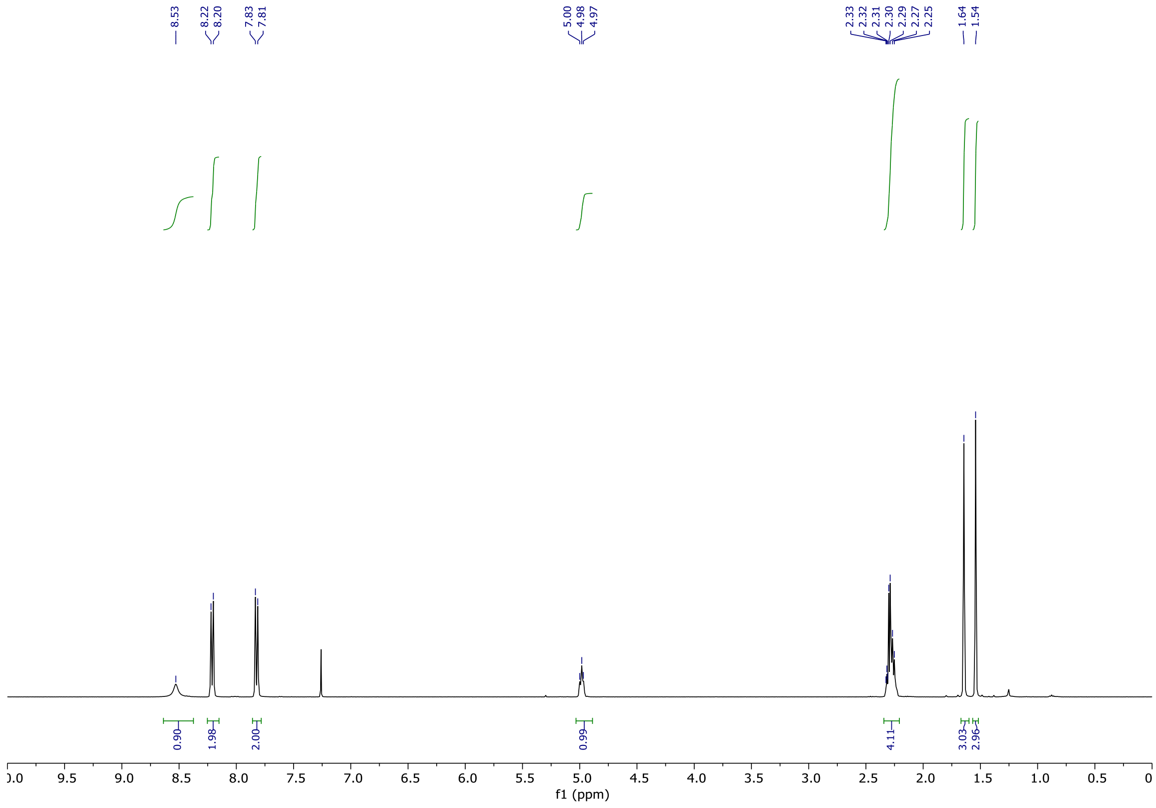

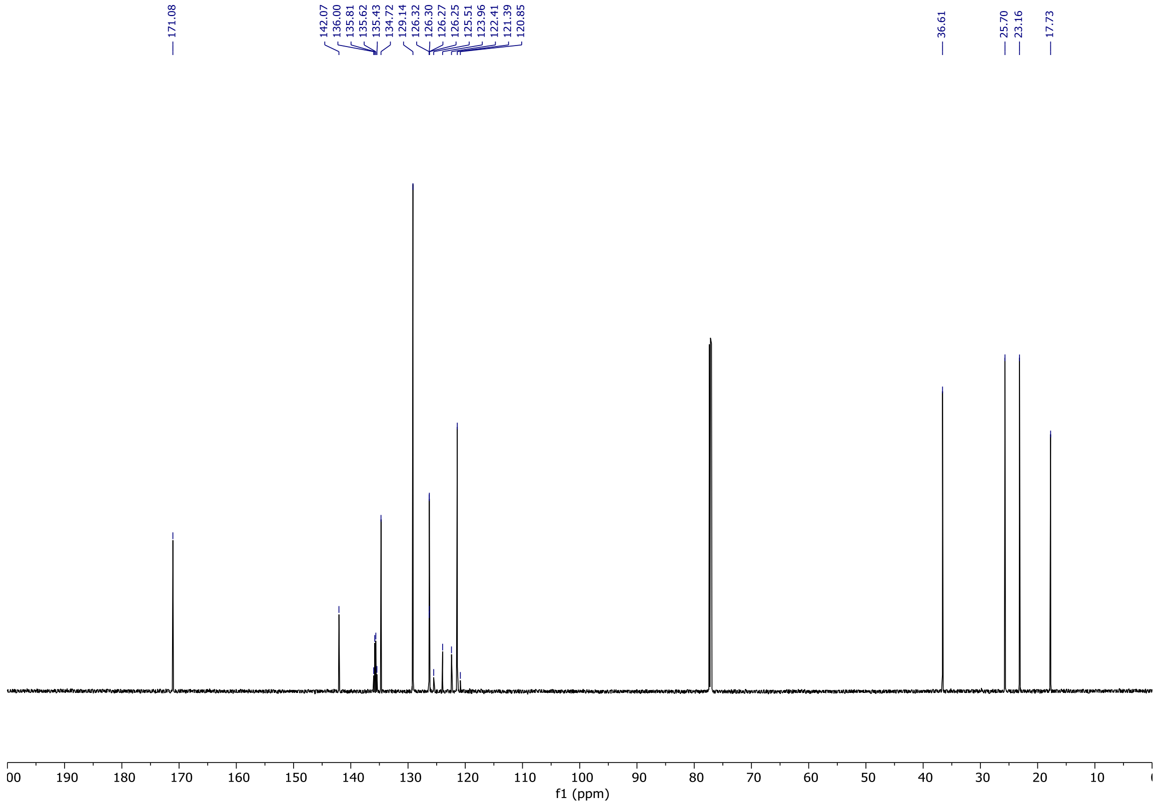

Supplementary Figure 2: ^{1}H (top) and ^{13}C NMR (bottom) for compound **1a** in $CDCl_3$

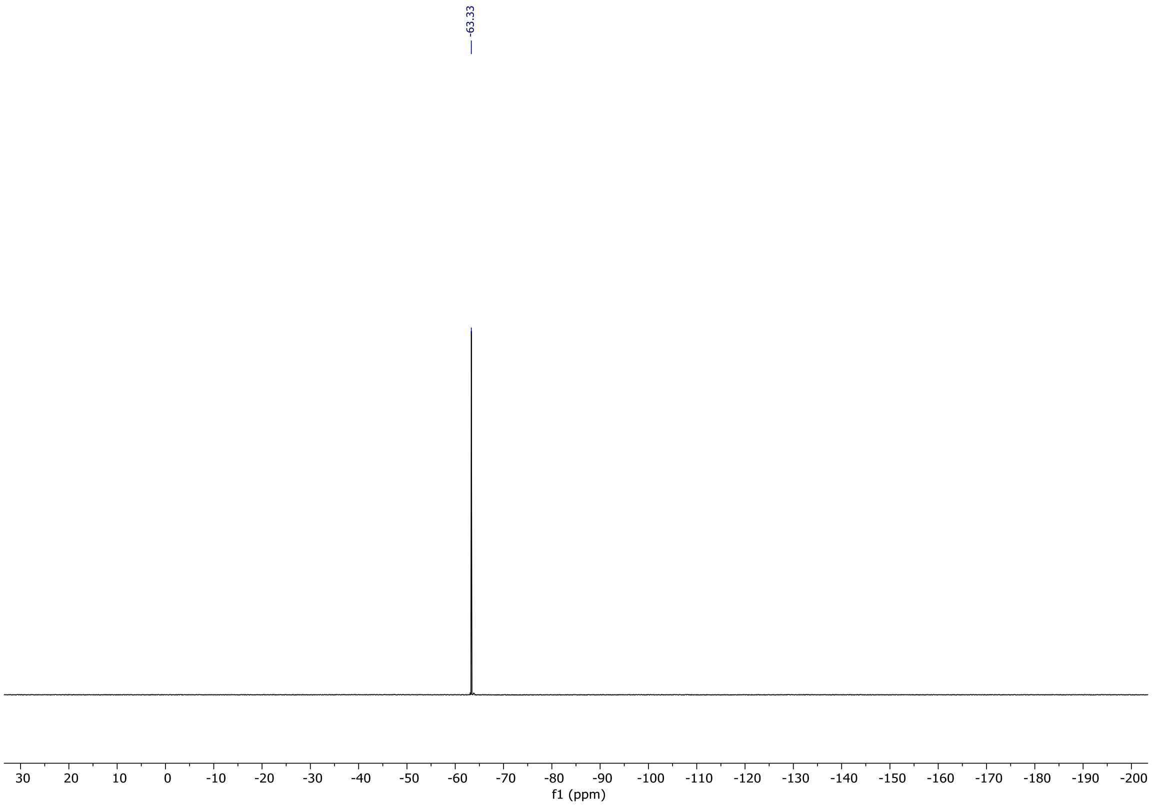

Supplementary Figure 3: ^{19}F NMR for compound **1a** in $CDCl_3$

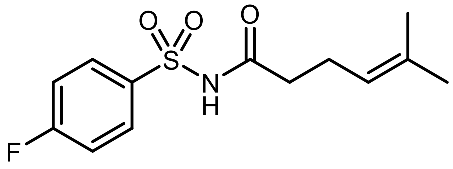

N-((4-fluorophenyl)sulfonyl)-5-methylhex-4-enamide (1b): Prepared according to **General Procedure A**. 403 mg, 71%. Colorless solid.

^{1}H NMR (700 MHz, $CDCl_3$) = δ 8.76 – 8.62 (m, 1H), 8.12 – 8.07 (m, 2H), 7.24 – 7.19 (m, 2H), 4.98 (t, *J* = 6.9 Hz, 1H), 2.31 – 2.22 (m, 4H), 1.64 (s, 3H), 1.54 (s, 3H ppm

^{13}C NMR (176 MHz, $CDCl_3$) δ 170.7, 166.1 (d, *J* = 257 Hz), 134.7, 134.6 (d, *J* = 3.1 Hz), 131.5 (d, *J* = 9.8 Hz), 121.5, 116.4 (d, *J* = 22.8 Hz), 35.7, 25.8, 23.2, 17.8 ppm

^{19}F NMR (377 MHz, $CDCl_3$) δ -102.64 – -102.74 (m) ppm

IR (neat) 3190, 2914, 1696, 1594, 1495, 1455, 1356, 1223, 1180, 1135 cm^{-1}

HRMS (EI+) *m/z* calculated for $C_{13}H_{16}FNO_3S$ $[M]^+$: 285.0835, found 285.0834.

R_f = 0.4 (1:1, Hex:EtAOc), one streaky yellow spot, $KMnO_4$, UV

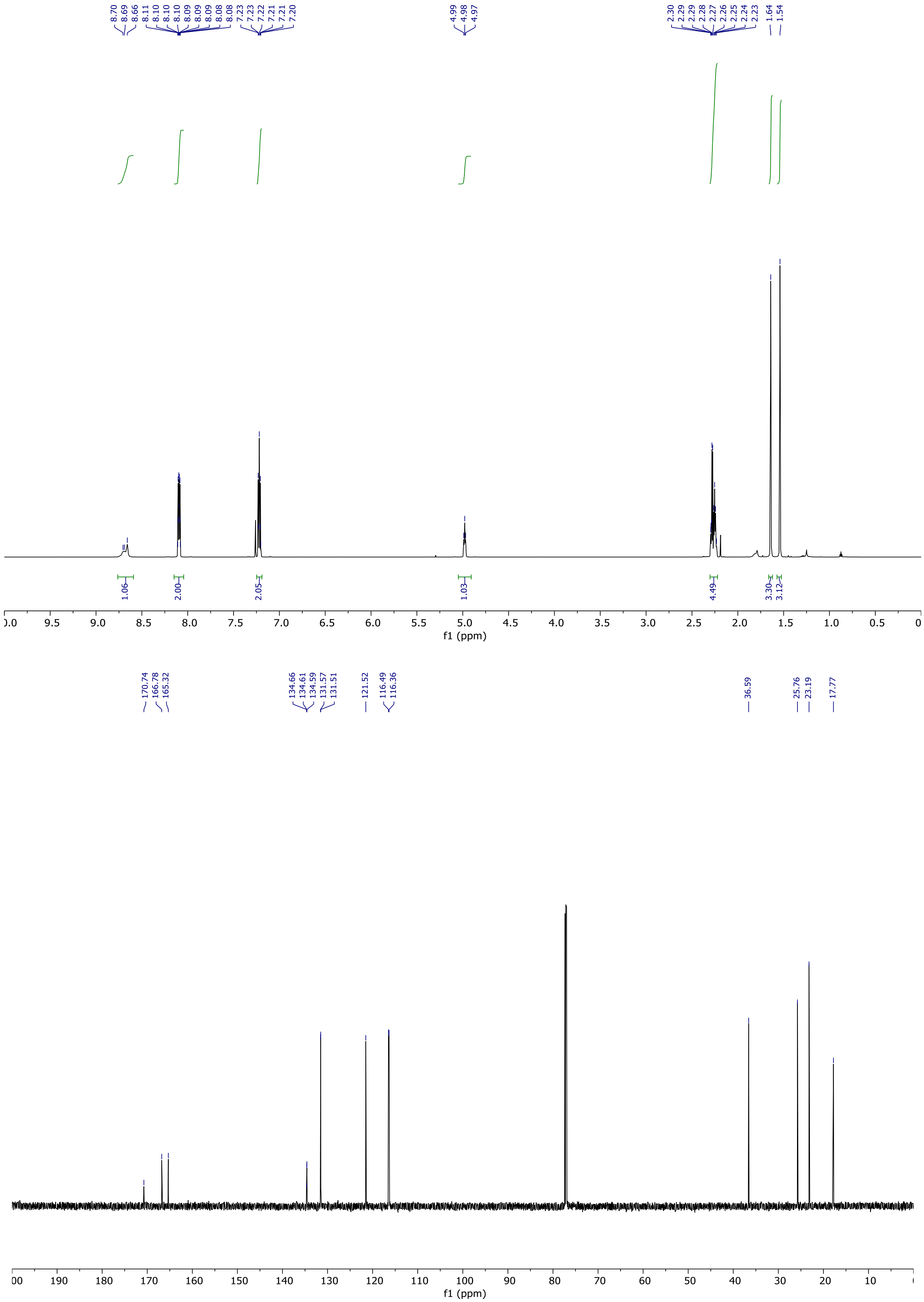

Supplementary Figure 4: ^{1}H (top) and ^{13}C NMR (bottom) for compound **1b** in $CDCl_3$

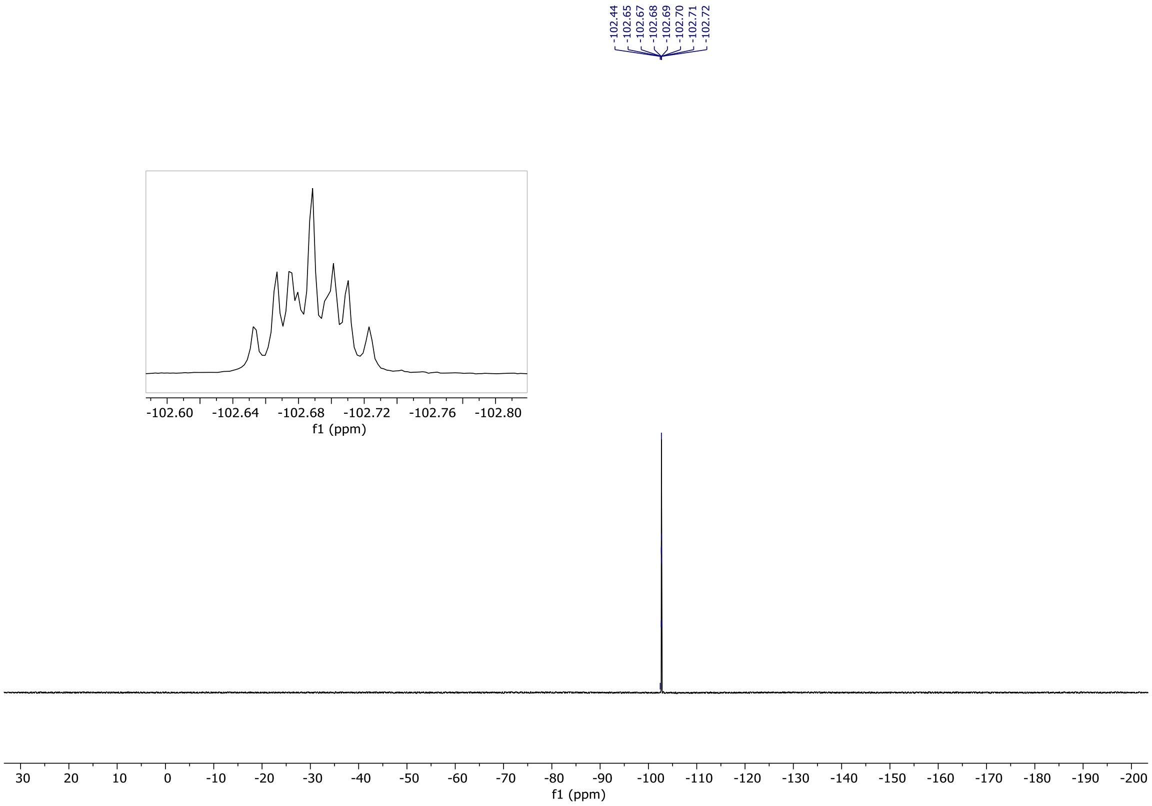

Supplementary Figure 5: ^{19}F NMR (377 MHz) for compound **1b** in $CDCl_3$

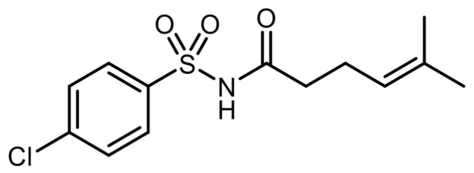

N-((4-chlorophenyl)sulfonyl)-5-methylhex-4-enamide (1c): Prepared according to **General Procedure A**. 401 mg, 63%. Colorless solid.

^{1}H NMR (700 MHz, $CDCl_3$) = δ 8.85 (s, 1H), 8.00 (d, *J* = 8.4 Hz, 2H), 7.52 (d, *J* = 8.4 Hz, 2H), 4.97 (t, *J* = 6.4 Hz, 1H), 2.33 – 2.21 (m, 4H), 1.63 (s, 3H), 1.53 (s, 3H) ppm

^{13}C NMR (176 MHz, $CDCl_3$) = δ 171.0, 140.9, 137.0, 134.6, 130.0, 129.4, 121.5, 36.6, 25.7, 23.2, 17.8 ppm

IR (neat) 3244, 2971, 1727, 1577, 1433, 1411, 1331, 1282, 1181, 1081 cm^{-1}

HRMS (ESI+) *m/z* calculated for $C_{13}H_{16}ClNO_3S$ $[M+H]^+$: 302.0612, found 302.0617.

R_f: (1:19 – Acetone:DCM) = 0.49

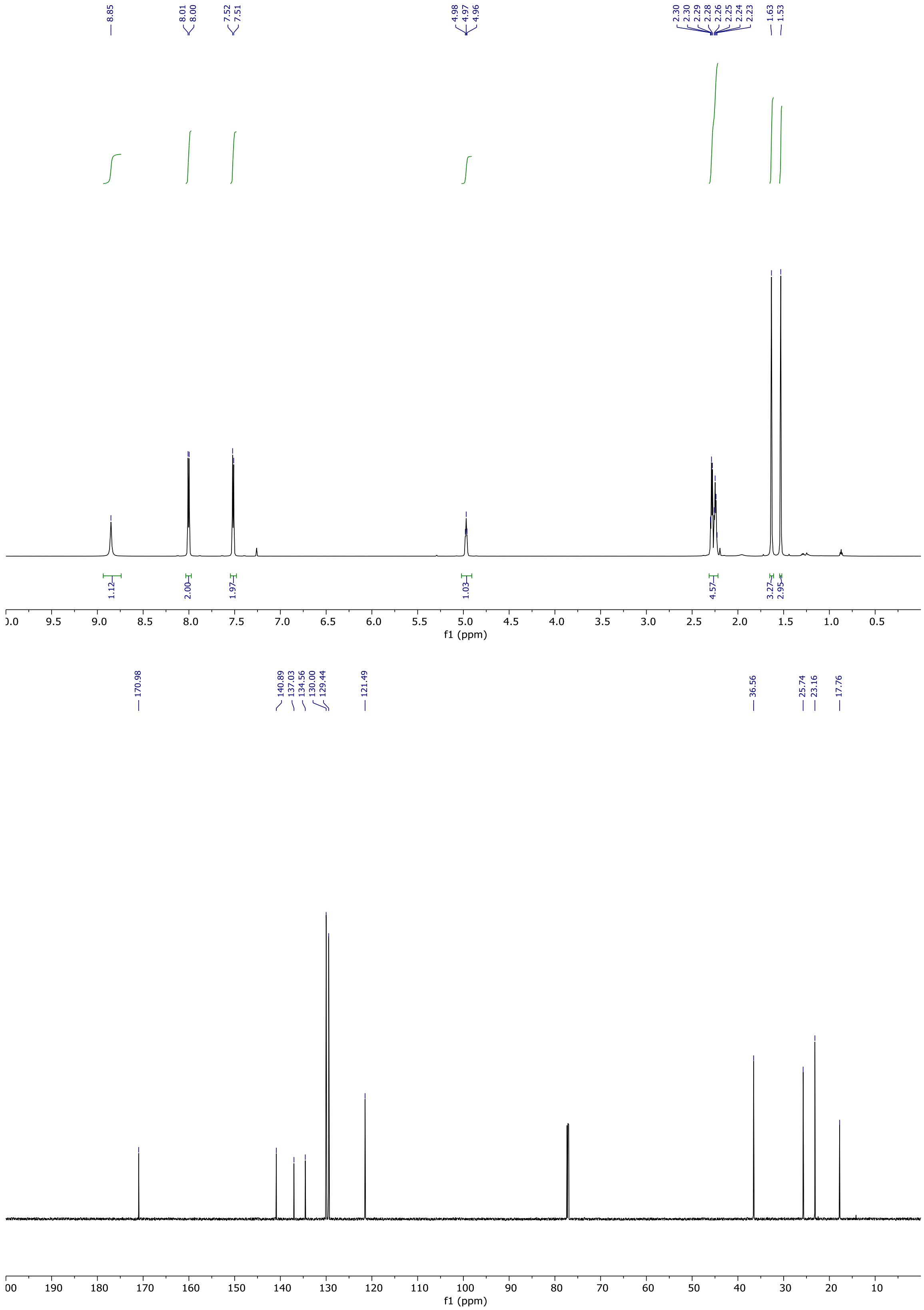

Supplementary Figure 6: ^{1}H (top) and ^{13}C NMR (bottom) for compound **1c** in $CDCl_3$

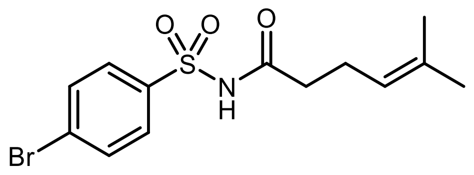

N-((4-bromophenyl)sulfonyl)-5-methylhex-4-enamide (1d): Prepared according to **General Procedure A**. 664 mg, 48%. Colorless solid.

^{1}H NMR (700 MHz, $CDCl_3$) = δ 8.81 (s, 1H), 7.92 (d, *J* = 8.6 Hz, 2H), 7.68 (d, *J* = 8.6 Hz, 2H), 5.00 – 4.95 (m, 1H), 2.31 – 2.22 (m, 4H), 1.64 (s, 3H), 1.53 (s, 3H) ppm

^{13}C NMR (176 MHz, $CDCl_3$) = δ 170.9, 137.6, 134.6, 132.4, 130.0, 129.5, 121.5, 36.6, 25.8, 23.2, 17.8 ppm

IR (neat) 3246, 2970, 1728, 1573, 1433, 1411, 1388, 1331, 1123, 1084 cm^{-1}

HRMS (ESI+) *m/z* calculated for $C_{13}H_{16}BrNO_3S$ $[M+H]^+$: 346.0107, found 346.0117.

R_f = 0.5 (1:1, Hex:EtAOc), one streaky yellow spot, $KMnO_4$, UV

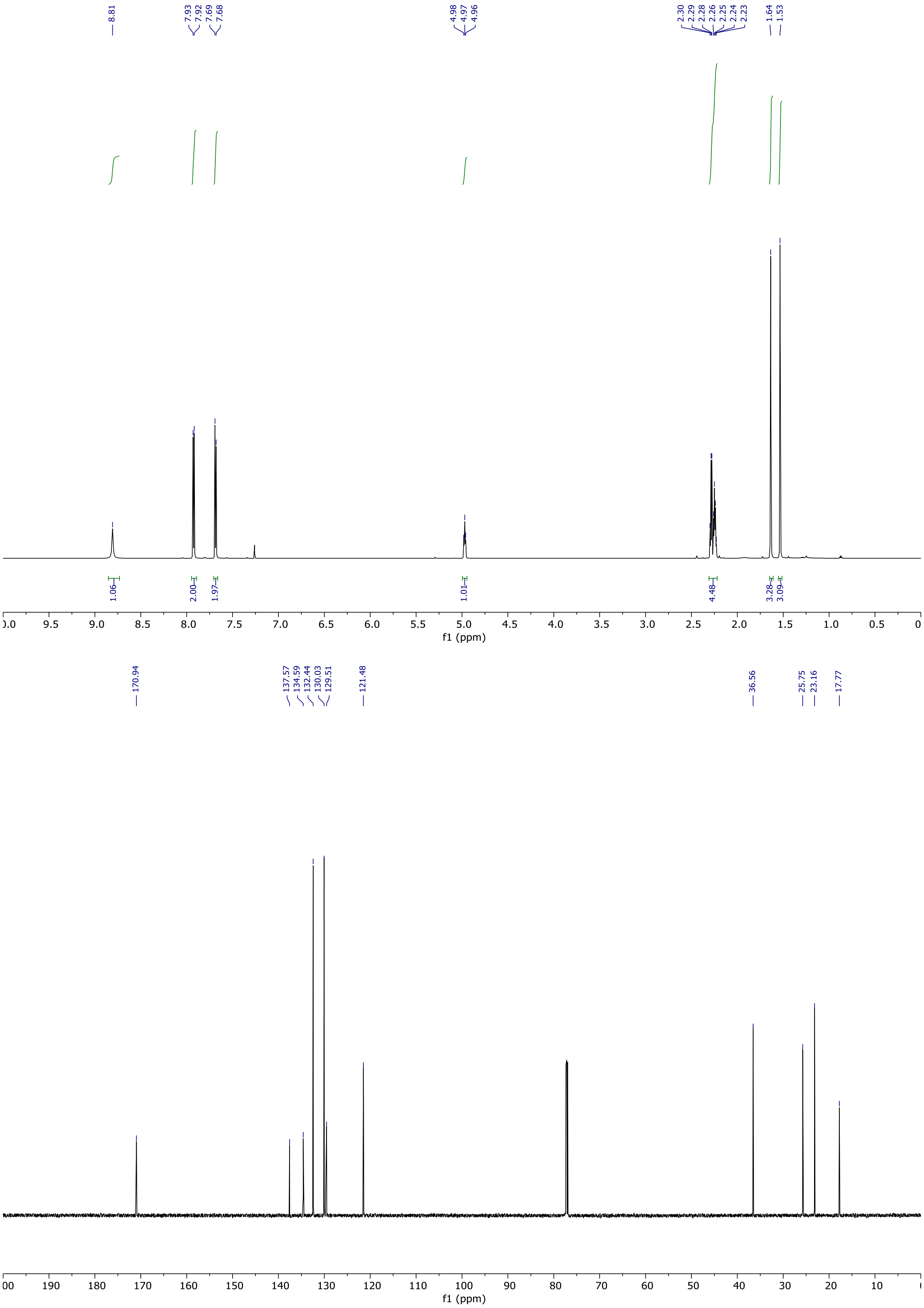

Supplementary Figure 7: ^{1}H (top) and ^{13}C NMR (bottom) for compound **1d** in $CDCl_3$

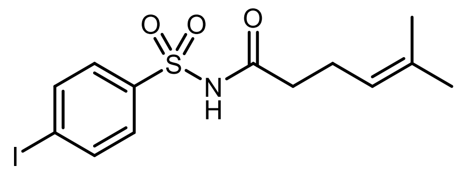

N-((4-iodophenyl)sulfonyl)-5-methylhex-4-enamide (1e): Prepared according to **General Procedure A**. 301 mg, 77%. Colorless solid.

^{1}H NMR (400 MHz, $CDCl_3$) = δ 8.11 (br s, 1H), 7.91 (d, *J* = 8.7 Hz, 2H), 7.76 (d, *J* = 8.6 Hz, 2H), 5.04 – 4.95 (m, 1H), 2.29 – 2.23 (m, 4H), 1.67 (s, 3H), 1.56 (s, 3H) ppm

^{13}C NMR (176 MHz, $CDCl_3$) = δ 170.3, 138.4, 138.3, 135.1, 129.9, 121.5, 102.2, 36.7, 25.8, 23.22, 17.8 ppm

IR (neat) 3248, 2908, 1728, 1567, 1434, 1410, 1384, 1330, 1170, 1084 cm^{-1}

HRMS (ESI+) *m/z* calculated for $C_{13}H_{16}INO_3S$ $[M+H]^+$: 393.9968, found 393.9978.

R_f: (1:19 – Acetone:DCM) = 0.63.

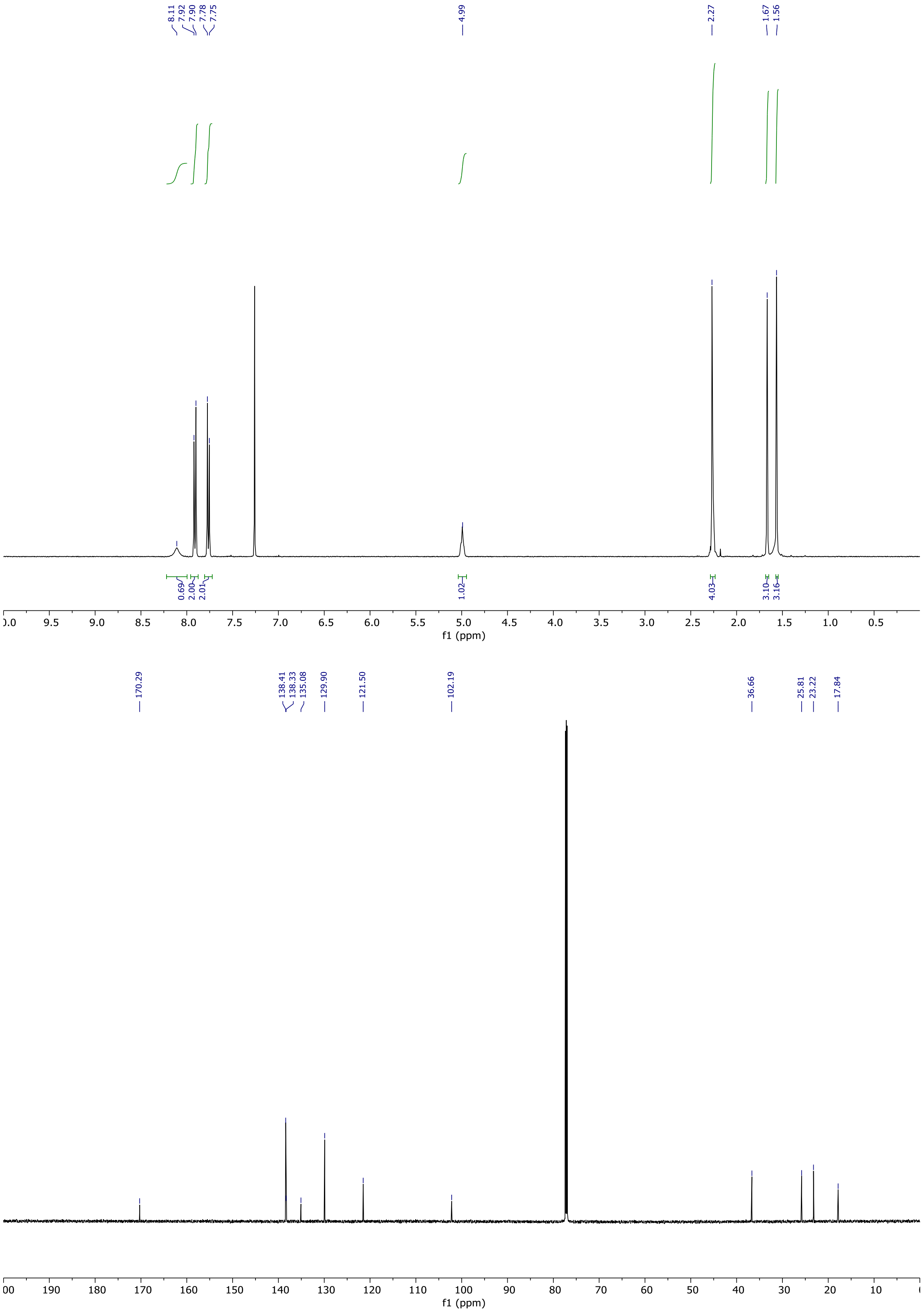

Supplementary Figure 8: ^{1}H (top) and ^{13}C NMR (bottom) for compound **1e** in $CDCl_3$

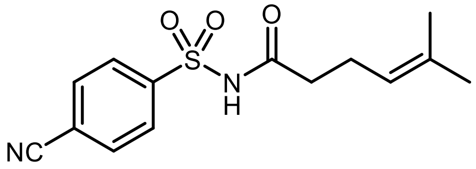

N-((4-cyanophenyl)sulfonyl)-5-methylhex-4-enamide (1f): Prepared according to **General Procedure A**. 213 mg, 33%. Colorless solid.

^{1}H NMR (400 MHz, $CDCl_3$): = δ 8.75 (br s, 1H), 8.19 (d, *J* = 8.3 Hz, 2H), 7.85 (d, *J* = 8.3 Hz, 2H), 5.01 – 4.93 (m, 1H), 2.33 – 2.20 (m, 4H), 1.64 (s, 3H), 1.54 (s, 3H) ppm

^{13}C NMR (176 MHz, $CDCl_3$) = δ 170.9, 142.7, 134.4, 132.9, 129.3, 121.4, 117.7, 117.7, 117.2, 36.6, 25.8, 23.1, 17.8 ppm

IR (neat) 3203, 2916, 2235, 1699, 1444, 1402, 1355, 1288, 1187, 1170, 1084 cm^{-1}

HRMS (ESI+) *m/z* calculated for $C_{14}H_{16}N_2O_3S$ $[M+H]^+$: 293.0954, found 293.0968.

R_f: (1:19 – Acetone:DCM) = 0.52.

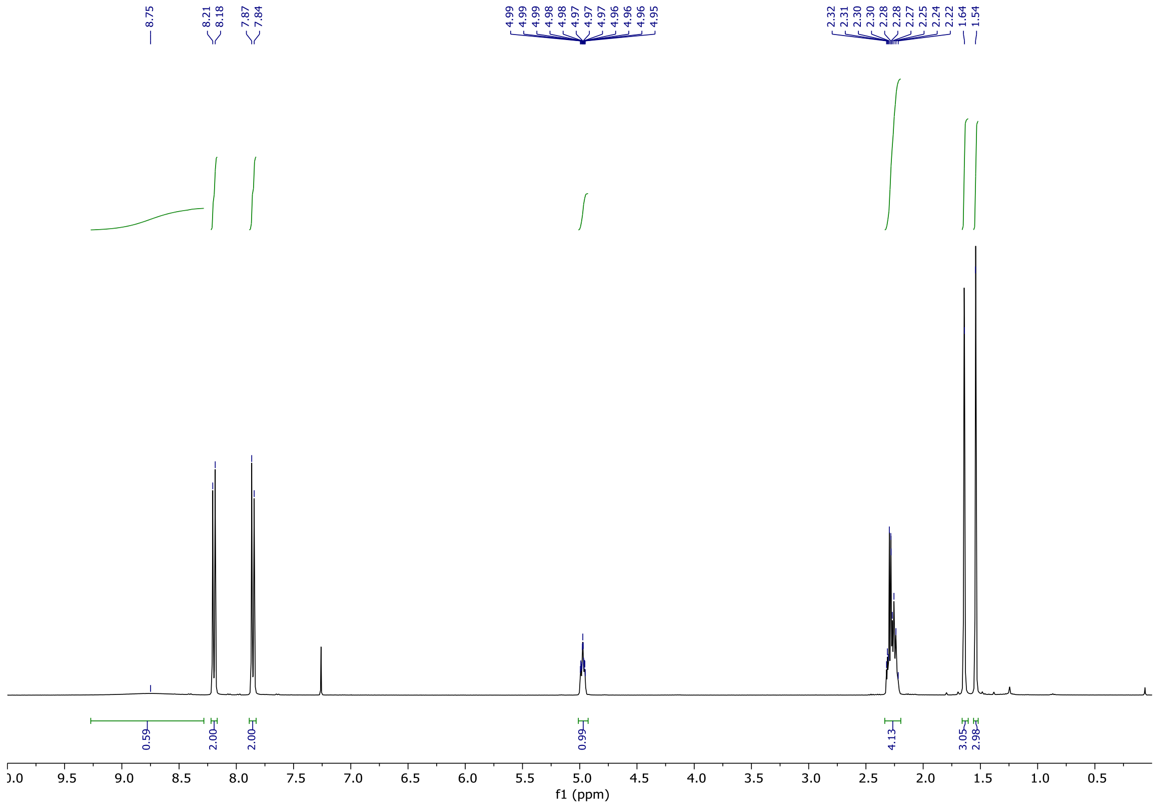

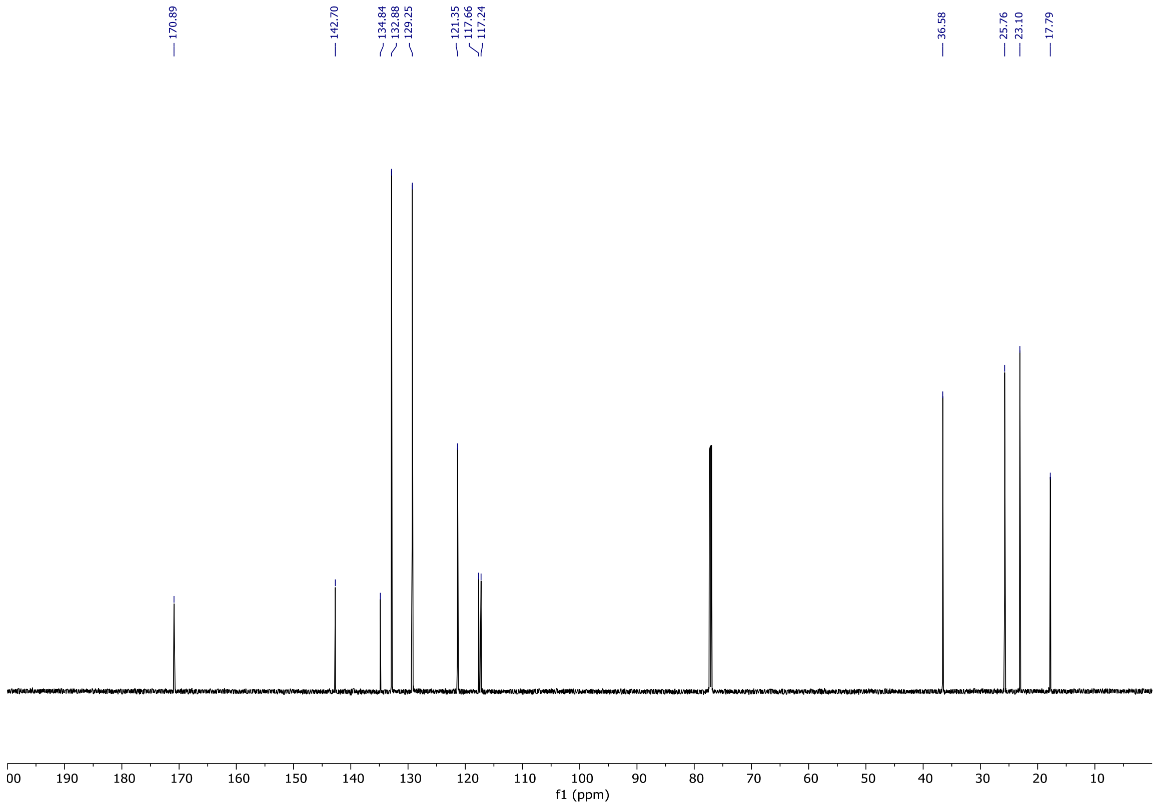

Supplementary Figure 9: ^{1}H (top) and ^{13}C NMR (bottom) for compound **1f** in $CDCl_3$

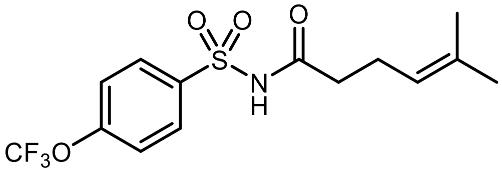

5-methyl-N-((4-(trifluoromethoxy)phenyl)sulfonyl)hex-4-enamide (1g): Prepared according to **General Procedure A**. 244 mg, 58%. Colorless solid.

^{1}H NMR (400 MHz, $CDCl_3$) = δ 8.21 (br s, 1H), 8.13 (d, *J* = 8.9 Hz, 2H), 7.37 (d, *J* = 8.8 Hz, 2H), 5.03 – 4.96 (m, 1H), 2.32 – 2.22 (m, 4H), 1.66 (s, 3H), 1.55 (s, 3H) ppm

^{13}C NMR (175 MHz, $CDCl_3$) = δ 170.6, 153.3, 136.8, 134.9, 131.0, 121.5, 120.8, 120.3 (q, *J* = 260 Hz) 36.7, 25.8, 23.2, 17.9 ppm

^{19}F NMR (377 MHz, $CDCl_3$) = δ -57.68 ppm

IR (neat): 3142, 2902, 1698, 1592, 1457, 1408, 1381, 1354, 1300, 1241 cm^{-1}

HRMS (ESI+) *m/z* calculated for $C_{14}H_{16}F_3NO_4S$ $[M+H]^+$: 352.0825, found 352.0834.

R_f: (1:9 – Acetone:DCM) = 0.81.

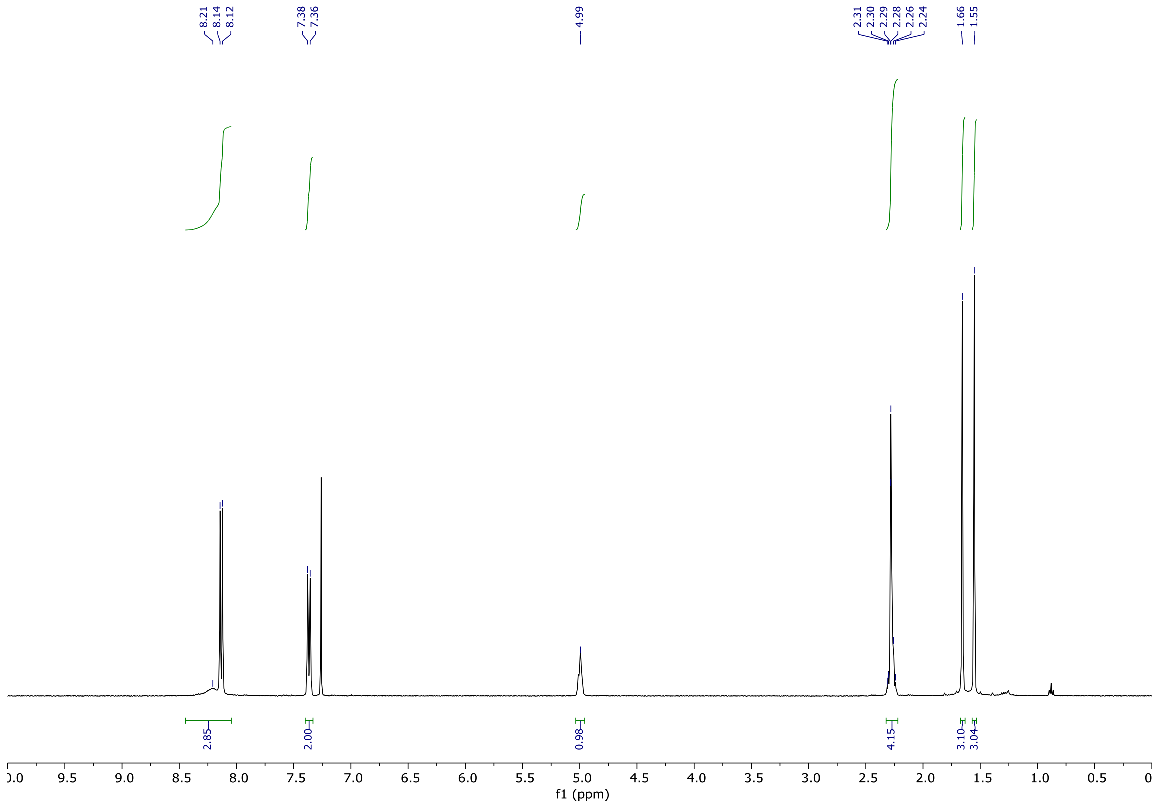

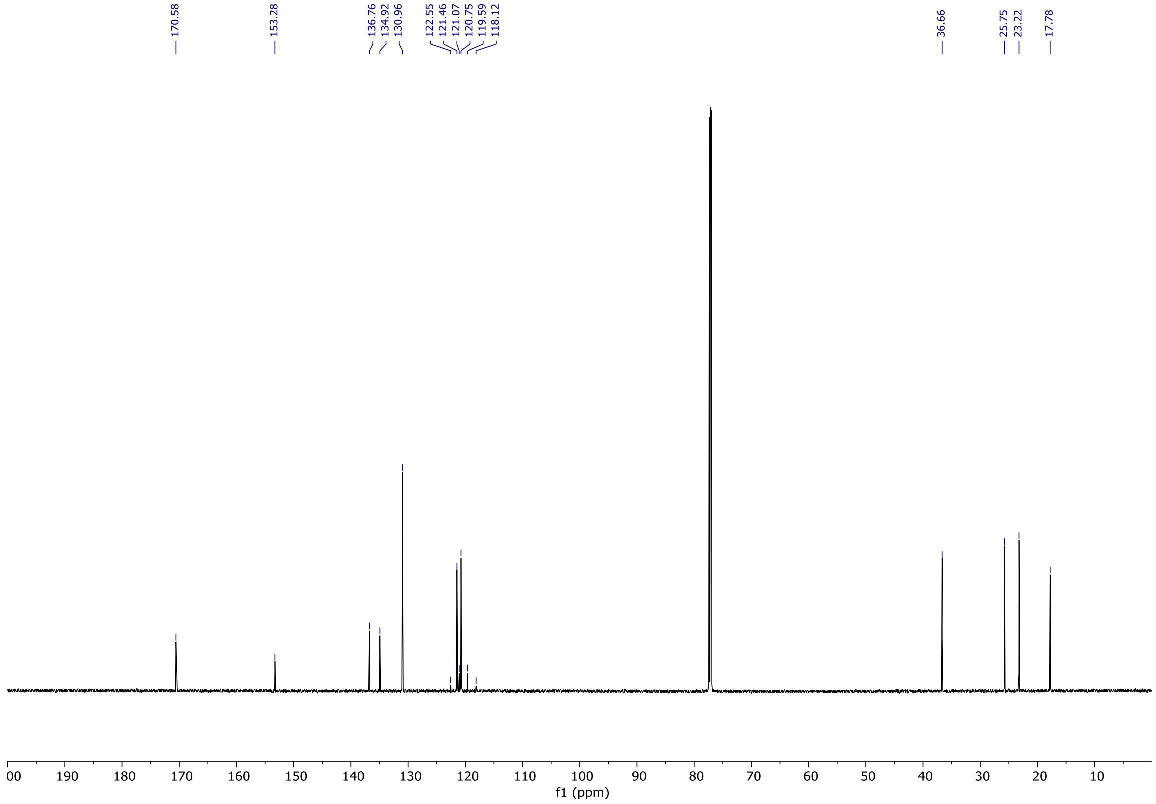

Supplementary Figure 10: ^{1}H (top) and ^{13}C NMR (bottom) for compound **1g** in $CDCl_3$

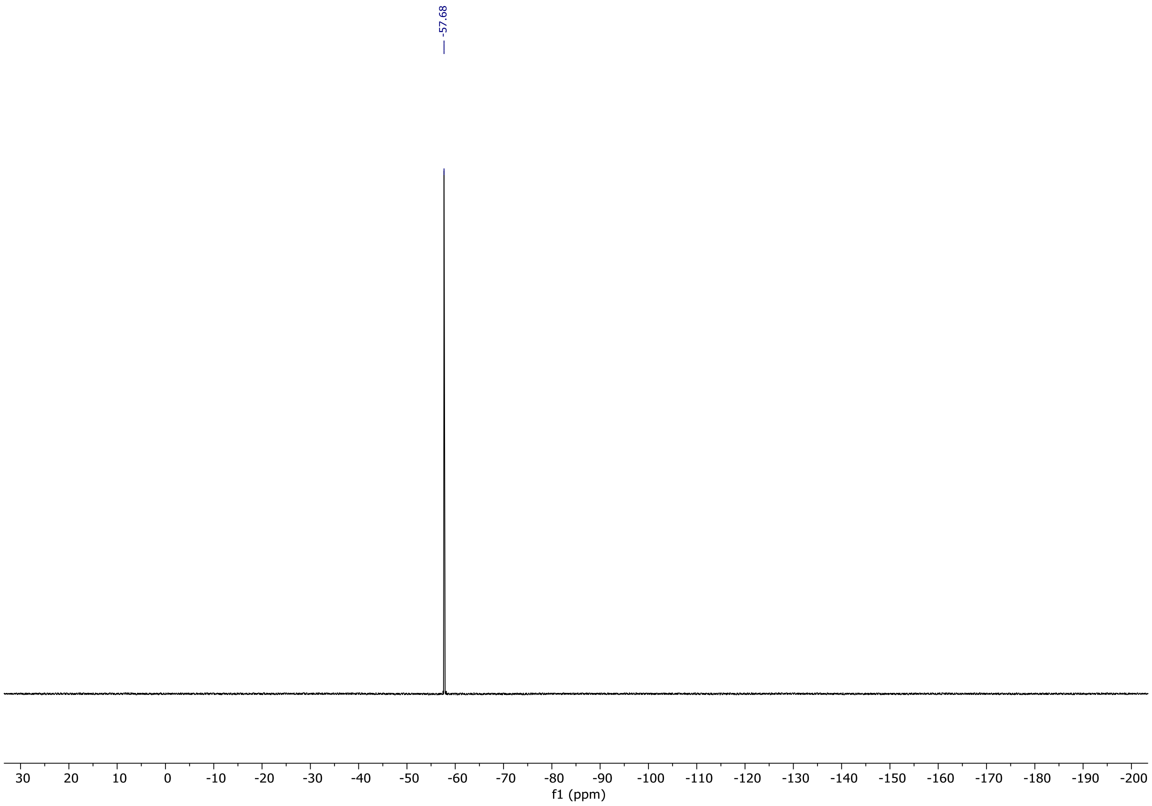

Supplementary Figure 11: ^{19}F NMR for compound **1g** in $CDCl_3$

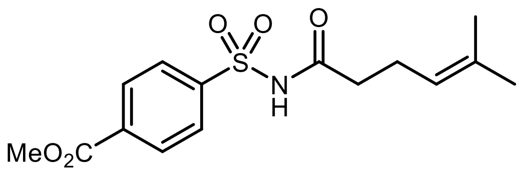

Methyl 4-(N-(5-methylhex-4-enoyl)sulfamoyl)benzoate (1h): Prepared according to **General Procedure A**. 456 mg, 70%. Colorless solid.

^{1}H NMR (500 MHz, $CDCl_3$) = δ 9.02 (br s, 1H), 8.19 (d, *J* = 8.6 Hz, 2H), 8.12 (d, *J* = 8.6 Hz, 2H), 4.99 – 4.93 (m, 1H), 3.96 (s, 3H), 2.33 – 2.27 (m, 2H), 2.27 – 2.20 (m, 2H), 1.61 (s, 3H), 1.51 (s, 3H) ppm

^{13}C NMR (176 MHz, $CDCl_3$) = δ 171.1, 165.7, 142.5, 135.0, 134.5, 130.2, 128.5, 121.5, 52.9, 36.6, 25.7, 23.1, 17.7 ppm

IR (neat) 3236, 2962, 1719, 1435, 1400, 1349, 1279, 1175, 1116, 1084 cm^{-1}

HRMS (ESI+) *m/z* calculated for $C_{15}H_{19}NO_5S$ $[M+H]^+$: 326.1057, found 326.1060.

R_f: (2:3 – EtOAc:Hex) = 0.48.

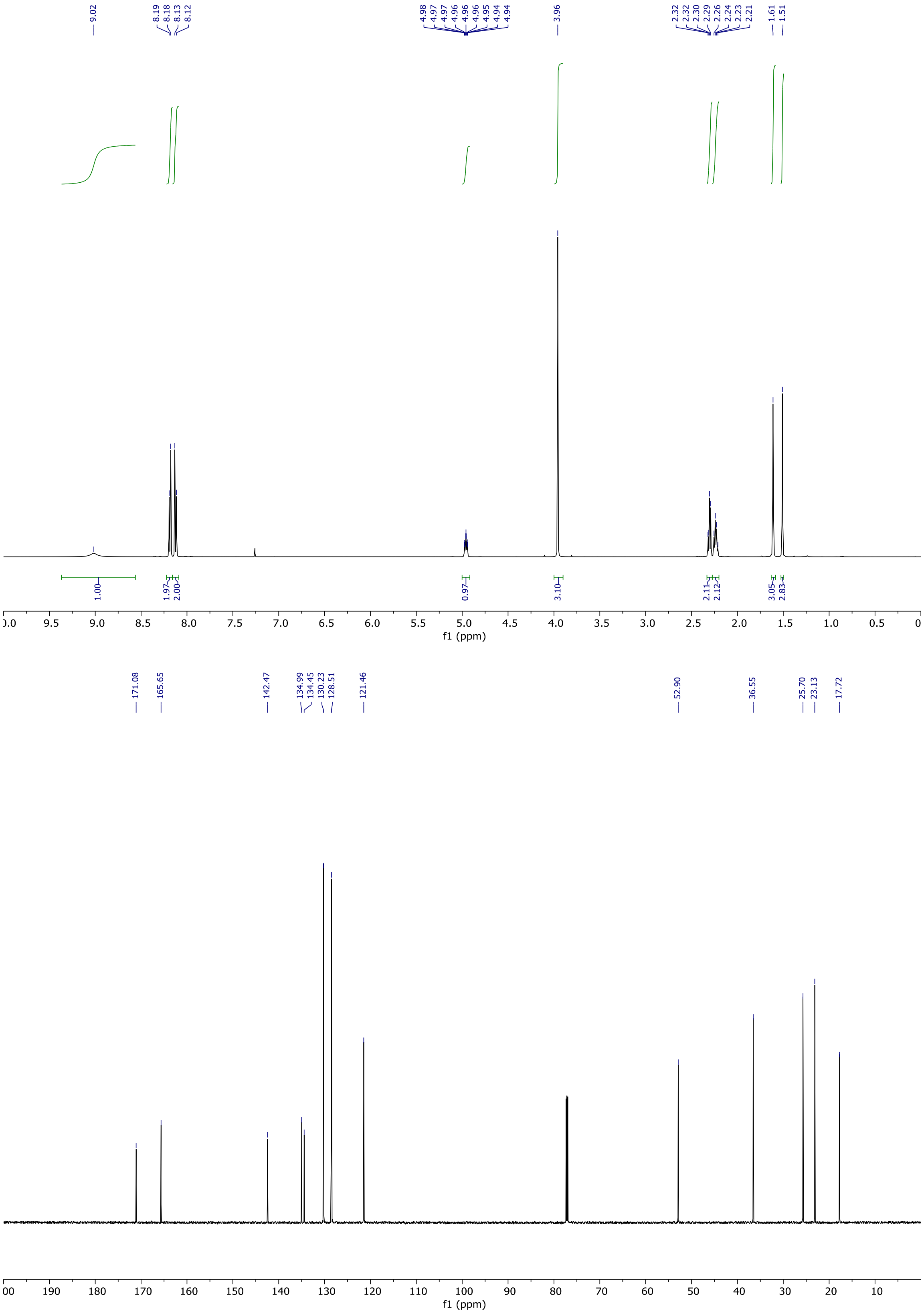

Supplementary Figure 12: ^{1}H (top) and ^{13}C NMR (bottom) for compound **1h** in $CDCl_3$

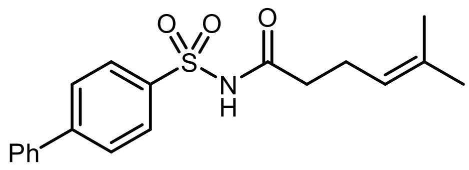

N-([1,1'-biphenyl]-4-ylsulfonyl)-5-methylhex-4-enamide (1i): Prepared according to **General Procedure A**. 531 mg, 70%. Colorless foam.

^{1}H NMR (500 MHz, $CDCl_3$) = δ 9.16 (br s, 1H), 8.15 (d, *J* = 8.5 Hz, 2H), 7.75 (d, *J* = 8.5 Hz, 2H), 7.63 – 7.56 (m, 2H), 7.48 (t, *J* = 7.4 Hz, 2H), 7.46 – 7.39 (m, 1H), 5.06 – 4.96 (m, 1H), 2.39 – 2.32 (m, 2H), 2.32 – 2.24 (m, 2H), 1.63 (s, 3H), 1.53 (s, 3H) ppm

^{13}C NMR (176 MHz, $CDCl_3$) = δ 171.2, 147.0, 139.2, 137.1, 134.2, 129.2, 129.0, 128.8, 127.7, 127.5, 121.6, 36.6, 25.7, 23.2, 17.7 ppm

IR (neat) 3234, 2912, 1693, 1594, 1481, 1433, 1338, 1165, 1124, 1086 cm^{-1}

HRMS (ESI+) *m/z* calculated for $C_{19}H_{21}NO_3S$ $[M+H]^+$: 344.1315, found 344.1318

R_f: (1:9 – Acetone:DCM) = 0.89.

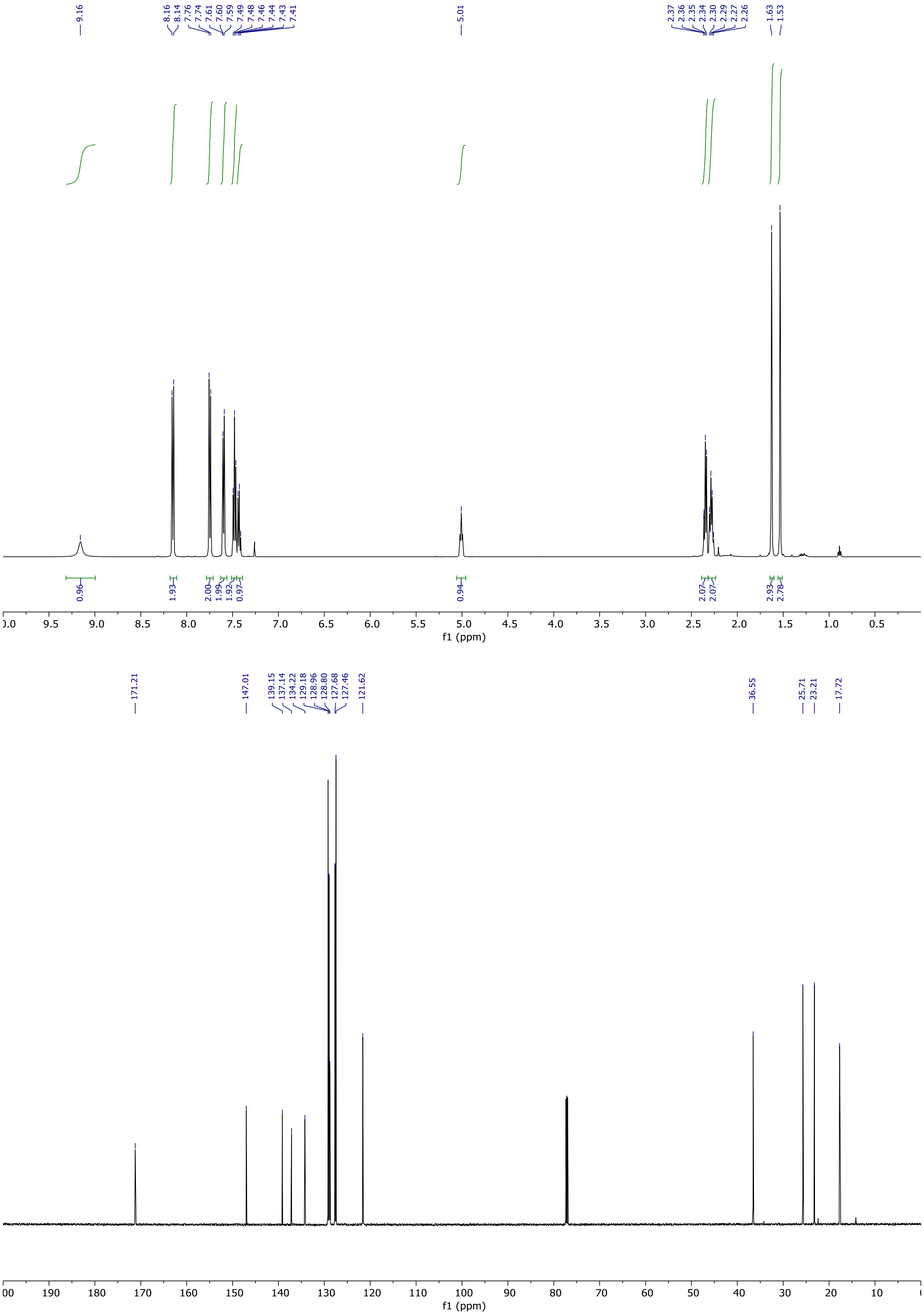

Supplementary Figure 13: ^{1}H (top) and ^{13}C NMR (bottom) for compound **1i** in $CDCl_3$

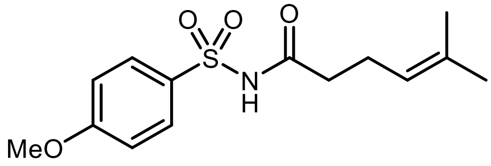

N-((4-methoxyphenyl)sulfonyl)-5-methylhex-4-enamide (1j): Prepared according to **General Procedure A**. 743 mg, 64%. Colorless solid.

^{1}H NMR (700 MHz, $CDCl_3$) = δ 8.95 (br s, 1H), 7.99 (d, *J* = 9.0 Hz, 2H), 6.99 (d, *J* = 9.0 Hz, 2H), 4.99 – 4.95 (m, 1H), 3.87 (s, 3H), 2.29 – 2.21 (m, 4H), 1.62 (s, 3H), 1.52 (s, 3H) ppm

^{13}C NMR (176 MHz, $CDCl_3$) = δ 171.0, 164.1, 134.2, 130.8, 130.0, 121.7, 114.2, 55.8, 36.5, 25.7, 23.2, 17.7 ppm

IR (neat) 3231, 2914, 1696, 1595, 1579, 1498, 1437, 1339, 1261, 1158 cm^{-1}

HRMS (ESI+) *m/z* calculated for $C_{14}H_{19}NO_4S$ $[M+H]^+$: 298.1108, found 298.1117.

R_f = 0.7 (1:1, Hex:EtAOc), one streaky yellow spot, $KMnO_4$, UV.

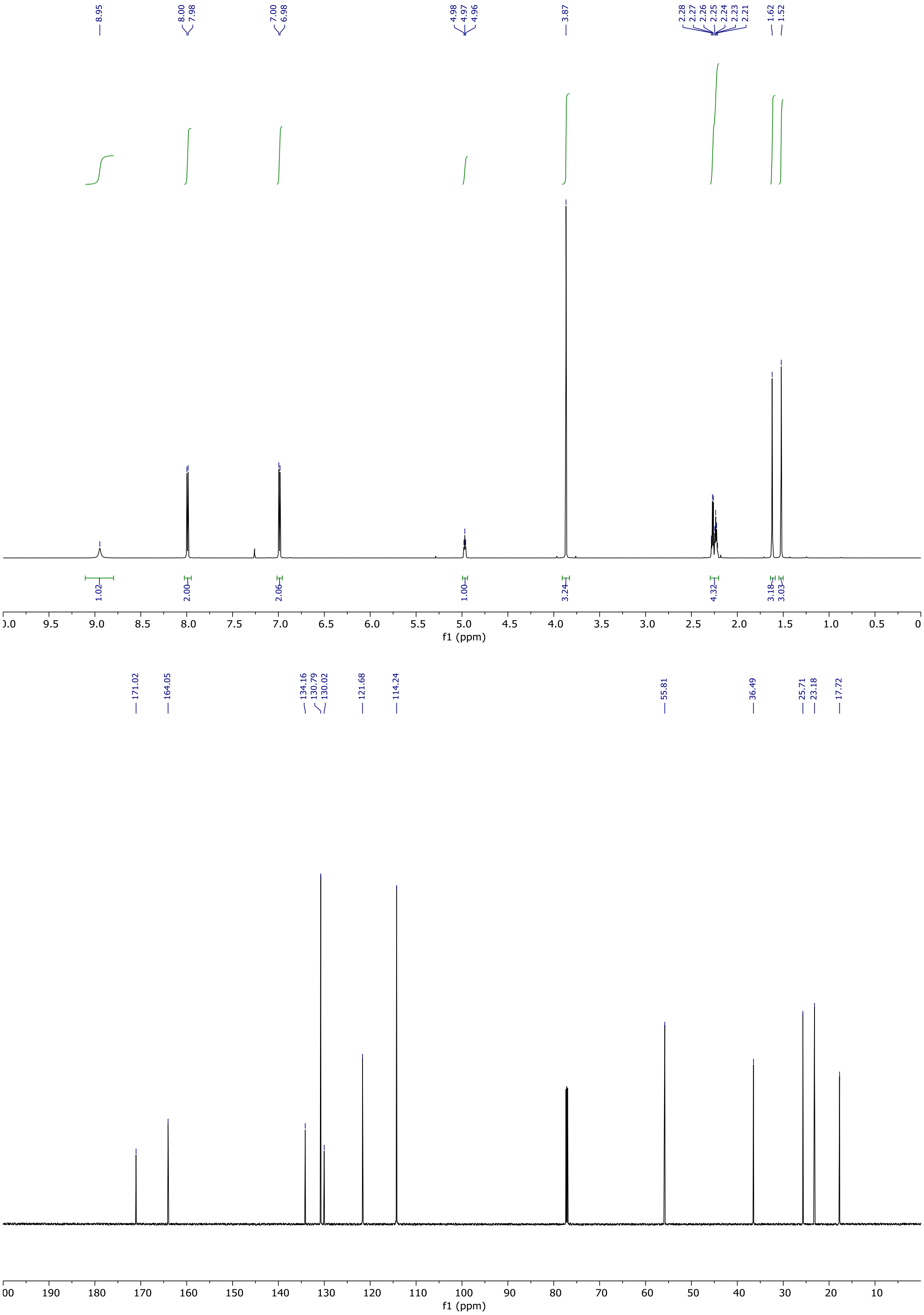

Supplementary Figure 14: ^{1}H (top) and ^{13}C NMR (bottom) for compound **1j** in $CDCl_3$

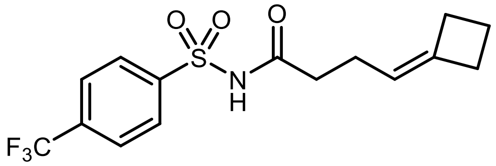

4-cyclobutylidene-N-((4-(trifluoromethyl)phenyl)sulfonyl)butanamide (1k): Prepared according to **General Procedure A**. 175 mg, 41%. Colorless solid.

^{1}H NMR (700 MHz, $CDCl_3$) = δ 8.67 (br s, 1H), 8.22 (d, *J* = 8.3 Hz, 2H), 7.83 (d, *J* = 8.4 Hz, 2H), 4.94 (tp, *J* = 7.2, 2.4 Hz, 1H), 2.58 (t, *J* = 7.9 Hz, 2H), 2.54 (t, *J* = 7.9 Hz, 2H), 2.30 (t, *J* = 7.2 Hz, 2H), 2.15 (q, *J* = 7.2 Hz, 2H), 1.89 (p, *J* = 7.9 Hz, 2H) ppm

^{13}C NMR (176 MHz, $CDCl_3$) = δ 170.8, 143.8, 142.1, 135.8 (q, *J* = 33.3 Hz), 129.2, 126.3 (q, *J* = 3.7 Hz), 123.2 (q, *J* = 273 Hz), 117.2, 36.5, 31.0, 29.3, 23.2, 17.0 ppm

^{19}F NMR (377 MHz, $CDCl_3$) = δ -63.32 ppm

IR (neat) 3134, 2920, 1699, 1458, 1404, 1357, 1319, 1165, 1132, 1089 cm^{-1}

HRMS (ESI+) *m/z* calculated for $C_{15}H_{16}F_3NO_3S$ $[M+H]^+$: 348.0876, found 348.0887.

R_f: (1:9 – Acetone:DCM) = 0.79

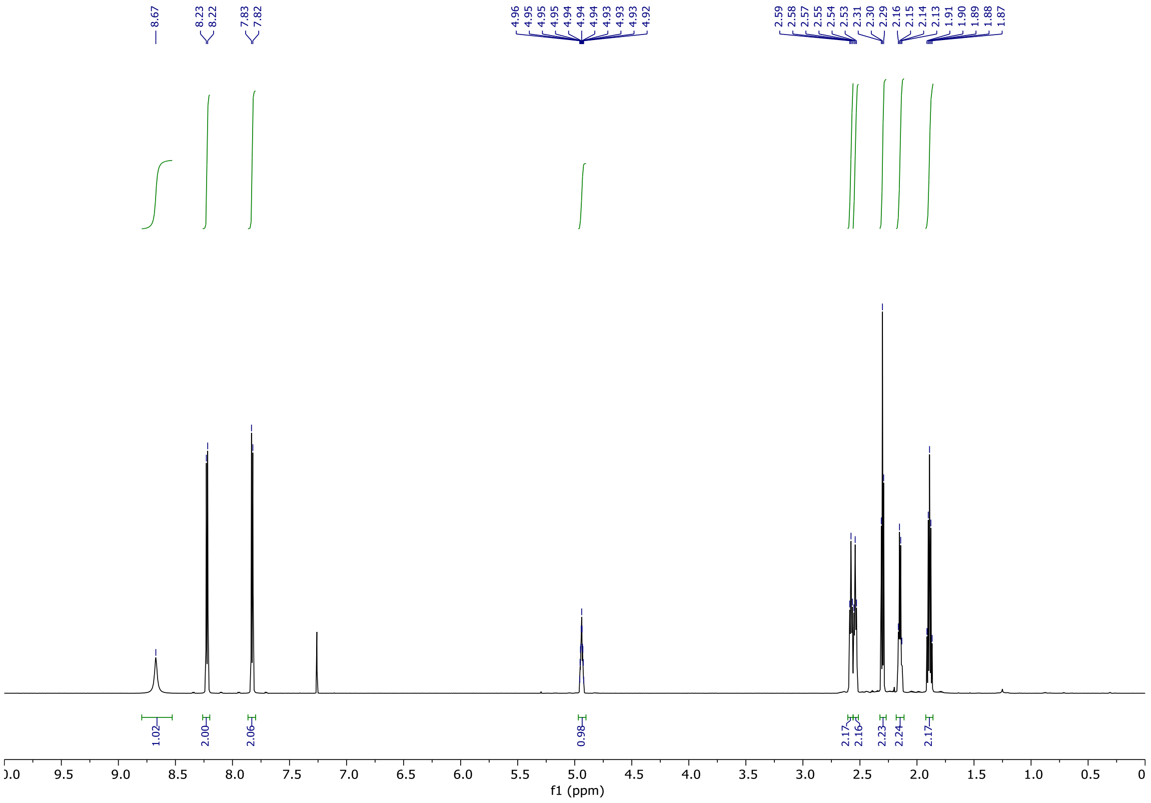

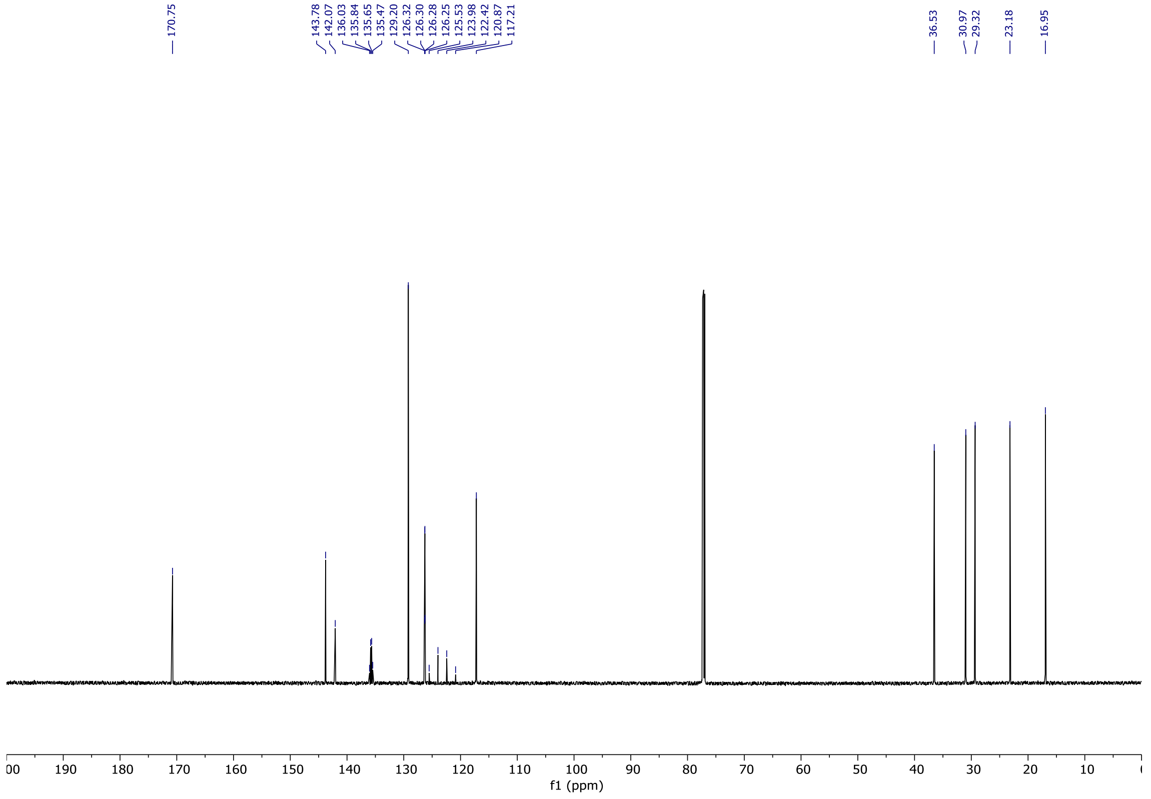

Supplementary Figure 15: ^{1}H (top) and ^{13}C NMR (bottom) for compound **1k** in $CDCl_3$

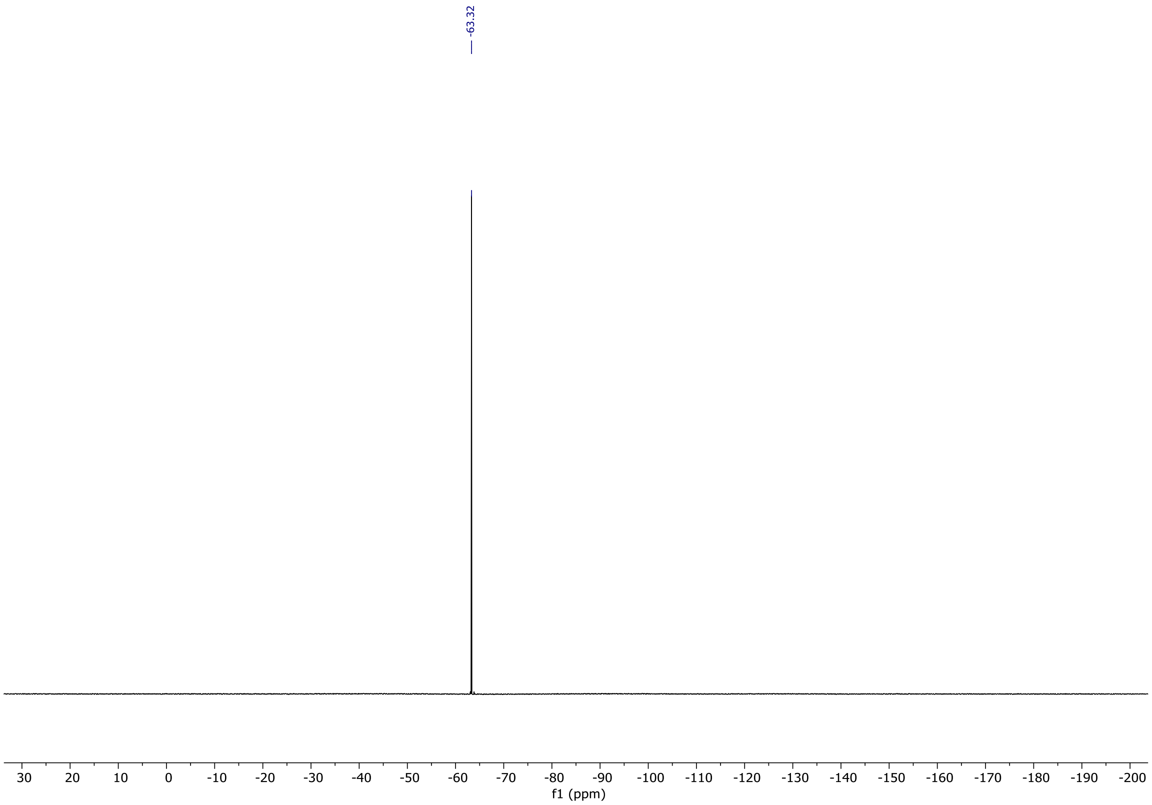

Supplementary Figure 16: ^{19}F NMR for compound **1k** in $CDCl_3$

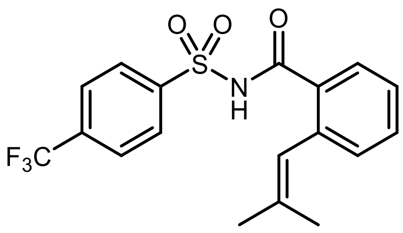

2-(2-methylprop-1-en-1-yl)-N-((4-(trifluoromethyl)phenyl)sulfonyl)benzamide (1l): Prepared according to **General Procedure A**. 194 mg, 81%. Colorless oil.

^{1}H NMR (400 MHz, $CDCl_3$) = δ 9.13 (s, 1H), 8.28 (d, *J* = 8.3 Hz, 2H), 7.84 (d, *J* = 8.4 Hz, 3H), 7.49 (t, *J* = 7.5 Hz, 1H), 7.33 (t, *J* = 7.7 Hz, 1H), 7.17 (d, *J* = 7.7 Hz, 1H), 6.40 (s, 1H), 2.02 (s, 3H), 1.67 (s, 3H) ppm

^{13}C NMR (176 MHz, $CDCl_3$) = δ 165.2, 142.2, 142.2, 137.1, 135.7 (q, *J* = 33.2 Hz), 132.7, 131.1, 130.4, 130.2, 129.4, 127.5, 126.2 (q, *J* = 3.5 Hz), 123.2 (q, *J* = 273 Hz), 122.9, 26.0, 19.5 ppm

^{19}F NMR (377 MHz, $CDCl_3$) = δ -63.30 ppm

IR (neat) 3106, 2912, 1679, 1596, 1428, 1404, 1361, 1321, 1164, 1122 cm^{-1}

HRMS (ESI+) *m/z* calculated for $C_{18}H_{16}F_3NO_3S$ $[M+H]^+$: 384.0876, found 384.0881.

R_f: (1:19 – Acetone:DCM, 1 drop HOAc) = 0.65.

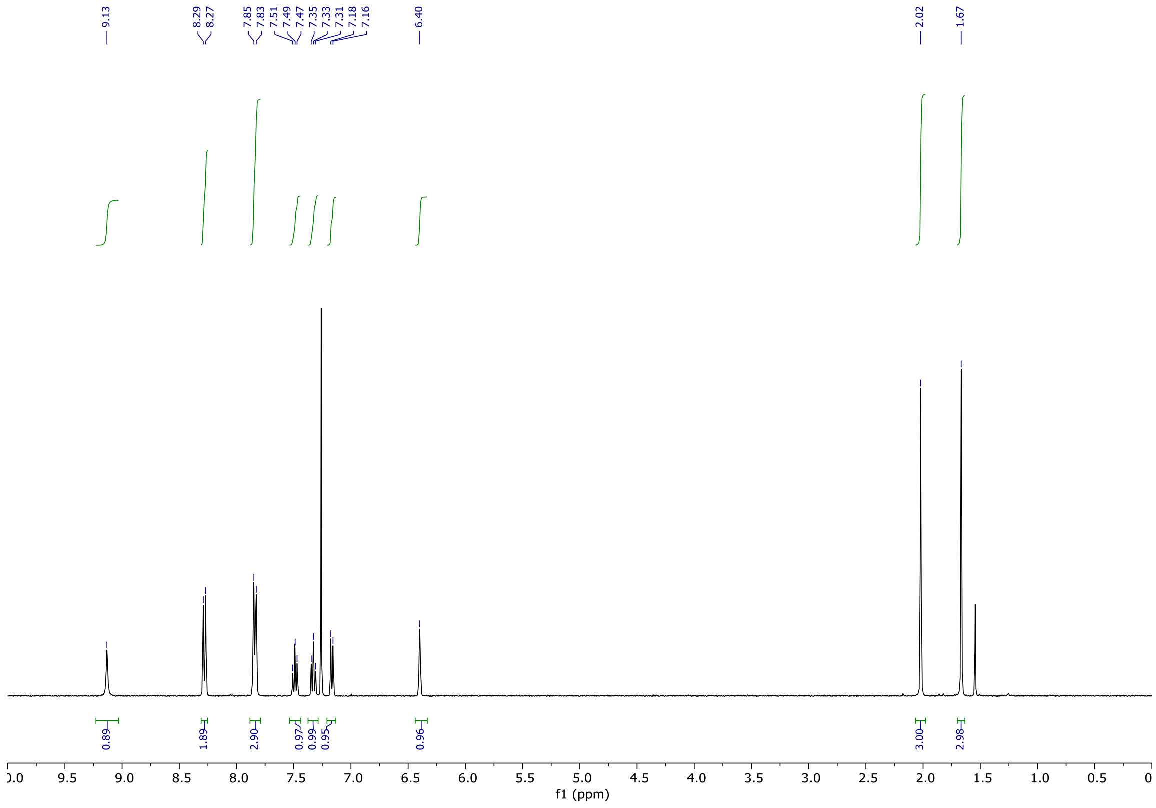

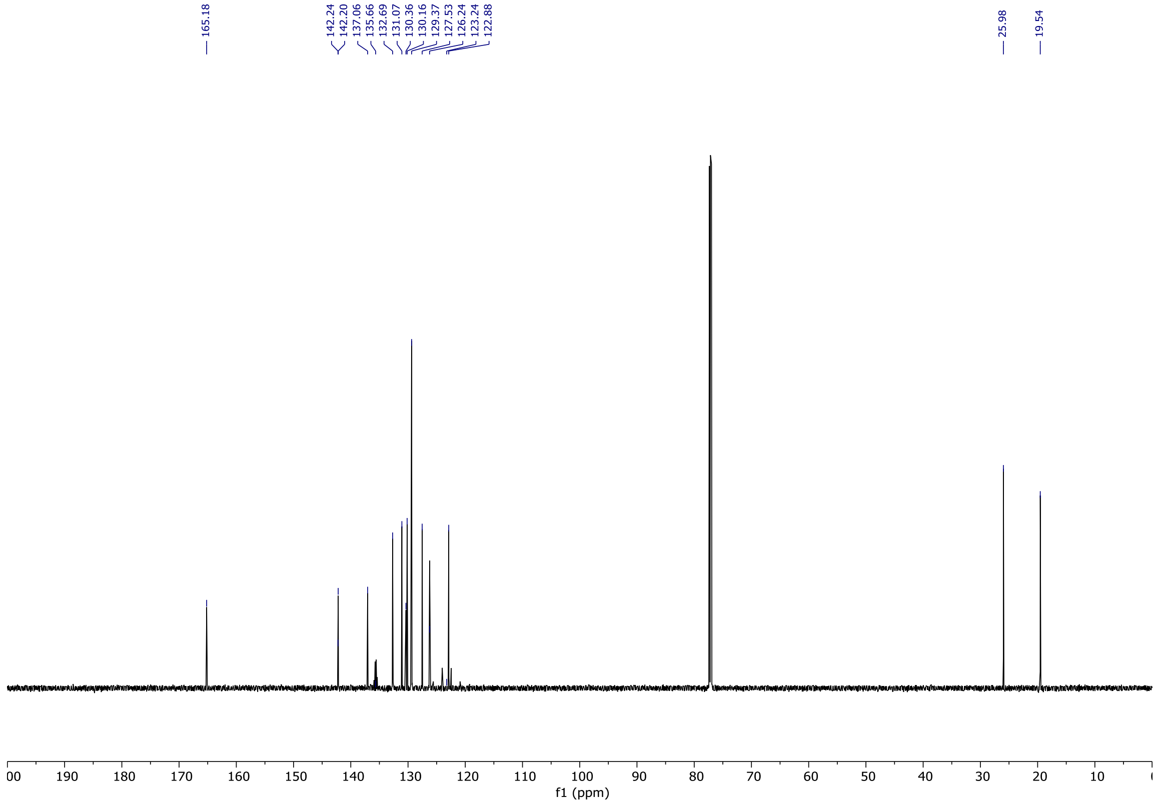

Supplementary Figure 17: ^{1}H (top) and ^{13}C NMR (bottom) for compound **1l** in $CDCl_3$

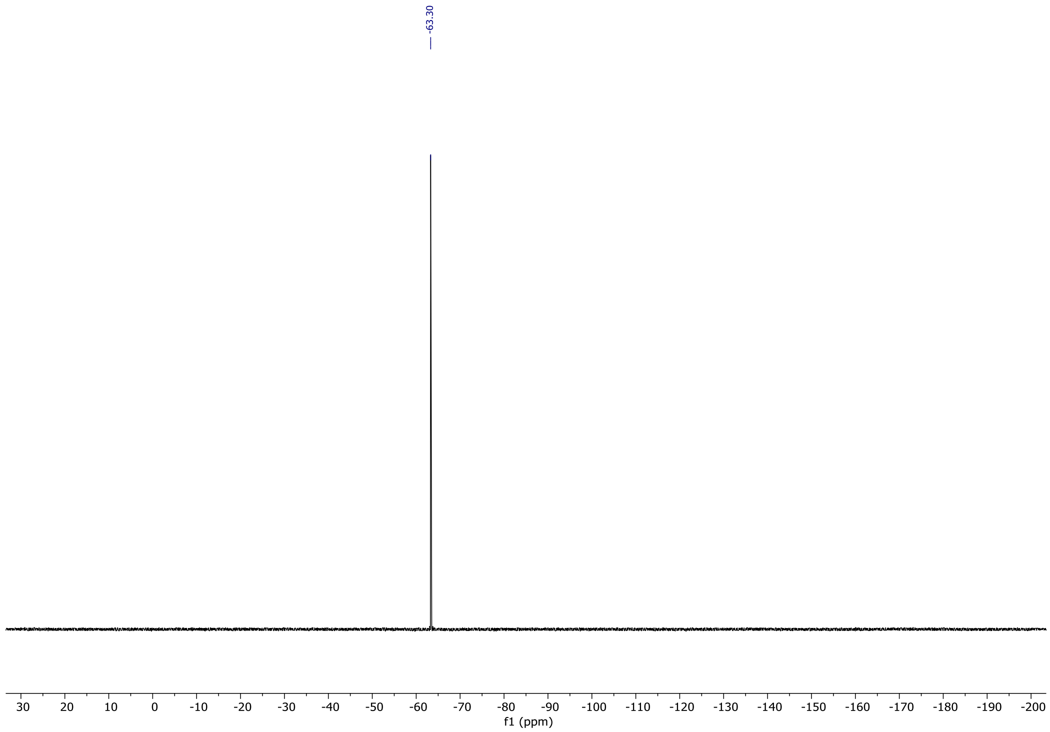

Supplementary Figure 18: ^{19}F NMR for compound **1l** in $CDCl_3$

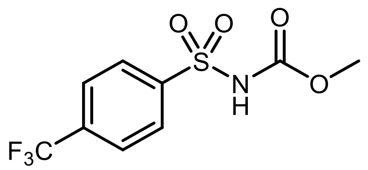

Methyl ((4-(trifluoromethyl)phenyl)sulfonyl)carbamate. In a 100 mL flame-dried flask, 4-(trifluoromethyl)benzenesulfonamide (901 mg, 4.0 mmol, 1 equiv.) was suspended in 40 mL dry dichloromethane. The reaction was cooled to 0 °C, then triethylamine (1.56 mL, 11.2 mmol, 2.8 equiv.) was added dropwise. Methyl chloroformate (0.37 mL, 4.8 mmol, 1.2 equiv.) was added dropwise and the reaction was stirred at 0 °C for 30 minutes. At this point, all starting material was consumed (TLC). The reaction was warmed to room temperature, then transferred to a separatory funnel with 10 mL additional dichloromethane. The organic phase was washed with 50 mL 1 M HCl, 50 mL water, dried over $MgSO_4$, and concentrated under reduced pressure to afford the product as a colorless solid (863 mg, 76%).

Partial characterization is provided below.

^{1}H NMR (400 MHz, $CDCl_3$) = δ 8.20 (d, *J* = 8.2 Hz, 2H), 8.08 – 7.50 (br s, 1H), 7.83 (d, *J* = 8.3 Hz, 2H), 3.73 (s, 3H) ppm

^{19}F NMR (377 MHz, $CDCl_3$) = δ -63.33 ppm

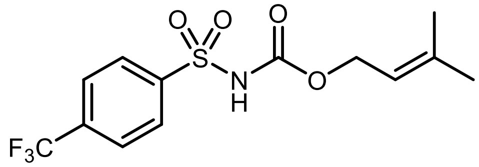

3-methylbut-2-en-1-yl ((4-(trifluoromethyl)phenyl)sulfonyl)carbamate (1m): Prepared according to a modified literature procedure.[12] To a 20 mL microwave vial with a stir bar was added methyl ((4-(trifluoromethyl)phenyl)sulfonyl)carbamate (350 mg, 1.24 mmol, 1 equiv.) and 3-methylbut-2-en-1-ol (10 mL, 98.5 mmol, 80 equiv.). The vial was sealed and heated with microwave irradiation at 100 °C for 30 minutes. Upon cooling to room temperature, the alcohol was distilled off under reduced pressure at 50 °C using a BioChromato Smart Evaporator. The residue was purified using flash chromatography on silica gel (0 – 10% acetone in DCM gradient) to give the product as a colorless solid. (254 mg, 61%).

^{1}H NMR (500 MHz, $CDCl_3$) = δ 8.19 (d, *J* = 8.2 Hz, 2H), 7.82 (d, *J* = 8.3 Hz, 2H), 7.40 (br s, 1H), 5.28 – 5.20 (m, 1H), 4.58 (d, *J* = 7.2 Hz, 2H), 1.74 (s, 3H), 1.66 (s, 3H) ppm

^{13}C NMR (176 MHz, $CDCl_3$) = δ 150.4, 142.0, 141.5, 135.7 (q, *J* = 33.3 Hz), 129.2, 126.3 (q, *J* = 3.7 Hz), 123.2 (q, *J* = 273 Hz), 117.1, 64.3, 25.8, 18.1 ppm

^{19}F NMR (377 MHz, $CDCl_3$) = δ -63.30 ppm

IR (neat) 3257, 1769, 1443, 1407, 1354, 1322, 1213, 1157, 1121, 1112 cm^{-1}

HRMS (ESI+) *m/z* calculated for $C_{13}H_{14}F_3NO_4S$ $[M+NH_4]^+$: 355.0934, found 355.0947.

R_f: (1:19 – Acetone:DCM) = 0.17

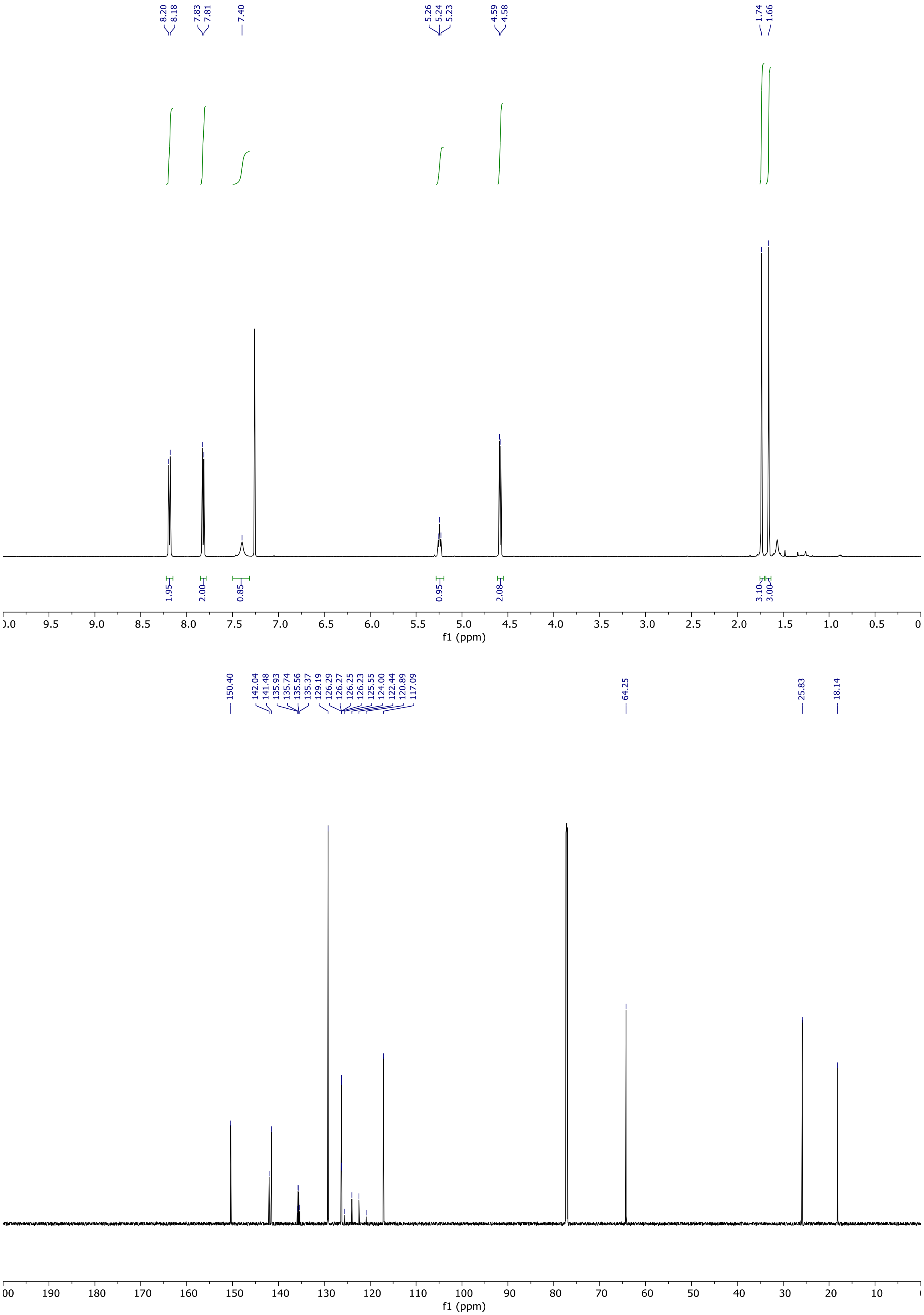

Supplementary Figure 19: ^{1}H (top) and ^{13}C NMR (bottom) for compound **1m** in $CDCl_3$

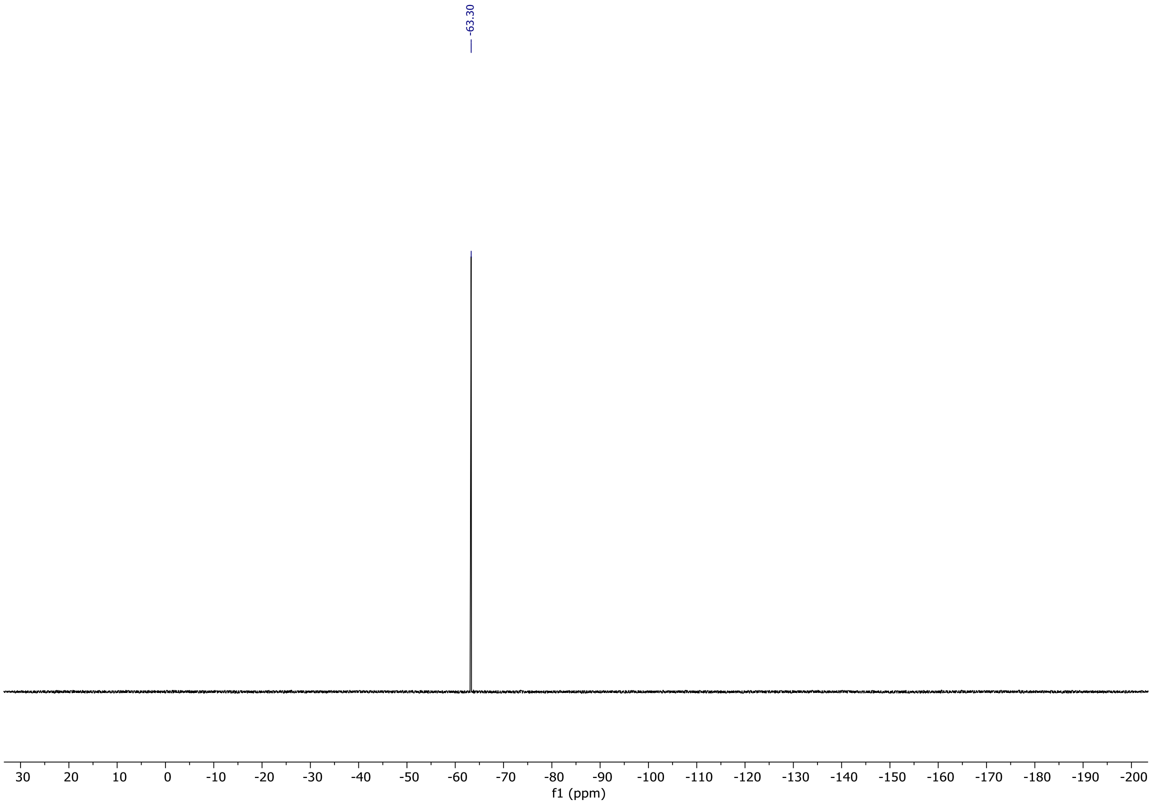

Supplementary Figure 20: ^{19}F NMR for compound **1m** in $CDCl_3$

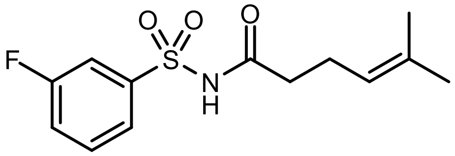

N-((3-fluorophenyl)sulfonyl)-5-methylhex-4-enamide (1n): Prepared according to **General Procedure A**. 260 mg, 46%. Colorless solid.

^{1}H NMR (700 MHz, $CDCl_3$) = δ 8.85 (s, 1H), 7.89 – 7.85 (m, 1H), 7.76 (dt, *J* = 8.0, 2.0 Hz, 1H), 7.54 (td, *J* = 8.1, 5.2 Hz, 1H), 7.35 (tdd, *J* = 8.3, 2.5, 0.6 Hz, 1H), 5.00-4.96 (m, 1H), 2.33 – 2.23 (m, 4H), 1.63 (s, 3H), 1.53 (s, 3H) ppm

^{13}C NMR (176 MHz, $CDCl_3$) = δ 170.9, 162.3 (d, *J* = 252 Hz), 140.6 (d, *J* = 7.2 Hz), 134.6, 130.9 (d, *J* = 7.7 Hz), 124.3 (d, *J* = 3.3 Hz), 121.5, 121.4 (d, *J* = 21 Hz), 115.8 (d, *J* = 25 Hz), 36.6, 25.7, 23.2, 17.7 ppm

^{19}F NMR (376 MHz, $CDCl_3$) = δ -109.30 ppm

IR (neat) 3241, 3068, 2915, 1724, 1594, 1478, 1429, 1406, 1335, 1229, 1118 cm^{-1}

HRMS (ESI+) *m/z* calculated for $C_{13}H_{16}FNO_3S$ $[M+H]^+$: 286.0908, found 286.0907.

R_f: (1:9 – Acetone:DCM) = 0.65.

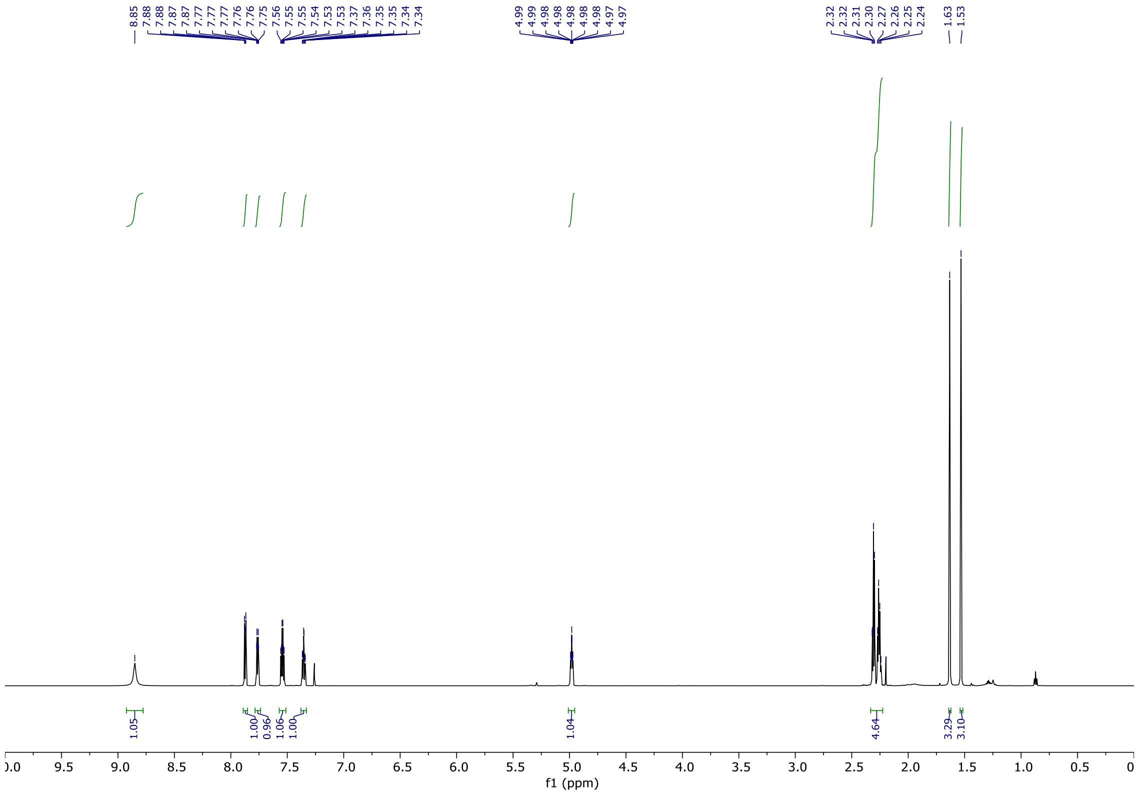

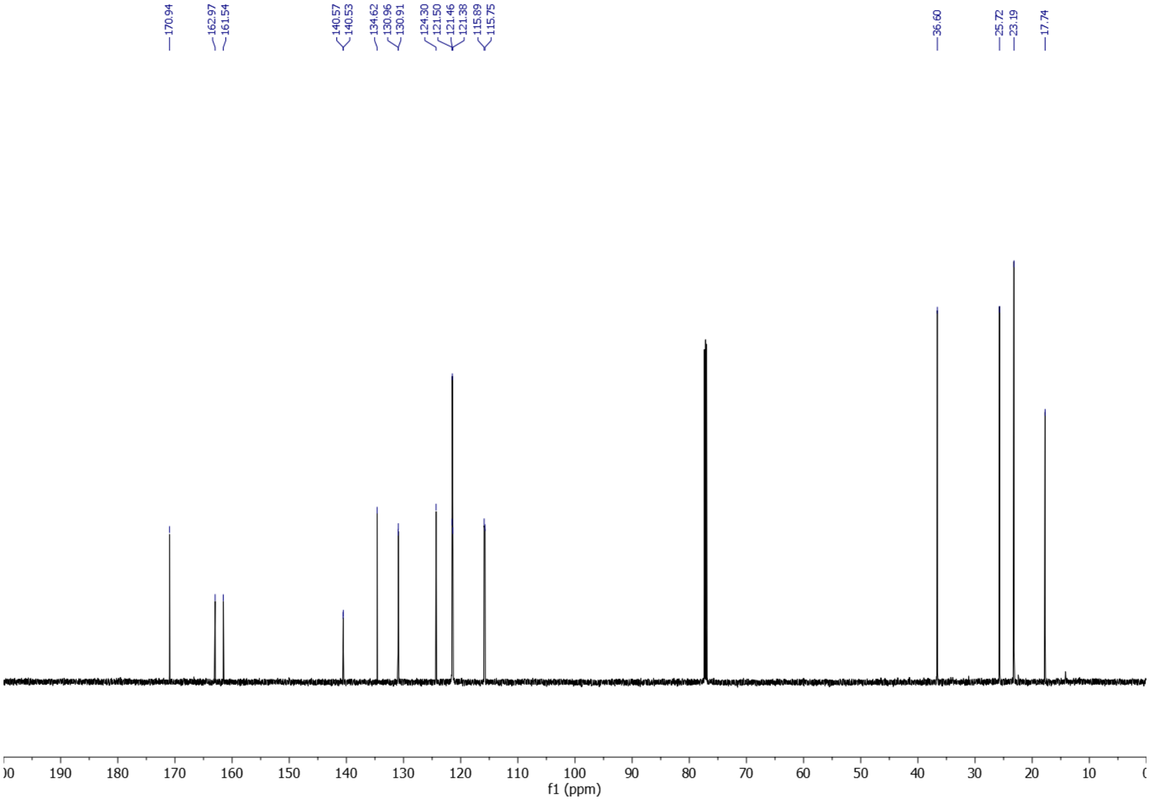

Supplementary Figure 21: ^{1}H (top) and ^{13}C NMR (bottom) for compound **1n** in $CDCl_3$

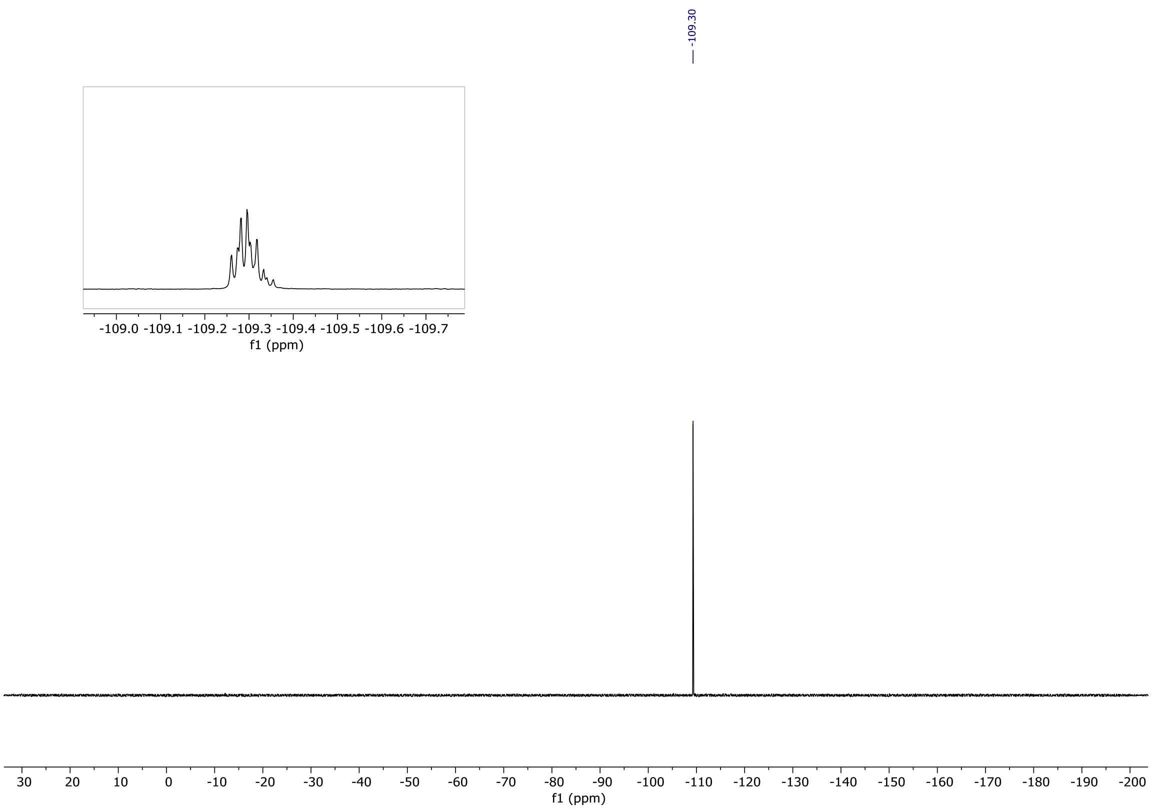

Supplementary Figure 22: ^{19}F NMR for compound **1n** in $CDCl_3$

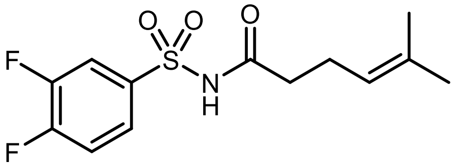

N-((3,4-difluorophenyl)sulfonyl)-5-methylhex-4-enamide (1o): Prepared according to **General Procedure A**. 387 mg, 58%. Colorless solid.

^{1}H NMR (700 MHz, $CDCl_3$) = δ 8.78 (s, 1H), 7.95 – 7.90 (m, 1H), 7.90 – 7.86 (m, 1H), 7.34 (q, *J* = 8.9 Hz, 1H), 4.99 (t, *J* = 7.0 Hz, 1H), 2.33 – 2.23 (m, 4H), 1.64 (s, 3H), 1.55 (s, 3H) ppm

^{13}C NMR (176 MHz, $CDCl_3$) = δ 170.9, 151.5 (dd, *J* = 259.5, 12.7 Hz), 150.1 (dd, *J* = 255, 13.4 Hz), 135.2 (dd, *J* = 5.6, 4.1 Hz), 134.8, 126.0 (dd, *J* = 4.0, 7.8 Hz), 121.4, 118.6 (dd, *J* = 20.5, 1.4 Hz), 118.2 (d, J = 18.7 Hz), 36.6, 25.7, 23.2, 17.8 ppm

^{19}F NMR (376 MHz, $CDCl_3$) = δ -126.95 (m), -133.25 (dt, J = 20.6, 8.5 Hz) ppm

IR (neat) 3196, 2919, 1702, 1606, 1511, 1441, 1348, 1277, 1174, 1123, 1075 cm^{-1}

HRMS (ESI+) *m/z* calculated for $C_{13}H_{15}F_2NO_3S$ $[M+H]^+$: 304.0813, found 304.0817.

R_f: (1:19 – Acetone:DCM, 1 drop HOAc) = 0.71.

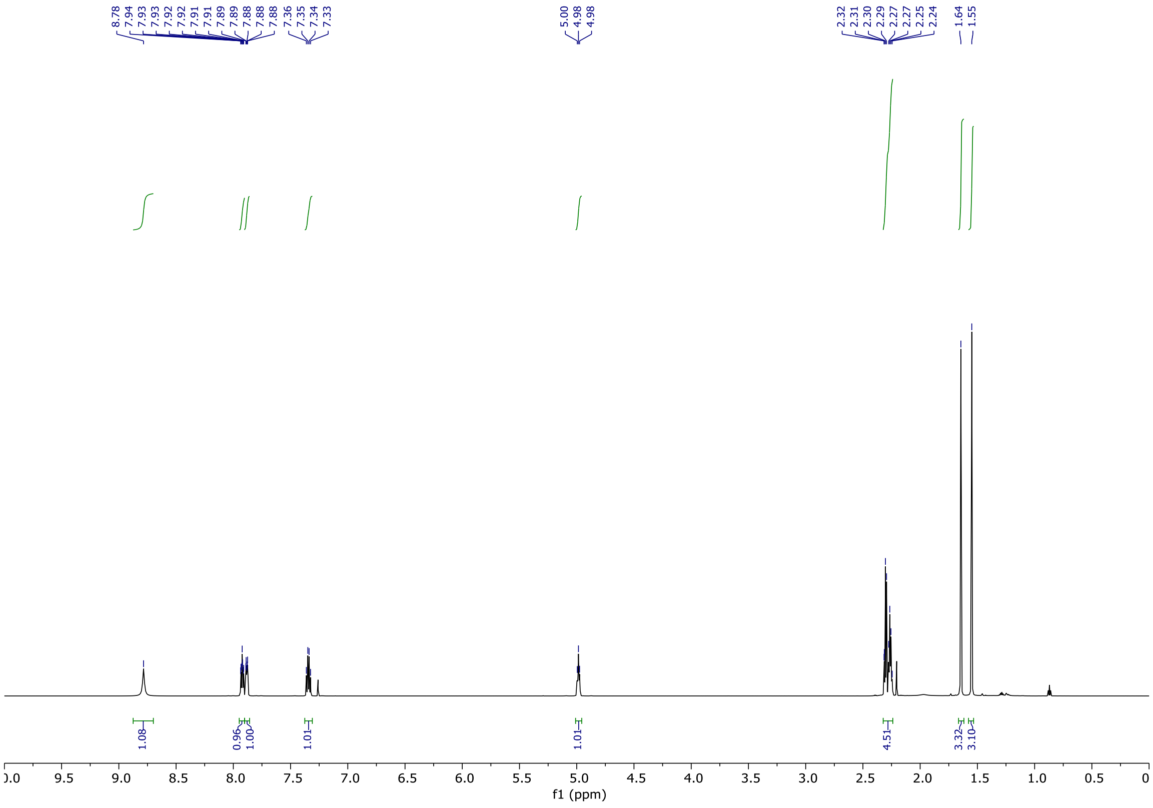

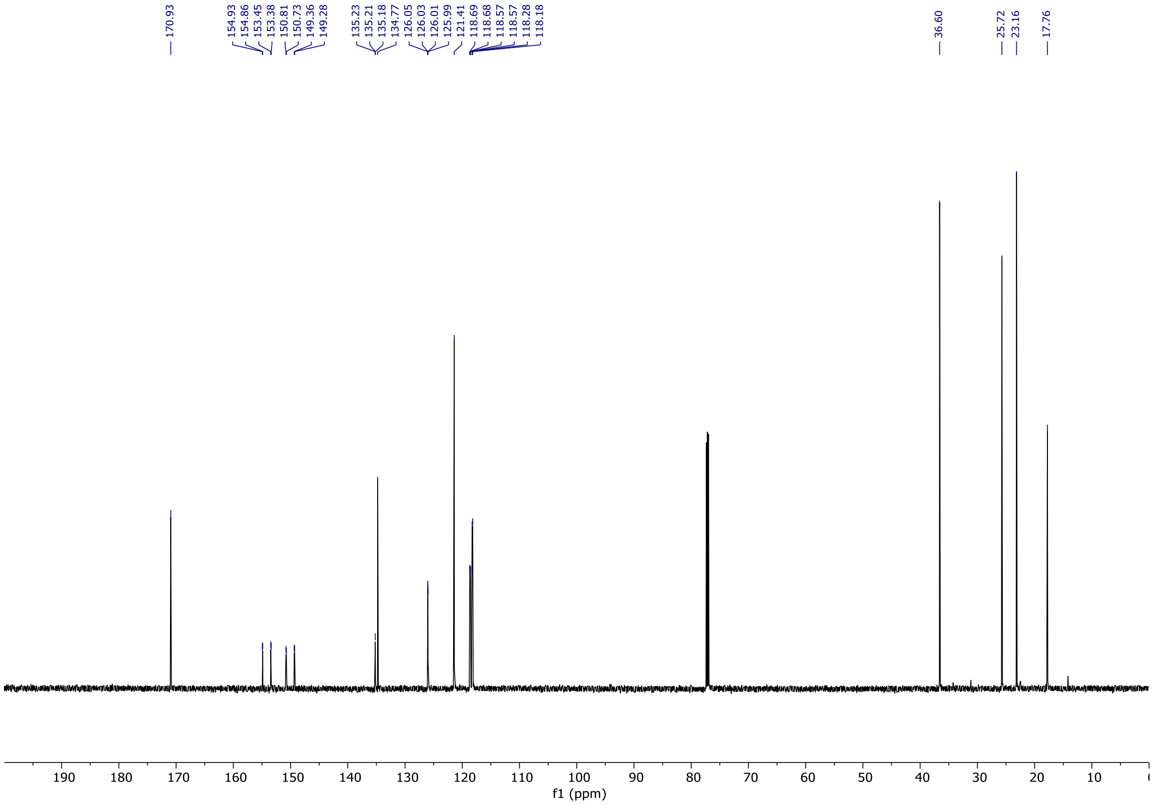

Supplementary Figure 23: ^{1}H (top) and ^{13}C NMR (bottom) for compound **1o** in $CDCl_3$

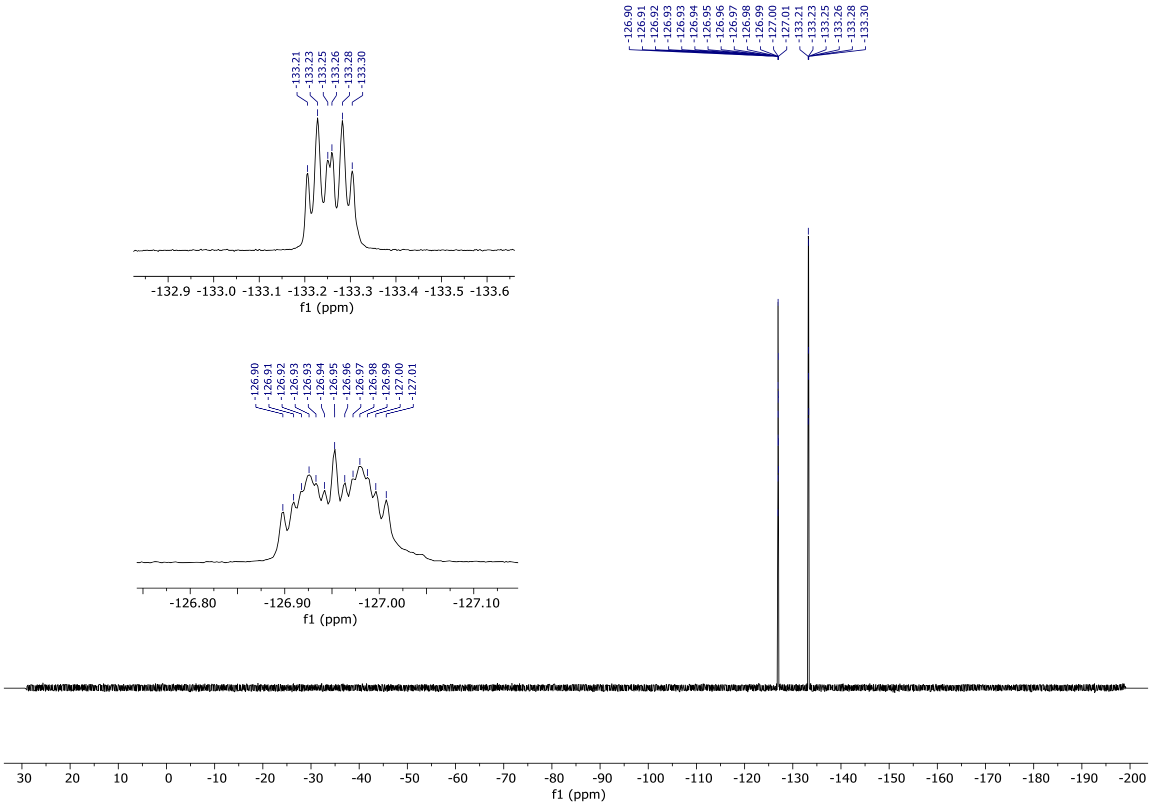

Supplementary Figure 24: ^{19}F NMR for compound **1o** in $CDCl_3$

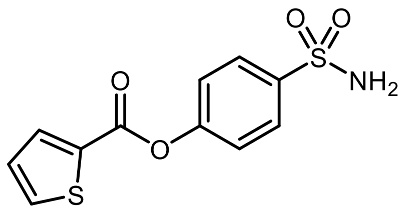

4-sulfamoylphenyl thiophene-2-carboxylate: Prepared according to **General Procedure B**. 318 mg, 51%. Tan solid.

^{1}H NMR: (400 MHz, DMSO-d_6) = δ 8.13 (dd, J = 5.0, 1.3 Hz, 1H), 8.06 (dd, J = 3.8, 1.3 Hz, 1H), 7.91 (d, J = 8.8 Hz, 2H), 7.51 (d, J = 8.8 Hz, 2H), 7.44 (s, 2H), 7.33 (dd, J = 5.0, 3.8 Hz, 1H) ppm

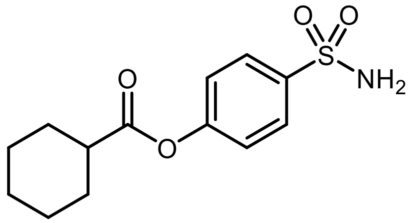

4-sulfamoylphenyl cyclohexanecarboxylate: Prepared according to **General Procedure B**. 430 mg, 69%. Colorless solid.

^{1}H NMR (400 MHz, $CDCl_3$) δ 7.95 (d, J = 8.7 Hz, 2H), 7.23 (d, J = 8.7 Hz, 2H), 4.79 (br s, 2H), 2.58 (tt, J = 11.2, 3.5 Hz, 1H), 2.13 – 2.00 (m, 2H), 1.88 – 1.78 (m, 2H), 1.74 – 1.66 (m, 1H), 1.65 – 1.56 (m, 2H), 1.46 – 1.26 (m, 3H) ppm

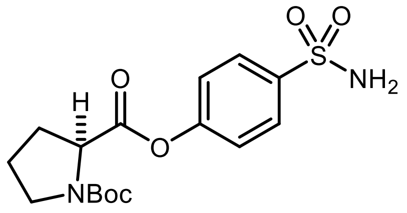

1-(tert-butyl) 2-(4-sulfamoylphenyl) (*S*)-pyrrolidine-1,2-dicarboxylate: Prepared according to **General Procedure B**. 606 mg, 77%. Pale yellow solid. At room temperature, N-Boc rotamers of this compound are well-resolved by ^{1}H NMR.

^{1}H NMR (400 MHz, $CDCl_3$) δ 7.94 (app dd, J = 20.0, 8.7 Hz, 2H), 7.24 (d, J = 8.7 Hz, 2H), 5.08 (br s, 2H), 4.48 (ddd, J = 19.4, 8.5, 4.4 Hz, 1H), 3.67 – 3.40 (m, 2H), 2.48 – 2.29 (m, 1H), 2.23 – 2.11 (m, 1H), 2.11 – 1.88 (m, 2H), 1.45 (app d, J = 13.1 Hz, 9H) ppm

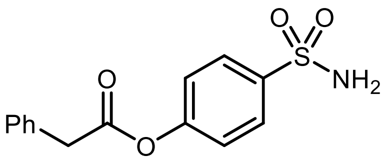

4-sulfamoylphenyl 2-phenylacetate: Prepared according to **General Procedure B**. 446 mg, 70%. Colorless solid.

^{1}H NMR (700 MHz, $CDCl_3$) δ 7.92 (d, *J* = 8.5 Hz, 2H), 7.40 – 7.30 (m, 5H), 7.22 (d, *J* = 8.5 Hz, 2H), 4.84 (br s, 2H), 3.89 (s, 2H) ppm

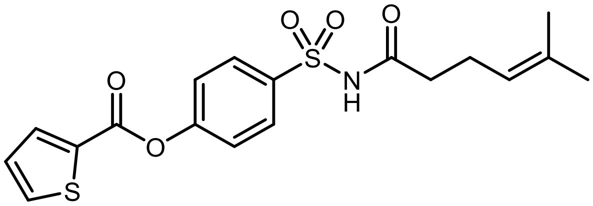

4-(N-(5-methylhex-4-enoyl)sulfamoyl)phenyl thiophene-2-carboxylate (1p): Prepared according to **General Procedure A**. 195 mg, 50%. Colorless solid.

^{1}H NMR (700 MHz, $CDCl_3$) = δ 8.60 (br s, 1H), 8.14 (d, *J* = 8.7 Hz, 2H), 8.00 (dd, *J* = 3.8, 0.9 Hz, 1H), 7.72 (dd, *J* = 5.0, 0.9 Hz, 1H), 7.43 (d, *J* = 8.7 Hz, 2H), 7.21 – 7.19 (m, 1H), 5.01 (t, *J* = 6.9 Hz, 1H), 2.33 – 2.23 (m, 4H), 1.66 (s, 3H), 1.56 (s, 3H) ppm

^{13}C NMR (176 MHz, $CDCl_3$) = δ 170.5, 159.7, 154.8, 135.8, 135.4, 134.5, 134.4, 131.8, 130.3, 128.3, 122.3, 121.5, 36.5, 25.6, 23.1, 17.7 ppm

IR (neat): 3244, 2254, 1718, 1434, 1339, 1252, 1203, 1179, 1157, 1060 cm^{-1}

HRMS (ESI+) *m/z* calculated for $C_{18}H_{19}NO_5S_2$ $[M+H]^+$: 394.0777, found 394.0782.

R_f: (1:6 – Acetone:DCM) = 0.81.

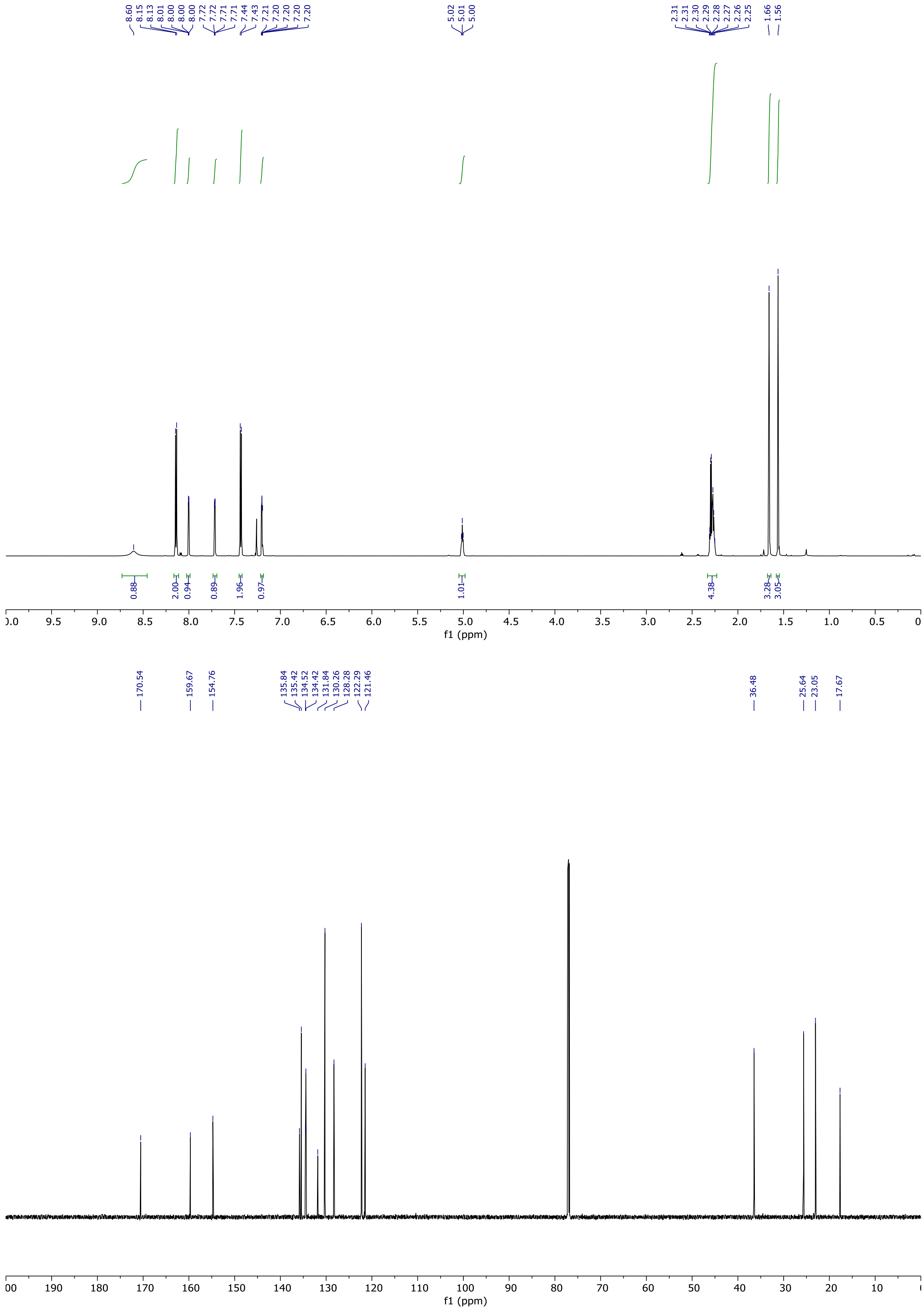

Supplementary Figure 25: ^{1}H (top) and ^{13}C NMR (bottom) for compound **1p** in $CDCl_3$

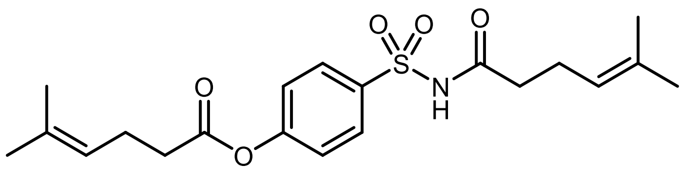

4-(N-(5-methylhex-4-enoyl)sulfamoyl)phenyl 5-methylhex-4-enoate (1q): Prepared according to **General Procedure A** from 4-hydroxybenzenesulfonamide using 2.1 equiv. of 5-methylhex-4-enoic acid, 2.6 equiv. EDC-HCl, and 0.2 equiv DMAP. 113 mg, 57%. Viscous, colorless oil.

^{1}H NMR (700 MHz, $CDCl_3$) = δ 8.35 (br s, 1H), 8.09 (d, *J* = 8.8 Hz, 2H), 7.27 (d, *J* = 8.5 Hz, 2H), 5.16 (t, *J* = 7.2 Hz, 1H), 5.02 – 4.98 (m, 1H), 2.61 (t, *J* = 7.4 Hz, 2H), 2.44 (q, *J* = 7.3 Hz, 2H), 2.30 – 2.23 (m, 4H), 1.72 (s, 3H), 1.66 (s, 6H), 1.56 (s, 3H) ppm

^{13}C NMR (176 MHz, $CDCl_3$) = δ 171.2, 170.4, 155.2, 135.7, 134.8, 134.1, 130.3, 122.4, 121.8, 121.6, 36.6, 34.7, 25.9, 25.8, 23.6, 23.2, 17.9, 17.8 ppm

IR (neat) 3242, 2915, 1763, 1723, 1696, 1590, 1435, 1204, 1177, 1159 cm^{-1}

HRMS (ESI+) *m/z* calculated for $C_{20}H_{27}NO_5S$ $[M+H]^+$: 394.1683, found 394.1688.

R_f: (1:19 – Acetone:DCM) = 0.68.

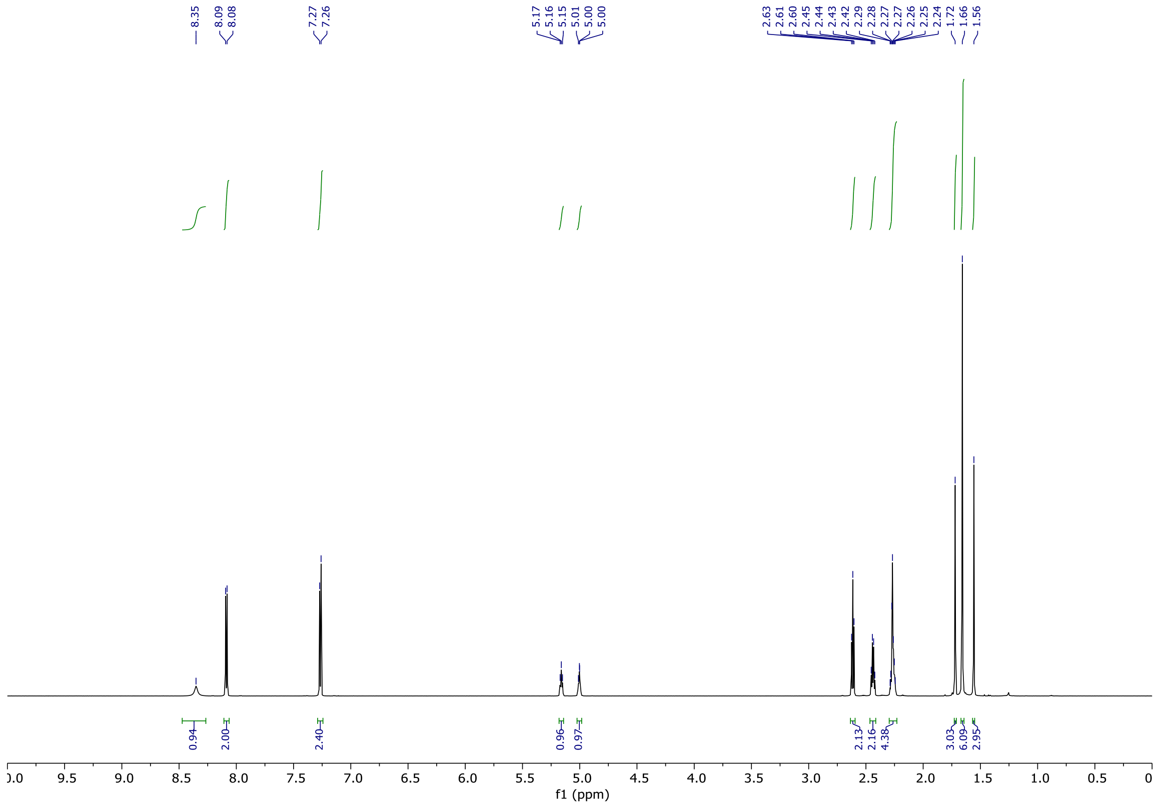

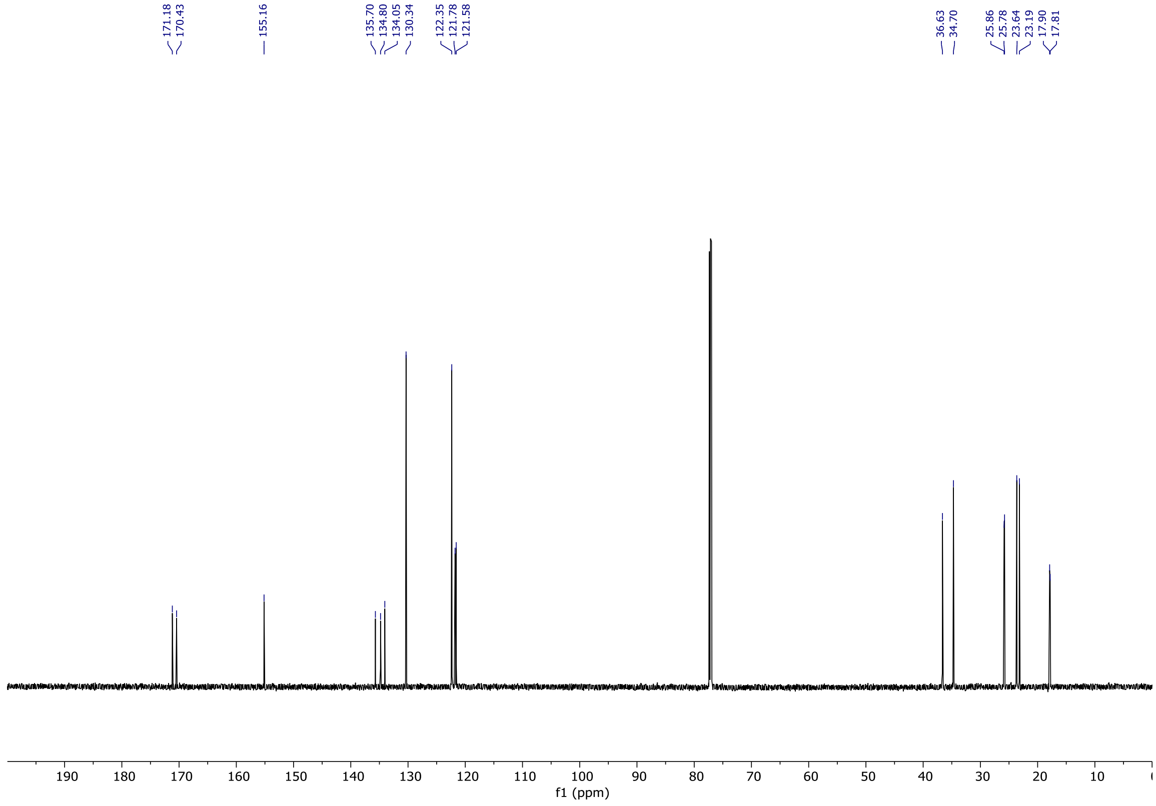

Supplementary Figure 26: ^{1}H (top) and ^{13}C NMR (bottom) for compound **1q** in $CDCl_3$

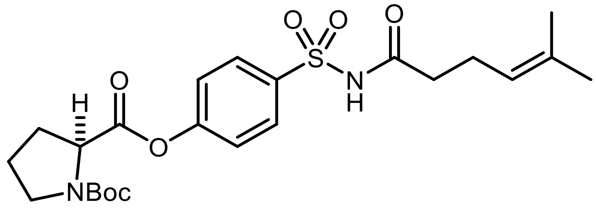

1-(tert-butyl) 2-(4-(N-(5-methylhex-4-enoyl)sulfamoyl)phenyl) (S)-pyrrolidine-1,2-dicarboxylate (1r): Prepared according to **General Procedure A**. 458 mg, 58%. Clear, glassy solid. At room temperature, N-Boc rotamers of this compound are well-resolved by ^{1}H and ^{13}C NMR.

^{1}H NMR (500 MHz, $CDCl_3$) = δ 8.64 (br s, 1H), 8.09 (app dd, *J* = 19.8, 8.5 Hz, 2H), 7.29 (app t, *J* = 8.1 Hz, 2H), 5.03 – 4.96 (m, 1H), 4.50 (app ddd, *J* = 31.8, 8.5, 4.4 Hz, 1H), 3.67 – 3.42 (m, 2H), 2.47 – 2.31 (m, 1H), 2.31 – 2.21 (m, 4H), 2.21 – 2.10 (m, 1H), 2.10 – 1.91 (m, 2H), 1.64 (s, 3H), 1.54 (s, 3H), 1.46 (app d, *J* = 13.6 Hz, 9H).

^{13}C NMR (176 MHz, $CDCl_3$) = δ 171.1, 170.9, 170.6, 170.6, 155.1, 154.8, 154.7, 153.8, 136.1, 136.0, 134.6, 134.6, 130.5, 130.3, 122.3, 121.9, 121.6, 121.6, 80.7, 80.5, 59.3, 59.2, 46.8, 46.6, 45.2, 36.6, 31.1, 30.1, 28.8, 28.6, 25.8, 25.5, 25.3, 23.9, 23.2, 17.8 ppm

IR (neat) 3202, 2976, 2931, 1772, 1670, 1403, 1366, 1347, 1204, 1124 cm^{-1}

HRMS (ESI+) *m/z* calculated for $C_{23}H_{32}N_2O_7S$ $[M+H]^+$: 481.2003, found 481.2013

R_f: (1:6 – Acetone:DCM) = 0.62

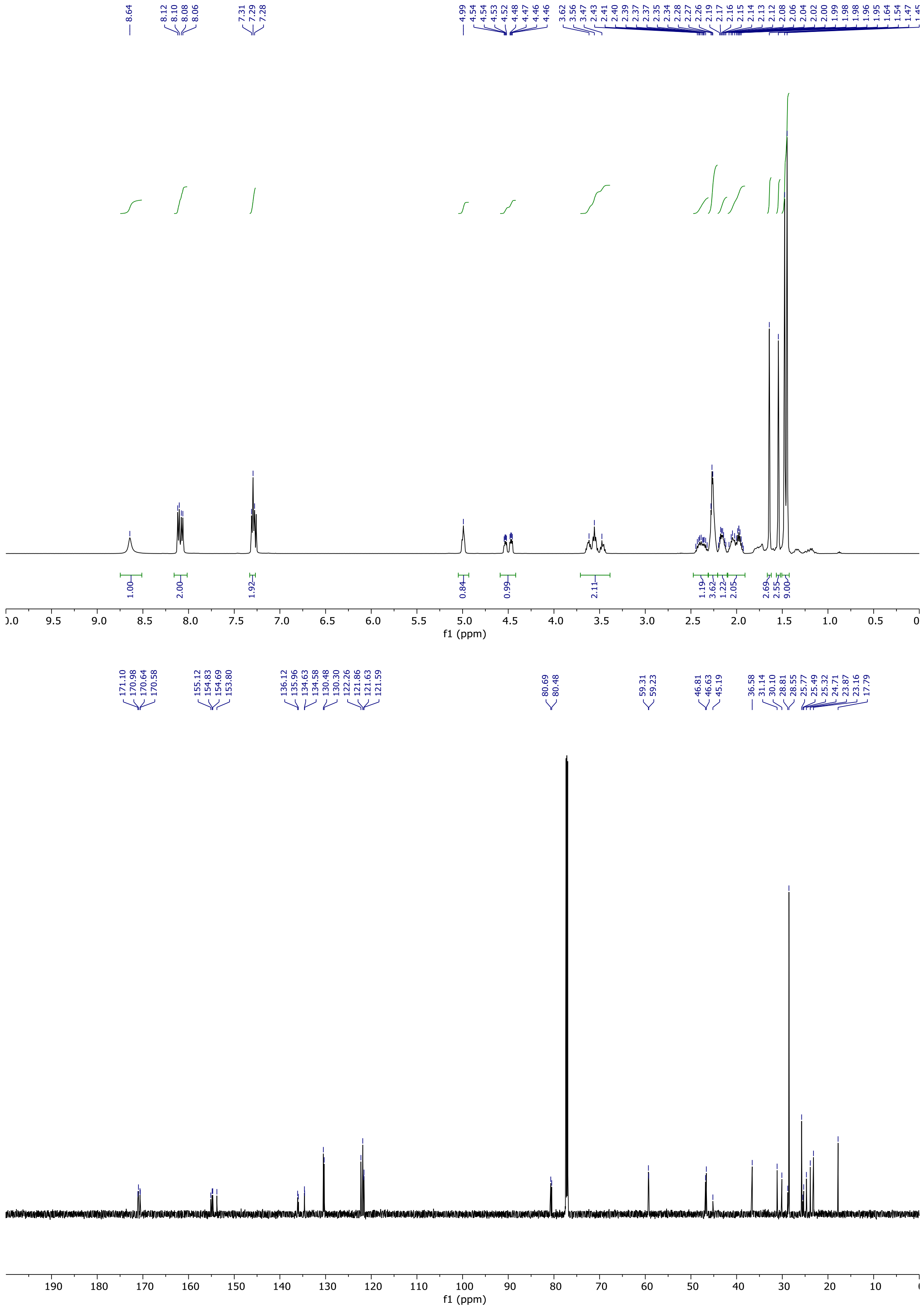

Supplementary Figure 27: ^{1}H (top) and ^{13}C NMR (bottom) for compound **1r** in $CDCl_3$

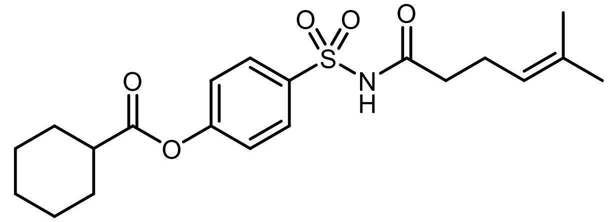

4-(N-(5-methylhex-4-enoyl)sulfamoyl)phenyl cyclohexanecarboxylate (1s): Prepared according to **General Procedure A**. 345 mg, 69%. Colorless solid.

^{1}H NMR (700 MHz, $CDCl_3$) = δ 8.38 (br s, 1H), 8.08 (d, *J* = 8.8 Hz, 2H), 7.26 (d, *J* = 8.8 Hz, 2H), 5.03 – 4.98 (m, 1H), 2.58 (tt, *J* = 11.2, 3.4 Hz, 1H), 2.29 – 2.23 (m, 4H), 2.09 – 2.03 (m, 2H), 1.85 – 1.79 (m, 2H), 1.72 – 1.67 (m, 1H), 1.66 (s, 3H), 1.63 – 1.57 (m, 2H) 1.56 (s, 3H), 1.41 – 1.27 (m, 3H) ppm

^{13}C NMR (176 MHz, $CDCl_3$) = δ 173.8, 170.4, 155.4, 135.6, 134.8, 130.3, 122.3, 121.6, 43.3, 36.6, 29.0, 25.8, 25.8, 25.4, 23.2, 17.8 ppm

IR (neat) 3255, 2932, 2854, 1755, 1721, 1685, 1585, 1448, 1406, 1147 cm^{-1}

HRMS (ESI+) *m/z* calculated for $C_{20}H_{27}NO_5S$ $[M+H]^+$: 394.1683, found 394.1684.

R_f: (2:3 – EtOAc:Hex) = 0.52.

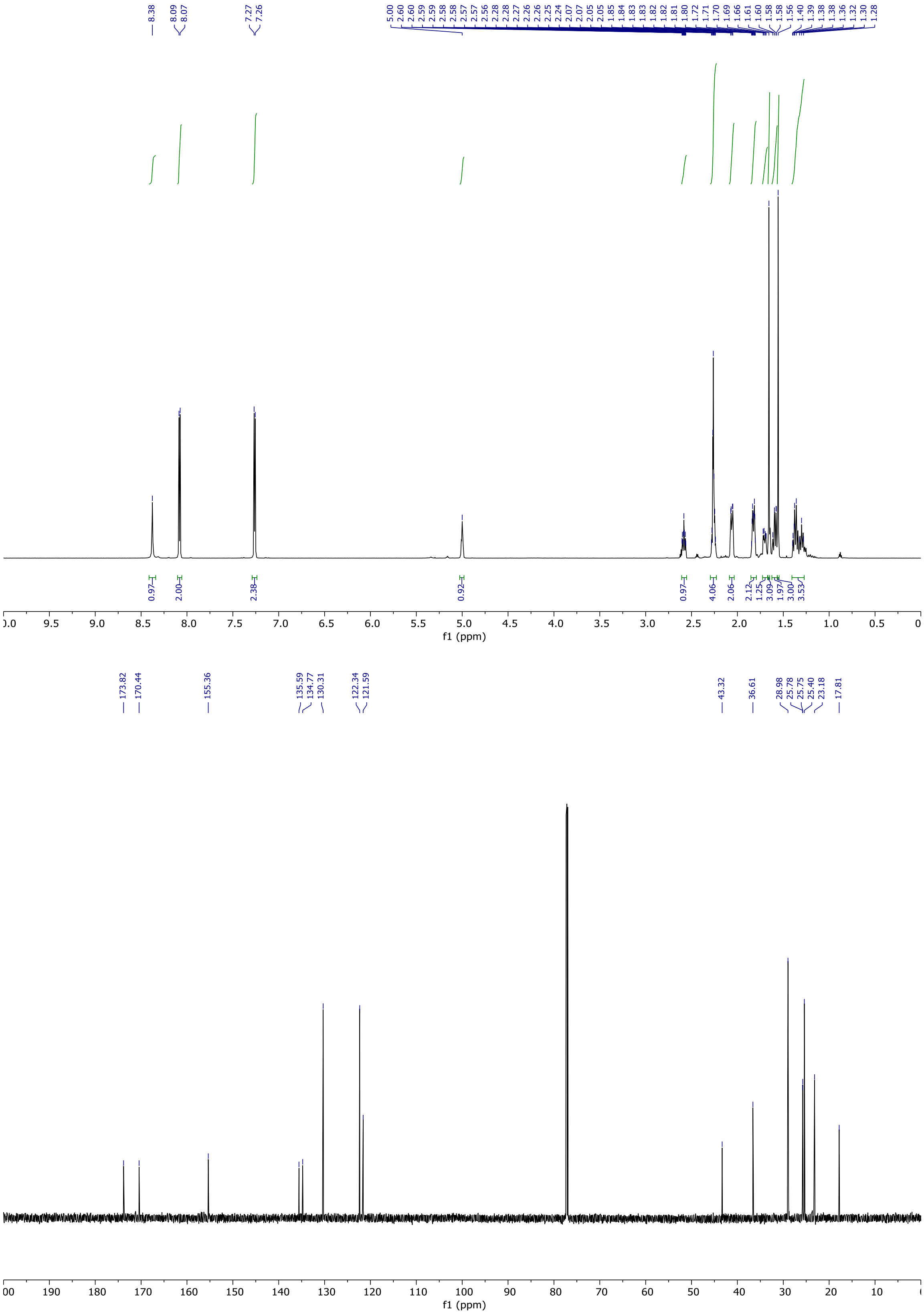

Supplementary Figure 28: ^{1}H (top) and ^{13}C NMR (bottom) for compound **1s** in $CDCl_3$

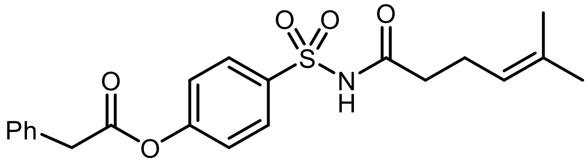

4-(N-(5-methylhex-4-enoyl)sulfamoyl)phenyl 2-phenylacetate (1t): Prepared according to **General Procedure A**. 131 mg, 55%. Colorless solid. Isolated with a small amount of an inseparable impurity (<10%).

^{1}H NMR (700 MHz, $CDCl_3$) = δ 8.80 (s, 1H), 8.07 (d, *J* = 8.8 Hz, 2H), 7.46 – 7.30 (m, 5H), 7.27 (d, *J* = 8.8 Hz, 2H), 5.01 – 4.94 (m, 1H), 3.90 (s, 2H), 2.27 – 2.21 (m, 4H), 1.63 (s, 3H), 1.53 (s, 3H) ppm

^{13}C NMR (176 MHz, $CDCl_3$) = δ 170.8, 169.3, 154.9, 135.8, 134.3, 132.8, 130.2, 129.3, 128.9, 127.6, 122.1, 121.5, 41.3, 36.4, 25.6, 23.0, 17.6 ppm

IR (neat) 3122, 2930, 1751, 1686, 1587, 1445, 1406, 1354, 1209, 1188 cm^{-1}

HRMS (ESI+) *m/z* calculated for $C_{21}H_{23}NO_5S$ $[M+H]^+$: 402.1370, found 402.1377

R_f: (2:3 – EtOAc:Hex) = 0.47.

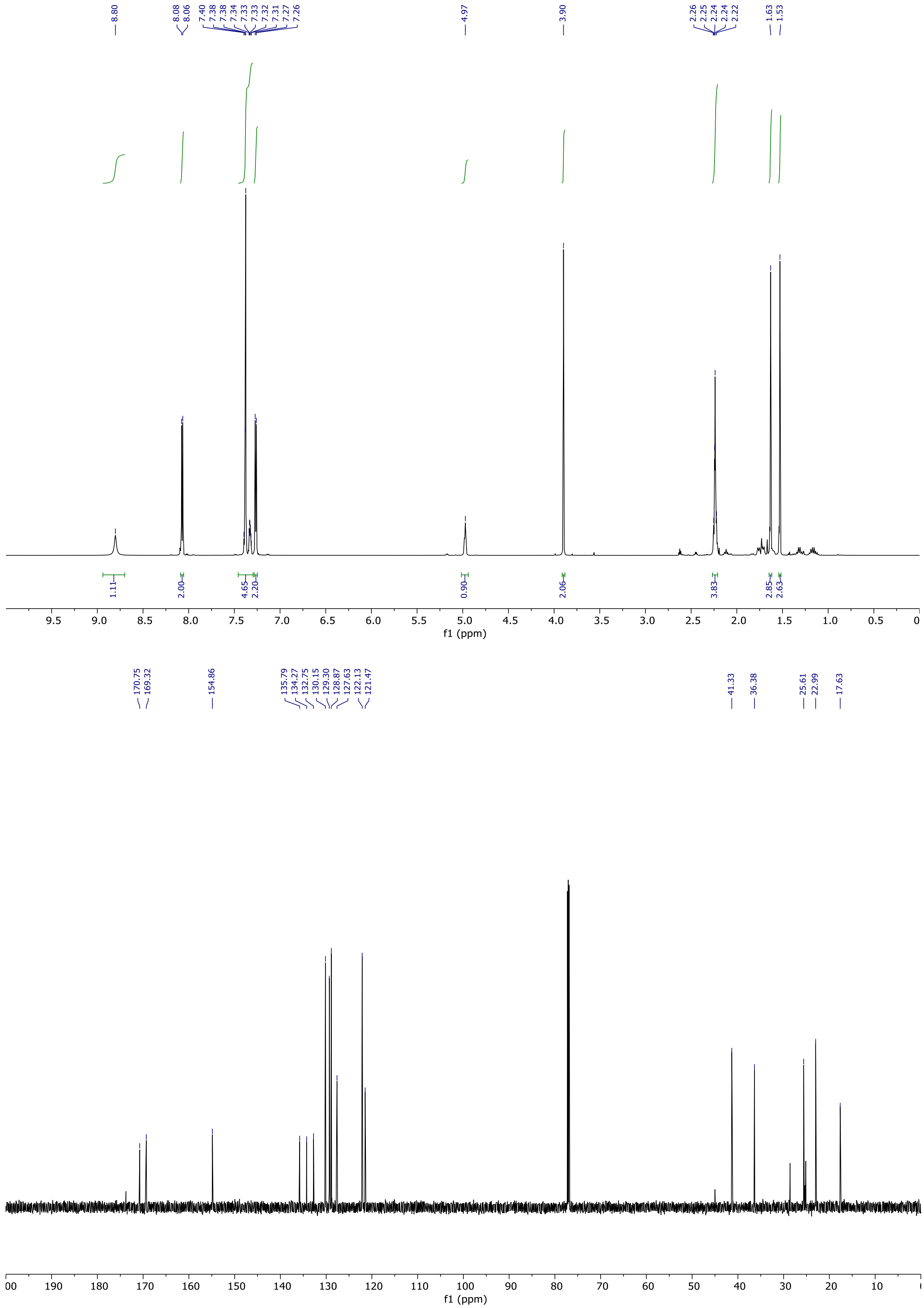

Supplementary Figure 29: ^{1}H (top) and ^{13}C NMR (bottom) for compound **1t** in $CDCl_3$

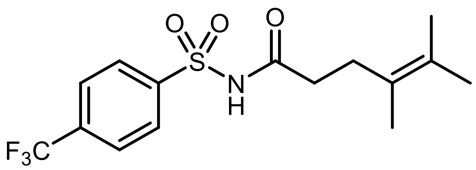

4,5-dimethyl-N-((4-(trifluoromethyl)phenyl)sulfonyl)hex-4-enamide (1u): Prepared according to **General Procedure A**. 238 mg, 68%. Colorless solid. Isolated with a small amount of an inseparable impurity (<10%).

^{1}H NMR (400 MHz, $CDCl_3$) = δ 8.21 (d, *J* = 8.2 Hz, 2H), 8.05 (br s, 1H), 7.82 (d, *J* = 8.3 Hz, 2H), 2.32 (app s, 4H), 1.63 (s, 3H), 1.59 (s, 3H), 1.58 (s, 3H) ppm

^{13}C NMR (176 MHz, $CDCl_3$) = δ 170.8, 142.0, 135.8, 129.2, 127.6, 126.3, 124.8, 123.2, 35.1, 29.2, 20.8, 20.3, 17.9 ppm

^{19}F NMR (376 MHz, $CDCl_3$) = δ -63.33 ppm

IR (neat) 3266, 2931, 2862, 1730, 1421, 1404, 1344, 1320, 1172, 1138 cm^{-1}

HRMS (ESI+) *m/z* calculated for $C_{15}H_{18}F_3NO_3S$ $[M+H]^+$: 350.1032, found 350.1034

R_f: (1:19 – Acetone:DCM) = 0.74.

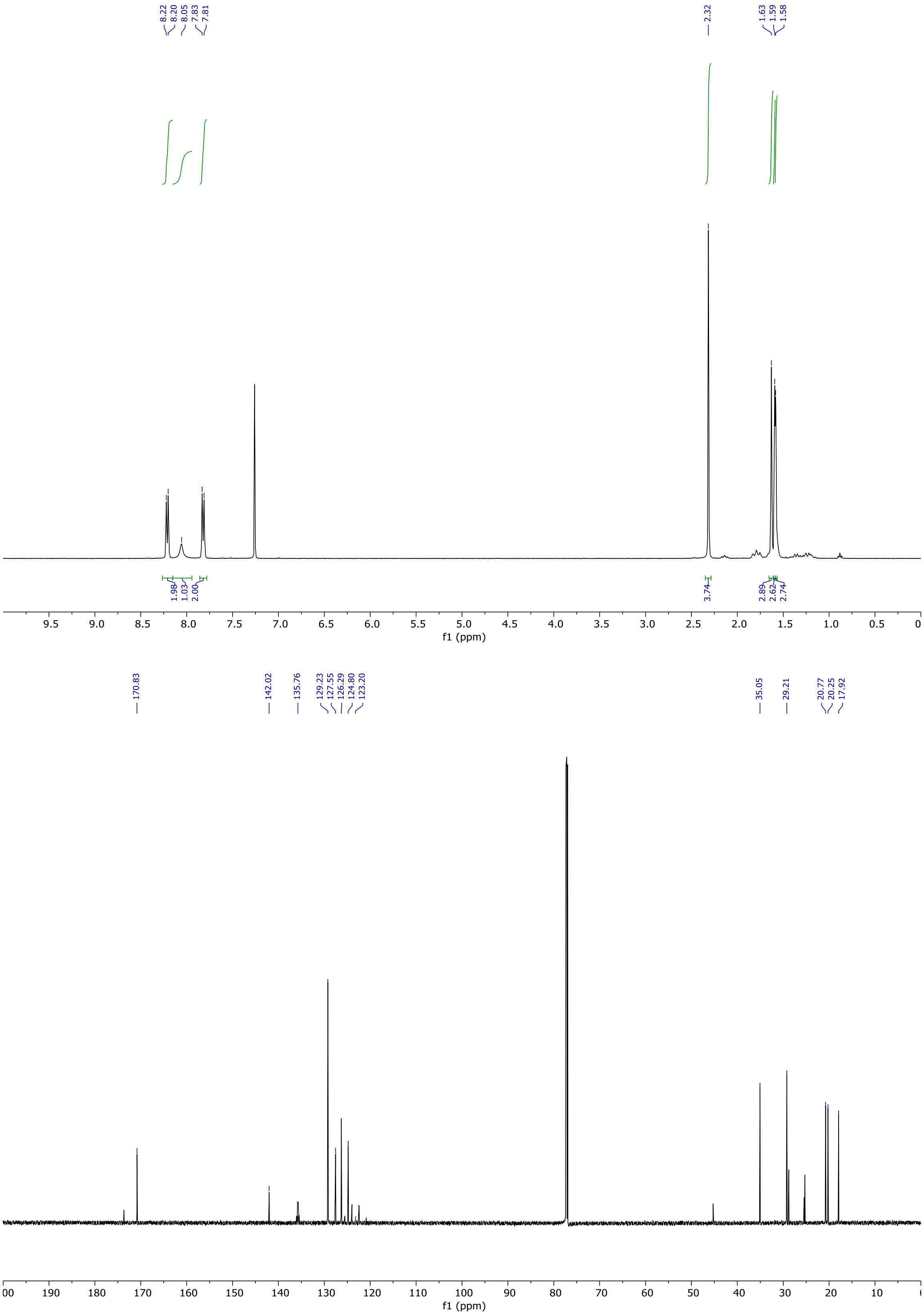

Supplementary Figure 30: ^{1}H (top) and ^{13}C NMR (bottom) for compound **1u** in $CDCl_3$

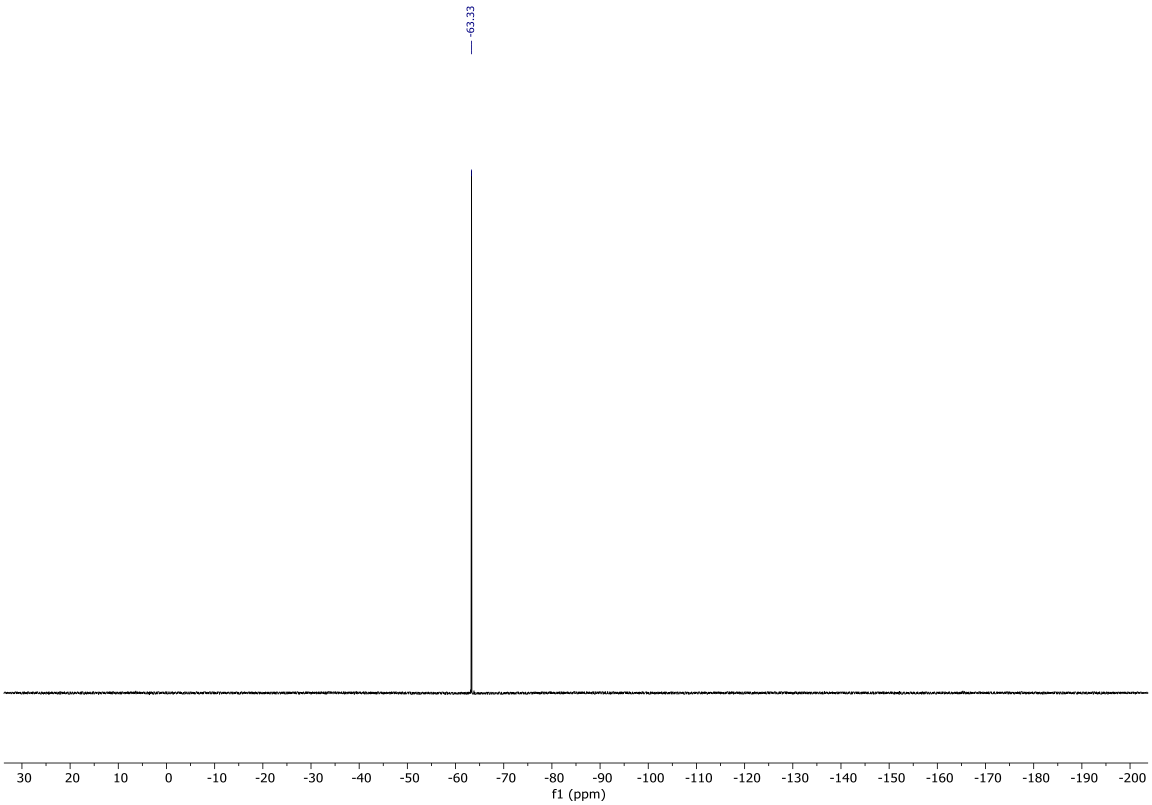

Supplementary Figure 31: ^{19}F NMR for compound **1u** in $CDCl_3$

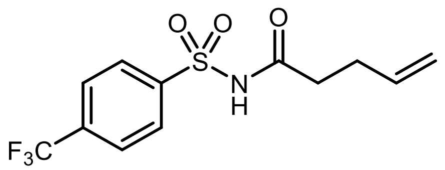

N-((4-(trifluoromethyl)phenyl)sulfonyl)pent-4-enamide (3): Prepared according to **General Procedure A** using commercially available 4-pentenoic acid. 247 mg, 40%. Colorless solid.

^{1}H NMR (700 MHz, $CDCl_3$) = δ 8.69 (br s, 1H), 8.21 (d, *J* = 8.2 Hz, 2H), 7.83 (d, *J* = 8.2 Hz, 2H), 5.71 (ddt, *J* = 16.9, 10.5, 6.5 Hz, 1H), 5.01 – 4.95 (m, 2H), 2.39 (t, *J* = 7.3 Hz, 2H), 2.32 (q, *J* = 6.9 Hz, 2H) ppm

^{13}C NMR (176 MHz, $CDCl_3$) = δ 170.3, 141.9, 135.8 (q, *J* = 33.2 Hz), 135.7, 129.2, 126.3 (q, *J* = 3.6 Hz), 123.2 (q, *J* = 273 Hz), 116.7, 35.7, 28.2 ppm

^{19}F NMR (376 MHz, $CDCl_3$) = δ -63.34 ppm

IR (neat) 3108, 2985, 1696, 1460, 1406, 1357, 1325, 1158, 1126, 1090 cm^{-1}

HRMS (ESI+) *m/z* calculated for $C_{12}H_{12}F_3NO_3S$ $[M+H]^+$: 308.0563, found 308.0563

R_f: (2:3 – EtOAc:Hex, 1 drop HOAc) = 0.54.

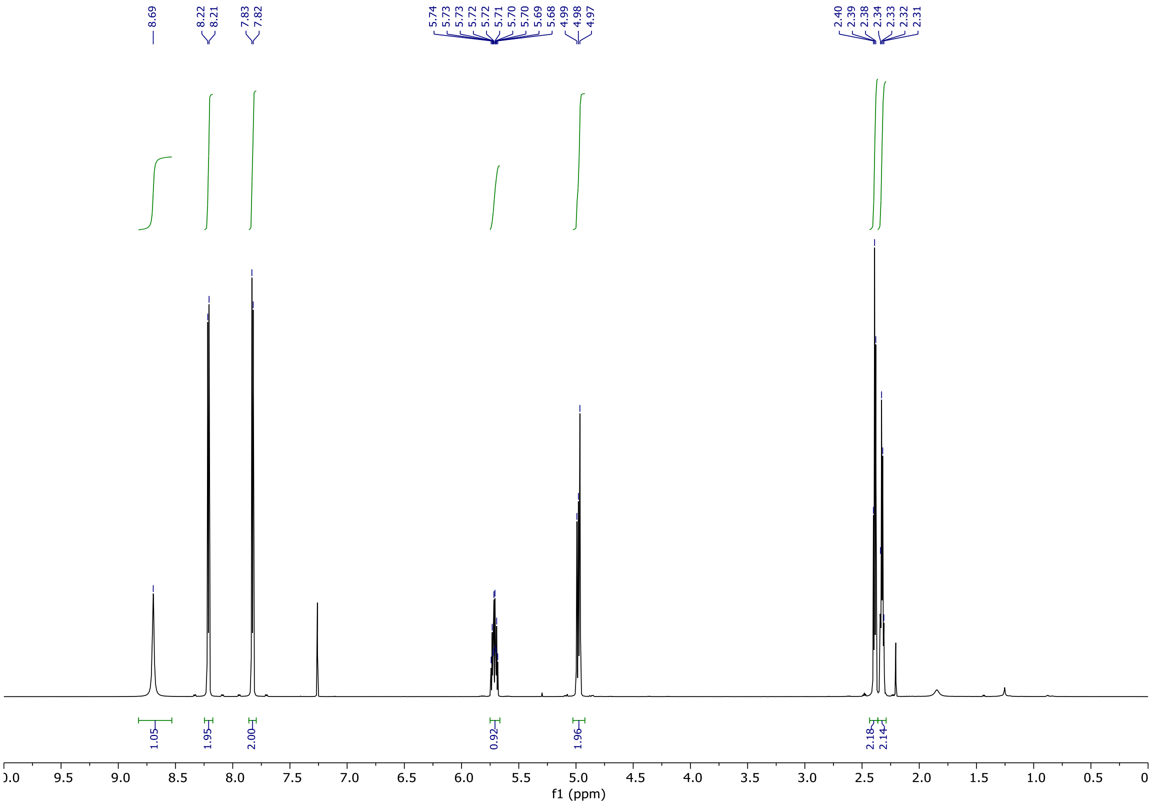

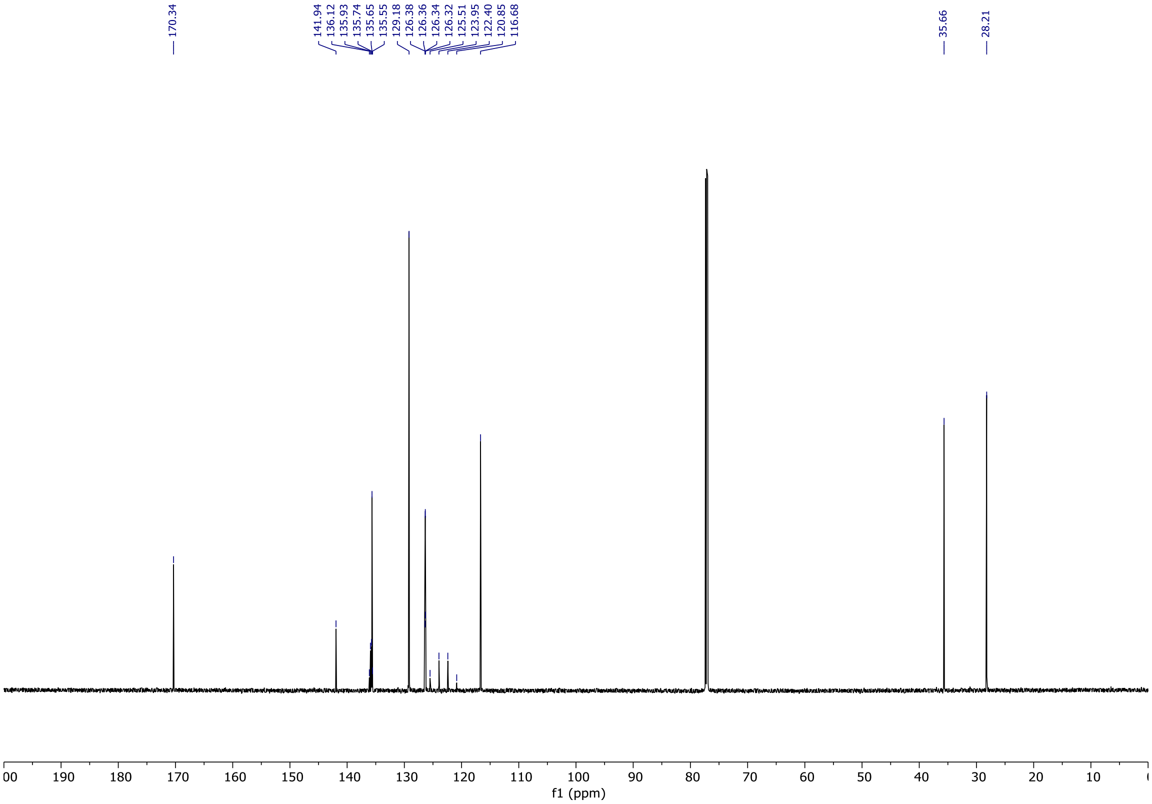

Supplementary Figure 32: ^{1}H (top) and ^{13}C NMR (bottom) for compound **3** in $CDCl_3$

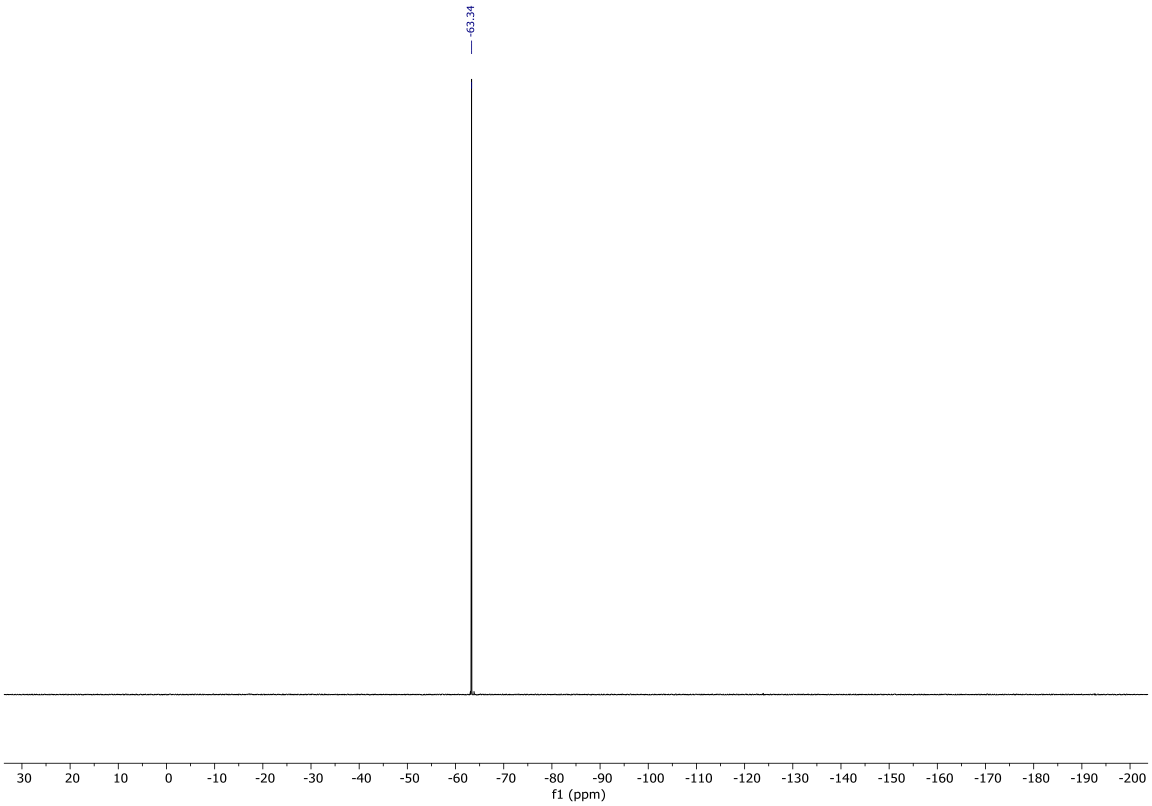

Supplementary Figure 33: ^{19}F NMR for compound **3** in $CDCl_3$

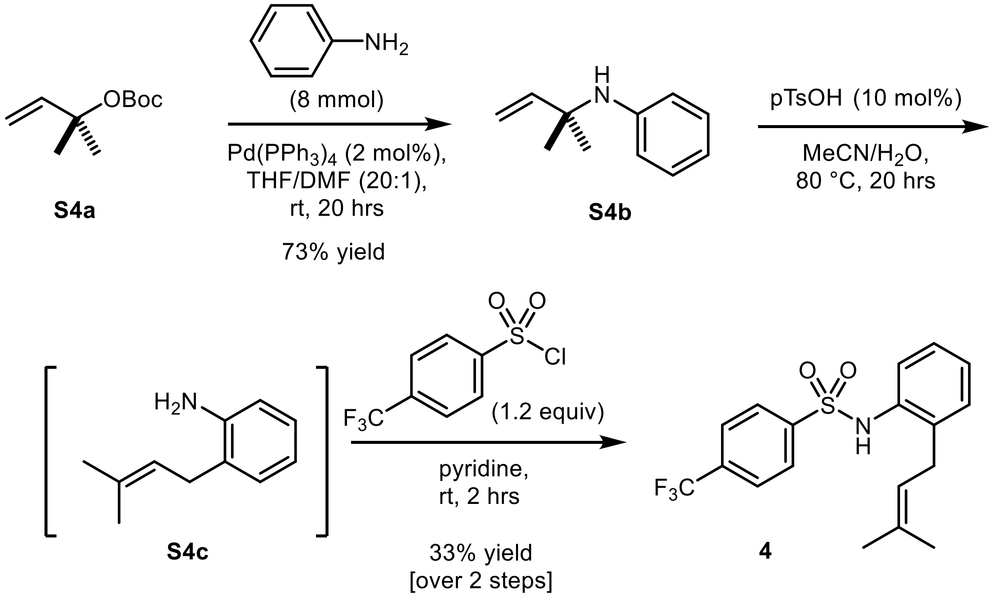

***N*-(2-(3-methylbut-2-en-1-yl)phenyl)-4-(trifluoromethyl)benzenesulfonamide (4)**: Procedure for the preparation of aniline **S4b** was adapted from a procedure previously reported.[13]

A solution of aniline (730 µL, 8.00 mmol) and tert-butyl-(2-methylbut-3-en-2-yl) carbonate[14] **S4a** (2.00 g, 10.8 mmol) in THF (20 mL) and DMF (1 mL) was treated with $Pd(PPh_3)_4$ (186 mg, 2 mol%) and stirred at room temperature for 20 h. The mixture was diluted with ethyl acetate (20 mL) and washed with brine (15 mL), dried over Na_2SO_4, filtered and concentrated *in vacuo*. The crude residue was purified by flash chromatography (95:5 Hex:EtOAc) to give aniline **S4b** as a light-yellow oil (947 mg, 73%). All spectra and characterization data matches that previously reported in the literature.[13,15]

Partial characterization of aniline **S4b** is provided below.

^{1}H NMR (500 MHz, $CDCl_3$) = δ 7.19 – 7.04 (m, 2H), 6.76 – 6.62 (m, 3H), 6.01 (dd, *J* = 17.5, 10.7 Hz, 1H), 5.26 – 5.01 (m, 2H), 3.70 (s, 1H), 1.39 (s, 7H) ppm

HRMS (ESI+) *m/z* calculated for $C_{11}H_{15}N$ [M+H]+ 162.1277, found 162.1275.

Procedure for the preparation of arylsulfonamide **4** was adapted from a procedure previously reported.[15]

Step 1: To a solution of aniline **S4b** (574 mg, 3.56 mmol) in MeCN:H_2O (10 mL:1.0 mL) under inert atmosphere was added *p*TsOH-H_2O (68 mg, 0.36 mmol, 0.1 equiv), and it was heated at 80 °C overnight. After cooling back to room temperature, it was washed with water (20 mL). The aqueous layer was extracted with EtOAc (20 mL x 2). The combined organic layers were washed with brine (30 mL), dried over Na_2SO_4, filtered, and concentrated. The crude product was directly used in next step without further purification.

Step 2: To a solution of the above crude material in pyridine (5 mL) at room temperature under inert atmosphere was added 4-(trifluoromethyl)benzenesulfonyl chloride (1045 mg, 4.27 mmol, 1.2 equiv). After one hour, EtOAc (20 mL) was added, and it was washed with 10% aq HCl (100 mL). The aqueous layer was extracted with EtOAc (20 mL x 2). The combined organic layers were washed with brine (30 mL), dried over Na_2SO_4, filtered, and concentrated. Flash column chromatography (Hex:EtOAc, 10:1) afforded 435 mg (33% over two steps) of compound **4** as a colorless solid.

^{1}H NMR (700 MHz, $CDCl_3$) = δ 7.83 (d, *J* = 8.2 Hz, 2H), 7.70 (d, *J* = 8.3 Hz, 2H), 7.44 (dd, *J* = 8.1, 1.3 Hz, 1H), 7.21 (td, *J* = 7.7, 1.7 Hz, 1H), 7.13 (td, *J* = 7.5, 1.3 Hz, 1H), 7.09 (dd, *J* = 7.6, 1.6 Hz, 1H), 6.63 (s, 1H), 4.95 (ddt, *J* = 7.0, 5.5, 1.5 Hz, 1H), 2.95 (d, *J* = 7.1 Hz, 2H), 1.74 (s, 3H), 1.69 (s, 3H) ppm

^{13}C NMR (176 MHz, $CDCl_3$) = δ 143.2, 135.1, 134.6 (q, *J* = 33.2 Hz), 134.3, 133.7, 130.2, 127.6, 127.5, 126.5, 126.1 (q, *J* = 3.7 Hz), 123.8, 123.1 (q, *J* = 272.9 Hz), 121.0, 31.1, 25.6, 17.8 ppm

^{19}F NMR (377 MHz, $CDCl_3$) = δ -63.17 ppm

IR (*neat*) = 3230, 1452, 1405, 1317, 1154, 1128, 1106, 1060, 907, 840, 755, 716, 674 cm^{-1}

HRMS (ESI+) *m/z* calculated for $C_{18}H_{18}F_3NO_2S$ $[M+H]^+$ 370.1083, found 370.1081.

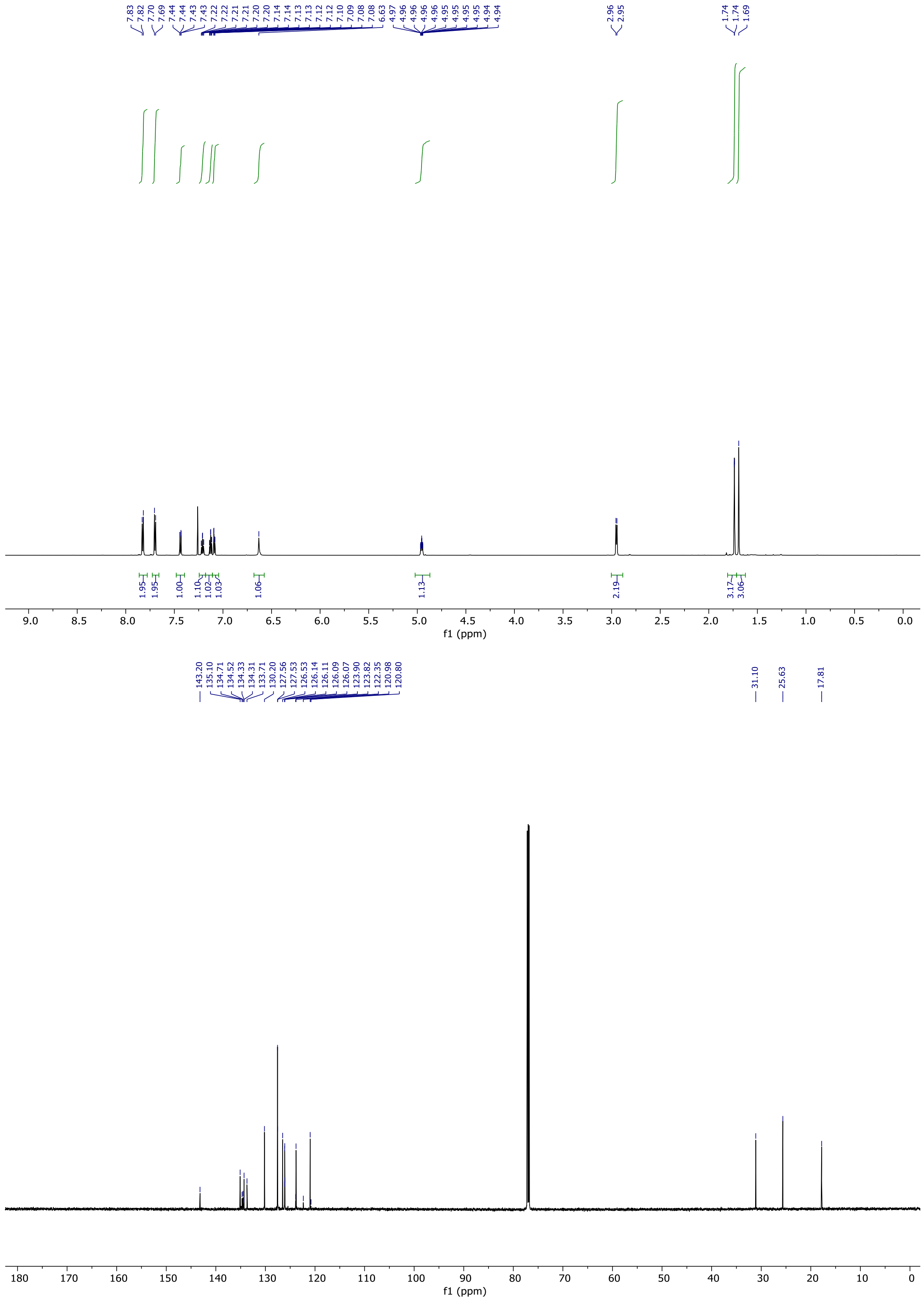

Supplementary Figure 34: ^{1}H (top) and ^{13}C NMR (bottom) for compound **4** in $CDCl_3$

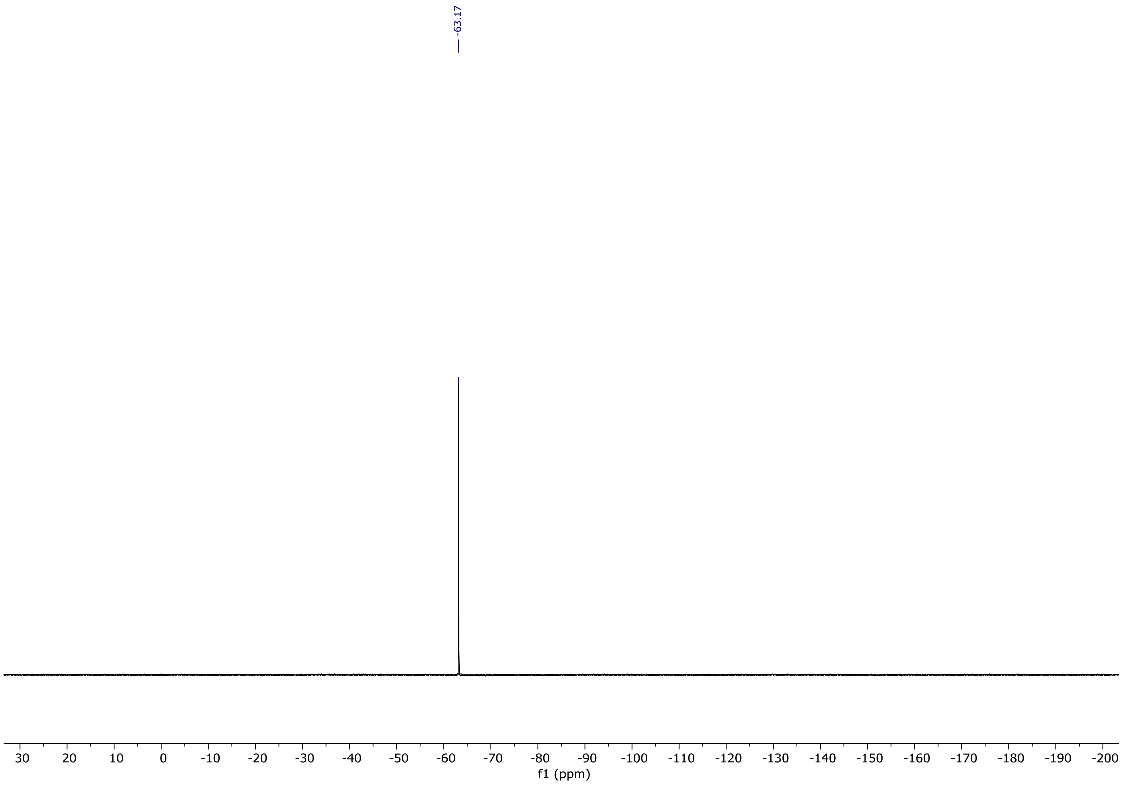

Supplementary Figure 35: ^{19}F NMR for compound **4** in $CDCl_3$

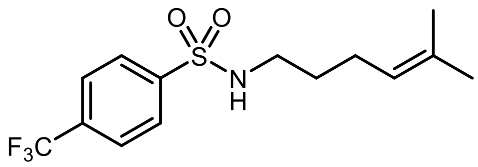

N-(5-methylhex-4-en-1-yl)-4-(trifluoromethyl)benzenesulfonamide (5): In a flame-dried flask with a stir bar, lithium aluminum hydride (24 mg, 0.6 mmol, 1.05 equiv) was suspended in 3 mL THF and cooled to -78 °C. A solution of 5-methyl-N-((4-(trifluoromethyl)phenyl)sulfonyl)hex-4-enamide (200 mg, 0.596 mmol, 1 equiv) in 3 mL THF was slowly added to the LAH solution down the side of the flask over ~2 minutes. The reaction was stirred at -78 °C for 3 hours, removed from the dry ice/acetone bath and allowed to warm to RT for 30 minutes. Quenched with 0.5 mL sat. aq. potassium sodium tartrate, added dropwise slowly at RT. The reaction was transferred to a separatory funnel with 50 mL EtOAc and 50 mL sat. aq. potassium sodium tartrate. The organic phase was isolated and the aqueous phase was washed with EtOAc (2 x 50 mL). The combined organic phases were washed with brine, dried over $MgSO_4$, filtered, and concentrated. The residue was purified with flash chromatography on silica gel (0 to 25% EtOAc in hexanes gradient) to give the product as a colorless solid (137 mg, 72%).

^{1}H NMR (700 MHz, $CDCl_3$) = δ 8.00 (d, *J* = 8.1 Hz, 2H), 7.79 (d, *J* = 8.2 Hz, 2H), 5.01 – 4.96 (m, 1H), 4.76 – 4.49 (m, 1H), 3.01 – 2.96 (m, 2H), 1.96 (q, *J* = 7.0 Hz, 2H), 1.65 (s, 3H), 1.55 – 1.48 (m, 5H) ppm

^{13}C NMR (176 MHz, $CDCl_3$) = δ 143.9, 134.5 (q, *J* = 33.1 Hz), 133.2, 127.7, 126.4 (q, *J* = 3.6 Hz), 123.4 (q, *J* = 273 Hz), 122.8, 43.2, 29.8, 25.8, 25.1, 17.8 ppm

^{19}F NMR (377 MHz, $CDCl_3$) = δ -63.15 ppm

IR (neat) 3256, 2913, 1431, 1404, 1318, 1294, 1196, 1153, 1122, 1060 cm^{-1}

HRMS (ESI+) *m/z* calculated for $C_{14}H_{18}F_3NO_2S$ $[M+H]^+$: 322.1083, found 322.1088.

R_f: (1:3 – EtOAc:Hex) = 0.75.

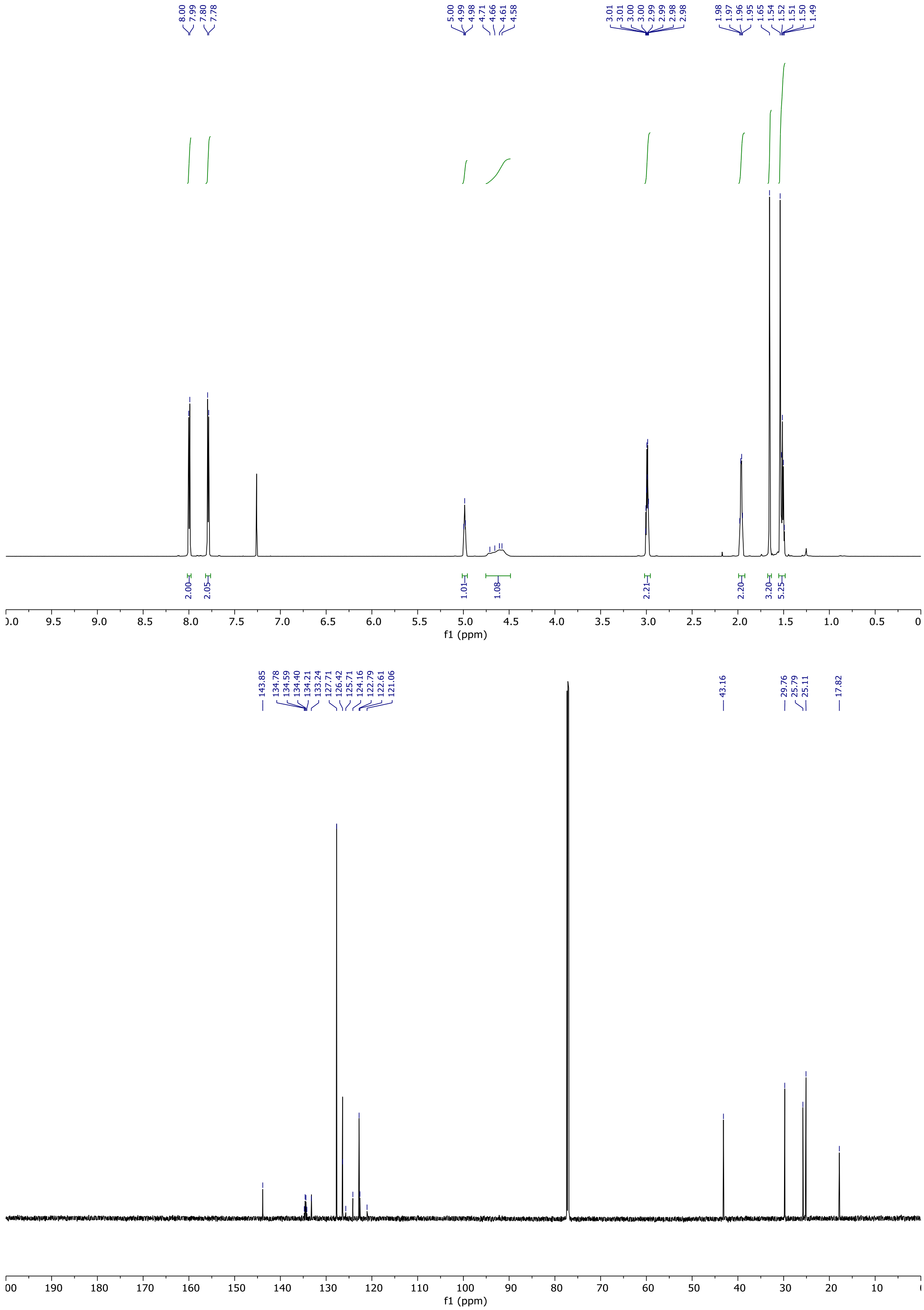

Supplementary Figure 36: ^{1}H (top) and ^{13}C NMR (bottom) for compound **5** in $CDCl_3$

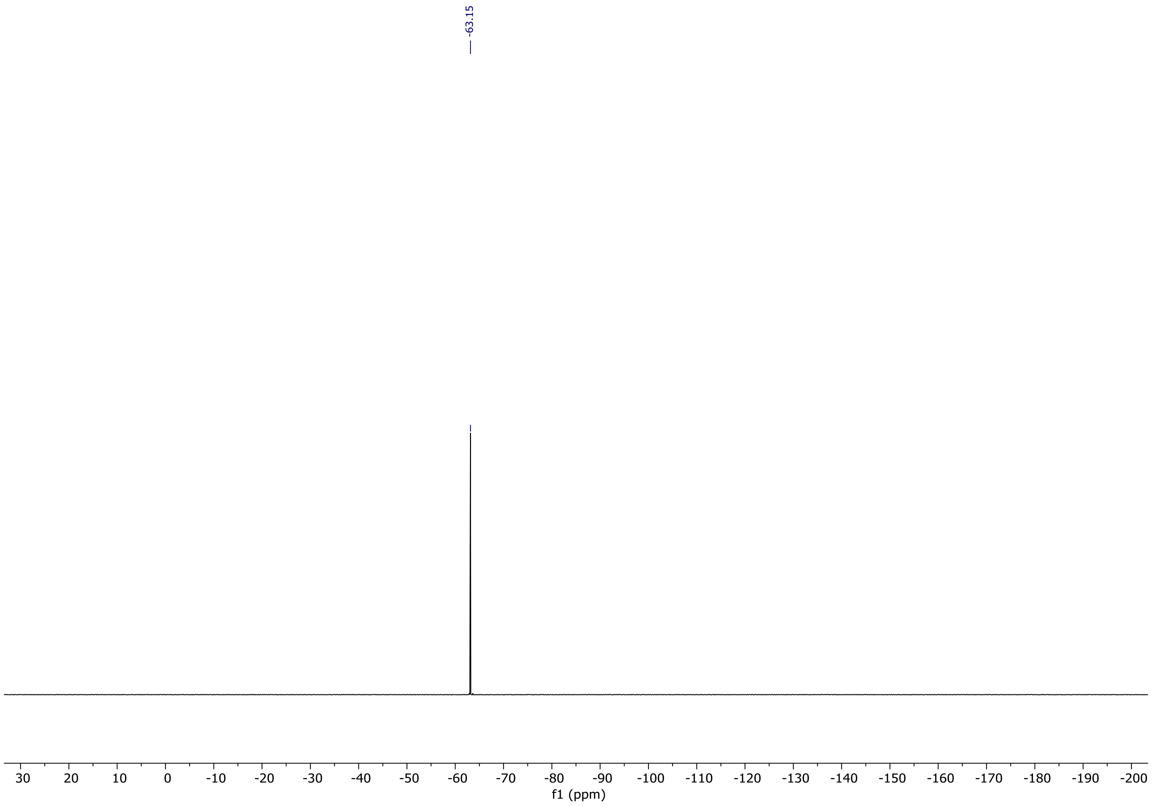

Supplementary Figure 37: ^{19}F NMR for compound **5** in $CDCl_3$

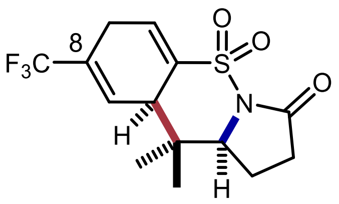

C8-trifluoromethyl cyclohexadiene-fused sultam (2a): Prepared according to **General Procedure C** with 67.1 mg, 0.2 mmol of **1a**. White powder (50.5 mg, 75% yield). R_f = 0.5 (1:1, Hex:EtOAc), one yellow spot, $KMnO_4$, UV.

^{1}H NMR (700 MHz, $CDCl_3$) = δ 7.02 (t, *J* = 3.2 Hz, 1H), 6.38 (s, 1H), 4.16 (dd, *J* = 8.4, 5.8 Hz, 1H), 3.49 (s, 1H), 3.19 – 2.99 (m, 2H), 2.58 – 2.42 (m, 2H), 2.20 (dddd, J = 13.3, 10.0, 8.5, 6.6 Hz, 1H), 1.92 (dddd, *J* = 13.1, 9.8, 7.1, 5.8 Hz, 1H), 1.14 (s, 3H), 0.79 (s, 3H) ppm

^{13}C NMR (176 MHz, $CDCl_3$) = δ 172.7, 134.3, 131.3, 127.9 (q, *J* = 31.4 Hz), 125.8 (q, *J* = 5.6 Hz), 122.8 (q, *J* = 272.3 Hz), 67.0, 45.3, 41.4, 30.9, 23.9, 22.0, 19.0, 13.8 ppm

^{19}F NMR (377 MHz, $CDCl_3$) = δ -69.85 ppm

IR (*neat*) = 2983, 1738, 1392, 1344, 1312, 1297, 1169, 1123, 986, 896, 706 cm^{-1}

HRMS (ESI+) *m/z* calculated for $C_{14}H_{16}F_3NO_3S$ $[M+H]^+$: 336.0876, found 336.0874.

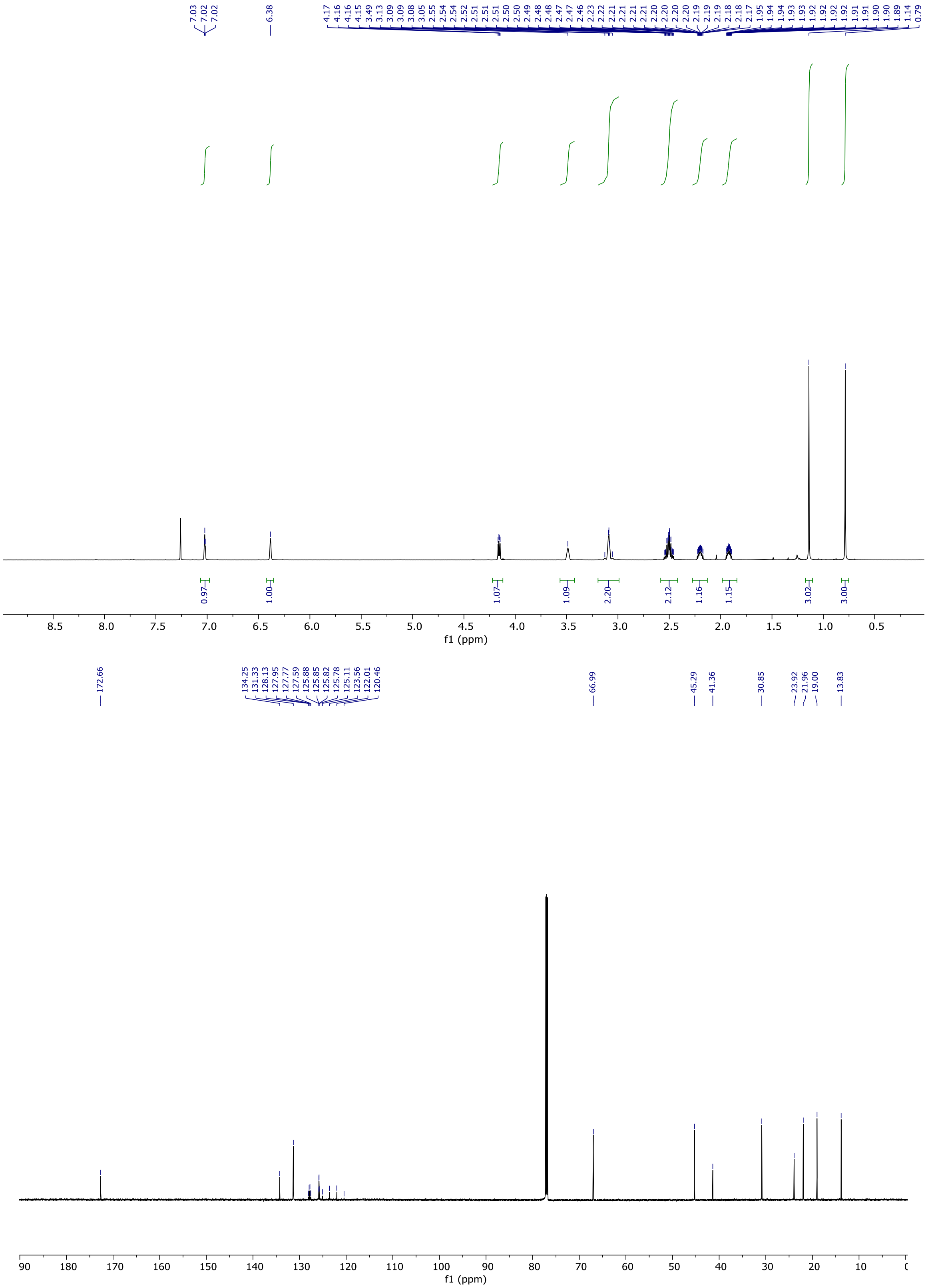

Supplementary Figure 38: ^{1}H (top) and ^{13}C NMR (bottom) for compound **2a** in $CDCl_3$

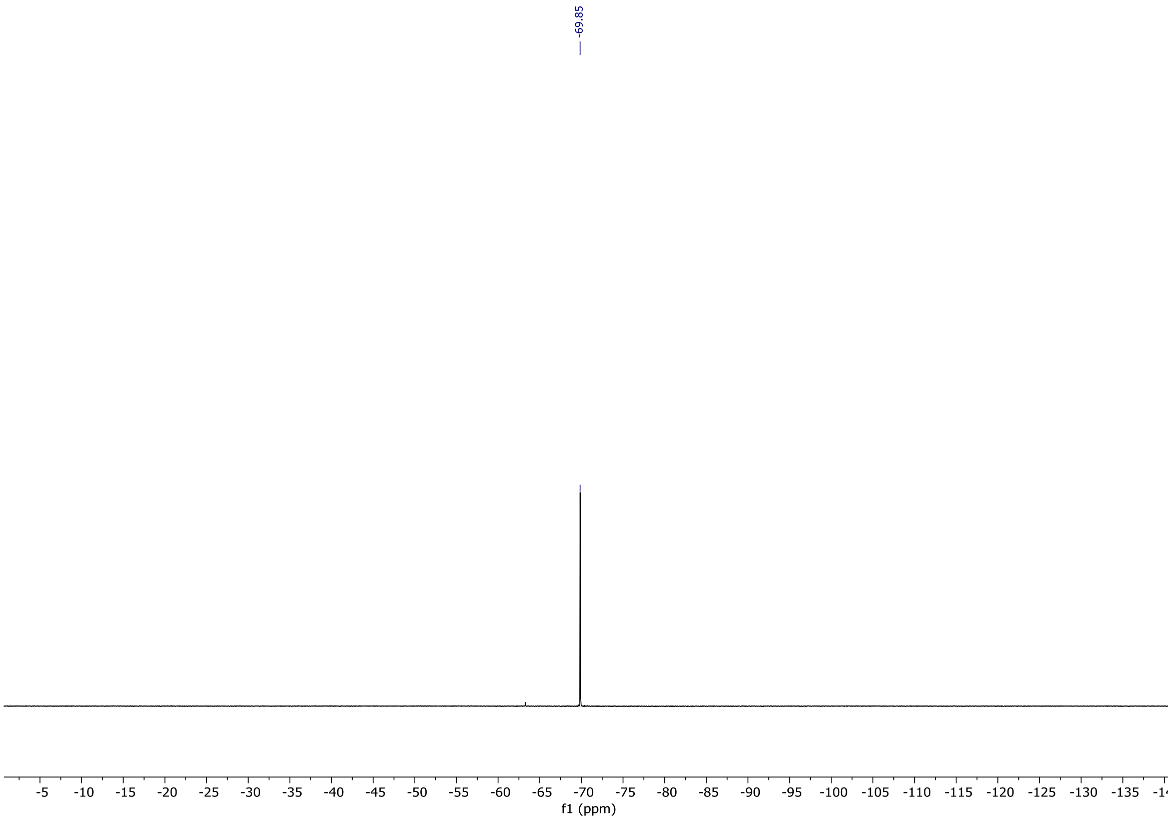

Supplementary Figure 39: ^{19}F NMR for compound **2a** in $CDCl_3$

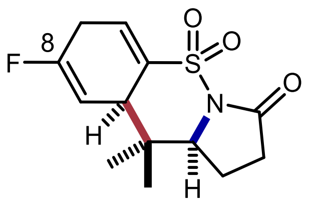

C8-fluoro cyclohexadiene-fused sultam (2b): Prepared according to **General Procedure C** with 57.1 mg, 0.2 mmol of **1b**. Off-white, tan powder (34.1 mg, 60% yield). R_f = 0.5 (1:1, Hex:EtOAc), one yellow spot, $KMnO_4$, UV.

^{1}H NMR (700 MHz, $CDCl_3$) = δ 6.95 – 6.85 (m, 1H), 5.33 (d, *J* = 14.8 Hz, 1H), 4.23 – 4.00 (m, 1H), 3.60 – 3.41 (m, 1H), 3.24 – 2.94 (m, 2H), 2.57 – 2.40 (m, 2H), 2.25 – 2.10 (m, 1H), 1.99 – 1.84 (m, 1H), 1.04 (s, 3H), 0.80 (s, 3H) ppm

^{13}C NMR (176 MHz, $CDCl_3$) = δ 172.8, 157.7 (d, *J* = 257.0 Hz), 135.2 (d, *J* = 2.5 Hz), 131.3 (d, *J* = 11.5 Hz), 99.2 (d, *J* = 18.1 Hz), 67.1, 46.5 (d, *J* = 7.5 Hz), 41.5 (d, *J* = 1.7 Hz), 31.0, 27.0 (d, *J* = 29.8 Hz), 22.0, 19.1, 13.4 ppm

^{19}F NMR (377 MHz, $CDCl_3$) = δ -102.48 (d, *J* = 17.0 Hz) ppm

IR (*neat*) = 2975, 1734, 1719, 1339, 1208, 1180, 1148, 1105, 1006, 954, 847 cm^{-1}

HRMS (ESI+) *m/z* calculated for $C_{13}H_{16}FNO_3S$ $[M+H]^+$: 286.0908, found 286.0910.

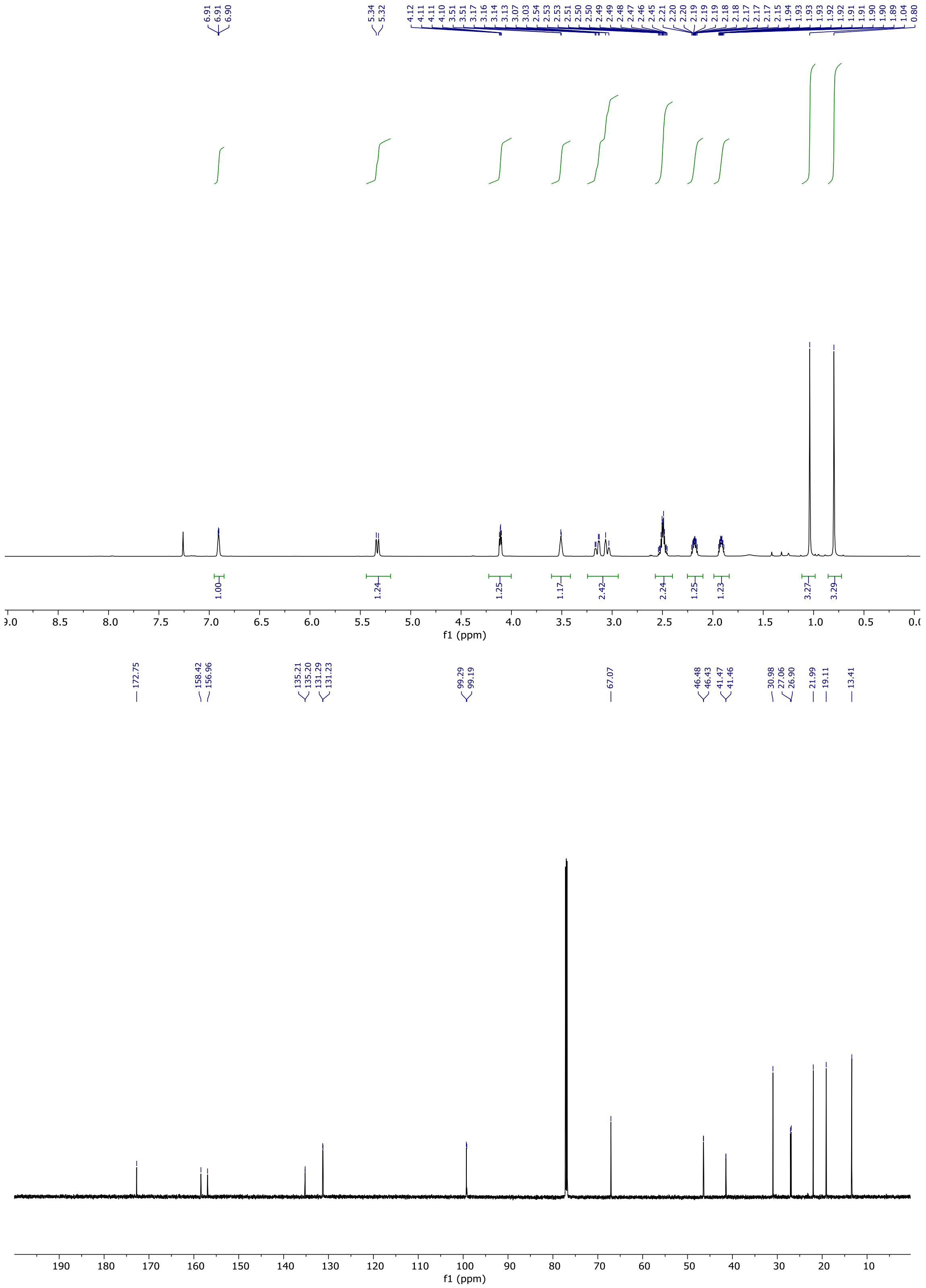

Supplementary Figure 40: ^{1}H (top) and ^{13}C NMR (bottom) for compound **2b** in $CDCl_3$

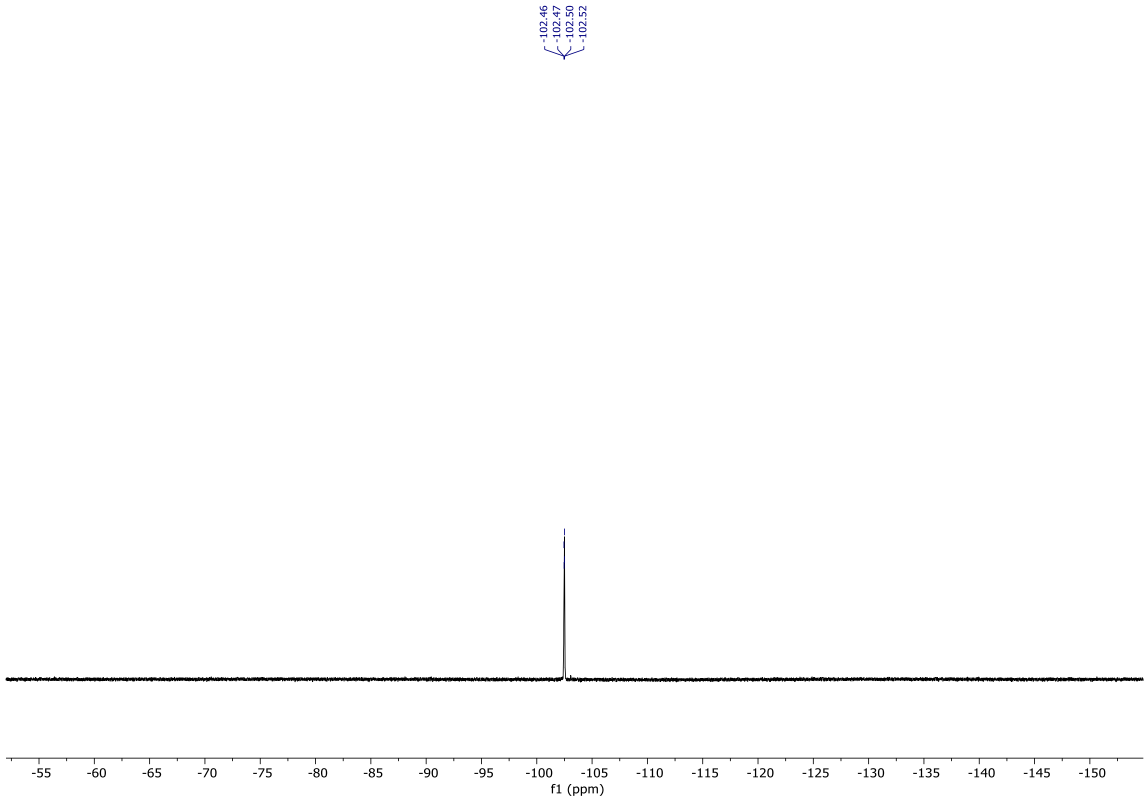

Supplementary Figure 41: ^{19}F NMR for compound **2b** in $CDCl_3$

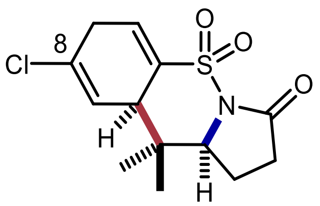

C8-chloro cyclohexadiene-fused sultam (2c): Prepared according to **General Procedure C** with 60.4 mg, 0.2 mmol of **1c**. Off-white, tan powder (31.5 mg, 52% yield). R_f = 0.3 (7:3, Hex:EtOAc), one yellow spot, $KMnO_4$, UV.

^{1}H NMR (500 MHz, $CDCl_3$) = δ 6.91 (t, *J* = 3.4 Hz, 1H), 5.97 – 5.83 (m, 1H), 4.11 (dd, *J* = 8.5, 5.7 Hz, 1H), 3.46 (q, *J* = 6.9 Hz, 1H), 3.34 – 3.05 (m, 2H), 2.59 – 2.42 (m, 2H), 2.30 – 2.07 (m, 1H), 2.01 – 1.81 (m, 1H), 1.09 (s, 3H), 0.81 (s, 3H) ppm

^{13}C NMR (176 MHz, $CDCl_3$) = δ 172.7, 134.4, 132.0, 130.9, 120.3, 66.9, 47.6, 41.6, 33.3, 30.9, 22.0, 19.1, 13.7 ppm

IR (*neat*) = 2971, 2935, 1736, 1676, 1339, 1205, 1175, 983, 953, 839, 643 cm^{-1}

HRMS (ESI+) *m/z* calculated for $C_{13}H_{16}ClNO_3S$ $[M+H]^+$: 302.0612, found 302.0611.

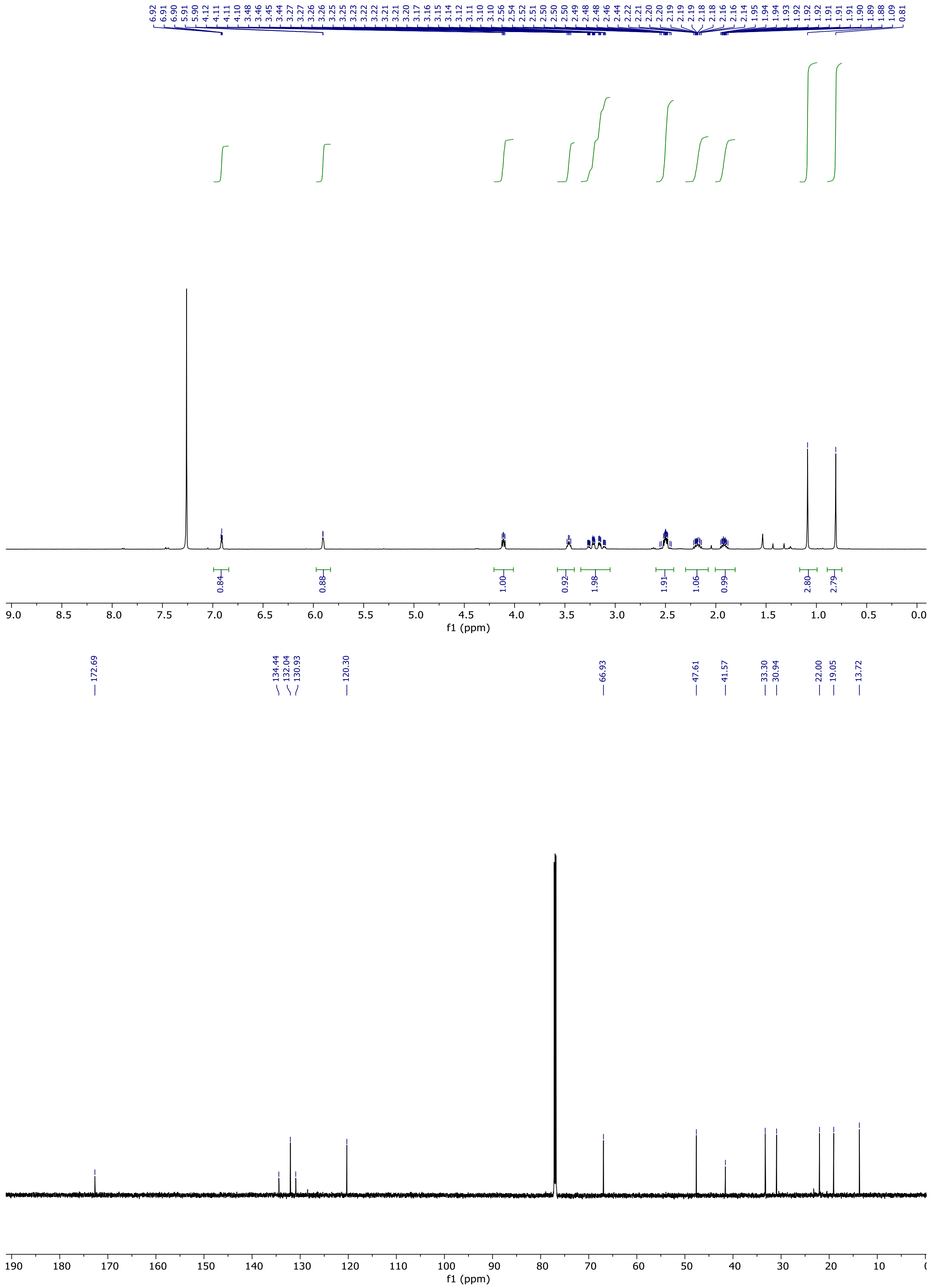

Supplementary Figure 42: ^{1}H (top) and ^{13}C NMR (bottom) for compound **2c** in $CDCl_3$

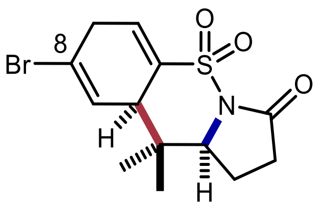

C8-bromo cyclohexadiene-fused sultam (2d): Prepared according to **General Procedure C** with 69.2 mg, 0.2 mmol of **1d**. Off-white, tan powder (45.2 mg, 65% yield). R_f = 0.5 (1:1, Hex:EtOAc), one yellow spot, $KMnO_4$, UV.

^{1}H NMR (400 MHz, $CDCl_3$) = δ 6.85 (t, J = 3.2 Hz, 1H), 6.13 (s, 1H), 4.10 (dd, J = 8.5, 5.7 Hz, 1H), 3.54 – 3.12 (m, 3H), 2.50 (ddd, J = 9.6, 7.4, 2.7 Hz, 2H), 2.18 (dq, J = 16.6, 8.4 Hz, 1H), 1.91 (dq, J = 14.9, 7.9 Hz, 1H), 1.09 (s, 3H), 0.82 (s, 3H) ppm

^{13}C NMR (176 MHz, $CDCl_3$) = δ 172.8, 134.2, 132.3, 124.4, 120.2, 66.8, 48.2, 41.4, 35.4, 30.9, 21.9, 19.0, 13.7 ppm

IR (*neat*) = 2969, 1737, 1471, 1344, 1169, 1124, 1103, 982, 954, 701, 655 cm^{-1}

HRMS (ESI+) *m/z* calculated for $C_{13}H_{16}BrNO_3S$ $[M+H]^+$: 346.0107, found 346.0105.

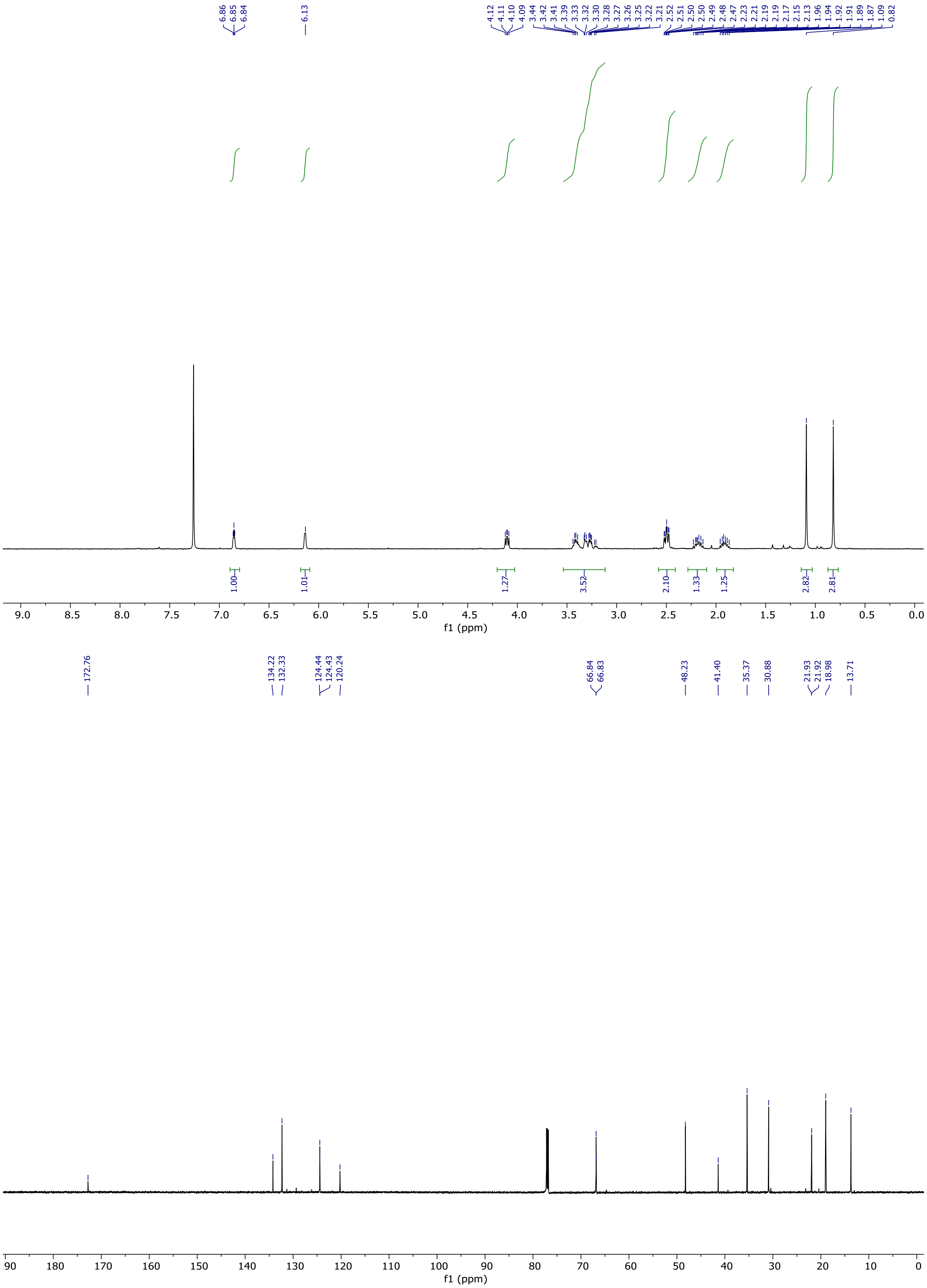

Supplementary Figure 43: ^{1}H (top) and ^{13}C NMR (bottom) for compound **2d** in $CDCl_3$

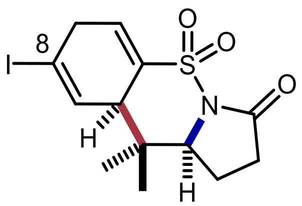

C8-iodo cyclohexadiene-fused sultam (2e): Prepared according to **General Procedure C** with 78.6 mg, 0.2 mmol of **1e**. Off-white, tan powder (52.2 mg, 66% yield). R_f = 0.4 (1:1, Hex:EtOAc), one yellow spot, $KMnO_4$, UV.

^{1}H NMR (700 MHz, $CDCl_3$) = δ 6.71 (q, *J* = 3.0 Hz, 1H), 6.41 (d, *J* = 2.8 Hz, 1H), 4.08 (dt, *J* = 8.5, 4.0 Hz, 1H), 3.46 – 3.19 (m, 3H), 2.48 (ddd, *J* = 15.8, 7.7, 4.8 Hz, 2H), 2.26 – 2.09 (m, 1H), 1.90 (ddt, *J* = 13.8, 10.7, 6.6 Hz, 1H), 1.08 (s, 3H), 0.81 (s, 3H) ppm

^{13}C NMR (176 MHz, $CDCl_3$) = δ 172.8, 134.0, 132.9, 132.7, 93.4, 66.8, 48.9, 41.4, 39.3, 30.9, 21.9, 19.0, 13.8 ppm

IR (*neat*) = 2970, 2928, 1729, 1330, 1219, 1163, 1134, 951, 822, 716 cm^{-1}

HRMS (ESI+) *m/z* calculated for $C_{13}H_{16}INO_3S$ $[M+H]^+$: 393.9968, found 393.9975.

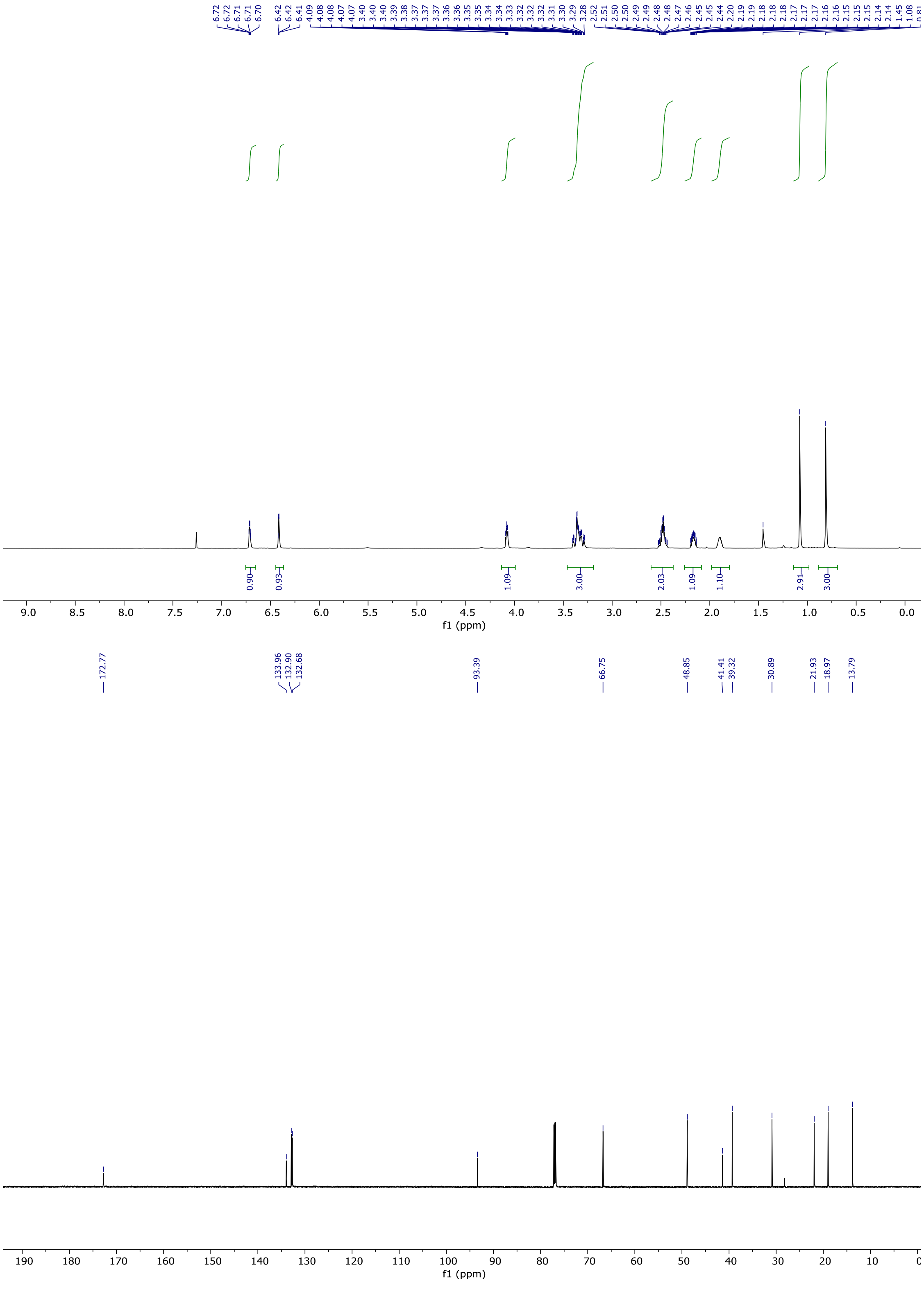

Supplementary Figure 44: ^{1}H (top) and ^{13}C NMR (bottom) for compound **2e** in $CDCl_3$

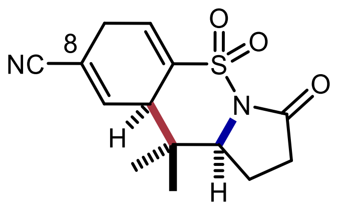

C8-cyano cyclohexadiene-fused sultam (2f): Prepared according to **General Procedure C** with 58.5 mg, 0.2 mmol of **1f**. Off-white, tan powder (22.7 mg, 39% yield). R_f = 0.5 (1:9, Acetone:CH_2Cl_2), one yellow spot, $KMnO_4$, UV.

^{1}H NMR (700 MHz, $CDCl_3$) = δ 6.94 (t, *J* = 3.0 Hz, 1H), 6.62 (s, 1H), 4.13 (dd, *J* = 8.6, 5.6 Hz, 1H), 3.49 (td, *J* = 7.5, 4.1 Hz, 1H), 3.27 – 3.04 (m, 2H), 2.58 – 2.39 (m, 2H), 2.19 (dddd, *J* = 19.9, 15.0, 10.7, 6.4 Hz, 1H), 1.96 – 1.83 (m, 1H), 1.13 (s, 3H), 0.80 (s, 3H) ppm

^{13}C NMR (176 MHz, $CDCl_3$) = δ 172.5, 139.3, 133.9, 130.5, 117.3, 112.0, 66.8, 46.0, 41.7, 30.8, 27.7, 22.0, 19.0, 14.2 ppm

IR (*neat*) = 2967, 2938, 2220, 1739, 1338, 1203, 1178, 1138, 1090, 996, 955, 845 cm^{-1}

HRMS (ESI+) *m/z* calculated for $C_{14}H_{16}N_2O_3S$ $[M+H]^+$: 293.0954, found 293.0961.

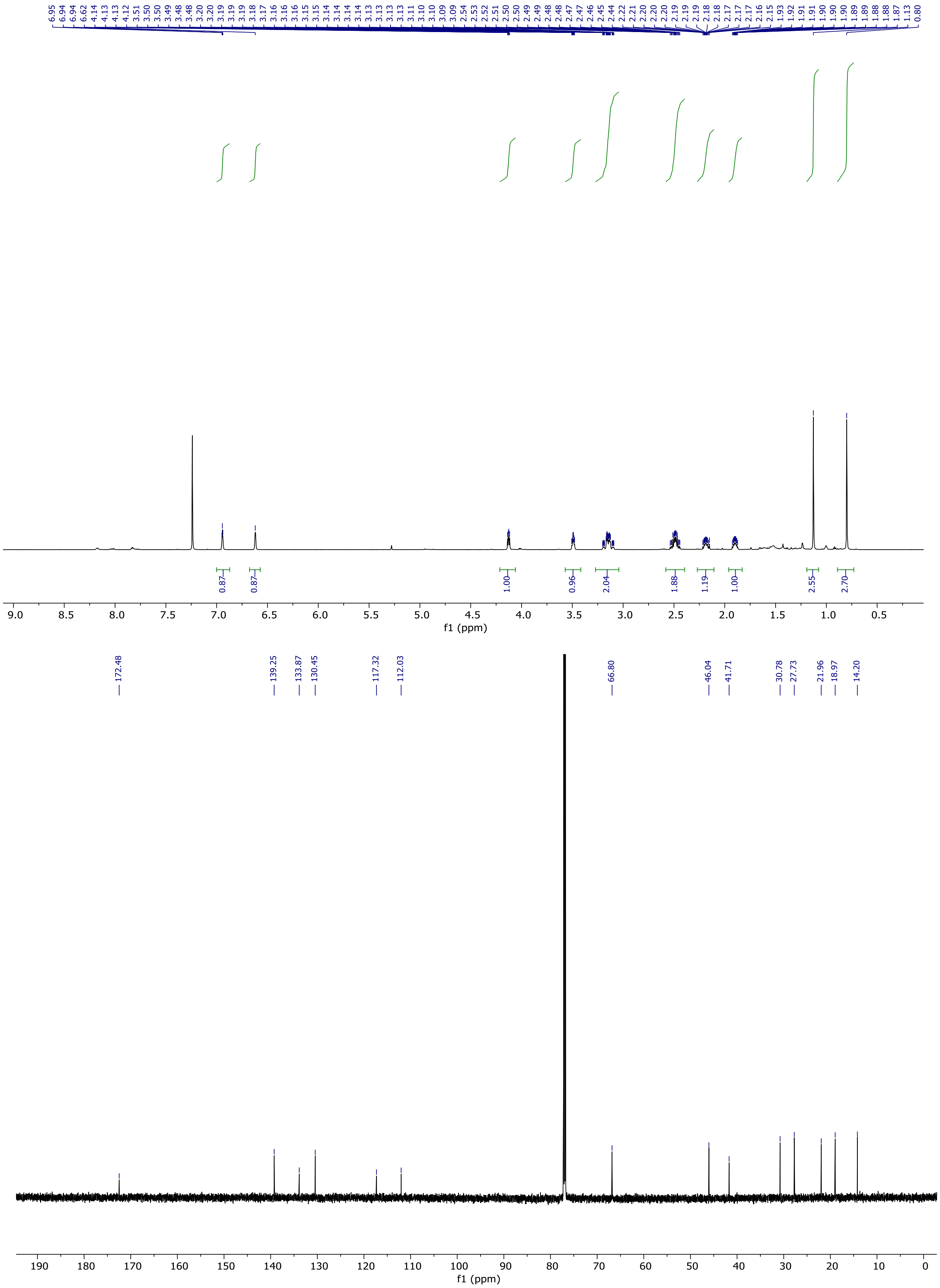

Supplementary Figure 45: ^{1}H (top) and ^{13}C NMR (bottom) for compound **2f** in $CDCl_3$

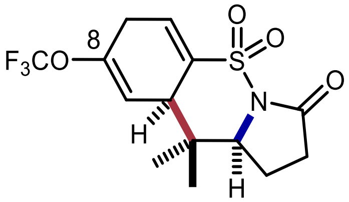

C8-trifluoromethoxy cyclohexadiene-fused sultam (2g): Prepared according to **General Procedure C** with 70.3 mg, 0.2 mmol of **1g**. Off-white, tan powder (56.5 mg, 80% yield). R_f = 0.5 (1:1, Hex:EtOAc), one yellow spot, $KMnO_4$, UV.

^{1}H NMR (700 MHz, $CDCl_3$) = δ 6.91 (t, *J* = 3.2 Hz, 1H), 5.64 (s, 1H), 4.13 (dd, *J* = 8.5, 5.8 Hz, 1H), 3.54 (q, *J* = 7.0 Hz, 1H), 3.21 – 2.99 (m, 2H), 2.60 – 2.41 (m, 2H), 2.19 (dddd, *J* = 13.5, 10.1, 8.5, 6.7 Hz, 1H), 1.92 (dddd, *J* = 13.1, 9.8, 7.2, 5.8 Hz, 1H), 1.07 (s, 3H), 0.80 (s, 3H) ppm

^{13}C NMR (176 MHz, $CDCl_3$) = δ 172.7, 145.3, 134.6, 131.2, 120.2 (q, *J* = 258.5 Hz), 109.9, 67.0, 46.5, 41.6, 30.9, 28.3, 22.0, 19.1, 13.5 ppm

^{19}F NMR (377 MHz, $CDCl_3$) = δ -57.44 ppm

IR (*neat*) = 2980, 1739, 1701, 1341, 1256, 1175, 1132, 1097, 1009, 955, 828, 673 cm^{-1}

HRMS (ESI+) *m/z* calculated for $C_{14}H_{16}F_3NO_4S$ $[M+H]^+$: 352.0825, found 352.0837.

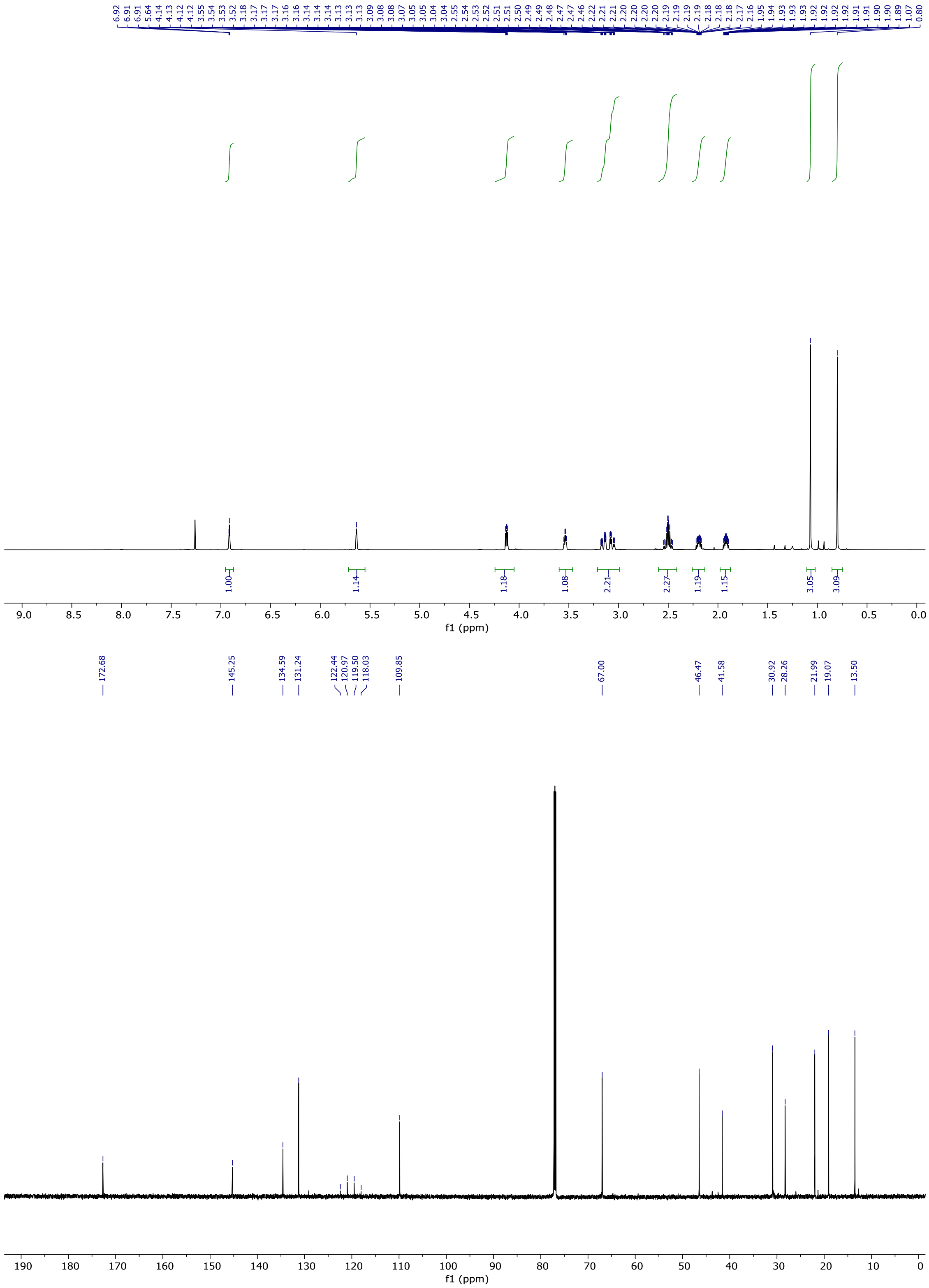

Supplementary Figure 46: ^{1}H (top) and ^{13}C NMR (bottom) for compound **2g** in $CDCl_3$

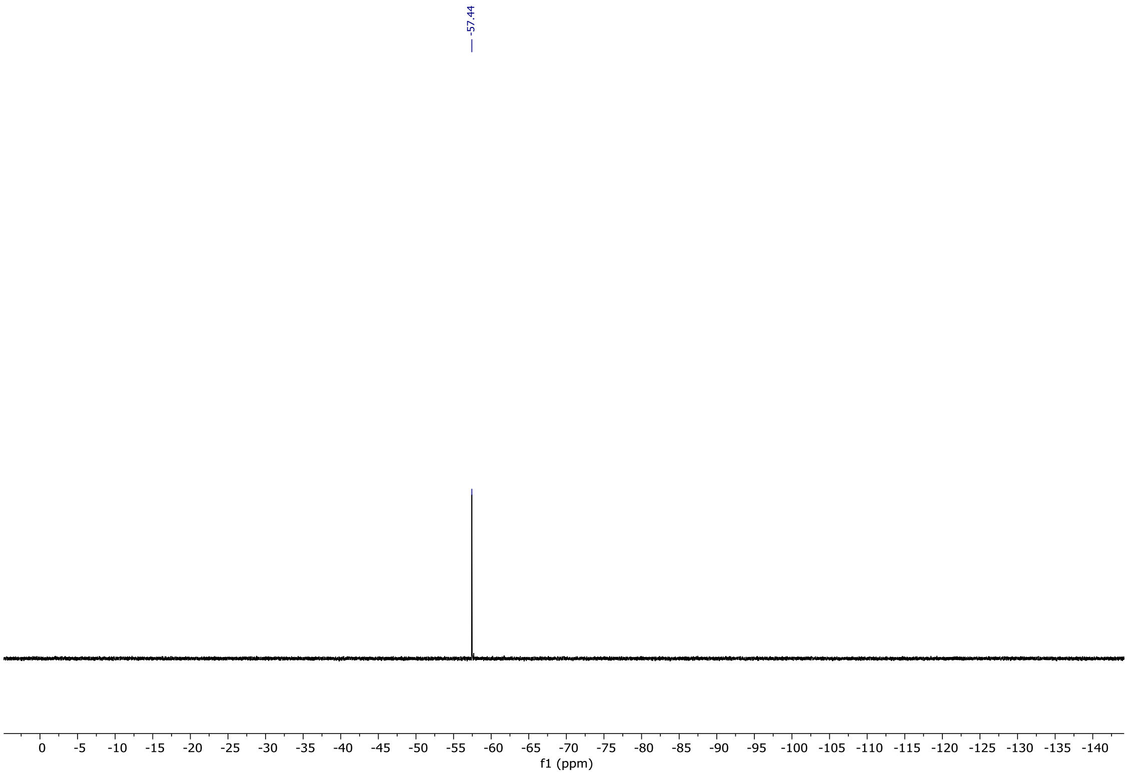

Supplementary Figure 47: ^{19}F NMR for compound **2g** in $CDCl_3$

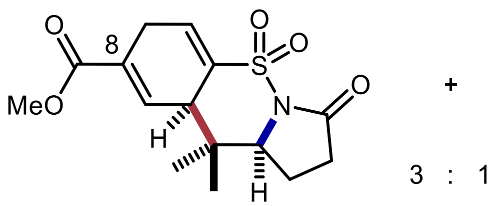

C8-methyl carboxylate cyclohexadiene-fused sultam (2h): Prepared according to **General Procedure C** with 65.1 mg, 0.2 mmol of **1h**. White powder (20.2 mg, 31% yield isolated as an inseparable 3:1 mixture of diene:arene products). R_f = 0.3 (1:1, Hex:EtOAc), one yellow spot, $KMnO_4$, UV.

diene ^{1}**H NMR** (700 MHz, $CDCl_3$) = δ 7.03 (s, 1H), 6.97 (s, 1H), 4.16 (dd, *J* = 8.5, 5.7 Hz, 1H), 3.80 (s, 3H), 3.52 (td, *J* = 7.4, 4.0 Hz, 1H), 3.26 – 3.11 (m, 2H), 2.56 – 2.41 (m, 2H), 2.25 – 2.12 (m, 2H), 1.97 – 1.86 (m, 1H), 1.16 (s, 3H), 0.78 (s, 3H) ppm

arene ^{1}**H NMR** (700 MHz, $CDCl_3$) = δ 8.15 (d, *J* = 1.6 Hz, 1H), 8.09 (dd, *J* = 8.3, 1.6 Hz, 1H), 8.01 (d, *J* = 8.3 Hz, 1H), 4.40 (dd, *J* = 8.2, 6.1 Hz, 1H), 3.97 (s, 3H), 2.63 (dd, *J* = 9.3, 7.5 Hz, 2H), 2.37 (dq, *J* = 12.7, 7.9 Hz, 1H), 2.26 – 2.10 (m, 1H), 1.49 (s, 3H), 1.33 (s, 3H) ppm

mixture of diene and arene ^{13}**C NMR** (176 MHz, $CDCl_3$) = δ 173.3, 172.7, 165.8, 165.3, 144.1, 139.8, 134.4, 133.6, 133.6, 133.2, 129.2, 128.8, 127.7, 125.0, 67.1, 64.7, 52.8, 52.2, 46.3, 41.4, 39.4, 30.9, 30.5, 26.1, 23.3, 22.0, 20.4, 19.0, 14.1 ppm

mixture of diene and arene **IR** (*neat*) = 2971, 2948, 2246, 1737, 1700, 1441, 1341, 1263, 1203, 1175, 1088, 922, 724 cm^{-1}

diene **HRMS** (ESI+) *m/z* calculated for $C_{15}H_{19}NO_5S$ $[M+H]^+$: 326.1057, found 326.1055.

arene **HRMS** (ESI+) *m/z* calculated for $C_{15}H_{17}NO_5S$ $[M+H]^+$: 324.0900, found 324.0909.

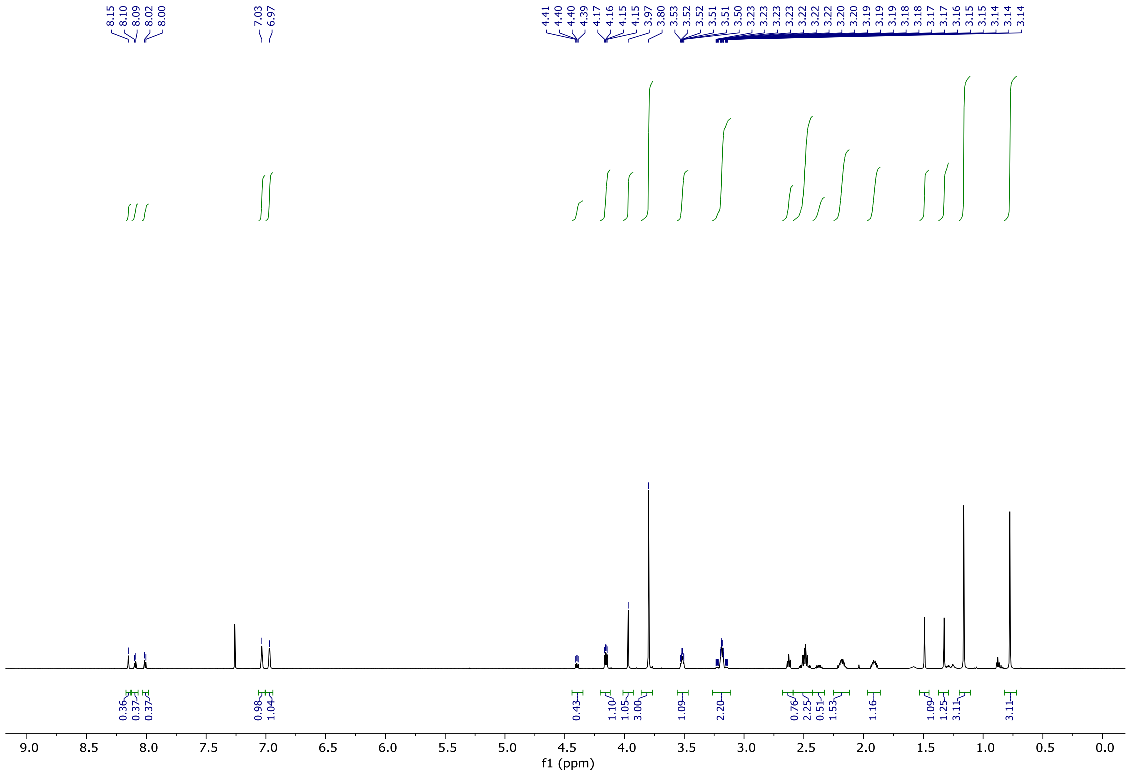

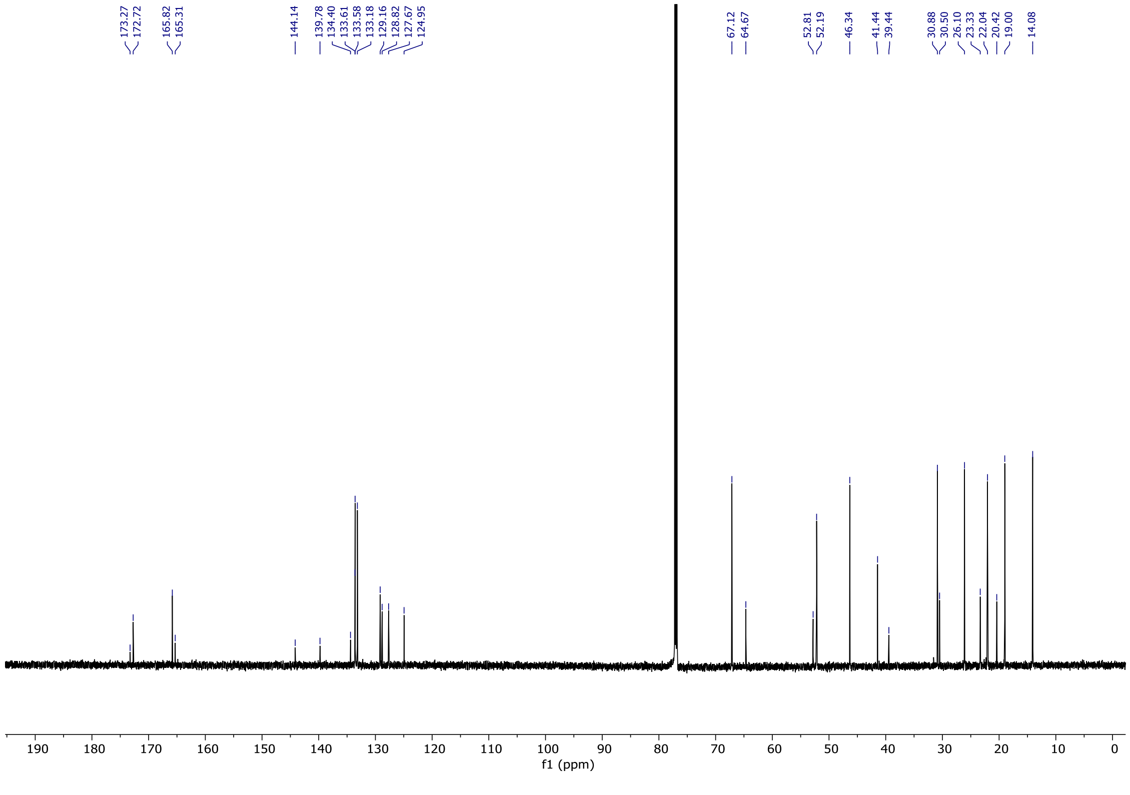

Supplementary Figure 48: ^{1}H (top) and ^{13}C NMR (bottom) for compound **2h** (mixture) in $CDCl_3$

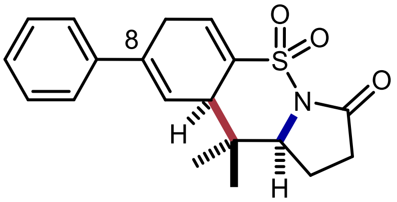

C8-phenyl cyclohexadiene-fused sultam (2i): Prepared according to **General Procedure C** with 68.7 mg, 0.2 mmol of **1i**. Clear, colorless oil which solidifies to a colorless solid over time (22.8 mg, 33% yield). R_f = 0.3 (1:1, Hex:EtOAc), one yellow spot, $KMnO_4$, UV.

^{1}H NMR (700 MHz, $CDCl_3$) = δ 7.42 – 7.35 (m, 4H), 7.35 – 7.31 (m, 1H), 7.13 – 7.09 (m, 1H), 6.15 – 6.07 (m, 1H), 4.17 (dd, *J* = 8.4, 5.9 Hz, 1H), 3.52 (td, *J* = 7.1, 4.1 Hz, 1H), 3.42 – 3.22 (m, 2H), 2.56 – 2.45 (m, 2H), 2.19 (dddd, *J* = 13.4, 9.8, 8.5, 6.7 Hz, 1H), 1.93 (dddd, *J* = 13.3, 9.7, 7.5, 6.0 Hz, 1H), 1.16 (s, 3H), 0.82 (s, 3H) ppm

^{13}C NMR (176 MHz, $CDCl_3$) = δ 173.0, 139.6, 136.0, 134.4, 133.5, 128.6, 128.2, 125.3, 119.5, 67.3, 46.4, 41.8, 31.0, 28.8, 22.1, 19.1, 13.7 ppm

IR (*neat*) = 2974, 2919, 2234, 1734, 1714, 1340, 1218, 1163, 1090, 956, 772 cm^{-1}

HRMS (ESI+) *m/z* calculated for $C_{19}H_{21}NO_3S$ $[M+H]^+$: 344.1315, found 344.1310.

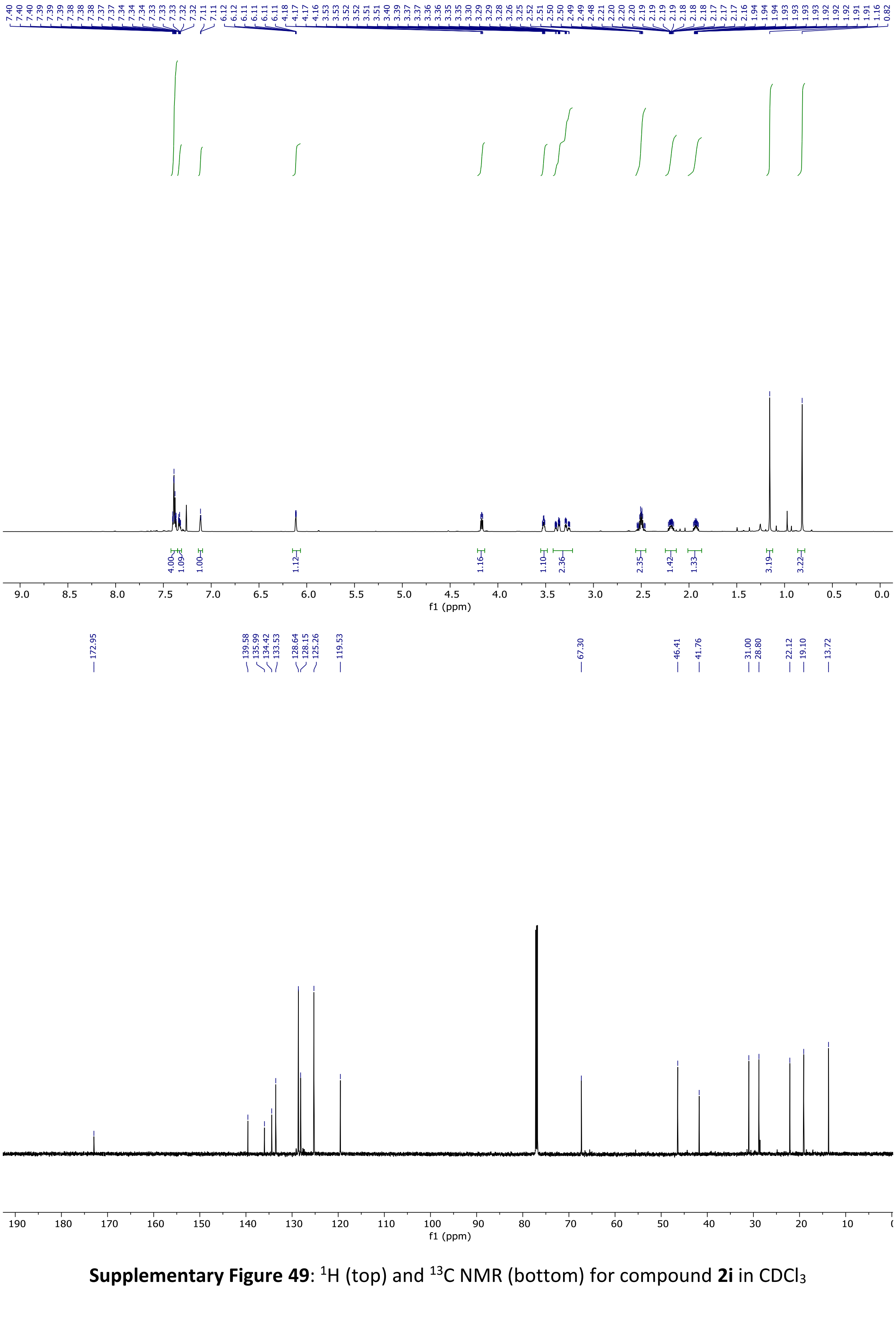

Supplementary Figure 49: ^{1}H (top) and ^{13}C NMR (bottom) for compound **2i** in $CDCl_3$

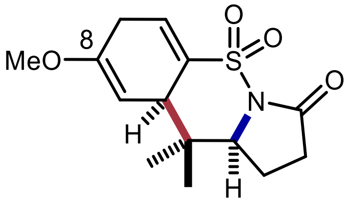

C8-methoxy cyclohexadiene-fused sultam (2j): Prepared according to a modification of **General Procedure C** with 89.2 mg, 0.3 mmol of **1j**, $NBu_4OP(O)(OBu)_2$ (58 mg, 0.13 mmol, 0.43 equiv), and photocatalyst **A** (3 mg, 1 mol%). in 1.5 mL of $PhCF_3$ (0.2 M). Off-white, tan powder (10.4 mg, 12% yield). R_f = 0.3 (1:1, Hex:EtOAc), one yellow spot (multiple spots are observed on TLC following decomposition), $KMnO_4$, UV.

The title diene product is unstable and readily undergoes decomposition. Partial characterization is provided below.

^{1}H NMR (500 MHz, $CDCl_3$) = δ 6.92 (t, *J* = 3.2 Hz, 1H), 4.65 (d, *J* = 3.1 Hz, 1H), 4.10 (dd, *J* = 8.4, 5.9 Hz, 1H), 3.61 (s, 3H), 3.46 (q, *J* = 5.9 Hz, 1H), 3.09 – 2.81 (m, 2H), 2.49 (ddd, *J* = 9.2, 7.5, 1.8 Hz, 2H), 2.28 – 2.09 (m, 1H), 2.00 – 1.83 (m, 1H), 1.06 (s, 3H), 0.75 (s, 3H) ppm

IR (*neat*) = 2941, 1736, 1688, 1580, 1340, 1186, 1163, 1023, 977, 731, 649 cm^{-1}

HRMS (ESI+) *m/z* calculated for $C_{14}H_{19}NO_4S$ $[M+H]^+$: 298.1108, found 298.1112.

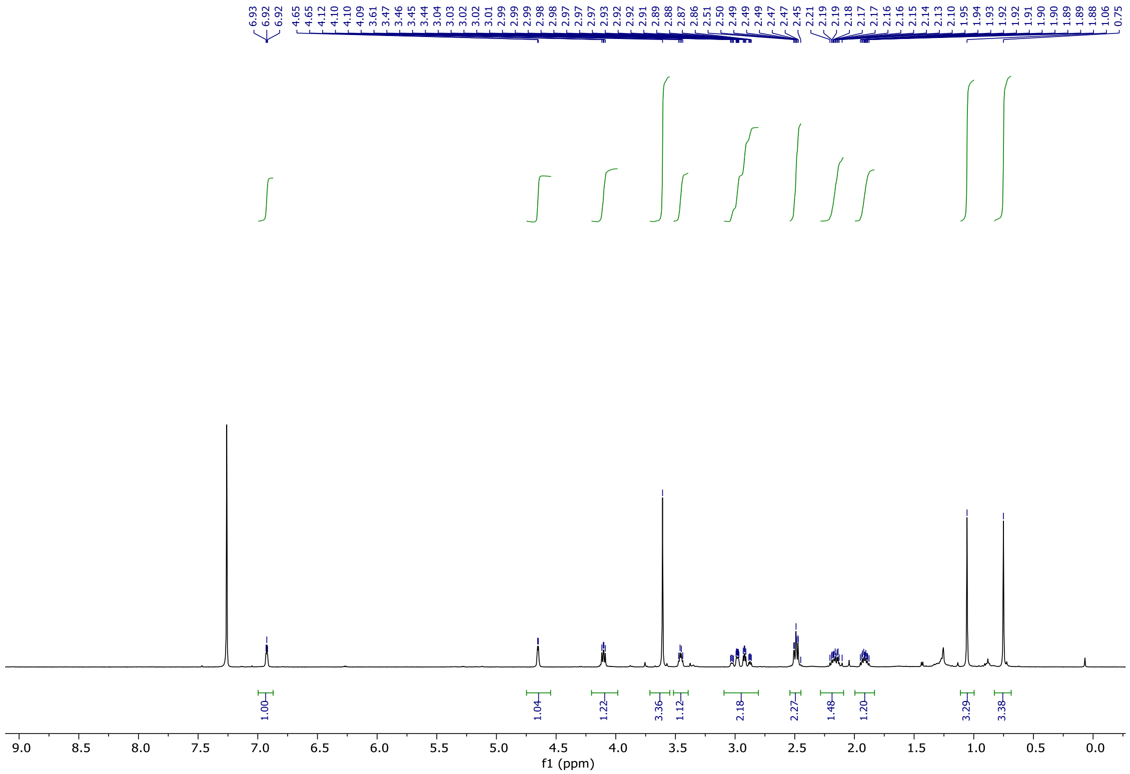

Supplementary Figure 50: ^{1}H NMR for compound **2j** in $CDCl_3$

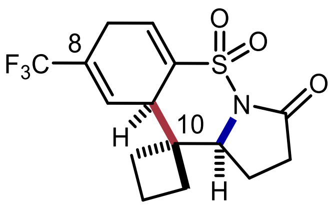

C8-trifluoromethyl C10-cyclobutyl cyclohexadiene-fused sultam (2k): Prepared according to **General Procedure C** with 69.5 mg, 0.2 mmol of **1k**. Off-white, tan powder (14.8 mg, 21% yield). R_f = 0.3 (1:1, Hex:EtOAc), one yellow spot, $KMnO_4$, UV.

^{1}H NMR (700 MHz, $CDCl_3$) = δ 7.02 (s, 1H), 6.73 – 6.62 (m, 1H), 4.17 (t, *J* = 7.5 Hz, 1H), 3.55 (q, *J* = 7.8, 6.2 Hz, 1H), 3.24 – 3.04 (m, 2H), 2.59 (td, *J* = 9.0, 7.8, 3.2 Hz, 2H), 2.37 (dtd, *J* = 14.7, 8.7, 6.4 Hz, 1H), 2.25 (dq, *J* = 15.9, 8.3 Hz, 1H), 2.09 (dq, *J* = 12.8, 5.3 Hz, 1H), 2.05 – 1.95 (m, 1H), 1.92 – 1.72 (m, 3H), 1.62 (q, *J* = 8.5, 7.5 Hz, 1H) ppm

^{13}C NMR (176 MHz, $CDCl_3$) = δ 172.8, 133.9, 131.6, 128.2 (q, *J* = 31.2 Hz), 126.4 (q, *J* = 5.6 Hz), 122.9 (q, *J* = 272.3 Hz), 64.7, 47.1, 42.7, 30.8, 24.1, 23.3, 19.9, 19.7, 14.1 ppm

^{19}F NMR (377 MHz, $CDCl_3$) = δ -69.80 ppm

IR (*neat*) = 2943, 1730, 1653, 1341, 1299, 1205, 1159, 1108, 1101, 958, 696 cm^{-1}

HRMS (ESI+) *m/z* calculated for $C_{15}H_{16}F_3NO_3S$ $[M+H]^+$: 348.0876, found 348.0884.

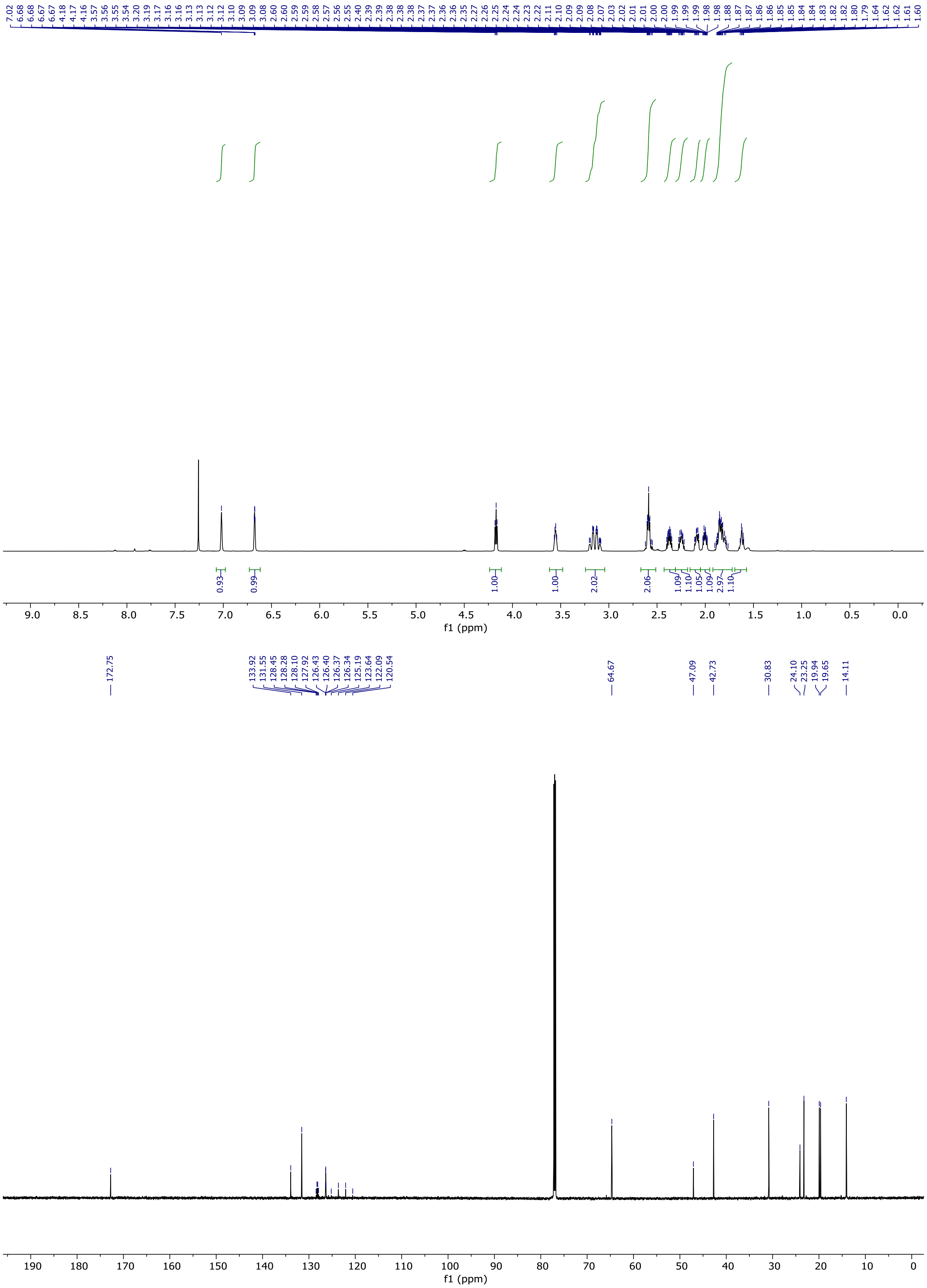

Supplementary Figure 51: ^{1}H (top) and ^{13}C NMR (bottom) for compound **2k** in $CDCl_3$

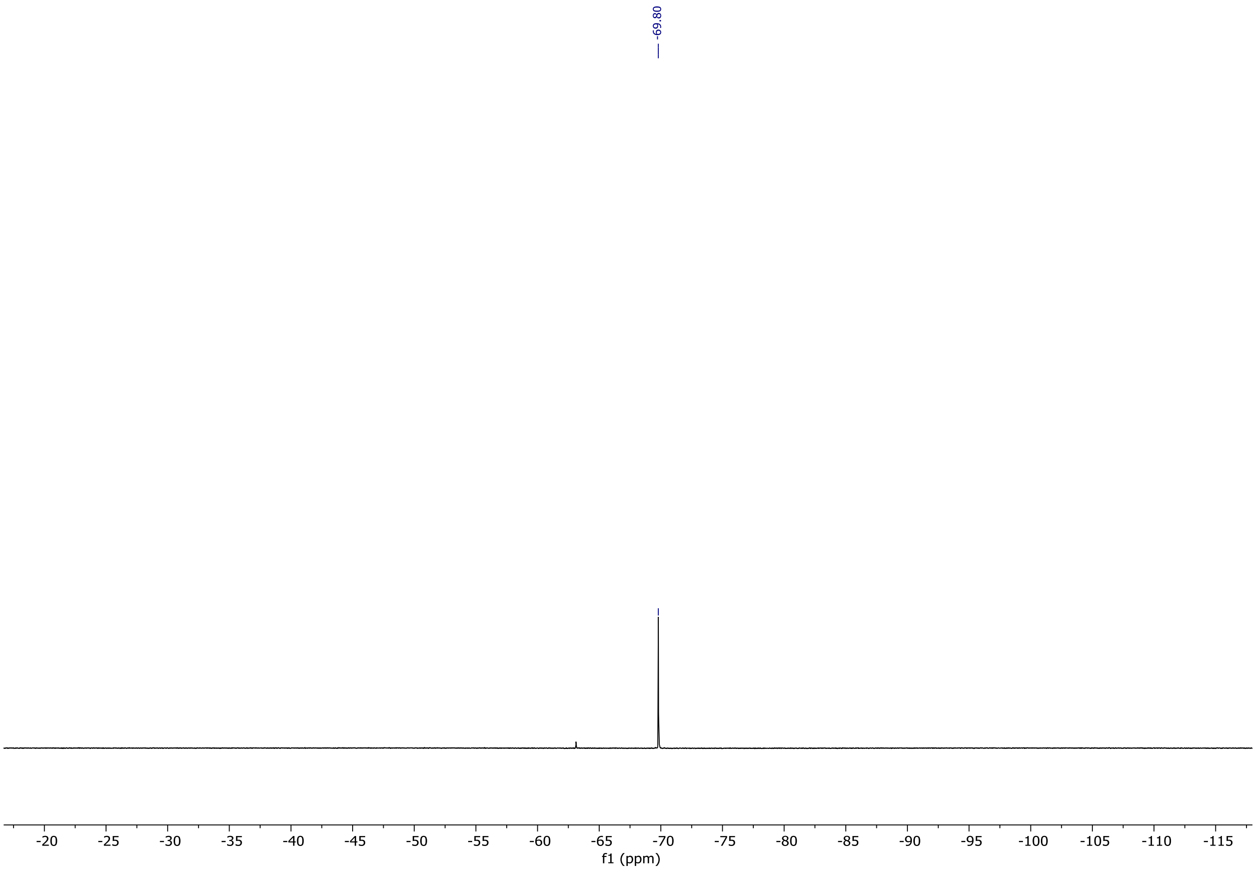

Supplementary Figure 52: ^{19}F NMR for compound **2k** in $CDCl_3$

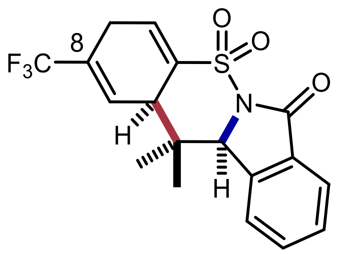

C8-trifluoromethyl benzo-fused cyclohexadiene-fused sultam (2l): Prepared according to **General Procedure C** with 76.7 mg, 0.2 mmol of **1l**. White powder (59.0 mg, 77% yield). R_f = 0.3 (1:1, Hex:EtOAc), one yellow spot, $KMnO_4$, UV.

^{1}H NMR (700 MHz, $CDCl_3$) = δ 7.95 (d, *J* = 7.6 Hz, 1H), 7.67 (td, *J* = 7.6, 1.3 Hz, 1H), 7.62 – 7.52 (m, 2H), 7.10 (t, *J* = 3.7 Hz, 1H), 6.49 (dt, *J* = 4.0, 1.8 Hz, 1H), 5.10 (s, 1H), 3.76 (s, 1H), 3.10 (s, 2H), 1.52 (s, 3H), 0.35 (s, 3H) ppm

^{13}C NMR (176 MHz, $CDCl_3$) = δ 163.8, 141.5, 134.5, 133.5, 130.7, 130.7, 129.5, 128.2 (q, *J* = 31.3 Hz), 125.9, 125.6 (q, *J* = 5.7 Hz), 124.2, 122.8 (q, *J* = 272.4 Hz), 69.1, 44.9, 43.2, 23.9, 22.4, 14.0 ppm

^{19}F NMR (377 MHz, $CDCl_3$) = δ -69.77 ppm

IR (*neat*) = 2973, 1729, 1467, 1350, 1315, 1168, 1109, 1011cm^{-1}

HRMS (ESI+) *m/z* calculated for $C_{18}H_{16}F_3NO_3S$ [M+H]$^+$: 384.0876, found 384.0884.

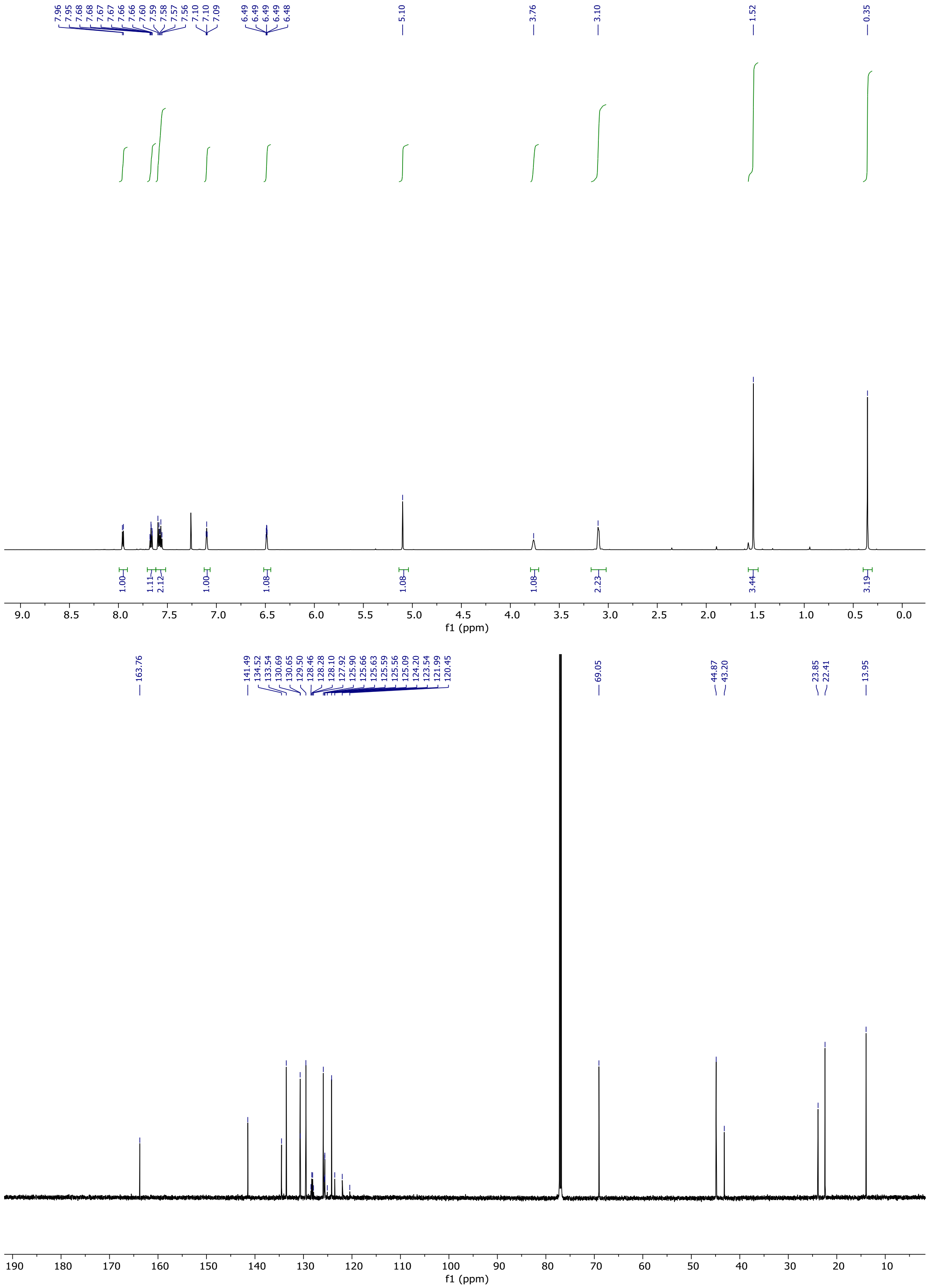

Supplementary Figure 53: ^{1}H (top) and ^{13}C NMR (bottom) for compound **2l** in $CDCl_3$

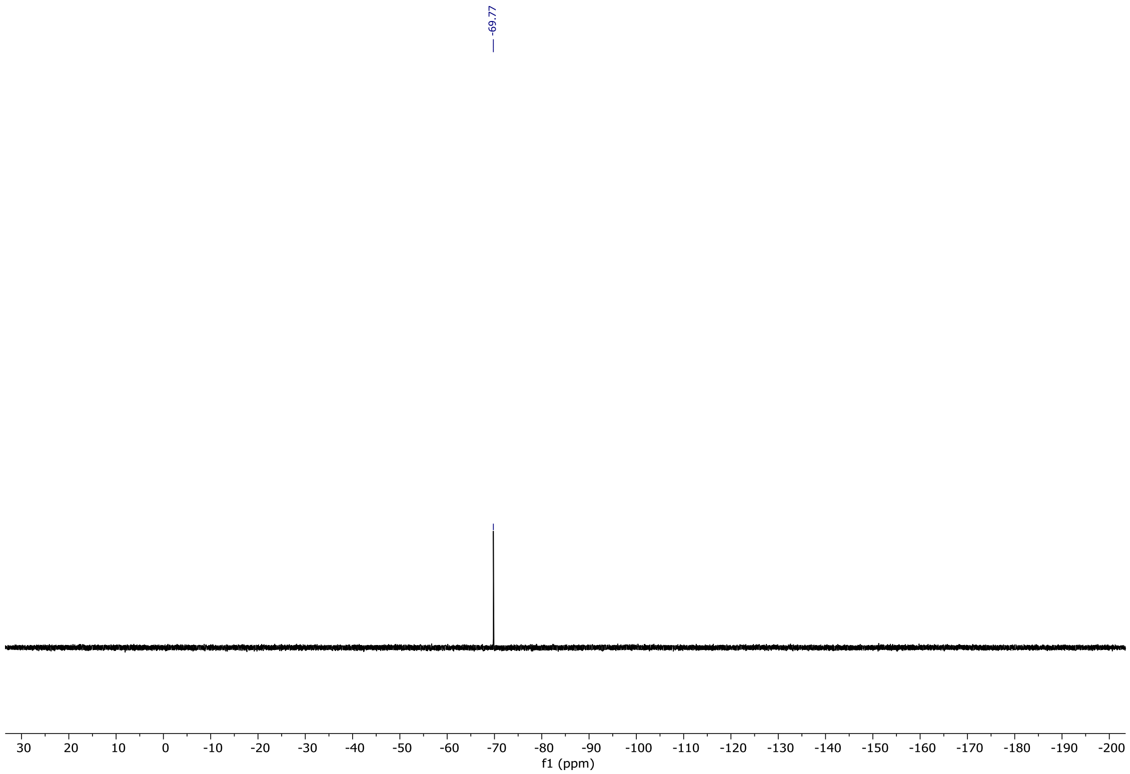

Supplementary Figure 54: ^{19}F NMR for compound **2l** in $CDCl_3$

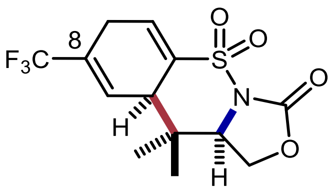

C8-trifluoromethyl carbamate cyclohexadiene-fused sultam (2m): Prepared according to **General Procedure C** with 67.5 mg, 0.2 mmol of **1m**. Off-white, tan powder (49.0 mg, 73% yield). R_f = 0.3 (1:1, Hex:EtOAc), one yellow spot, $KMnO_4$, UV.

^{1}H NMR (700 MHz, $CDCl_3$) = δ 7.06 (s, 1H), 6.36 (s, 1H), 4.42 (t, *J* = 8.8 Hz, 1H), 4.36 (dd, *J* = 8.4, 4.2 Hz, 1H), 4.21 (dd, *J* = 9.2, 4.2 Hz, 1H), 3.55 (d, *J* = 8.5 Hz, 1H), 3.12 (q, *J* = 6.2, 5.6 Hz, 2H), 1.11 (s, 3H), 0.87 (s, 3H) ppm

^{13}C NMR (176 MHz, $CDCl_3$) = δ 149.8, 133.8, 131.9, 128.3 (q, *J* = 31.9, 31.3 Hz), 125.0 (q, *J* = 5.7 Hz), 122.6 (q, *J* = 272.4 Hz), 64.2, 63.2, 45.1, 40.5, 23.9, 21.6, 13.7 ppm

^{19}F NMR (377 MHz, $CDCl_3$) = δ -69.90 ppm

IR (*neat*) = 2924, 1779, 1365, 1301, 1159, 1114, 1048, 967, 708, 648 cm^{-1}

HRMS (ESI+) *m/z* calculated for $C_{13}H_{14}F_3NO_4S$ $[M+H]^+$: 338.0668, found 338.0679.

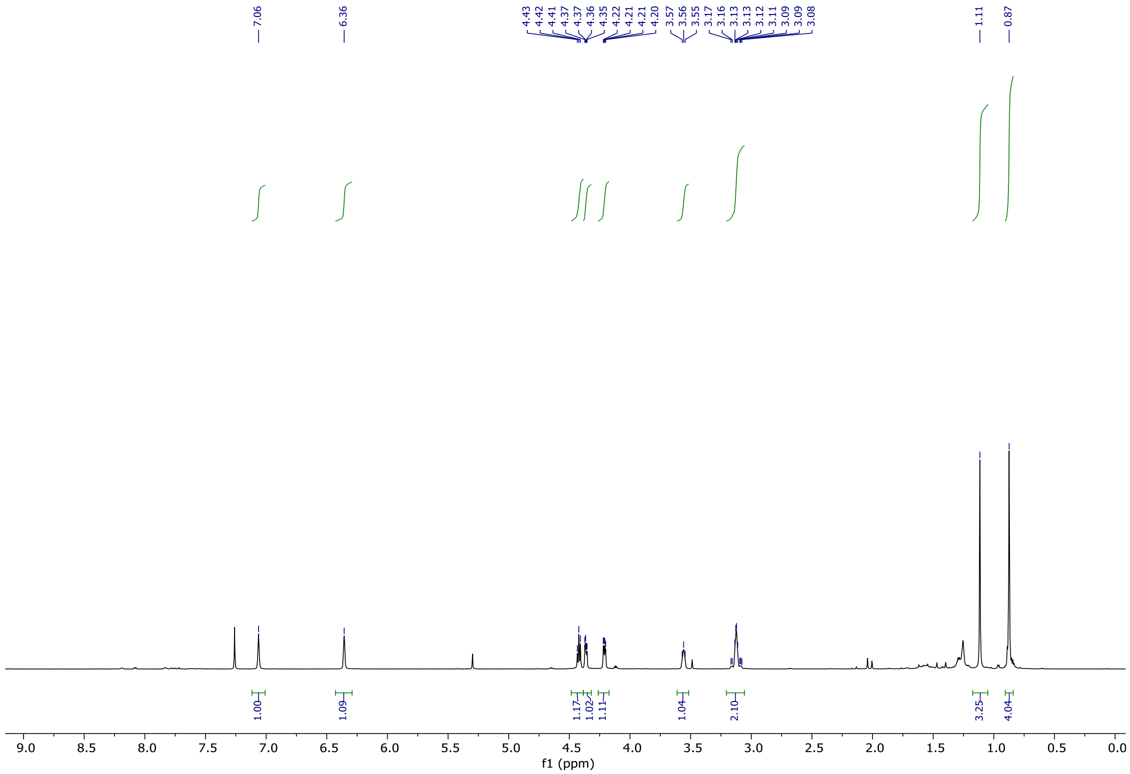

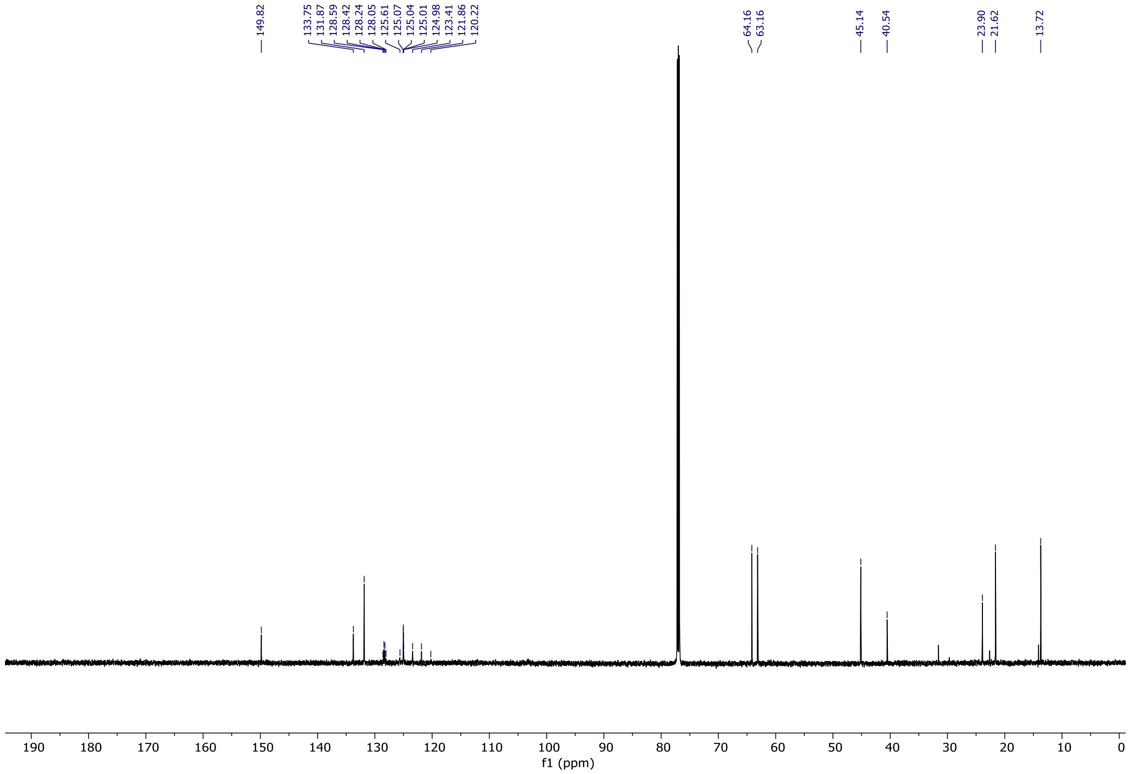

Supplementary Figure 55: ^{1}H (top) and ^{13}C NMR (bottom) for compound **2m** in $CDCl_3$

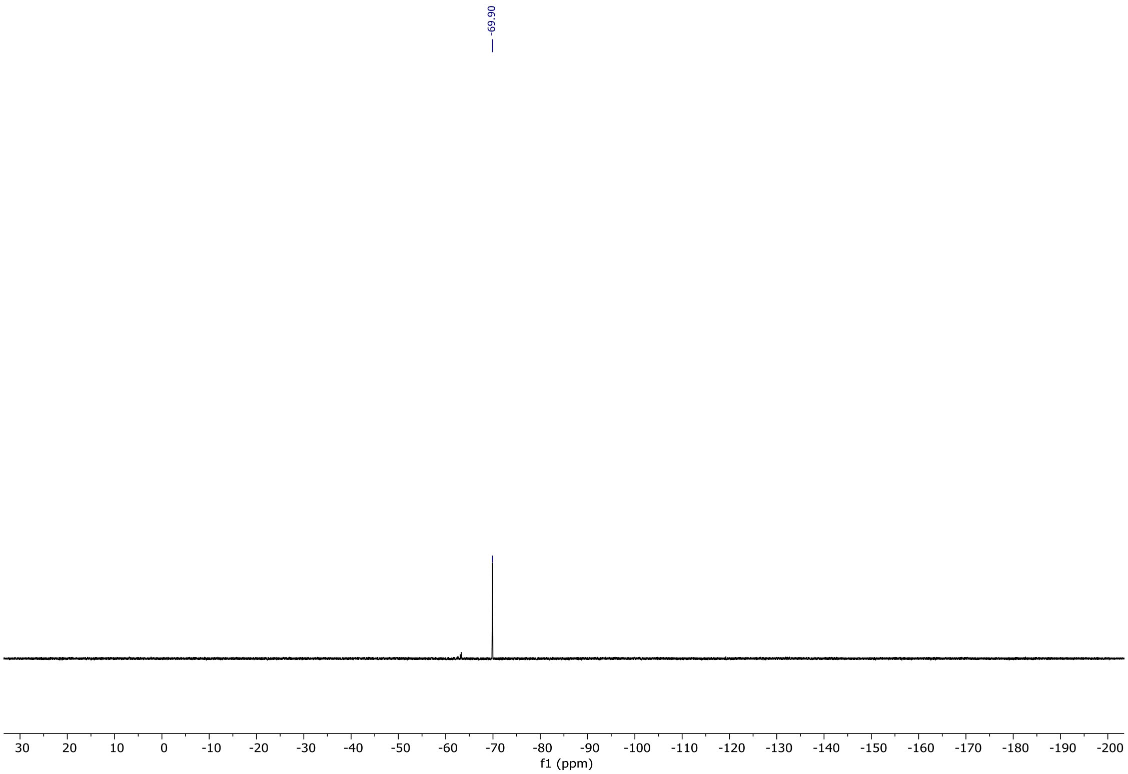

Supplementary Figure 56: ^{19}F NMR for compound **2m** in $CDCl_3$

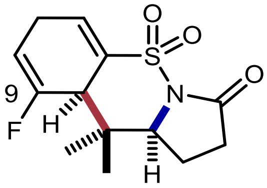

C9-fluoro cyclohexadiene-fused sultam (2n): Prepared according to **General Procedure C** with 57.1 mg, 0.2 mmol of **1n**. Light-yellow powder (23.4 mg, 41% yield). R_f = 0.3 (1:1, Hex:EtOAc), one yellow spot, $KMnO_4$, UV.

^{1}H NMR (700 MHz, $CDCl_3$) = δ 6.98 (s, 1H), 5.48 (ddt, *J* = 17.7, 4.0, 2.3 Hz, 1H), 4.09 (dd, *J* = 8.3, 6.2 Hz, 1H), 3.59 (q, *J* = 5.9 Hz, 1H), 3.15 – 2.93 (m, 2H), 2.55 – 2.42 (m, 2H), 2.25 – 2.11 (m, 1H), 1.91 (dddd, *J* = 13.5, 9.7, 7.6, 6.2 Hz, 1H), 1.17 (d, *J* = 3.8 Hz, 3H), 0.89 (s, 3H) ppm

^{13}C NMR (176 MHz, $CDCl_3$) = δ 172.8, 155.9 (d, *J* = 258.4 Hz), 134.1 (d, *J* = 10.6 Hz), 133.9 (d, *J* = 2.1 Hz), 102.7 (d, *J* = 19.4 Hz), 66.8, 46.9 (d, *J* = 26.4 Hz), 41.6 (d, *J* = 4.3 Hz), 30.8, 25.8 (d, *J* = 7.5 Hz), 23.4 (d, *J* = 7.6 Hz), 19.1, 13.5 ppm

^{19}F NMR (377 MHz, $CDCl_3$) = δ -100.54 (d, *J* = 17.6 Hz) ppm

IR (*neat*) = 2976, 2936, 1738, 1703, 1343, 1170, 1132, 977, 855, 673, 634 cm^{-1}

HRMS (ESI+) *m/z* calculated for $C_{13}H_{16}FNO_3S$ $[M+H]^+$: 286.0908, found 286.0908.

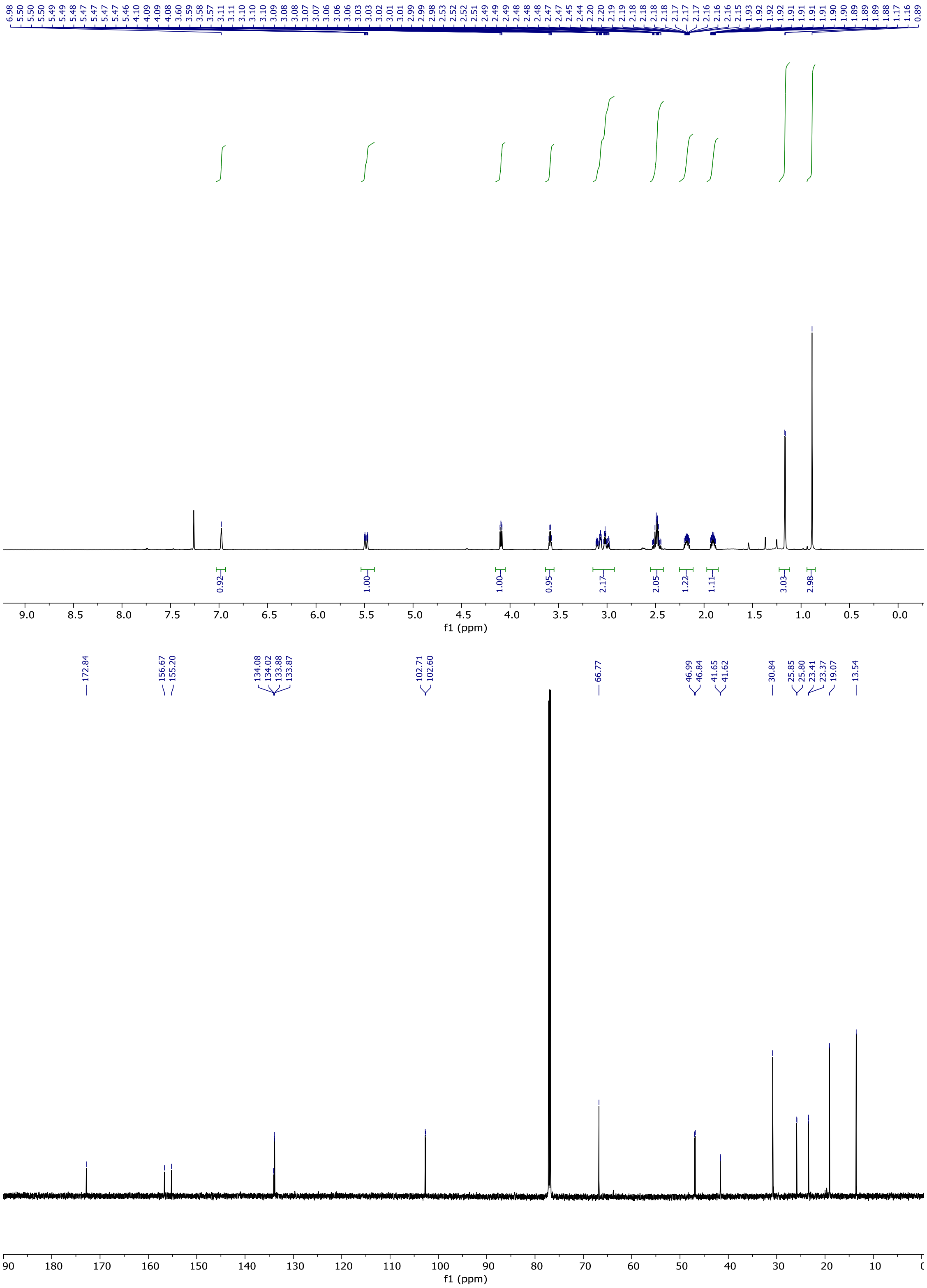

Supplementary Figure 57: ^{1}H (top) and ^{13}C NMR (bottom) for compound **2n** in $CDCl_3$

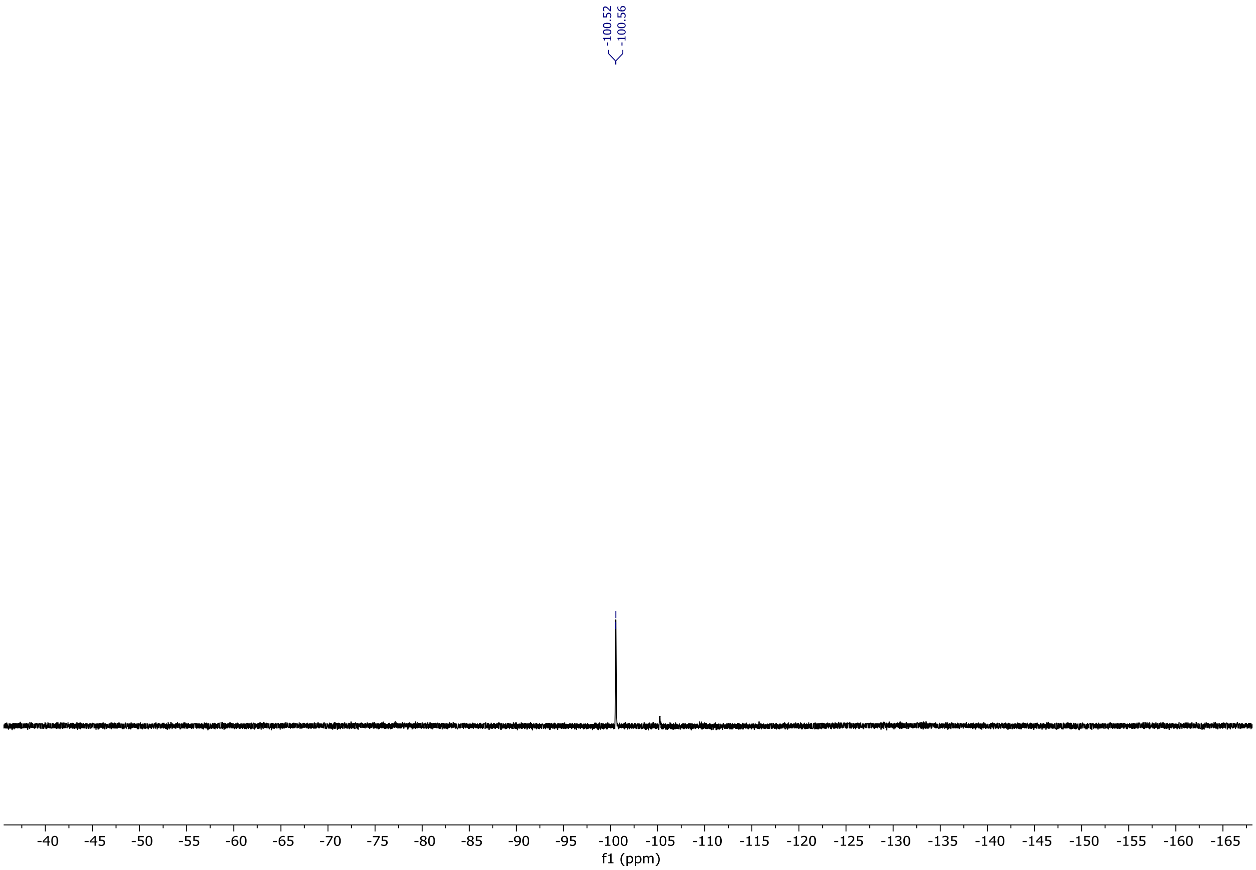

Supplementary Figure 58: ^{19}F NMR for compound **2n** in $CDCl_3$

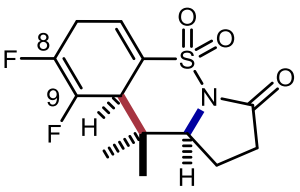

C8, C9-difluoro cyclohexadiene-fused sultam (2o): Prepared according to **General Procedure C** with 60.7 mg, 0.2 mmol of **1o**. Off-white, tan powder (27.9 mg, 46% yield). R_f = 0.4 (1:1, Hex:EtOAc), one yellow spot, $KMnO_4$, UV.

^{1}H NMR (700 MHz, $CDCl_3$) = δ 6.85 (s, 1H), 4.10 (t, *J* = 7.0 Hz, 1H), 3.76 (s, 1H), 3.39 – 3.15 (m, 2H), 2.59 – 2.42 (m, 2H), 2.20 (dddd, *J* = 18.6, 10.3, 6.3, 1.9 Hz, 1H), 2.00 – 1.84 (m, 1H), 1.18 (s, 3H), 0.90 (s, 3H) ppm

^{13}C NMR (176 MHz, $CDCl_3$) = δ 172.6, 141.7 (dd, *J* = 256.1, 12.6 Hz), 139.5 (dd, *J* = 257.5, 12.7 Hz), 134.1 (d, *J* = 9.2 Hz), 130.8 (d, *J* = 10.2 Hz), 66.6, 48.1 (d, *J* = 21.9 Hz), 42.11 (dd, *J* = 4.1, 2.0 Hz), 30.8, 27.4 (d, *J* = 25.2 Hz), 23.1 (d, *J* = 7.2 Hz), 19.0, 13.5 ppm

^{19}F NMR (377 MHz, $CDCl_3$) = δ -137.19, -137.33 ppm

IR (*neat*) = 2976, 1735, 1345, 1200, 1178, 1120, 1010, 962, 879, 826 cm^{-1}

HRMS (ESI+) *m/z* calculated for $C_{13}H_{15}F_2NO_3S$ $[M+H]^+$: 304.0813, found 304.0816.

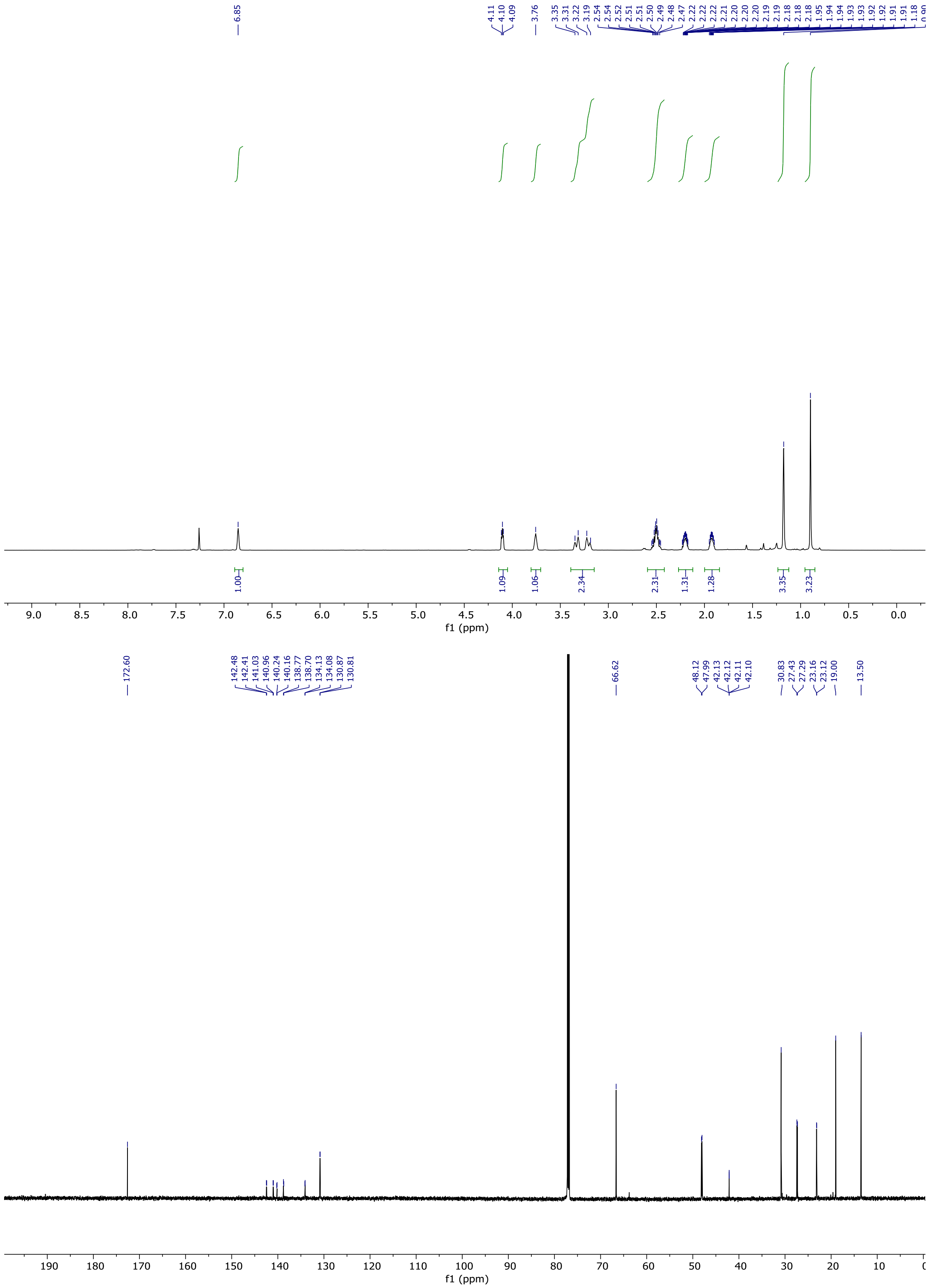

Supplementary Figure 59: ^{1}H (top) and ^{13}C NMR (bottom) for compound **2o** in $CDCl_3$

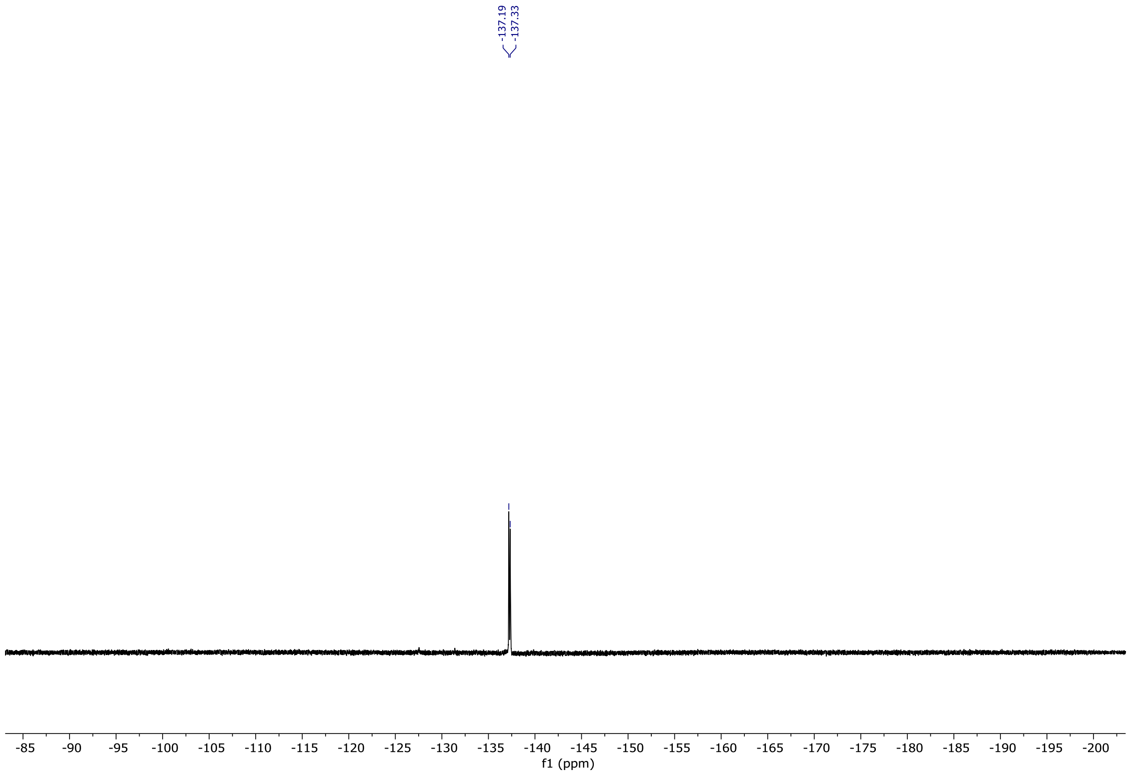

Supplementary Figure 60: ^{19}F NMR for compound **2o** in $CDCl_3$

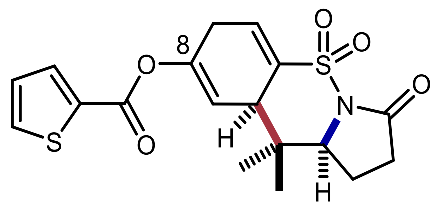

C8-thiophene-2-carboxylate cyclohexadiene-fused sultam (2p): Prepared according to **General Procedure C** with 78.7 mg, 0.2 mmol of **1p**. Off-white, tan powder (35.9 mg, 46% yield). R_f = 0.3 (1:1, Hex:EtOAc), one yellow spot, $KMnO_4$, UV.

^{1}H NMR (700 MHz, $CDCl_3$) = δ 7.88 (dd, *J* = 3.7, 1.3 Hz, 1H), 7.66 (dd, *J* = 4.9, 1.3 Hz, 1H), 7.16 (dd, *J* = 5.0, 3.7 Hz, 1H), 6.96 (t, *J* = 3.6 Hz, 1H), 5.65 (dd, *J* = 4.3, 2.0 Hz, 1H), 4.21 – 4.06 (m, 1H), 3.58 (td, *J* = 7.1, 4.1 Hz, 1H), 3.35 – 3.02 (m, 2H), 2.51 (ddd, *J* = 9.2, 7.4, 2.0 Hz, 2H), 2.27 – 2.12 (m, 1H), 2.01 – 1.86 (m, 1H), 1.07 (s, 3H), 0.92 (s, 3H) ppm

^{13}C NMR (176 MHz, $CDCl_3$) = δ 172.8, 160.1, 146.5, 134.8, 134.6, 133.8, 132.2, 132.0, 128.1, 111.2, 67.2, 46.6, 41.6, 31.0, 28.3, 22.0, 19.1, 13.5 ppm

IR (*neat*) = 2975, 2916, 1736, 1701, 1520, 1469, 1342, 1272, 1204, 1175, 1131, 875, 743 cm^{-1}

HRMS (ESI+) *m/z* calculated for $C_{18}H_{19}NO_5S_2$ $[M+H]^+$: 394.0777, found 394.0780.

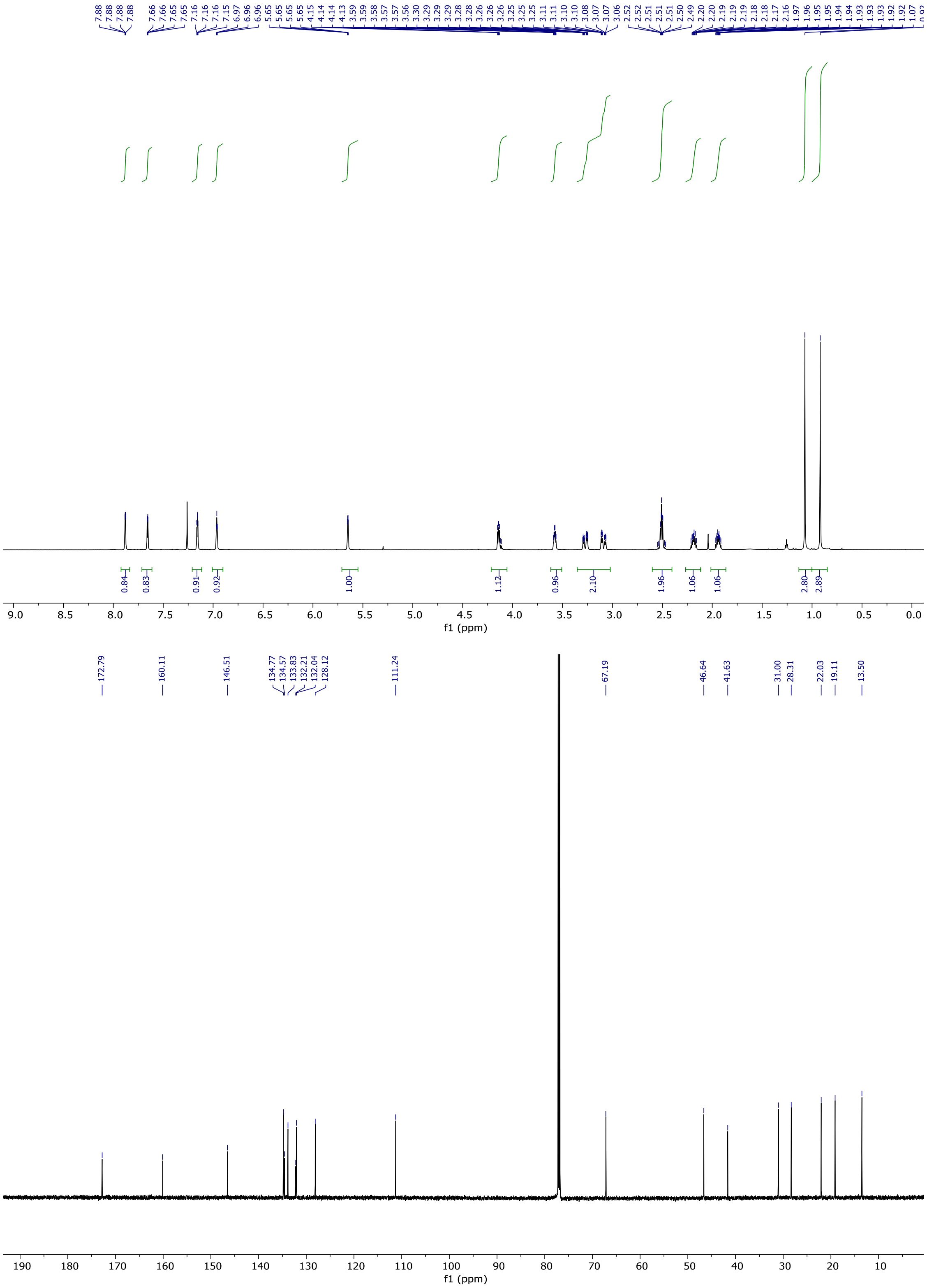

Supplementary Figure 61: ^1H (top) and ^{13}C NMR (bottom) for compound **2p** in $CDCl_3$

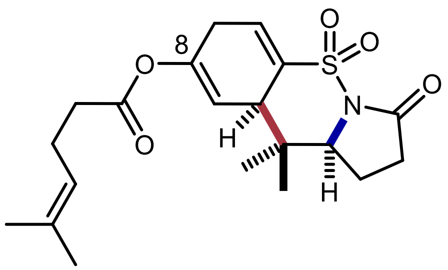

C8-homoprenyl carboxylate cyclohexadiene-fused sultam (2q): Prepared according to **General Procedure C** with 78.7 mg, 0.2 mmol of **1q**. Light-yellow powder (33.3 mg, 42% yield). R_f = 0.2 (1:1, Hex:EtOAc), one yellow spot, $KMnO_4$, UV.

^{1}H NMR (700 MHz, $CDCl_3$) = δ 6.91 (t, *J* = 3.1 Hz, 1H), 5.46 (dd, *J* = 4.4, 1.9 Hz, 1H), 5.10 (ddt, *J* = 8.6, 7.2, 1.4 Hz, 1H), 4.11 (dd, *J* = 8.4, 5.8 Hz, 1H), 3.51 (td, *J* = 7.1, 4.2 Hz, 1H), 3.18 – 2.87 (m, 2H), 2.49 (ddd, *J* = 9.1, 7.3, 3.5 Hz, 2H), 2.44 (t, *J* = 7.5 Hz, 2H), 2.34 (q, *J* = 7.5 Hz, 2H), 2.21 – 2.11 (m, 1H), 1.92 (dddd, *J* = 13.3, 9.5, 7.6, 5.8 Hz, 2H), 1.70 (d, *J* = 1.8 Hz, 3H), 1.63 (s, 3H), 1.04 (s, 3H), 0.86 (s, 3H) ppm

^{13}C NMR (176 MHz, $CDCl_3$) = δ 172.8, 171.5, 146.6, 134.5, 133.6, 132.1, 121.8, 110.5, 67.2, 46.5, 41.6, 34.3, 31.0, 28.2, 25.7, 23.5, 22.0, 19.1, 17.7, 13.4 ppm

IR (*neat*) = 2972, 2932, 2257, 1742, 1452, 1343, 1168, 1129, 908, 726 cm^{-1}

HRMS (ESI+) *m/z* calculated for $C_{20}H_{27}NO_5S$ $[M+H]^+$: 394.1683, found 394.1690.

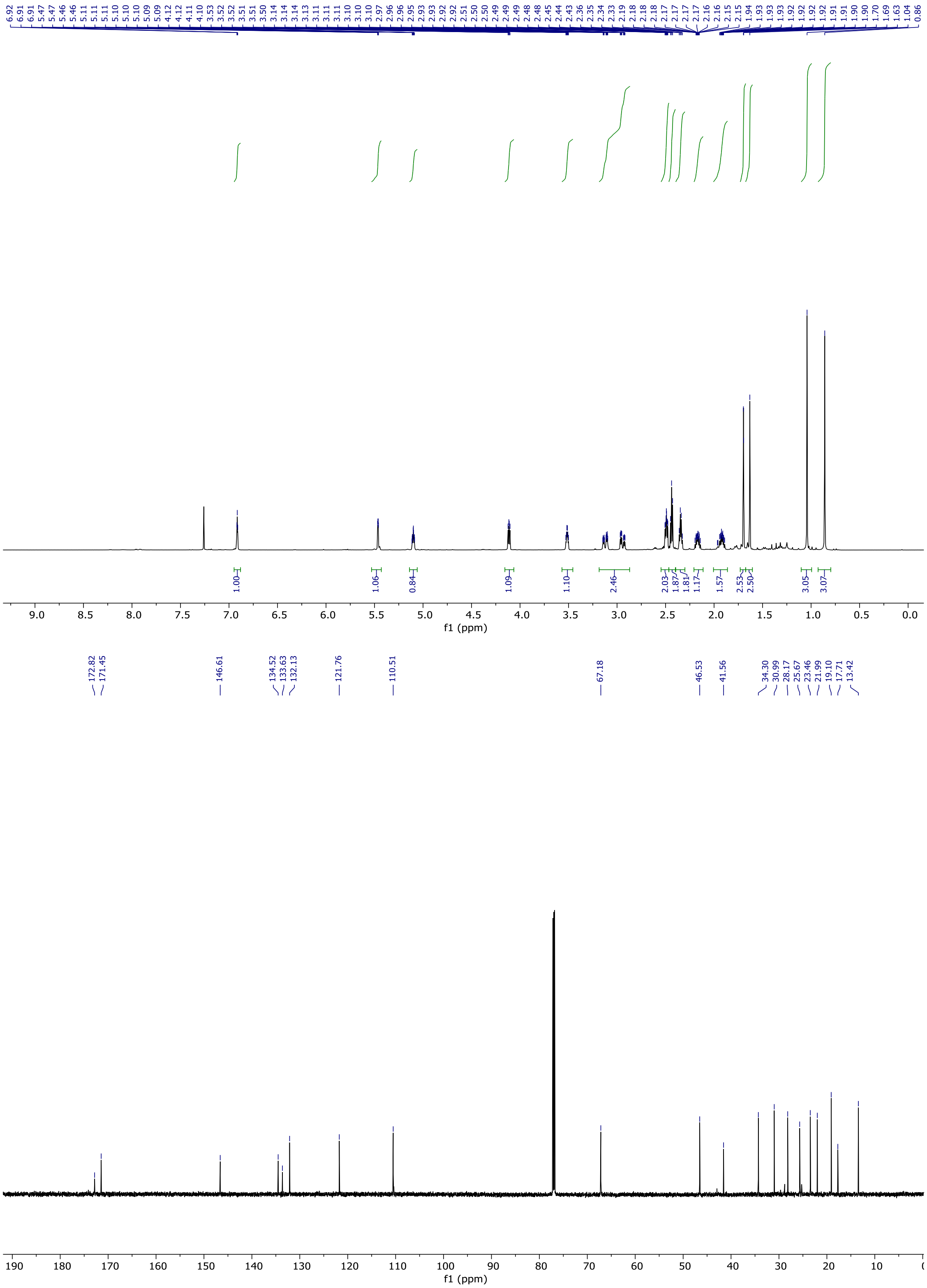

Supplementary Figure 62: ^{1}H (top) and ^{13}C NMR (bottom) for compound **2q** in $CDCl_3$

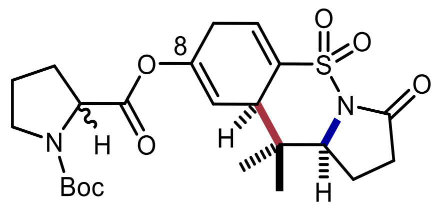

C8-*N*-Boc-proline cyclohexadiene-fused sultam (2r): Prepared according to **General Procedure C** with 75.0 mg, 0.16 mmol of **1r**. Light-yellow foam (33.6 mg, 45% yield). R_f = 0.2 (1:1, Hex:EtOAc), one yellow spot, $KMnO_4$, UV.

^{1}H NMR (700 MHz, $CDCl_3$) = δ 6.96 – 6.84 (m, 1H), 5.58 – 5.45 (m, 1H), 4.33 (ddd, *J* = 37.3, 8.6, 3.9 Hz, 1H), 4.11 (td, *J* = 8.8, 5.5 Hz, 1H), 3.62 – 3.38 (m, 3H), 3.13 (ddt, *J* = 23.1, 7.3, 2.6 Hz, 1H), 3.06 – 2.90 (m, 1H), 2.56 – 2.41 (m, 2H), 2.41 – 2.22 (m, 1H), 2.22 – 2.10 (m, 1H), 2.10 – 1.84 (m, 4H), 1.45 (d, *J* = 16.4 Hz, 9H), 1.03 (d, *J* = 2.7 Hz, 3H), 0.88 – 0.80 (m, 3H) ppm

^{13}C NMR (176 MHz, $CDCl_3$) = δ 172.8, 171.2, 154.4, 153.6, 146.6, 146.5, 134.6, 134.5, 132.2, 131.8, 110.7, 80.3, 80.2, 67.2, 67.11, 58.9, 46.6, 46.4, 41.5, 31.0, 29.9, 28.4, 28.0, 24.5, 23.6, 22.0, 19.1, 13.4 ppm

IR (*neat*) = 2975, 2249, 1745, 1702, 1405, 1361, 1339, 1163, 1143, 906, 729 cm^{-1}

HRMS (ESI+) *m/z* calculated for $C_{23}H_{32}N_2O_7S$ $[M+H]^+$: 481.2003, found 481.2000.

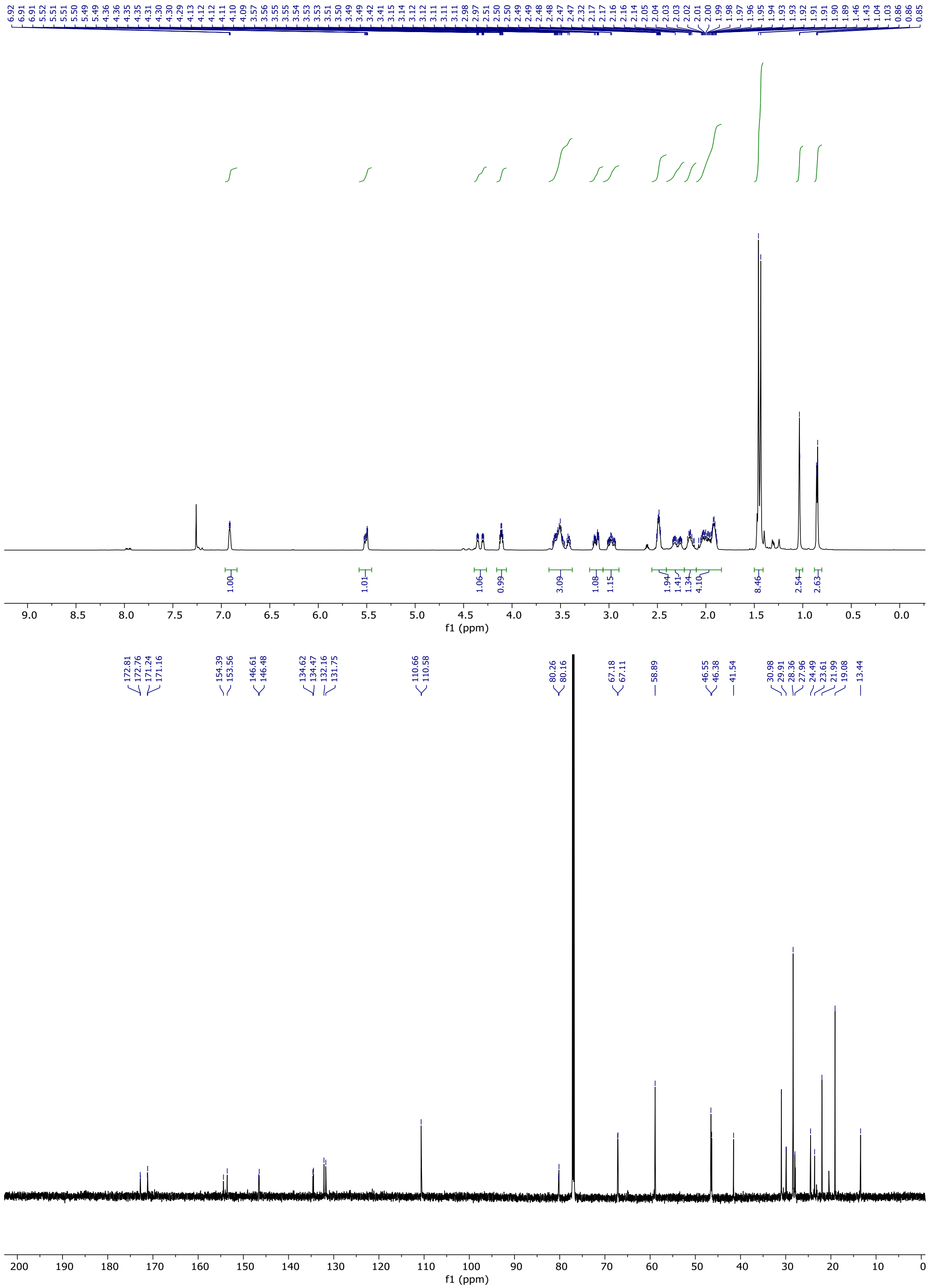

Supplementary Figure 63: ^{1}H (top) and ^{13}C NMR (bottom) for compound **2r** in $CDCl_3$

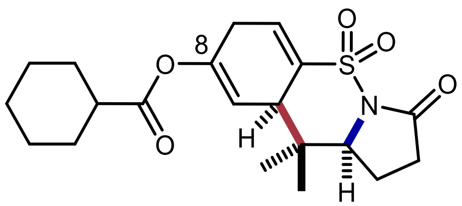

C8-cyclohexyl carboxylate cyclohexadiene-fused sultam (2s): Prepared according to **General Procedure C** with 78.7 mg, 0.2 mmol of **1s.** Off-white, tan powder (41.7 mg, 53% yield). R_f = 0.4 (1:1, Hex:EtOAc), one yellow spot, $KMnO_4$, UV.

^{1}H NMR (700 MHz, $CDCl_3$) = δ 6.91 (t, *J* = 3.7 Hz, 1H), 5.44 (dd, *J* = 4.3, 1.9 Hz, 1H), 4.11 (dd, *J* = 8.4, 5.7 Hz, 1H), 3.51 (td, *J* = 7.1, 4.1 Hz, 1H), 3.18 – 2.86 (m, 2H), 2.49 (ddd, *J* = 10.0, 7.2, 3.3 Hz, 2H), 2.40 (tt, *J* = 11.3, 3.7 Hz, 1H), 2.23 – 2.11 (m, 1H), 2.01 – 1.85 (m, 3H), 1.77 (dq, *J* = 7.8, 3.9 Hz, 2H), 1.72 – 1.60 (m, 1H), 1.54 – 1.43 (m, 2H), 1.38 – 1.15 (m, 3H), 1.04 (s, 3H), 0.86 (s, 3H) ppm

^{13}C NMR (176 MHz, $CDCl_3$) = δ 174.1, 172.8, 146.6, 134.5, 132.1, 110.4, 67.2, 46.6, 42.9, 41.6, 31.0, 28.8, 28.8, 28.1, 25.6, 25.2, 25.2, 22.0, 19.1, 13.4 ppm

IR (*neat*) = 2937, 2857, 1742, 1704, 1450, 1344, 1132, 1001, 894, 668 cm^{-1}

HRMS (ESI+) *m/z* calculated for $C_{20}H_{27}NO_5S$ $[M+H]^+$: 394.1683, found 394.1690.

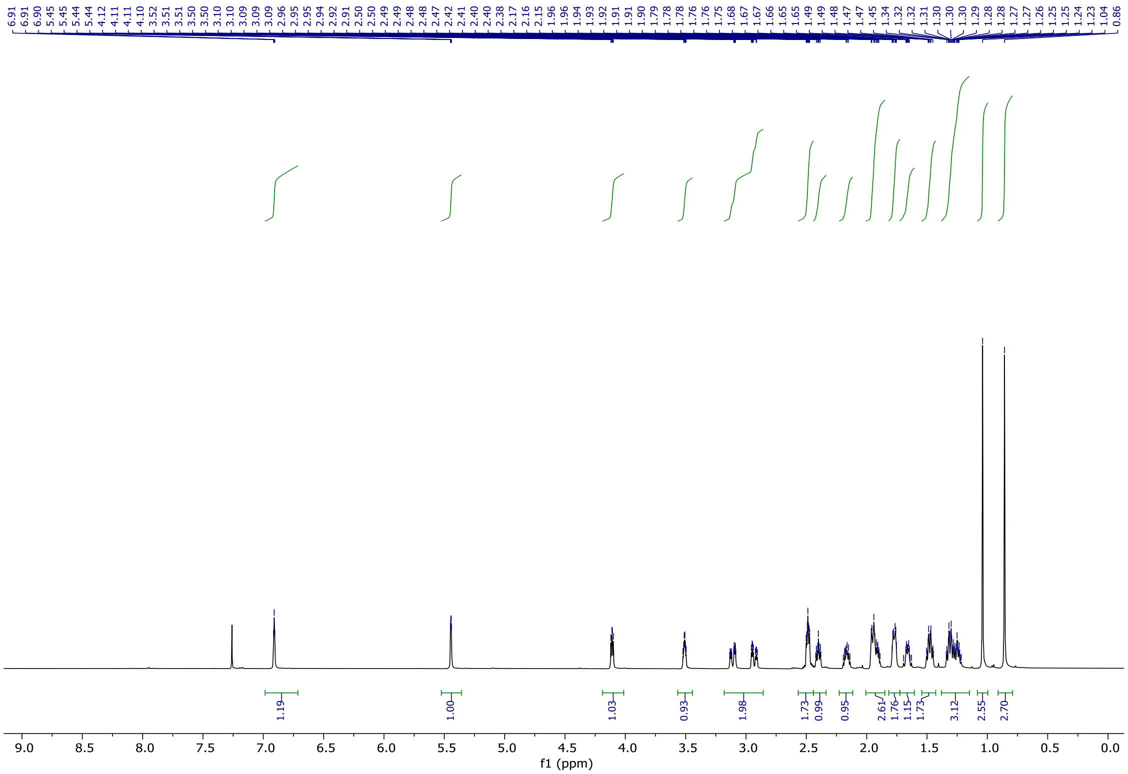

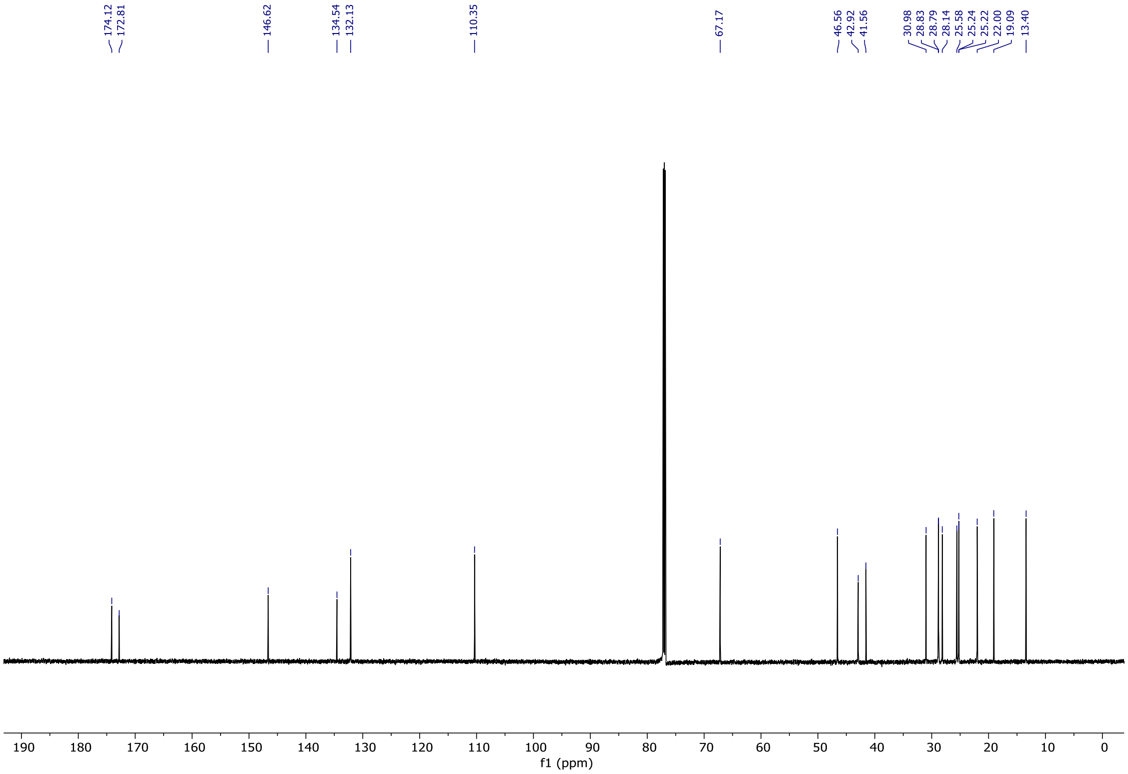

Supplementary Figure 64: ^{1}H (top) and ^{13}C NMR (bottom) for compound **2s** in $CDCl_3$

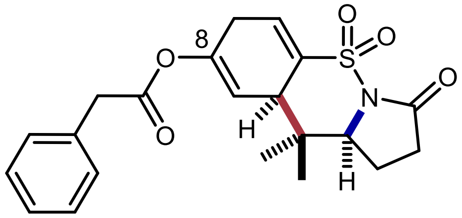

C8-benzyl carboxylate cyclohexadiene-fused sultam (2t): Prepared according to **General Procedure C** with 74.5 mg, 0.19 mmol of **1t**. Light-yellow oil (20.3 mg, 27% yield). R_f = 0.3 (1:1, Hex:EtOAc), one yellow spot, $KMnO_4$, UV.

^{1}H NMR (700 MHz, $CDCl_3$) = δ 7.41 – 7.33 (m, 2H), 7.33 – 7.27 (m, 3H), 6.89 (t, *J* = 3.7 Hz, 1H), 5.49 (dd, *J* = 4.4, 1.9 Hz, 1H), 4.10 (dd, *J* = 8.5, 5.7 Hz, 1H), 3.73 (s, 2H), 3.50 (td, *J* = 7.1, 4.2 Hz, 1H), 3.19 – 2.85 (m, 3H), 2.57 – 2.42 (m, 2H), 2.24 – 2.07 (m, 2H), 1.99 – 1.84 (m, 1H), 1.03 (s, 3H), 0.84 (s, 3H) ppm

^{13}C NMR (176 MHz, $CDCl_3$) = δ 172.8, 169.7, 146.6, 134.5, 133.0, 132.0, 129.2, 128.8, 127.5, 110.8, 67.2, 46.5, 41.6, 41.1, 31.0, 28.1, 22.0, 19.1, 13.4 ppm

IR (*neat*) = 2971, 1740, 1340, 1164, 1125, 1006, 952, 698 cm^{-1}

HRMS (ESI+) *m/z* calculated for $C_{21}H_{23}NO_5S$ $[M+H]^+$: 402.1370, found 402.1371.

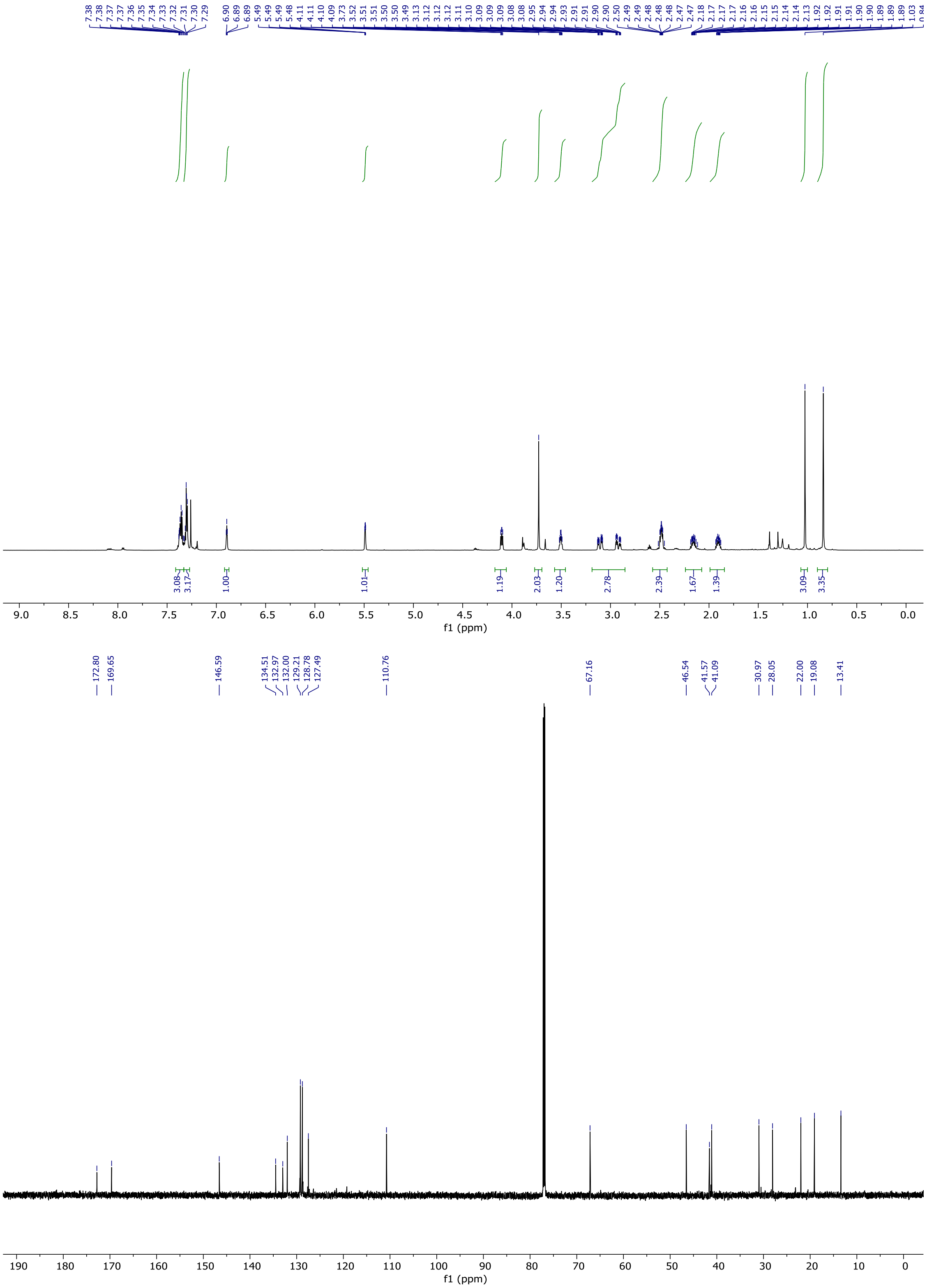

Supplementary Figure 65: ^1H (top) and ^{13}C NMR (bottom) for compound **2t** in $CDCl_3$

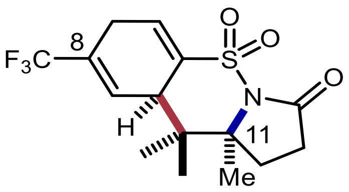

C8-trifluoromethyl-C11-methyl cyclohexadiene-fused sultam (2u): Prepared according to **General Procedure C** with 69.9 mg, 0.2 mmol of **1u**. Light-yellow powder (23.6 mg, 34% yield). R_f = 0.3 (1:1, Hex:EtOAc), one yellow spot, $KMnO_4$, UV.

^{1}H NMR (700 MHz, $CDCl_3$) = δ 7.02 (d, *J* = 3.8 Hz, 1H), 6.36 (dq, *J* = 3.5, 1.8 Hz, 1H), 3.81 (t, *J* = 3.9 Hz, 1H), 3.08 (q, *J* = 6.6, 6.2 Hz, 2H), 2.57 (ddd, *J* = 18.1, 10.2, 7.8 Hz, 1H), 2.47 (ddd, *J* = 18.1, 10.3, 5.0 Hz, 1H), 2.23 (ddd, *J* = 13.2, 10.3, 7.9 Hz, 1H), 1.80 (ddd, *J* = 13.1, 10.2, 5.0 Hz, 1H), 1.67 (s, 3H), 1.10 (s, 3H), 0.88 (s, 3H) ppm

^{13}C NMR (176 MHz, $CDCl_3$) = δ 172.8, 134.5, 131.1, 128.1 (q, *J* = 31.4 Hz). 126.5 (q, *J* = 5.5 Hz), 122.8 (q, *J* = 272.5 Hz), 72.1, 43.7, 40.7, 29.9, 28.2, 23.8, 23.0, 21.4, 17.9 ppm

^{19}F NMR (377 MHz, $CDCl_3$) = δ -69.85 ppm

IR (*neat*) = 2962, 1754, 1654, 1337, 1300, 1253, 1163, 1116, 977, 896, 707 cm^{-1}

HRMS (ESI+) *m/z* calculated for $C_{15}H_{18}F_3NO_3S$ $[M+H]^+$: 350.1032, found 350.1041.

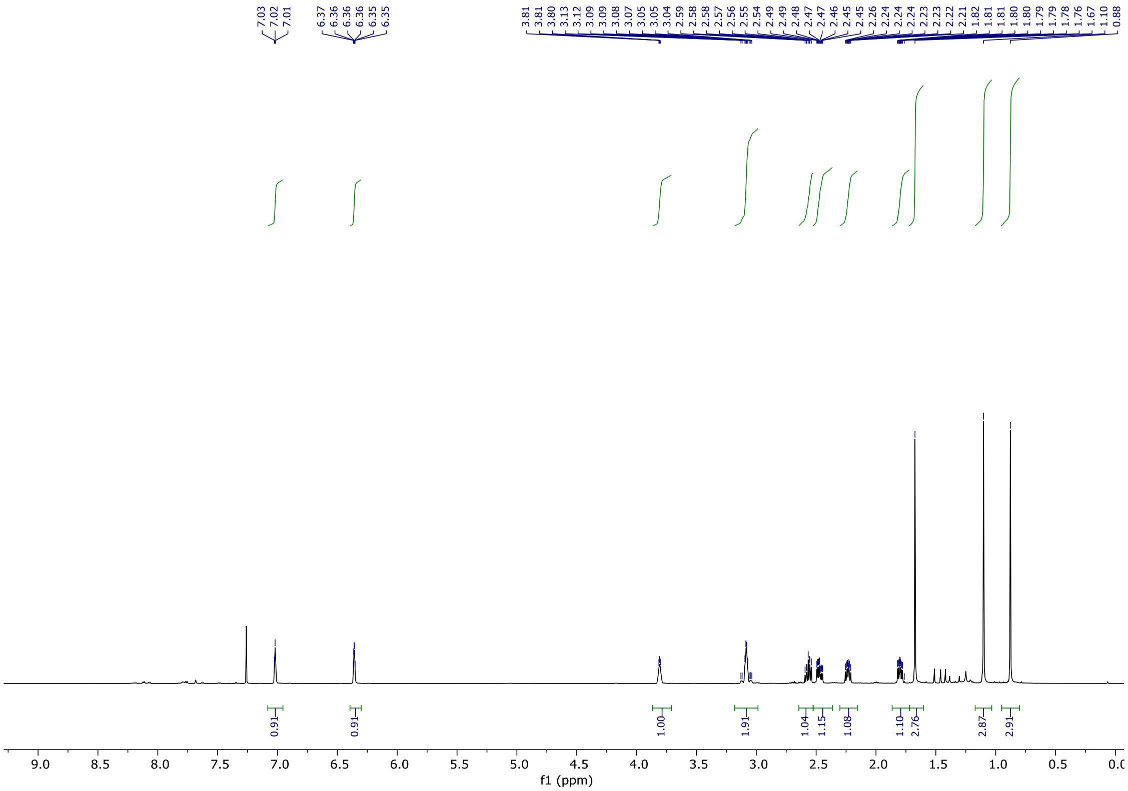

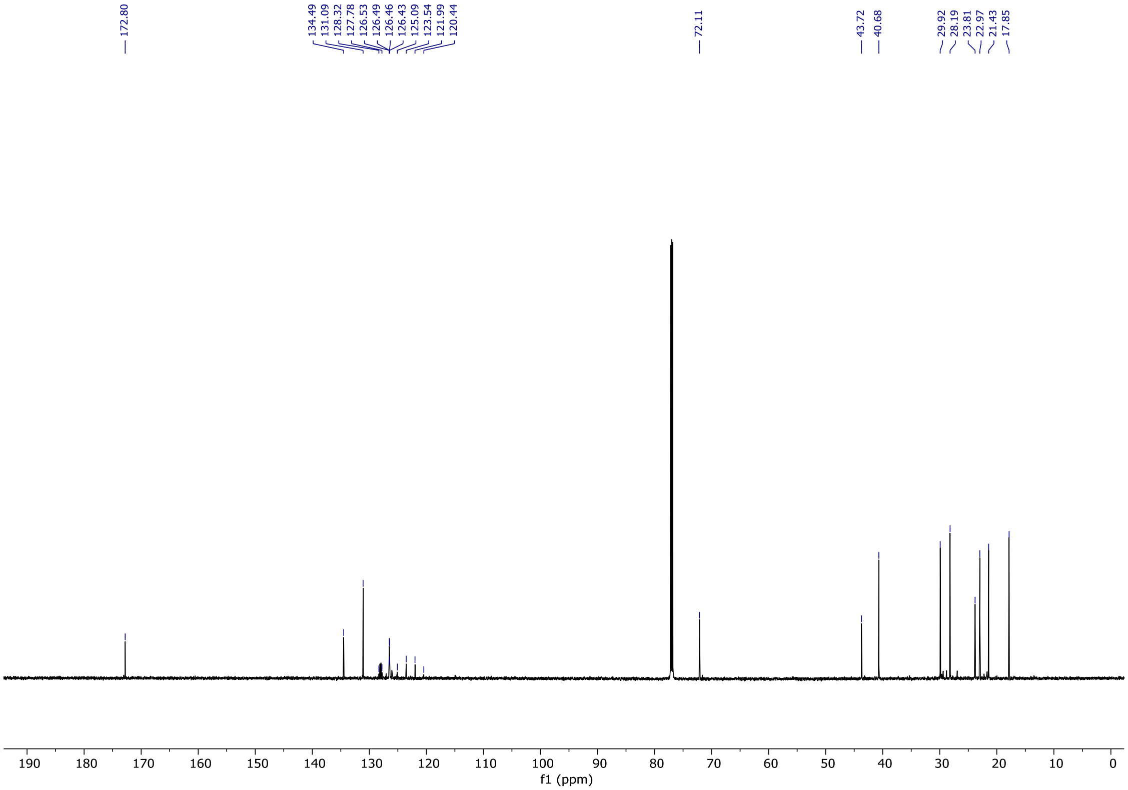

Supplementary Figure 66: ^{1}H (top) and ^{13}C NMR (bottom) for compound **2u** in $CDCl_3$

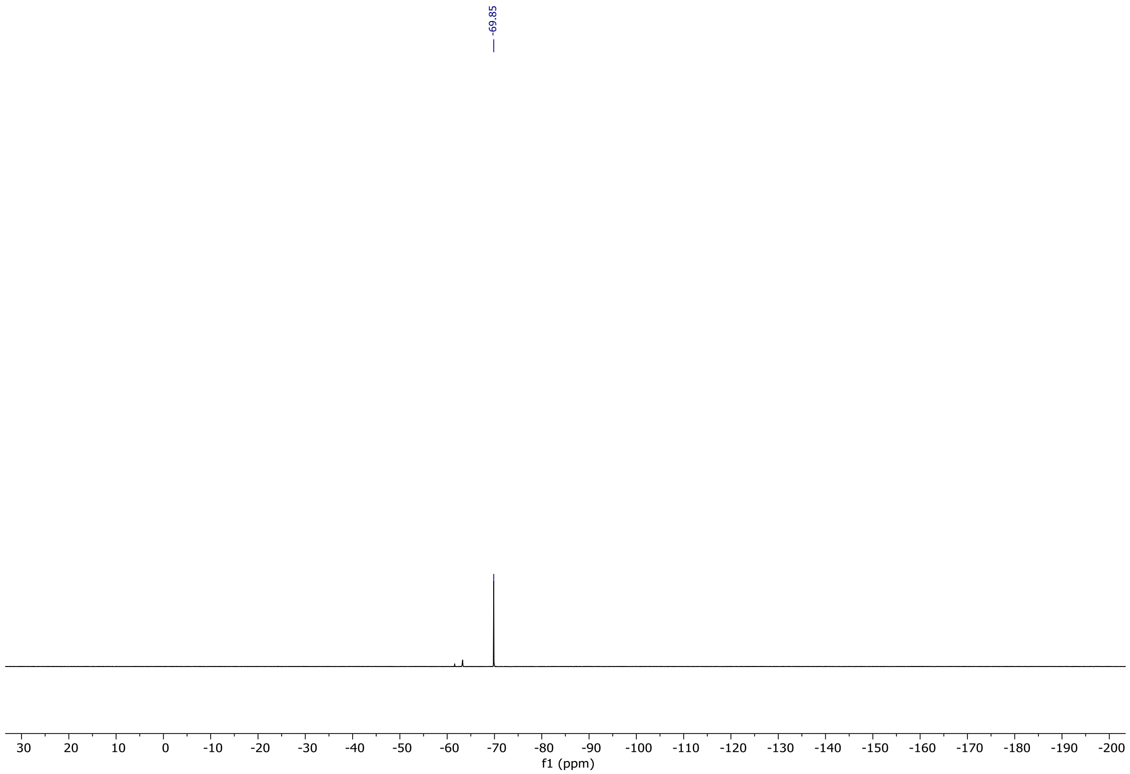

Supplementary Figure 67: ^{19}F NMR for compound **2u** in $CDCl_3$

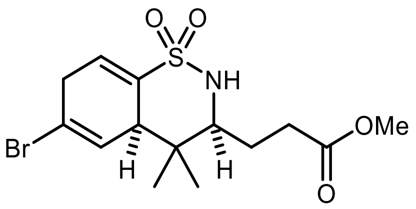

Amide alcoholysis (3d): To a stirred suspension of **2d** (20.3 mg, 59 µmol) in 2 mL MeOH in a 2 dram vial at room temperature was added saturated aqueous K_2CO_3 solution (1 ml) and the reaction was stirred vigorously for 20 minutes before quenching by transfer into a separatory funnel containing 30 mL sat. aq. NH_4Cl. The reaction vial was rinsed with 5 mL additional water and 10 mL CH_2Cl_2. The aqueous phase was further washed with CH_2Cl_2 (2 x 10 mL) and the combined organic phases were dried over $MgSO_4$, filtered, and concentrated to afford the product as a colorless solid (20.3 mg, 54 µmol, 92%). R_f = 0.63 (1:1, Hex:EtOAc), one yellow spot, $KMnO_4$.

^{1}H NMR (500 MHz, $CDCl_3$) = δ 6.59 (t, *J* = 3.4 Hz, 1H), 6.17 – 6.13 (m, 1H), 4.04 (d, *J* = 11.4 Hz, 1H), 3.69 (s, 3H), 3.43 – 3.12 (m, 4H), 2.61 – 2.42 (m, 2H), 2.11 – 2.01 (m, 1H), 1.53 – 1.44 (m, 1H), 1.10 (s, 3H), 0.81 (s, 3H).

^{13}C NMR (176 MHz, $CDCl_3$) = δ 174.1, 135.7, 128.3, 125.1, 120.6, 63.7, 52.0, 48.8, 42.3, 35.2, 30.8, 24.2, 23.1, 13.8.

IR (neat) = 3240, 2973, 2920, 1726, 1672, 1418, 1333, 1307, 1149, 1070 cm^{-1}.

HRMS (ESI+) m/z calculated for $C_{14}H_{20}BrNO_4S$ $[M+H]^+$: 378.0369, found 378.0370.

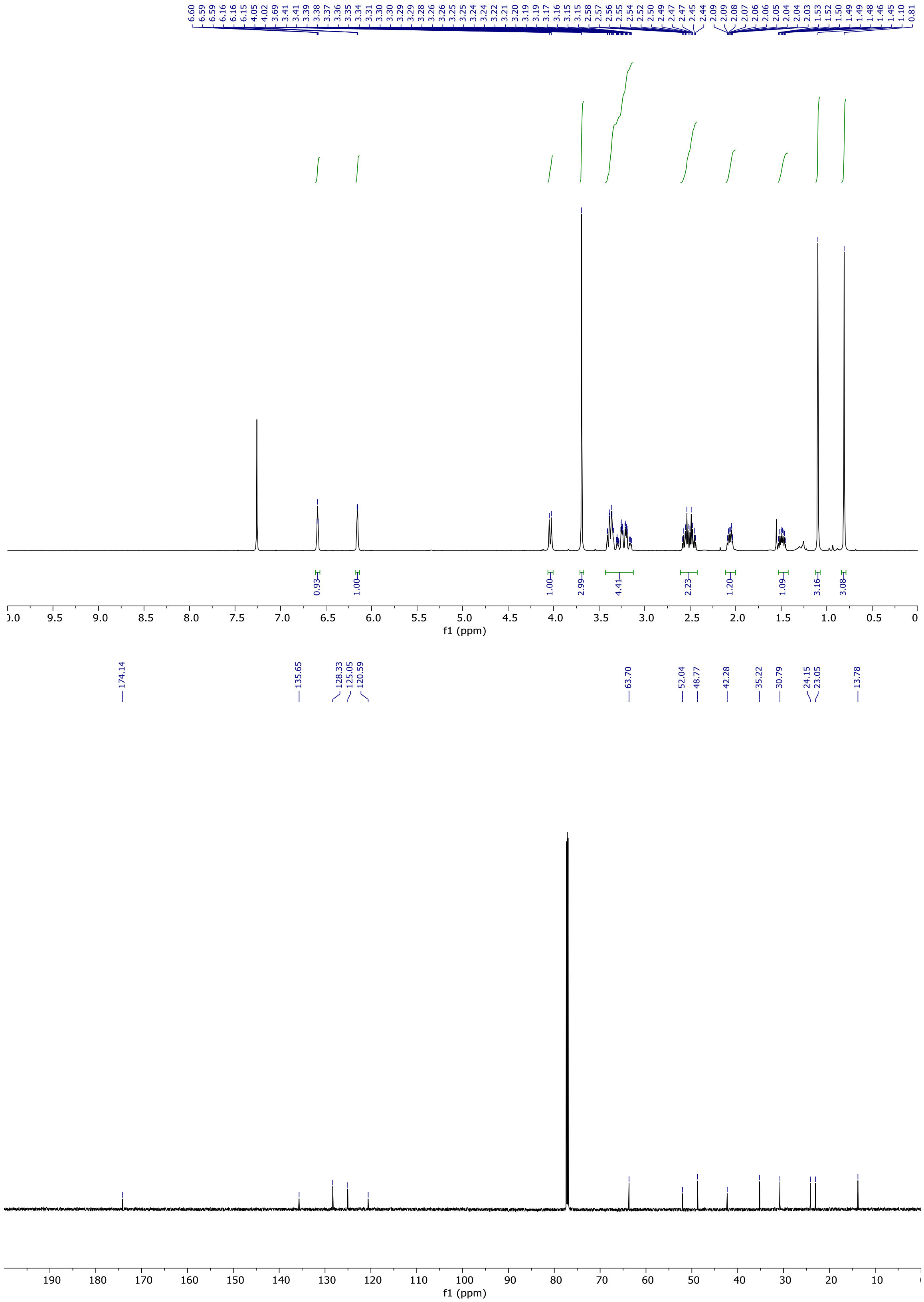

Supplementary Figure 68: ^{1}H (top) and ^{13}C NMR (bottom) for compound **3d** in $CDCl_3$

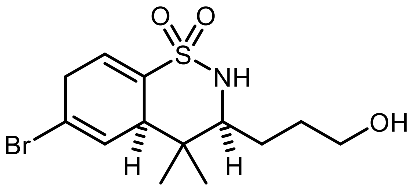

Amide reduction (4d): A stirred suspension of $LiAlH_4$ (3.8 mg, 100 µmol, 1.0 equiv.) in 0.7 mL dry THF in an oven-dried 1 dram vial with a Teflon septum screw cap was cooled to -78 °C. To this suspension, a room temperature suspension of **2d** (34.2 mg, 99 µmol, 1.0 equiv) in 1.2 mL THF was added dropwise via syringe. The reaction was stirred under nitrogen for 1.5 hours, at which point it was allowed to warm to 0 °C. After 10 minutes, the reaction was allowed to warm to room temperature and was quenched by careful addition of several drops of saturated aqueous potassium sodium tartrate. The reaction was transferred to a separatory funnel with 10 mL EtOAc and washed with 30 mL saturated aqueous potassium sodium tartrate. The aqueous phase was washed with additional EtOAc (2x10 mL) and the combined organic phases were dried over $MgSO_4$, filtered, and concentrated to give a pale yellow residue. The residue was purified via flash chromatography on silica gel (CH_2Cl_2 to 40% acetone in CH_2Cl_2) to afford the product as a colorless solid. (18.9 mg, 54 µmol, 55%). R_f = 0.20 (9:1, CH_2Cl_2:acetone), one yellow spot, $KMnO_4$.

^{1}H NMR (500 MHz, $CDCl_3$) = δ 6.61 – 6.58 (m, 1H), 6.18 – 6.14 (m, 1H), 4.06 (d, *J* = 10.9 Hz, 1H), 3.80 – 3.63 (m, 2H), 3.43 – 3.34 (m, 2H), 3.27 (dddd, *J* = 23.3, 7.4, 3.1, 2.2 Hz, 1H), 3.18 (dddd, *J* = 23.3, 7.6, 4.0, 1.2 Hz, 1H), 1.94 – 1.79 (m, 2H), 1.72 – 1.60 (m, 1H), 1.33 (t, *J* = 5.0 Hz, 1H,), 1.29 – 1.16 (m, 2H), 1.08 (s, 3H), 0.79 (s, 3H).

^{13}C NMR (176 MHz, $CDCl_3$) = δ 135.7, 127.9, 125.2, 120.5, 63.8, 62.2, 48.9, 42.4, 35.2, 29.4, 25.7, 23.1, 13.8.

IR (neat) = 3491, 3062, 2934, 2851, 1416, 1315, 1228, 1156, 1055, 1032 cm^{-1}.

HRMS (ESI+) m/z calculated for $C_{13}H_{20}BrNO_3S$ $[M+H]^+$: 350.0420, found 350.0417.

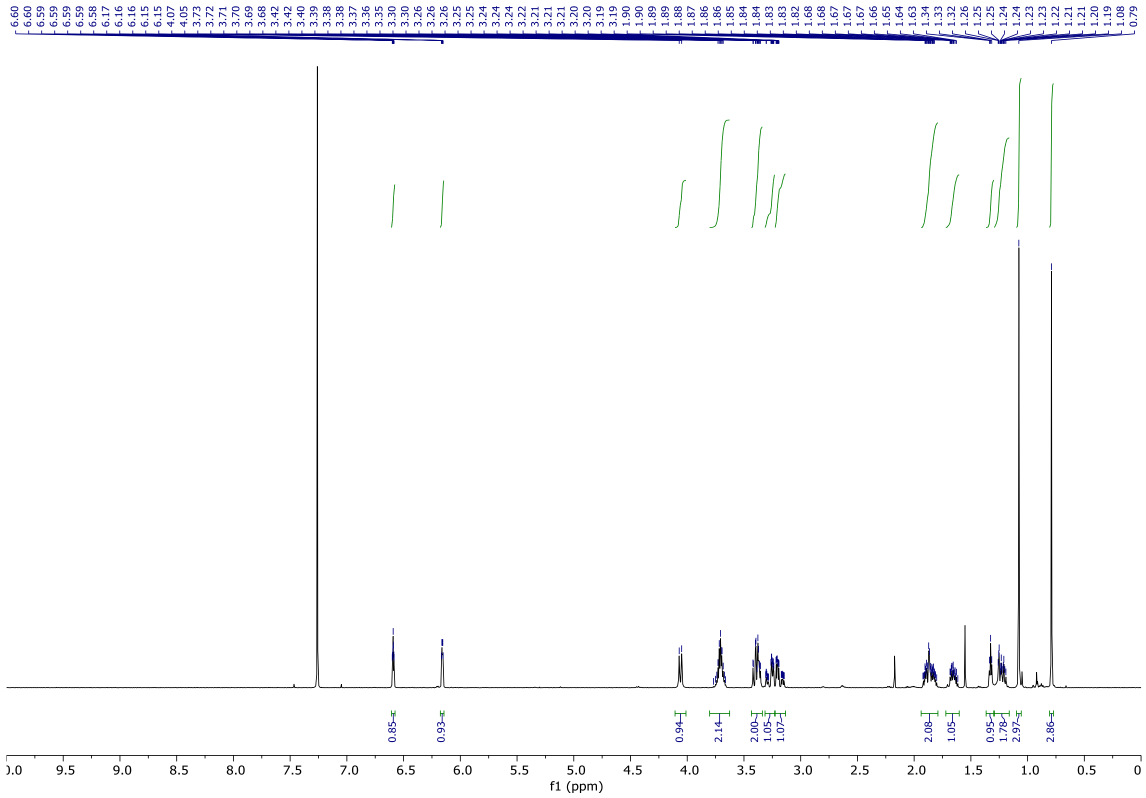

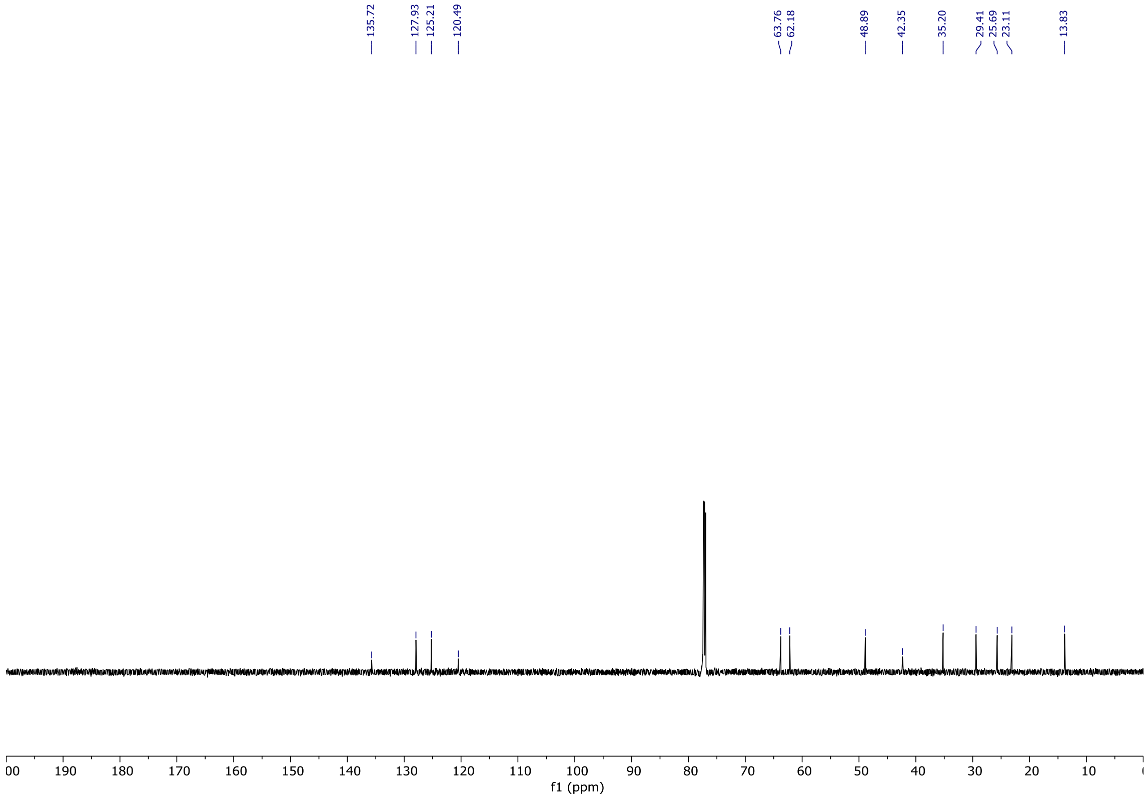

Supplementary Figure 69: ^{1}H (top) and ^{13}C NMR (bottom) for compound **4d** in $CDCl_3$

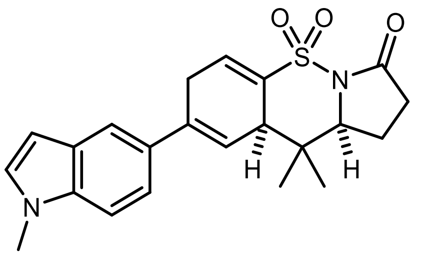

Alkenyl bromide cross-coupling (5d): To a vial containing flame-dried CsF (33 mg, 220 µmol, 3.0 equiv) and a magnetic stir bar were added **2d** (25 mg, 72 µmol, 1.0 equiv), (1-methylindol-5-yl)boronic acid (14 mg, 79 µmol, 1.1 equiv.), and triphenylphosphine (3.8 mg, 14 µmol, 0.20 equiv.). The vial was purged of oxygen by three sequential evacuation/N_2 back-fill cycles. In a separate flame-dried vial, $Pd(OAc)_2$ (1.6 mg, 7.2 µmol, 0.10 equiv.) was dissolved in 1.0 mL anhydrous THF. The palladium solution was degassed by three freeze-pump-thaw cycles, then added in one shot to the vial containing the solids. The reaction was heated to 66 °C for 14 hours with vigorous stirring. Upon cooling to room temperature, the contents of the vial were transferred to a separatory funnel with 10 mL CH_2Cl_2 and 10 mL water. The organic phase was isolated and the aqueous phase was washed with CH_2Cl_2 (3 x 10 mL). The combined organic phases were washed with sat. aq. NaCl, then dried over $MgSO_4$, filtered, and concentrated under reduced pressure. The residue was purified *via* flash chromatography on silica gel (5 to 50% EtOAc in hexanes) to give the product as a colorless solid (8.3 mg, 21 µmol, 29%). R_f = 0.19 (1:1 Hex:EtOAc), one yellow spot, $KMnO_4$.

^{1}H NMR (700 MHz, $CDCl_3$) = δ 7.64 (s, 1H), 7.33 – 7.28 (m, 2H), 7.14 (t, *J* = 3.3 Hz, 1H), 7.08 (d, *J* = 3.0 Hz, 1H), 6.50 (d, *J* = 3.0 Hz, 1H), 6.11 – 6.08 (m, 1H), 4.18 (dd, *J* = 8.4, 5.9 Hz, 1H), 3.81 (s, 3H), 3.56 – 3.32 (m, 3H), 2.56 – 2.45 (m, 2H), 2.21 – 2.15 (m, 1H), 1.97 – 1.90 (m, 1H), 1.17 (s, 3H), 0.83 (s, 3H).

^{13}C NMR (176 MHz, $CDCl_3$) = δ 173.2, 137.0, 136.7, 134.6, 134.2, 131.4, 129.9, 128.7, 119.4, 117.8, 117.8, 109.4, 101.5, 67.6, 46.7, 42.0, 33.1, 31.2, 29.6, 22.4, 19.3, 13.8.

IR (neat) = 2956, 2930, 2855, 1743, 1492, 1348, 1335, 1161, 1091, 978 cm^{-1}.

HRMS (ESI+) m/z calculated for $C_{22}H_{24}N_2O_3S$ $[M+H]^+$: 397.1580, found 397.1572.

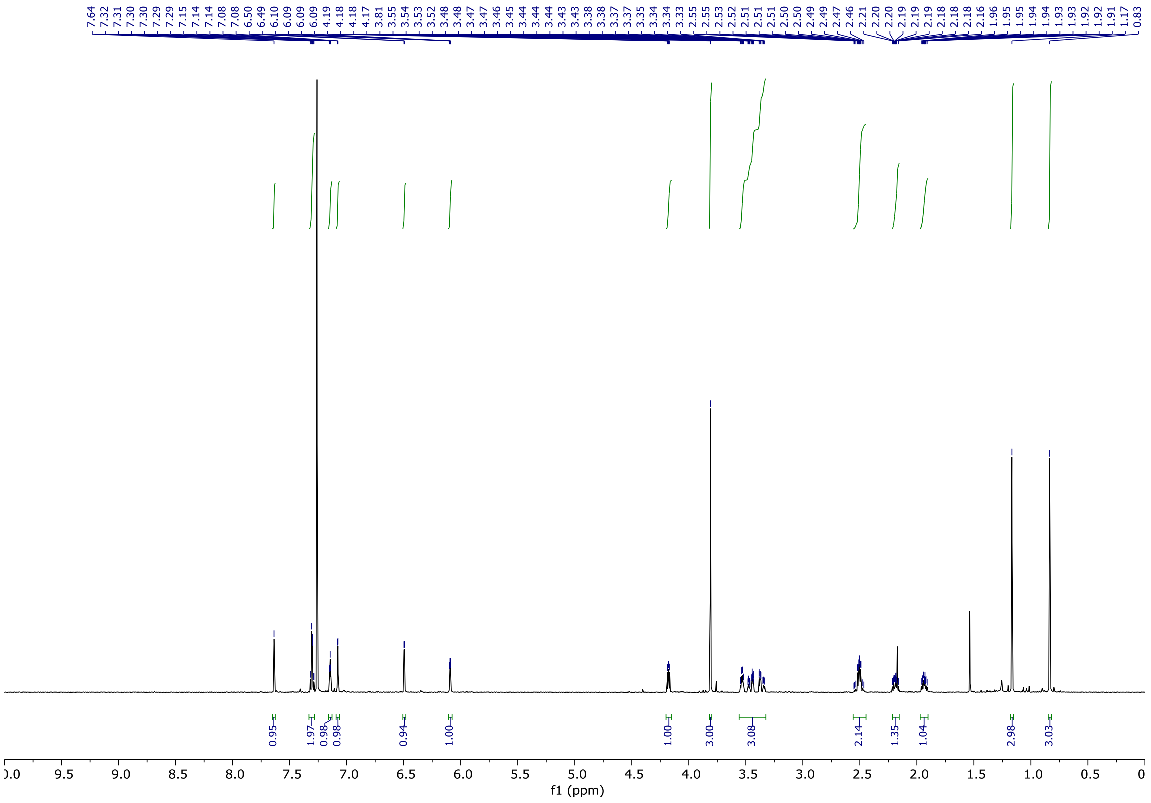

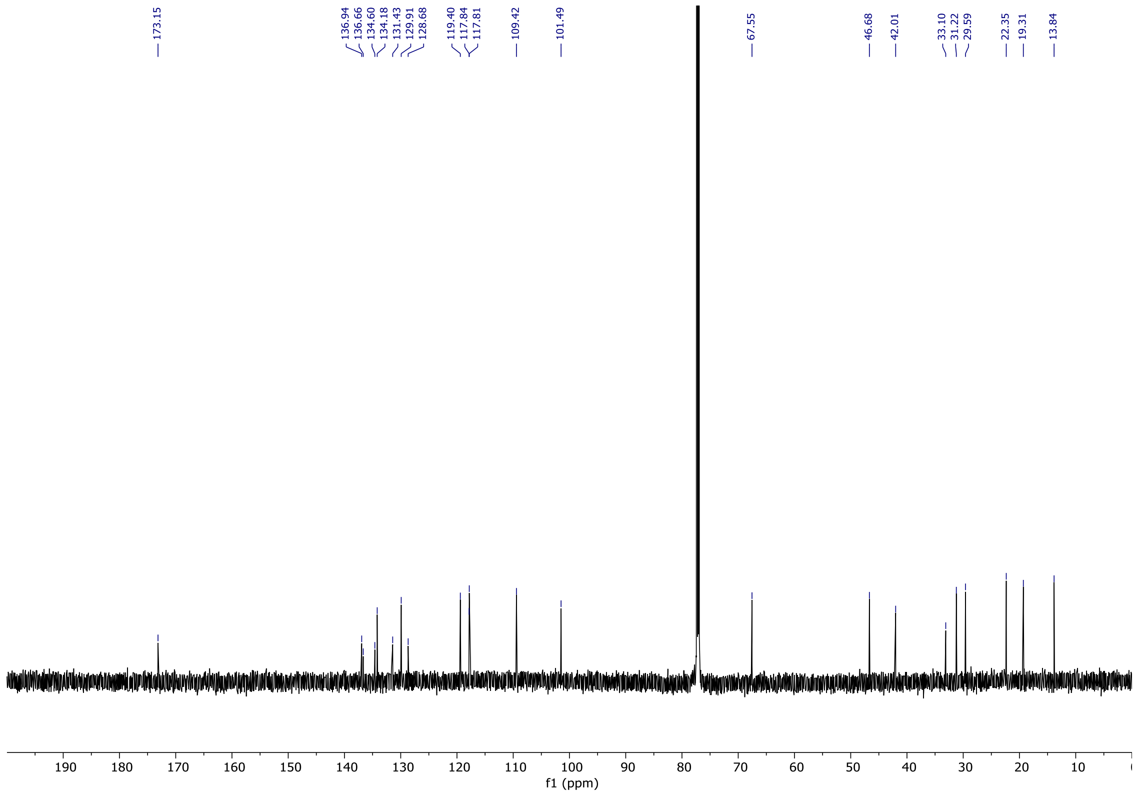

Supplementary Figure 70: ^{1}H (top) and ^{13}C NMR (bottom) for compound **5d** in $CDCl_3$

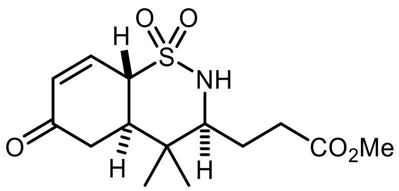

Amide, ester alcoholysis (3s): Under ambient atmosphere at room temperature, K_2CO_3 (19.5 mg, 141 µmol, 5.0 equiv) was added in one portion to a stirred suspension of **2s** (11.1 mg, 28.0 µmol, 1.0 equiv.) in 1 mL anhydrous MeOH. The reaction was stirred vigorously for 15 minutes before quenching by transfer into a separatory funnel containing 30 mL sat. aq. NH_4Cl. The reaction vial was rinsed with 5 mL additional water and 10 mL CH_2Cl_2. The aqueous phase was further washed with CH_2Cl_2 (2 x 10 mL) and the combined organic phases were dried over $MgSO_4$, filtered, and concentrated to give a pale yellow residue. The residue was purified via flash chromatography on silica gel (CH_2Cl_2 to 10% acetone in CH_2Cl_2, then isocratic hold) to afford the product as a colorless solid as a single diasteromer. (6.3 mg, 20. µmol, 71%). R_f = 0.25 (9:1, CH_2Cl_2:acetone), one yellow spot, $KMnO_4$.

^{1}H NMR (500 MHz, $CDCl_3$) = δ 6.61 – 6.58 (m, 1H), 6.18 – 6.14 (m, 1H), 4.06 (d, *J* = 10.9 Hz, 1H), 3.80 – 3.63 (m, 2H), 3.43 – 3.34 (m, 2H), 3.27 (dddd, *J* = 23.3, 7.4, 3.1, 2.2 Hz, 1H), 3.18 (dddd, *J* = 23.3, 7.6, 4.0, 1.2 Hz, 1H), 1.94 – 1.79 (m, 2H), 1.72 – 1.60 (m, 1H), 1.33 (t, *J* = 5.0 Hz, 1H), 1.29 – 1.16 (m, 2H), 1.08 (s, 3H), 0.79 (s, 3H).

^{13}C NMR (176 MHz, $CDCl_3$) = δ 196.7, 174.3, 139.6, 132.2, 64.6, 59.2, 52.2, 47.3, 37.6, 37.0, 30.9, 23.9, 23.0, 13.3.

IR (neat) = 3231, 2976, 2686, 1733, 1678, 1466, 1423, 1309, 1248, 1166 cm^{-1}.

HRMS (ESI+) m/z calculated for $C_{14}H_{21}NO_5S$ $[M+H]^+$: 316.1213, found 316.1201.

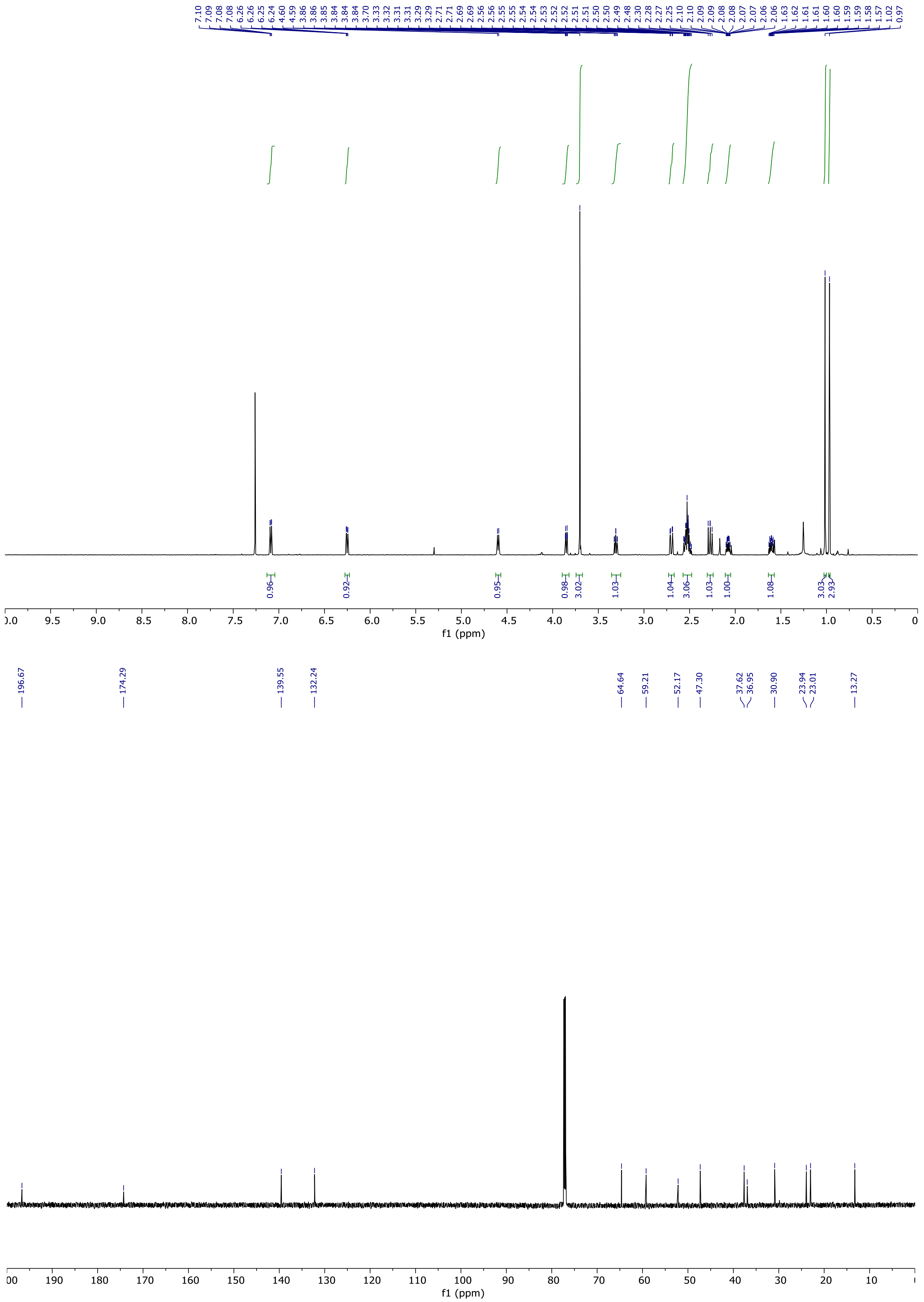

Supplementary Figure 71: ^{1}H (top) and ^{13}C NMR (bottom) for compound **3s** in $CDCl_3$

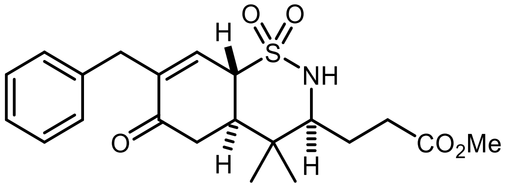

Enone α-alkylation (4s): In a flame dried vial with a stir bar, enone **3s** (10.0 mg, 32 µmol, 1 equiv.) was dissolved in 0.5 mL DMF. K_2CO_3 (6.6 mg, 48 µmol, 1.5 equiv) was added in one portion under nitrogen, followed by benzyl bromide via microsyringe (4.0 µL, 34 µmol, 1.06 equiv.). The reaction was stirred at room temperature for 2 hours, during which time a color change was observed from clear colorless to clear yellow. The reaction was transferred to a separatory funnel with 10 mL of diethyl ether and 10 mL water. The organic phase was isolated and the aqueous phase was washed with diethyl ether (3 x 10 mL). The combined organic phases were washed with 5 wt% aqueous LiCl, then dried over $MgSO_4$, filtered, and concentrated under reduced pressure. The residue was purified using flash chromatography on silica gel (0 to 10% acetone in CH_2Cl_2) to obtain the title product as a colorless solid and single diasteromer. (8.0 mg, 20 µmol, 62%). R_f = 0.33 (1:1 Hex:EtOAc), one yellow spot, $KMnO_4$.

^{1}H NMR (500 MHz, $CDCl_3$) δ 7.31 – 7.25 (m, 2H), 7.24 – 7.15 (m, 3H), 6.74 (s, 1H), 4.45 (d, *J* = 10.3 Hz, 1H), 3.88 – 3.81 (m, 1H), 3.70 (s, 3H), 3.64 (d, *J* = 15.2 Hz, 1H), 3.51 (d, *J* = 15.3 Hz, 1H), 3.29 (ddd, *J* = 12.8, 11.6, 2.4 Hz, 1H), 2.71 (dd, *J* = 16.0, 3.4 Hz, 1H), 2.58 – 2.44 (m, 3H), 2.27 (dd, *J* = 15.8, 14.4 Hz, 1H), 2.06 (dtd, *J* = 14.3, 7.1, 2.6 Hz, 1H), 1.62 – 1.51 (m, 1H), 0.99 (s, 3H), 0.93 (s, 3H).

^{13}C NMR (176 MHz, $CDCl_3$) = δ 196.2, 174.3, 142.7, 138.1, 135.1, 129.2, 128.8, 126.7, 64.6, 59.7, 52.1, 47.4, 37.9, 36.7, 35.3, 30.9, 24.0, 23.1, 13.3.

IR (neat) = 3250, 2973, 1731, 1677, 1495, 1420, 1379, 1330, 1264, 1156 cm^{-1}.

HRMS (ESI+) m/z calculated for $C_{21}H_{27}NO_5S$ $(M+NH_4)^+$: 423.1948, found 423.1932.

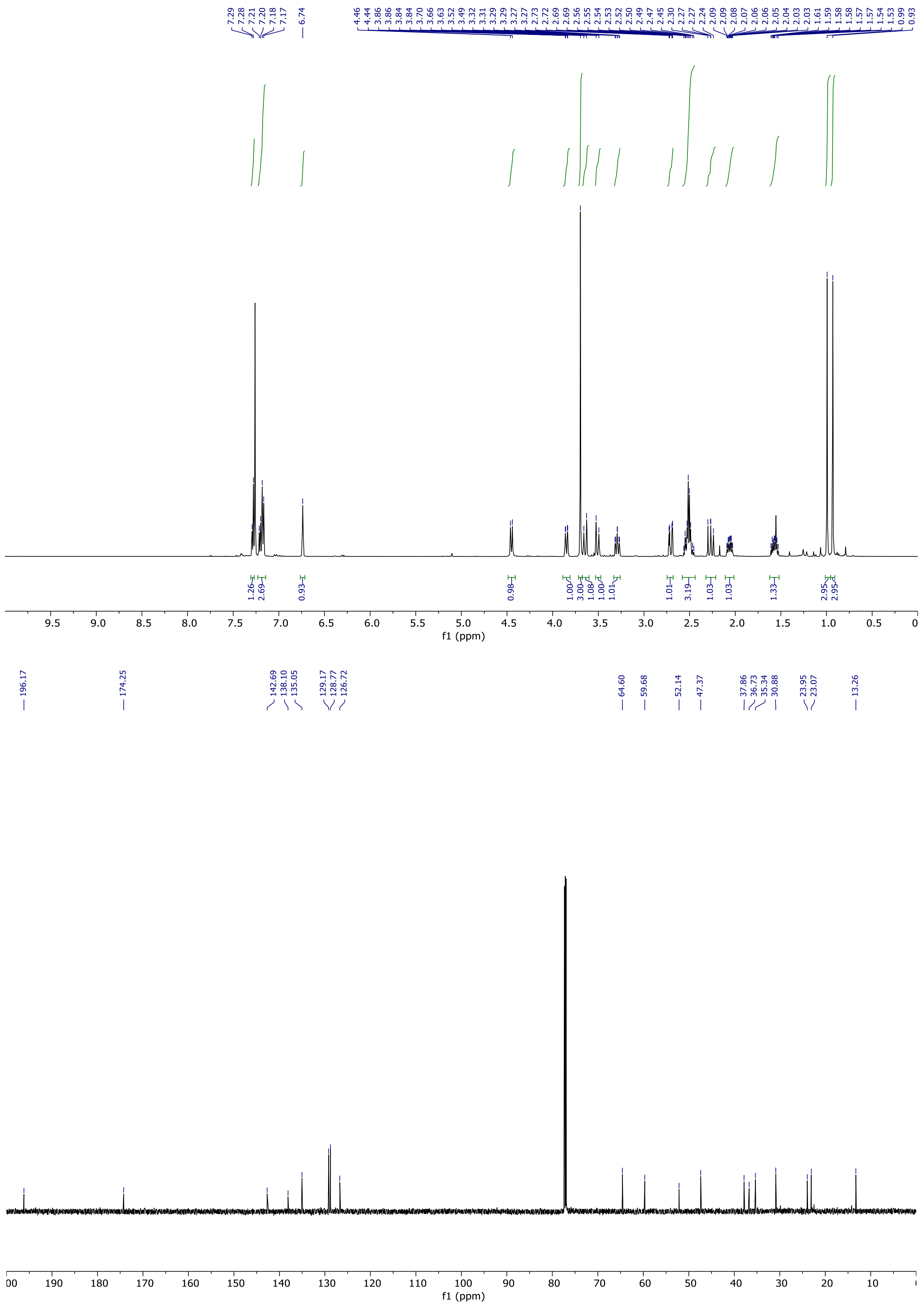

Supplementary Figure 72: ^{1}H (top) and ^{13}C NMR (bottom) for compound **4s** in $CDCl_3$

Stern – Volmer Fluorescence Quenching: Fluorescence quenching experiments were conducted on a Horiba PTI QuantaMaster 8000 using FelixGX software. Samples were prepared in dichloromethane (CH_2Cl_2 is a successful solvent for this reaction) due to the poor solubility of the photocatalyst in 1:1 trifluorotoluene:^{t}BuOH. Each sample was degassed in the sealed septum screw-capped cuvette by sparging with argon for 30 seconds immediately prior to each measurement. The solutions were irradiated at 420 nm and luminescence was measured at 593 nm. I_o/I values were generated from the average of three scans taken per quencher concentration. Solutions of a given concentration were produced and measured in triplicate (triplicate of triplicates).

Substrate / sulfonyl enamide used in fluorescence quenching and cyclic voltammetry studies:

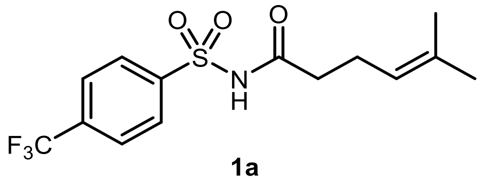

1a

Stern – Volmer Fluorescence Quenching Data Analysis: The sulfonyl enamide substrate does not quench the excited state of the photocatalyst. Additionally, when the substrate is present in solution with tetrabutylammonium dibutyl phosphate, there is no additional quenching of the excited state of the photocatalyst beyond the contribution from the phosphate base by itself. This data suggests that neither direct oxidation of the substrate by the photocatalyst nor oxidative multiple-site concerted proton- electron transfer (MS-CPET) of a sulfonyl amide-phosphate complex is primarily responsible for *N*-radical formation and initiation of the cyclization cascade.

[Photocatalyst] = 0.2 mM

Without base		With 0.6 mM base	
[substrate] (mM)	Average I_o/I	[substrate] (mM)	Average I_o/I
0.15	1.071446586	0.15	1.945956
0.3	1.083896878	0.3	1.892258
0.6	1.100651471	0.6	1.859189
1.2	1.074968438	1.2	1.899883

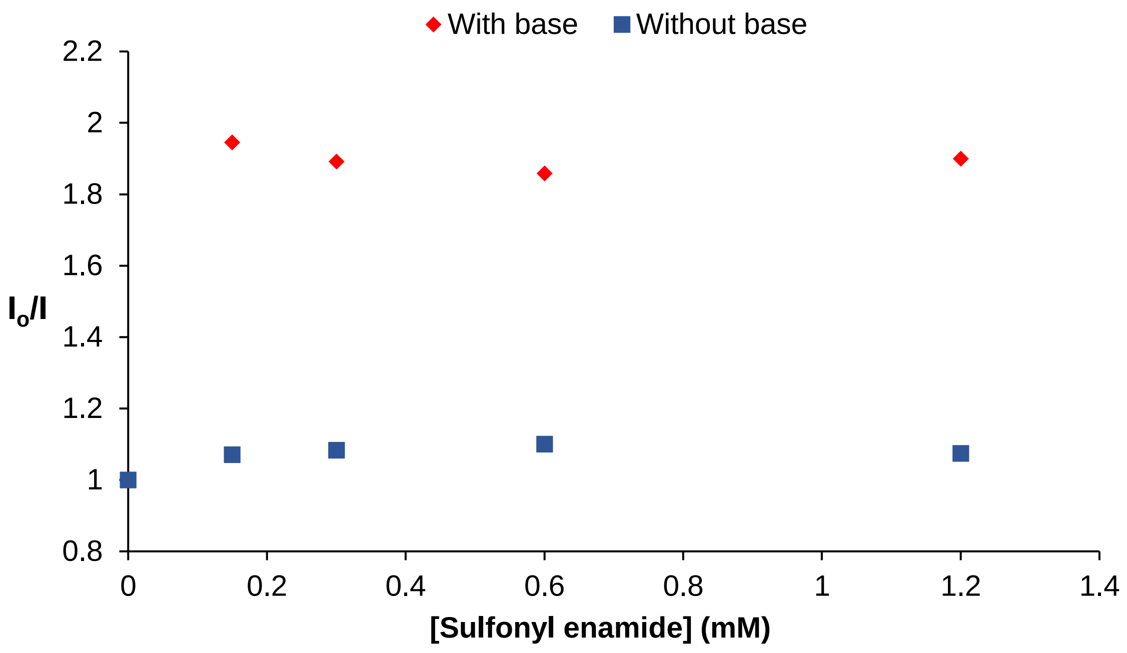

Supplementary Figure 73: Irradiation of solutions containing only **1a** and photocatalyst (square data points) demonstrates no quenching of the excited state of the photocatalyst by the sulfonyl enamide substrate. Titration experiments wherein substrate was added to a solution with constant base concentration also showed no change in quenching.

Experiment A: Constant [Ir], fixed substrate:phosphate ratio, variable [substrate:phosphate]

[Photocatalyst] = 0.05 mM, [phosphate] = 0.65*[substrate]

[substrate] mM	Avg I_o/I
0	1
0.05	1.163948
0.1	1.311931
0.15	1.389046
0.2	1.489546
0.25	1.501165

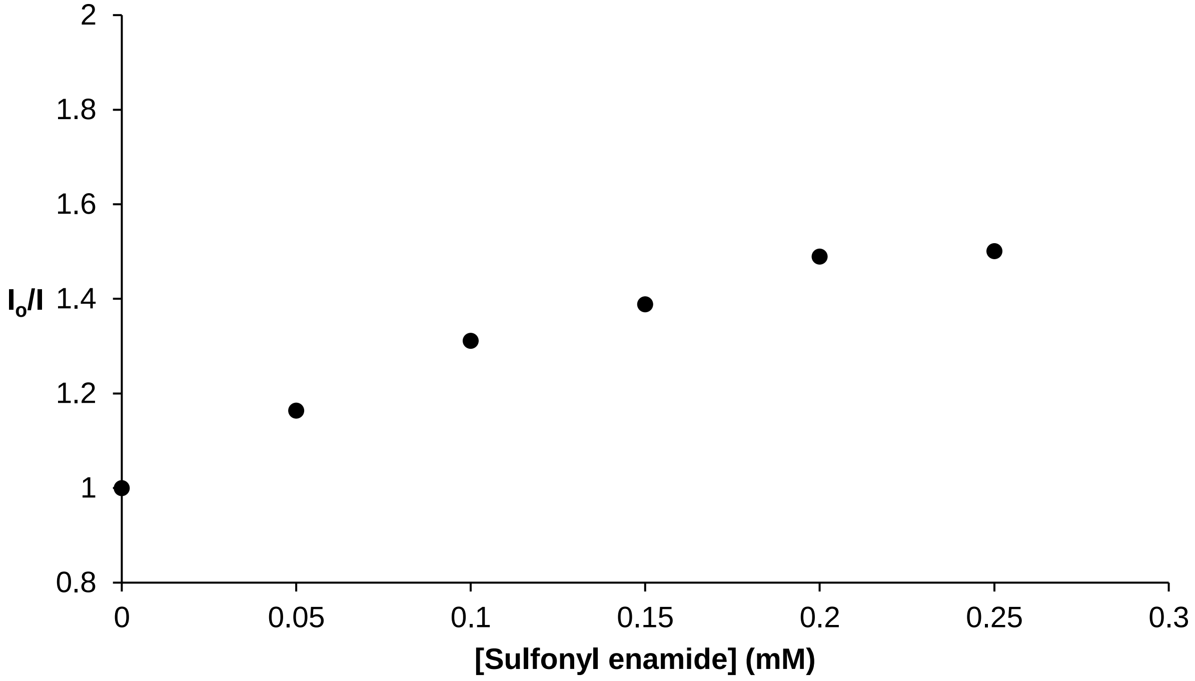

Supplementary Figure 74: Experiment A containing an increasing concentration of the substrate/base mixture demonstrate some quenching of the excited state of the photocatalyst.

Experiment B: Constant [Ir], variable [phosphate], no substrate

[Photocatalyst] = 0.05 mM, [phosphate] = 0.65*(theoretical [substrate])

Theoretical [substrate] (mM)	Average I_o/I
0	1
0.05	1.242801
0.1	1.36209
0.15	1.460931
0.2	1.523735
0.25	1.618825

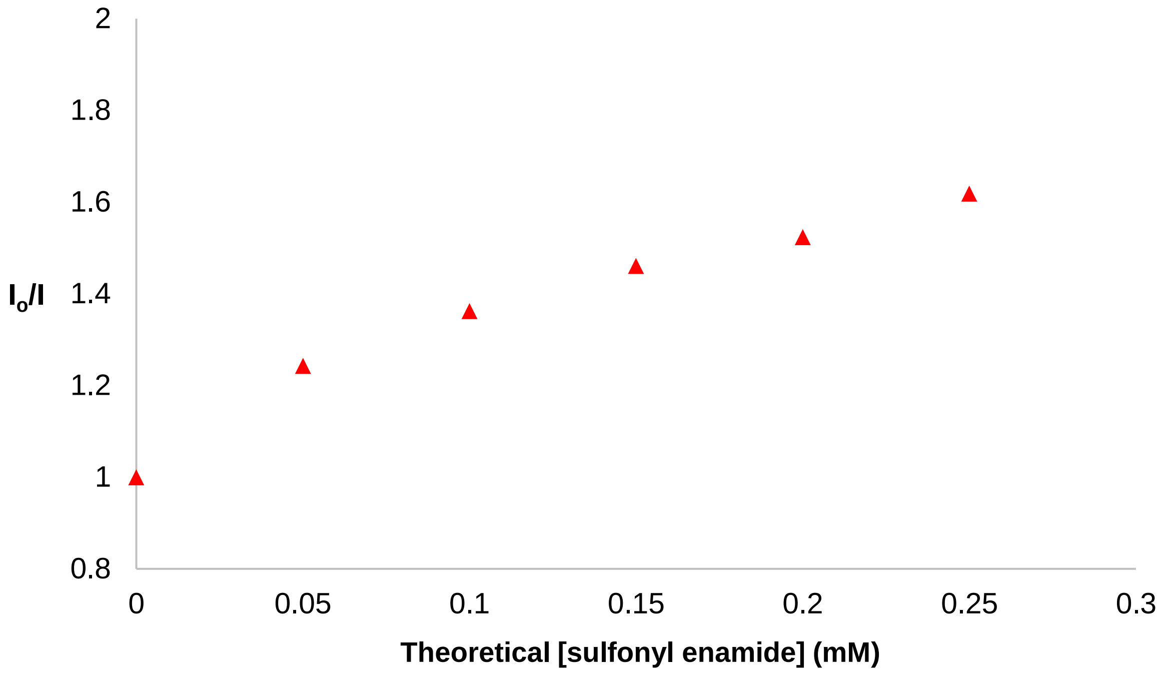

Supplementary Figure 75: Experiment B containing only dibutyl phosphate base and the photocatalyst exhibits some quenching of the excited state of the photocatalyst.

We wish to make a direct comparison of the luminescence quenching between experiment A, where the base is present in a fixed ratio to the sulfonamide; and experiment B, where the sulfonamide is absent, but the concentrations of the base are the same as those in experiment A. For this reason, experiment B is plotted such that the *x*-axis will remain the same when the plots of the two experiments are superimposed.

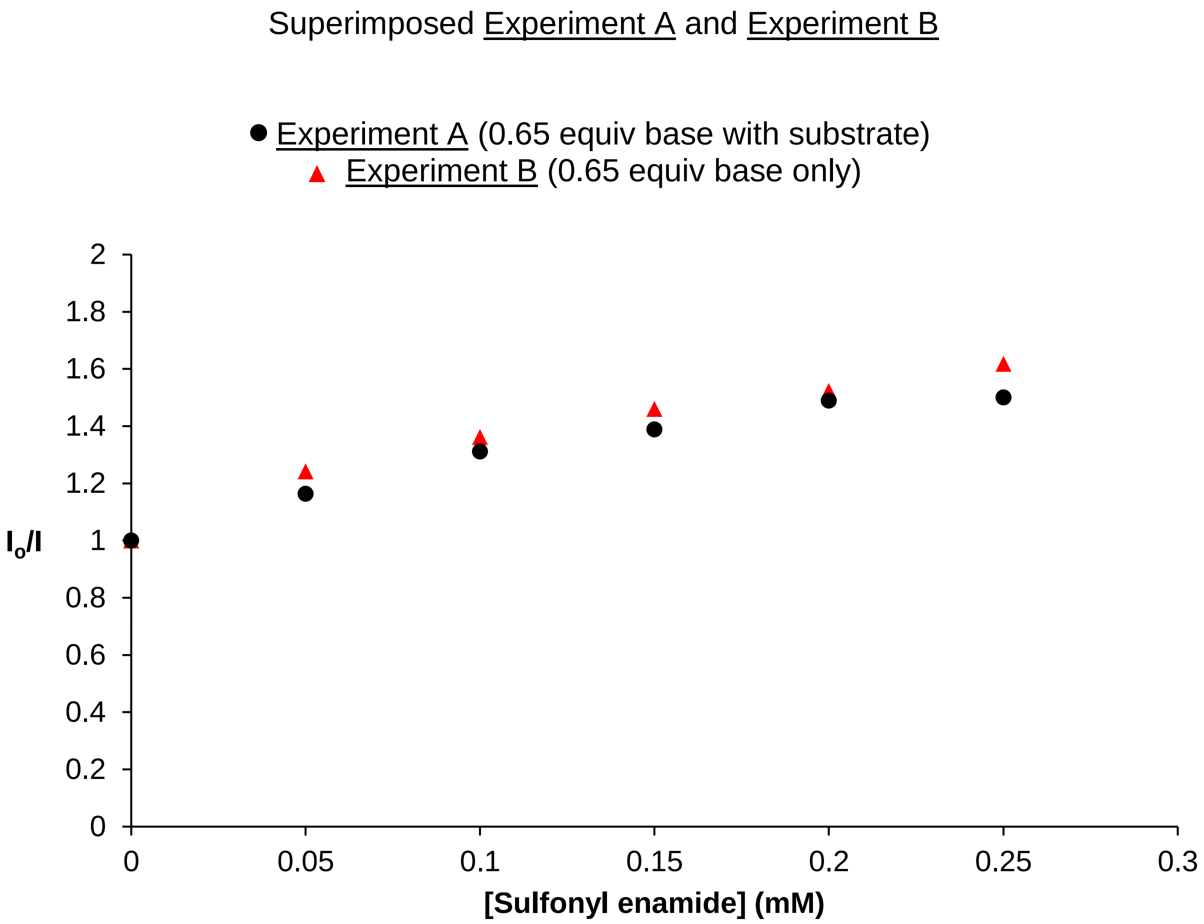

Supplementary Figure 76: Experiments A and B superimposed on the same graph.

Cyclic voltammetry. Cyclic voltammetry was performed using a CHI620E electrochemical analyzer (from CH Instruments) and was performed with a three-electrode set-up, using a glassy carbon working electrode (3 mm diameter), a graphite counter electrode and a silver/silver ion nonaqueous reference electrode. All experiments were conducted in dichloromethane with 0.1 M NBu_4PF_6.

In all experiments containing the substrate, the concentration of the substrate was 10 mM. In the control containing no substrate, the tetrabutylammonium dibutylphosphate base concentration is 10 mM.

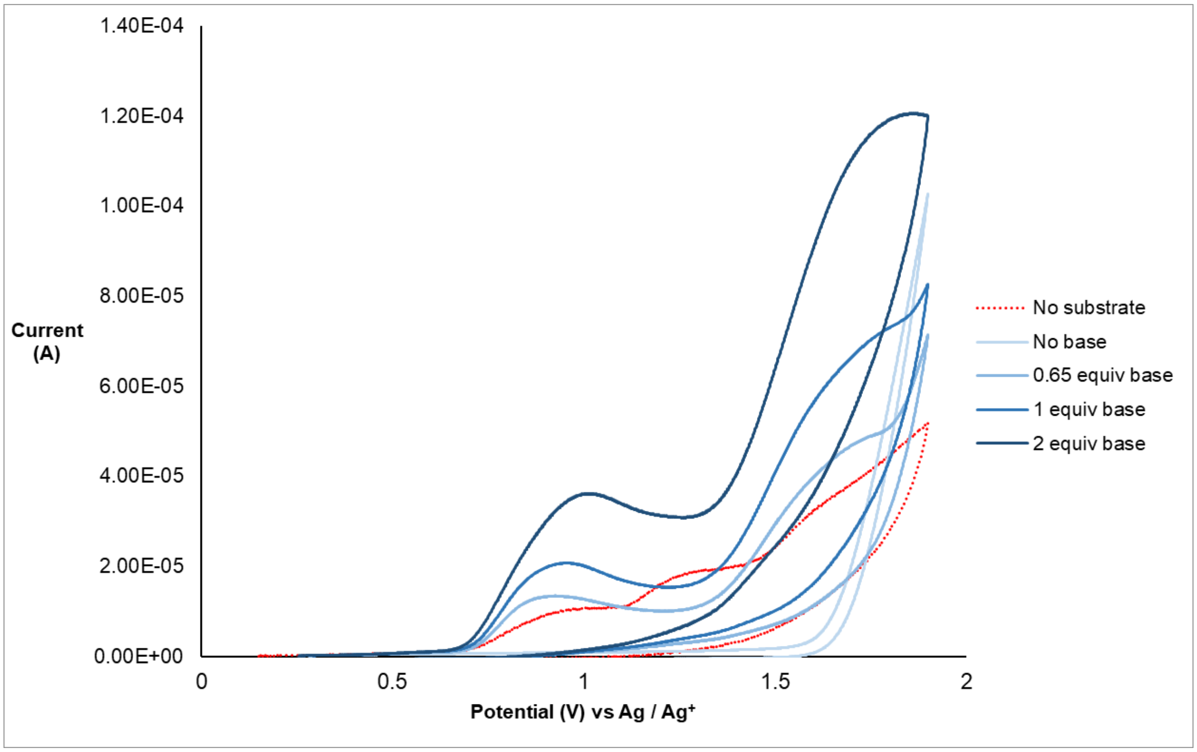

Supplementary Figure 77: Superimposition of all base titration voltammograms**.**

The oxidation of the substrate occurs at a potential >1.6 V vs Ag/Ag$^+$. This result suggests that direct oxidation and subsequent deprotonation of the substrate (1a) is an unlikely process. A solution of the tetrabutylammonium dibutylphosphate base alone exhibits an oxidation wave at ~1 V vs Ag/Ag$^+$. As the concentration of tetrabutylammonium dibutylphosphate base increases relative to substrate **1a**, the current response increases for the oxidation wave ~1 V vs Ag/Ag$^+$. These data seem to suggest that as the tetrabutylammonium dibutylphosphate base is oxidatively active at oxidation potentials attainable by photocatalyst **A** and that the base may be the species being oxidized (to phosphate radical) in solution and thus initiating the *N*-radical cascade process. Importantly, there is no obvious oxidation potential shift (to less positive potential) for the substrate **1a** as one would expect for a MS-CPET process.

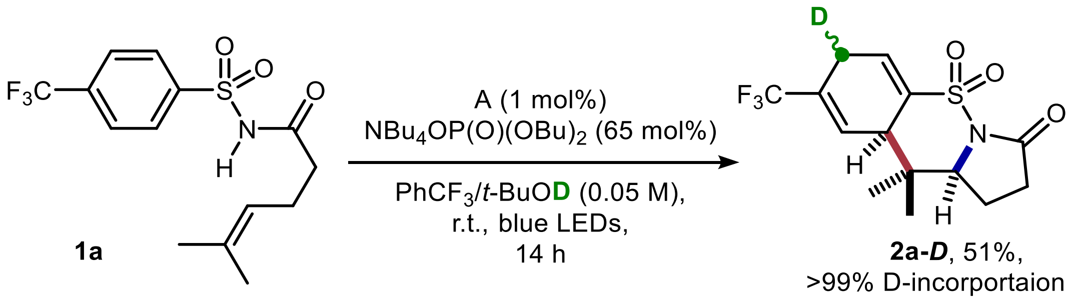

D*-C8-trifluoromethyl cyclohexadiene-fused sultam (2a-*D*)**: To an oven dried 2-dram vial was added substrate **1a** (67.1 mg, 0.2 mmol), $NBu_4OP(O)(OBu)_2$ (58 mg, 0.13 mmol, 0.65 equiv), and photocatalyst **A** (2 mg, 1 mol%). The vial contents were then dissolved in a 1:1 mixture of *t*-BuOD:$PhCF_3$ (2 mL each, 0.05 M). The reaction solution was degassed by sparging with argon for 15 min. Then the vial was quickly capped and sealed with parafilm. The reaction was irradiated with two, H150 blue Kessil lamps positioned ~5 cm away and cooled with an overhead fan. After 14 h, the reaction was directly concentrated *in vacuo.* The resultant residue was subjected to flash column chromatography over silica providing the pure diene **2a-*D. Off-white, tan powder (33.4 mg, 51% yield). R_f = 0.3 (1:1, Hex:EtOAc), one yellow spot, $KMnO_4$, UV.

^{1}H NMR (700 MHz, $CDCl_3$) = δ 7.02 (d, *J* = 2.9 Hz, 1H), 6.38 (s, 1H), 4.16 (dd, *J* = 8.5, 5.8 Hz, 1H), 3.48 (s, 1H), 3.17 – 2.99 (m, 1H), 2.60 – 2.43 (m, 2H), 2.20 (dddd, *J* = 13.7, 10.3, 8.6, 6.6 Hz, 1H), 1.99 – 1.83 (m, 1H), 1.14 (s, 3H), 0.78 (s, 3H) ppm

^{13}C NMR (176 MHz, $CDCl_3$) = δ 172.7, 134.3, 131.3, 127.8 (q, *J* = 31.2 Hz), 125.9 (q, *J* = 5.6 Hz), 122.8 (q, *J* = 272.0 Hz), 67.0, 45.3, 41.4, 30.9, 24.3 – 22.8 (m), 22.0, 19.0, 13.8 ppm

^{19}F NMR (377 MHz, $CDCl_3$) = δ -69.85 ppm

IR (*neat*) = 2982, 1738, 1473, 1344, 1299, 1168, 1122, 986, 897, 700 cm^{-1}

HRMS (ESI+) *m/z* calculated for $C_{14}H_{15}DF_3NO_3S$ [M+H]+ 337.0939, found 337.0943.

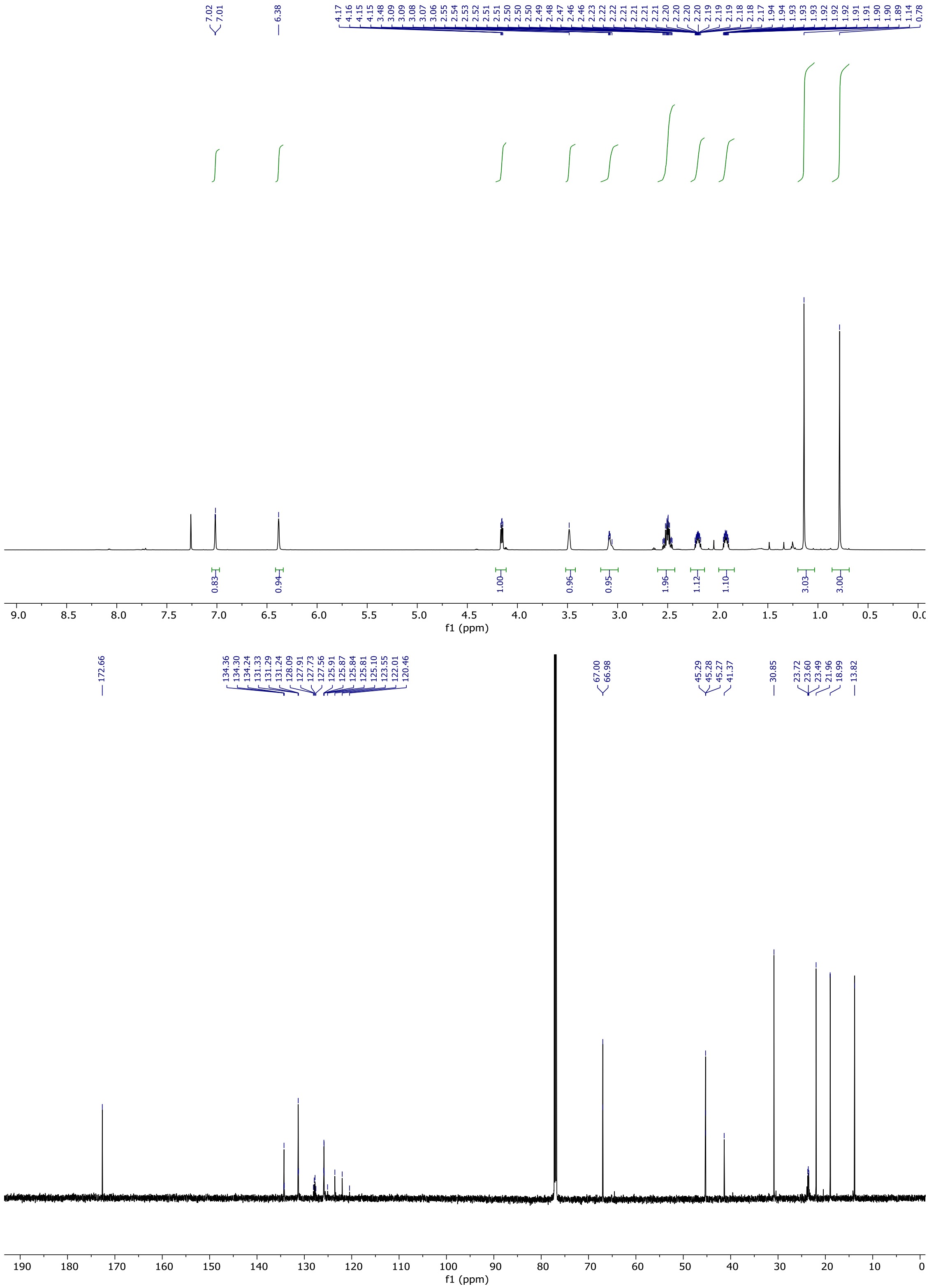

Supplementary Figure 78: ^{1}H (top) and ^{13}C NMR (bottom) for compound **2a-*D*** in $CDCl_3$

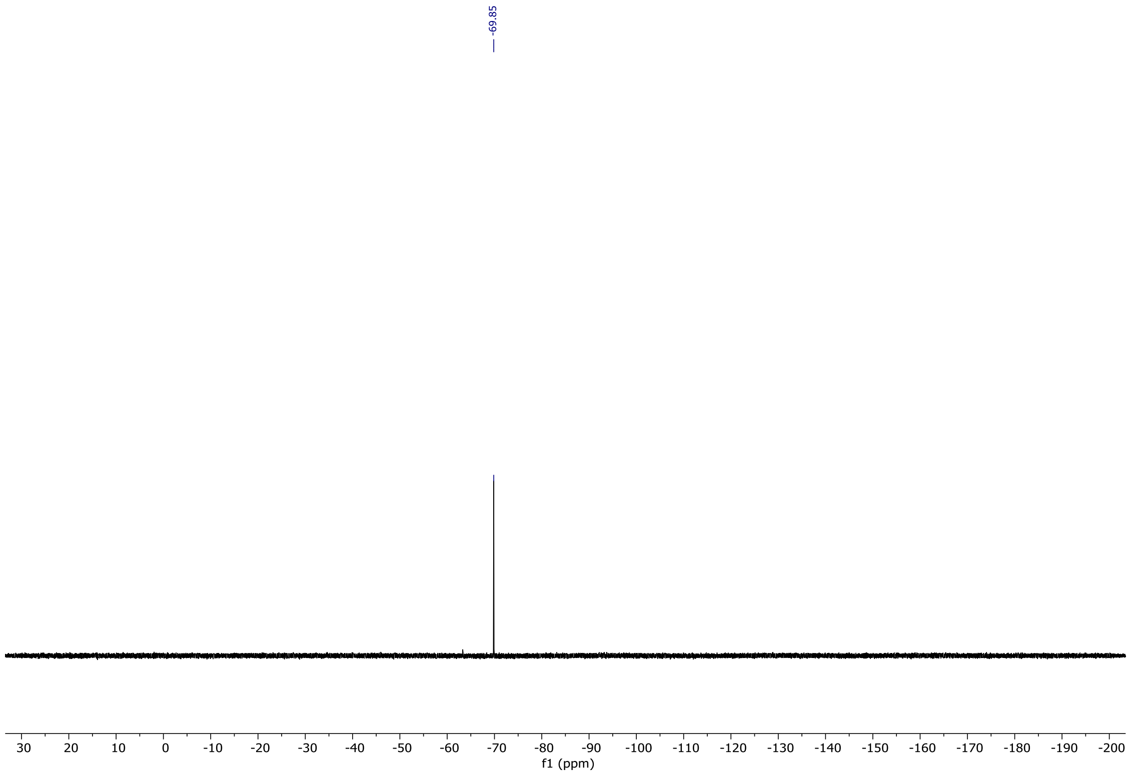

Supplementary Figure 79: ^{19}F NMR for compound **2a-*D*** in $CDCl_3$

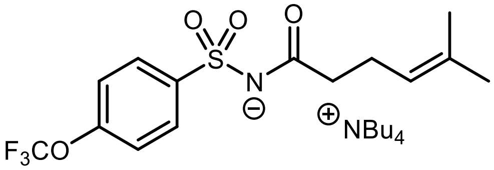

Arylsulfonamidyl anion salt [NBu_4][1g]: To a flame-dried round-bottom flask with a stir bar was added sodium hydride (60 wt% dispersion in mineral oil, 22.9 mg, 0.598 mmol, 1.05 equiv). The flask was evacuated and backfilled with nitrogen three times before pentane (1 mL) was added via syringe. The flask was gently swirled and the pentane/mineral oil solution was removed via syringe, taking care not to remove any sodium hydride. This process was repeated twice more to give the sodium hydride as a free-flowing powder. Anhydrous THF (4 mL) was then added via syringe, and the resulting suspension was cooled to 0 °C in an ice bath. To this suspension was added dropwise a solution of **1g** (200. mg, 0.569 mmol, 1.00 equiv). in 1 mL anhydrous THF. The resulting clear solution was stirred at 0 °C for ten minutes, then removed from the ice bath and allowed to warm to room temperature. NBu_4Cl (158 mg, 0.569 mmol, 1.00 equiv) was added as a fine suspension in 5 mL anhydrous THF. The cloudy solution was stirred for 1 hour at room temperature. The contents of the reaction were filtered through a 1 cm pad of Celite on a medium porosity frit. The Celite was washed with 5 mL additional THF, 5 mL CH_2Cl_2, and the filtrate was concentrated to give a pale yellow oil. This oil was dried under vacuum at 50 °C for 2 days to give the product as an immobile pale yellow oil that eventually solidified to a colorless solid in freezer storage (298 mg, 0.502 mmol, 88% yield).

Partial characterization is provided below:

^{1}H NMR (400 MHz, DMSO-d_6) = δ 7.85 – 7.78 (m, 2H), 7.36 – 7.30 (m, 2H), 5.04 – 4.96 (m, 1H), 3.21 – 3.12 (m, 8H), 2.03 (q, *J* = 7.3 Hz, 2H), 1.93 – 1.84 (m, 2H), 1.63 – 1.51 (m, 11H), 1.50 (s, 3H), 1.30 (sext, *J* = 7.3 Hz, 8H), 0.93 (t, *J* = 7.3 Hz, 12H).

^{13}C NMR (176 MHz, DMSO-d_6) δ 177.10, 148.75, 145.91, 129.93, 129.00, 124.71, 119.99 (q, J = 256 Hz), 119.81, 57.51, 39.33, 25.44, 24.56, 23.04, 19.19, 17.41, 13.46.

^{19}F NMR (377 MHz, DMSO-d_6) = δ -56.72.

HRMS (ESI-) *m/z* calculated for $C_{14}H_{15}F_3NO_4S^-$ (M): 350.0674, found 350.00675.

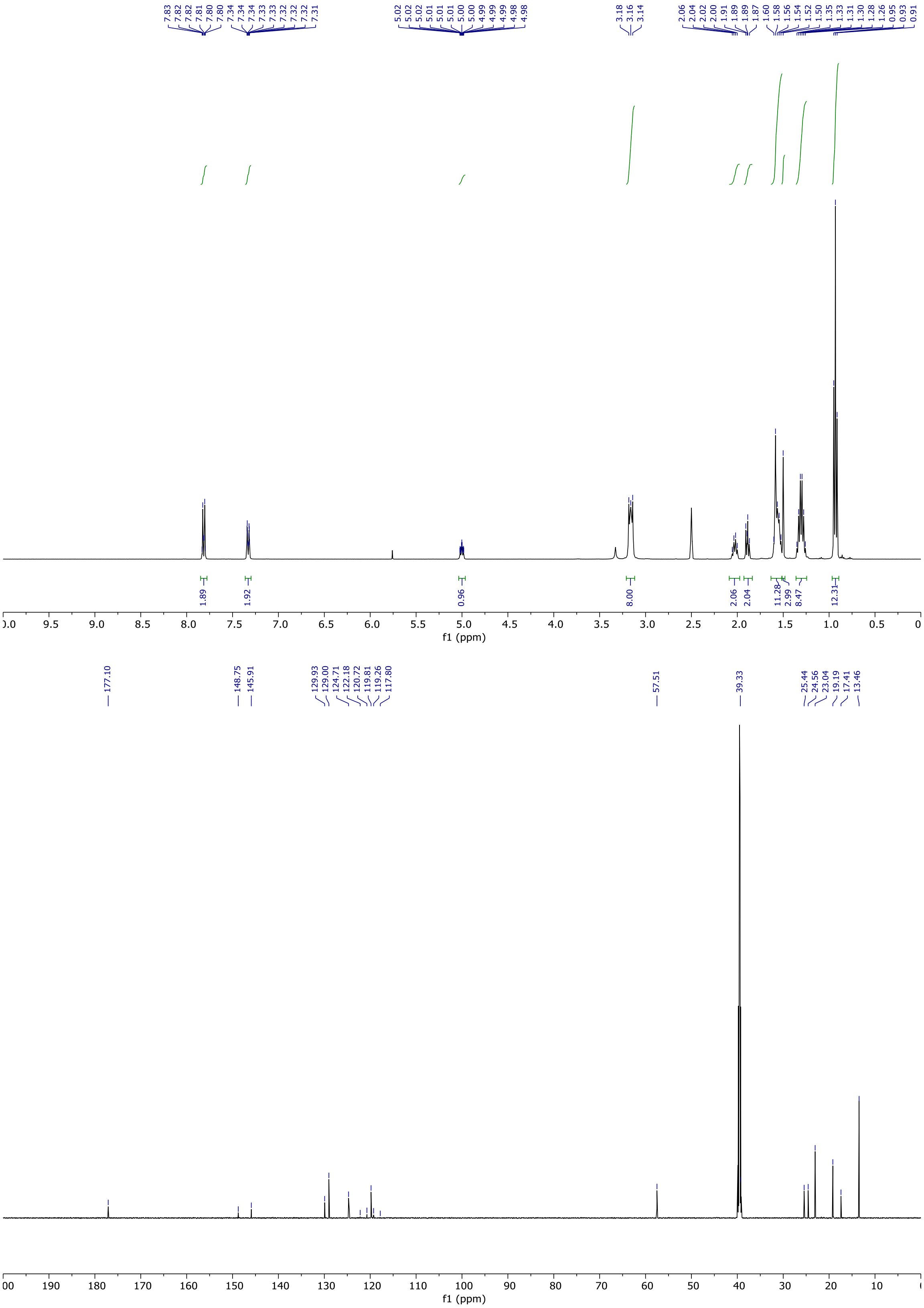

Supplementary Figure 80: ^{1}H (top) and ^{13}C NMR (bottom) for compound **[NBu$_4$][1g]** in $CDCl_3$

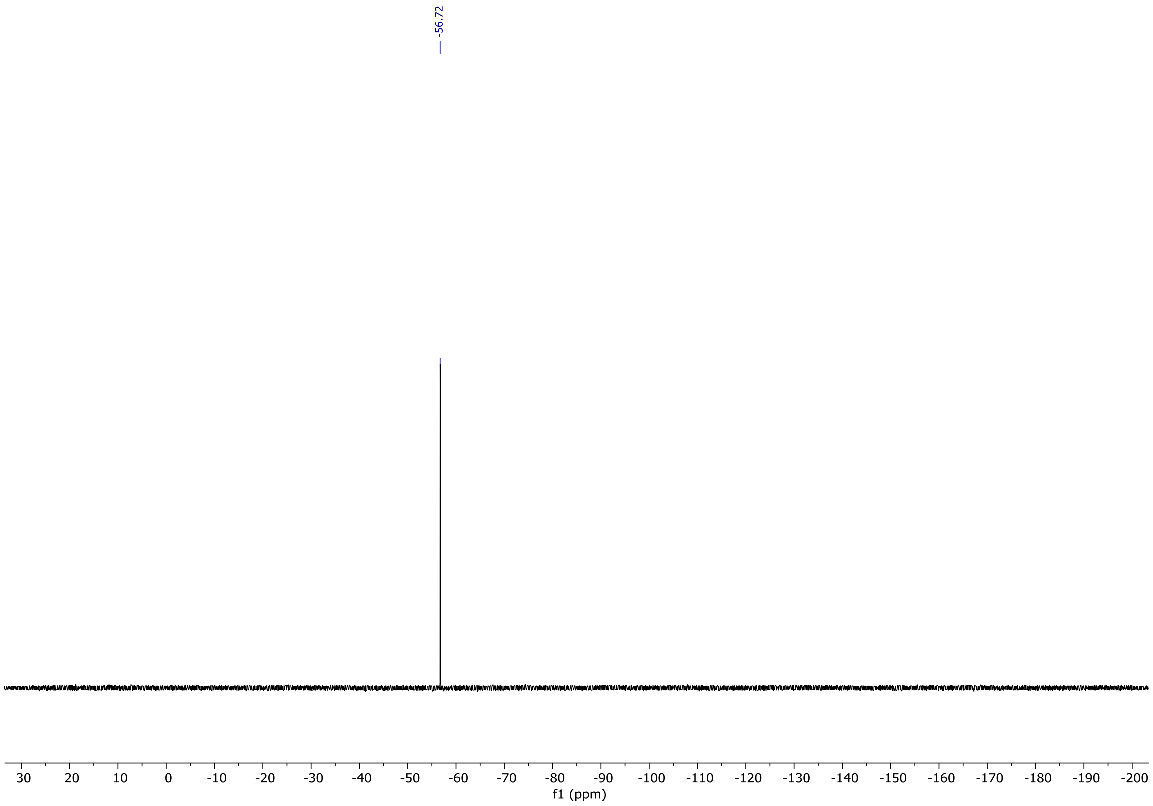

Supplementary Figure 81: ^{19}F NMR for compound **[NBu_4][1g]** in $CDCl_3$

X-Ray Crystallographic Data

Crystallographic data for C8-trifluoromethyl cyclohexadiene-fused sultam 2a

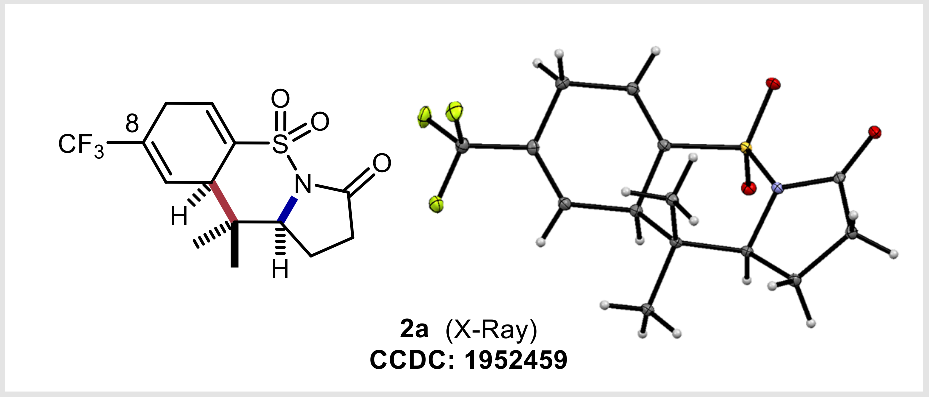

Structural figure of compound **2a**, with 50% probability ellipsoids.

Accession Number The structure of **2a** has been deposited in the Cambridge Crystallographic Data Center under accession number CCDC: 1952459.

Structure Determination:[16]

Colorless needles of **2a** were grown from a dichloromethane/pentane solution of the compound at 23 °C. A crystal of dimensions 0.18 x 0.15 x 0.12 mm was mounted on a Rigaku AFC10K Saturn 944+ CCD-based X-ray diffractometer equipped with a low temperature device and Micromax-007HF Cu-target micro-focus rotating anode (λ = 1.54187 A) operated at 1.2 kW power (40 kV, 30 mA). The X-ray intensities were measured at 85(1) K with the detector placed at a distance 42.00 mm from the crystal. A total of 2028 images were collected with an oscillation width of 1.0° in ω. The exposure times were 1 sec. for the low angle images, 3 sec. for high angle. Rigaku d*trek images were exported to CrysAlisPro for processing and corrected for absorption. The integration of the data yielded a total of 20775 reflections to a maximum 2Θ value of 138.66° of which 2593 were independent and 2538 were greater than 2σ(I). The final cell constants (**Supplemental Table 3**) were based on the xyz centroids of 10883 reflections above 10σ(I). Analysis of the data showed negligible decay during data collection. The structure was solved and refined with the Bruker SHELXTL (version 2018/3) software package, using the space group P2(1)/n with Z = 4 for the formula $C_{14}H_{16}NO_3F_3S$. All non-hydrogen atoms were refined anisotropically with the hydrogen atoms placed in idealized positions. Full matrix least-squares refinement based on F^2 converged at R1 = 0.0371 and wR2 = 0.0952 [based on I > 2σ(I)], R1 = 0.0377 and wR2 = 0.0958 for all data. Acknowledgement is made for funding from NSF grant CHE-0840456 for X-ray instrumentation.

Supplemental Table 3. Crystal data and structure refinement.

**Empirical formula**	C14 H16 F3 N O3 S
**Formula weight**	335.34
**Temperature**	85(2) K
**Wavelength**	1.54184 A
**Crystal system, space group**	Monoclinic, P2(1)/n
**Unit cell dimensions**	a = 11.0713(2) A alpha = 90 deg.
	b = 6.16810(10) A beta = 99.5210(10) deg.
	c = 21.0140(3) A gamma = 90 deg.
**Volume**	1415.26(4) A^3
**Z, Calculated density**	4, 1.574 Mg/m^3
**Absorption coefficient**	2.492 mm^-1
**F(000)**	696
**Crystal size**	0.180 x 0.150 x 0.120 mm
**Theta range for data collection**	4.253 to 69.332 deg.
**Limiting indices**	-13<=h<=12, -7<=k<=7, -25<=l<=25
**Reflections collected / unique**	20775 / 2593 [R(int) = 0.0488]
**Completeness to theta**	= 67.684 98.5 %
**Absorption correction**	Semi-empirical from equivalents
**Max. and min. transmission**	1.00000 and 0.66699
**Refinement method**	Full-matrix least-squares on F^2
**Data / restraints / parameters**	2593 / 0 / 201
**Goodness-of-fit on F^2**	1.069
**Final R indices [I>2sigma(I)]**	R1 = 0.0371, wR2 = 0.0952
**R indices (all data)**	R1 = 0.0377, wR2 = 0.0958
**Extinction coefficient**	n/a
**Largest diff. peak and hole**	0.314 and -0.436 e.A^-3

Crystallographic data for enone 3s

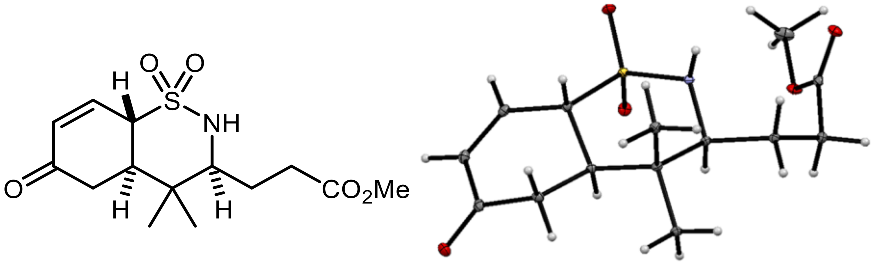

3s (X-Ray)
CCDC: 1984122

Structural figure of compound **3s**, with 50% probability ellipsoids.

The structure of 2a has been deposited in the Cambridge Crystallographic Data Center under accession number CCDC: 1984122.

Structure Determination.

Colorless plates of **3s** were grown from a dichloromethane solution of the compound at 22 °C. A crystal of dimensions 0.24 x 0.20 x 0.20 mm was mounted on a Rigaku AFC10K Saturn 944+ CCD-based X-ray diffractometer equipped with a low temperature device and Micromax-007HF Cu-target micro-focus rotating anode (λ = 1.54187 A) operated at 1.2 kW power (40 kV, 30 mA). The X-ray intensities were measured at 85(1) K with the detector placed at a distance 42.00 mm from the crystal. A total of 2028 images were collected with an oscillation width of 1.0° in ω. The exposure times were 1 sec. for the low angle images, 3 sec. for high angle. Rigaku d*trek images were exported to CrysAlisPro for processing and corrected for absorption. The integration of the data yielded a total of 10616 reflections to a maximum 2Θ value of 139.02° of which 2675 were independent and 2601 were greater than 2σ(I). The final cell constants (**Supplemental Table 4**) were based on the xyz centroids of 6381 reflections above 10σ(I). Analysis of the data showed negligible decay during data collection. The structure was solved and refined with the Bruker SHELXTL (version 2018/3) software package, using the space group P1bar with Z = 2 for the formula $C_{14}H_{21}NO_5S$. All non-hydrogen atoms were refined anisotropically with the hydrogen atoms placed in a combination of idealized and refined positions. Full matrix least-squares refinement based on F^2 converged at R1 = 0.0369 and wR2 = 0.1040 [based on I > 2σ(I)], R1 = 0.0380 and wR2 = 0.1069 for all data. Additional details are presented in Table S4 and are given as Supporting Information in a CIF file. Acknowledgement is made for funding from NSF grant CHE-0840456 for X-ray instrumentation.

Supplemental Table 4. Crystal data and structure refinement.

**Empirical formula**	C14 H21 N O5 S
**Formula weight**	315.38
**Temperature**	85(2) K
**Wavelength**	1.54184 A
**Crystal system, space group**	Triclinic, P-1
**Unit cell dimensions**	a = 7.7850(3) A alpha = 114.012(5) deg.<br>b = 10.0802(5) A beta = 94.736(4) deg.<br>c = 11.0547(6) A gamma = 107.621(4) deg.
**Volume**	733.86(7) A^3
**Z, Calculated density**	2, 1.427 Mg/m^3
**Absorption coefficient**	2.163 mm^-1
**F(000)**	336
**Crystal size**	0.240 x 0.200 x 0.200 mm
**Theta range for data collection**	4.506 to 69.510 deg.
**Limiting indices**	-9<=h<=9, -12<=k<=12, -13<=l<=13
**Reflections collected / unique**	10616 / 2675 [R(int) = 0.0413]
**Completeness to theta** = 67.684	98.3 %
**Absorption correction**	Semi-empirical from equivalents
**Max. and min. transmission**	1.00000 and 0.80825
**Refinement method**	Full-matrix least-squares on F^2
**Data / restraints / parameters**	2675 / 0 / 198

Goodness-of-fit on F^2 1.130

Final R indices [I>2σ(I)] R1 = 0.0369, wR2 = 0.1040

R indices (all data) R1 = 0.0380, wR2 = 0.1069

Extinction coefficient 0.0195(15)

Largest diff. peak and hole 0.362 and -0.437 e.A^-3

Supplementary References

1 Teegardin, K., Day, J. I., Chan, J. & Weaver, J. Advances in Photocatalysis: A Microreview of Visible Light Mediated Ruthenium and Iridium Catalyzed Organic Transformations. *Organic Process Research & Development* **20**, 1156-1163 (2016).

2 Prier, C. K., Rankic, D. A. & MacMillan, D. W. C. Visible Light Photoredox Catalysis with Transition Metal Complexes: Applications in Organic Synthesis. *Chemical Reviews* **113**, 5322-5363 (2013).

3 Luo, J. & Zhang, J. Donor–Acceptor Fluorophores for Visible-Light-Promoted Organic Synthesis: Photoredox/Ni Dual Catalytic C(sp3)–C(sp2) Cross-Coupling. *ACS Catalysis* **6**, 873-877 (2016).

4 Treat, N. J. *et al.* Metal-Free Atom Transfer Radical Polymerization. *Journal of the American Chemical Society* **136**, 16096-16101 (2014).

5 Romero, N. A. & Nicewicz, D. A. Organic Photoredox Catalysis. *Chemical Reviews* **116**, 10075-10166 (2016).

6 Monos, T. M., Sun, A. C., McAtee, R. C., Devery, J. J. & Stephenson, C. R. J. Microwave-Assisted Synthesis of Heteroleptic Ir(III)+ Polypyridyl Complexes. *The Journal of Organic Chemistry* **81**, 6988-6994 (2016).

7 Monos, T. M., McAtee, R. C. & Stephenson, C. R. J. Arylsulfonylacetamides as bifunctional reagents for alkene aminoarylation. *Science* **361**, 1369-1373 (2018).

8 Ladouceur, S., Fortin, D. & Zysman-Colman, E. Enhanced Luminescent Iridium(III) Complexes Bearing Aryltriazole Cyclometallated Ligands. *Inorganic Chemistry* **50**, 11514-11526 (2011).

9 Morton, C. M. *et al.* C–H Alkylation via Multisite-Proton-Coupled Electron Transfer of an Aliphatic C–H Bond. *Journal of the American Chemical Society* **141**, 13253-13260 (2019).

10 Staveness, D. *et al.* Providing a New Aniline Bioisostere through the Photochemical Production of 1-Aminonorbornanes. *Chem* **5**, 215-226 (2019).

11 Huffman, T. R., Wu, Y., Emmerich, A. & Shenvi, R. A. Intermolecular Heck Coupling with Hindered Alkenes Directed by Potassium Carboxylates. *Angewandte Chemie International Edition* **58**, 2371-2376 (2019).

12 Isaksson, R., Kumpiņa, I., Larhed, M. & Wannberg, J. Rapid and straightforward transesterification of sulfonyl carbamates. *Tetrahedron Letters* **57**, 1476-1478 (2016).

13 Beare, K. D. & McErlean, C. S. P. Revitalizing the aromatic aza-Claisen rearrangement: implications for the mechanism of ‘on-water’ catalysis. *Organic & Biomolecular Chemistry* **11**, 2452-2459 (2013).

14 Chantarasriwong, O. *et al.* Evaluation of the pharmacophoric motif of the caged Garciniaxanthones. *Organic & Biomolecular Chemistry* **7**, 4886-4894 (2009).

15 Xu, H. *et al.* Domino Aryne Annulation via a Nucleophilic–Ene Process. *Journal of the American Chemical Society* **140**, 3555-3559 (2018).

16 Sheldrick, G. M. Crystal structure refinement with SHELXL. *Acta crystallographica. Section C, Structural chemistry* **71**, 3-8 (2015).
